# Trends in the global burden of gout attributable to kidney dysfunction, 1990-2021: a population-based analysis

**DOI:** 10.3389/fendo.2025.1677340

**Published:** 2025-10-21

**Authors:** Jiaxian Xu, Mingming Lei, Dandan Xu

**Affiliations:** ^1^ College of Sports Medicine and Health, Chengdu Sport University, Chengdu, China; ^2^ Department of Sports Injury, Affiliated Sports Hospital of Chengdu Sport University, Chengdu, China; ^3^ Department of Intensive Care Unit, The Affiliated Hospital of Xuzhou Medical University, Xuzhou, Jiangsu, China

**Keywords:** gout, kidney dysfunction, disability-adjusted life years (DALYs), public health, socio-demographic index (SDI)

## Abstract

**Background:**

Gout, primarily driven by hyperuricemia, is a prevalent inflammatory arthritis with kidney dysfunction being a significant risk factor. This study aims to comprehensively evaluate the global burden of gout attributable to kidney dysfunction from 1990 to 2021, highlighting differences across regions, genders, Socio-Demographic Index (SDI) levels, and age groups.

**Methods:**

Using data from the Global Burden of Disease (GBD) 2021 study, we assessed the burden of gout due to kidney dysfunction using disability-adjusted life years (DALYs) and years lived with disability (YLDs). Kidney dysfunction was defined as an estimated glomerular filtration rate (eGFR) below 60 mL/min/1.73 m² or an albumin-to-creatinine ratio (ACR) ≥30 mg/g. The data were stratified by region, country, gender, age, and SDI quintiles. The annual percentage change (EAPC) was calculated to determine temporal trends.

**Results:**

From 1990 to 2021, global DALYs and YLDs attributable to gout due to kidney dysfunction increased significantly, rising from 78,564.8 years (95% uncertainty interval 48,687.9–118,061.9) to 200,033.3 years (125,245.0–296,812.4), a 2.5-fold increase. The age-standardized DALY rate (ASDR) and age-standardized YLD rate both increased, from 2.1 per 100,000 population in 1990 to 2.4 per 100,000 in 2021. DALY and YLD rates were consistently higher in males than in females. High SDI countries, especially North America and Australasia, had the most significant rise in burden, whereas low SDI regions experienced a decline. The burden increased with age, particularly in those aged 60 and above.

**Interpretation:**

This study underscores the substantial increase in the global burden of gout attributable to kidney dysfunction, particularly among the elderly, males, and populations in high SDI regions. The observed trends are driven by multiple factors, including lifestyle and dietary habits, diagnostic capacity, and demographic shifts. Future efforts should focus on improving surveillance and access to care in low-SDI regions to address potential underestimation of the burden. High-SDI regions should prioritize personalized health management strategies for the elderly, emphasizing early detection and intervention for gout and kidney dysfunction.

## Introduction

Gout, a common inflammatory arthritis triggered by monosodium urate crystal deposition, is primarily driven by hyperuricemia (1). It is a chronic condition that often recurs, inflicting significant pain and long-term complications on patients (2, 3). The global prevalence and incidence of gout are increasing, in the year 2020, the global prevalence of gout was estimated at 55.8 million individuals, with a 95% uncertainty interval ranging from 44.4 to 69.8 million. The age-standardized prevalence rate was 659.3 per 100,000 population, with an uncertainty interval of 525.4 to 822.3 per 100,000. This represents a 22.5% increase (ranging from 20.9% to 24.2%) since 1990 (4), and the burden of gout is increasing among younger and older populations (5–7). Many factors contribute to gout, including chronic kidney disease, metabolic disorders, genetic predisposition, diet, environment, and seasonal variations, in addition to hyperuricemia (2, 5, 8, 9). Gout is frequently accompanied by comorbidities such as cardiovascular, renal, and metabolic disorders (8). Gout affects more individuals globally than rheumatoid arthritis or lupus (10), in GBD 2017, gout accounted for 2.6% of the total number of patients with musculoskeletal diseases worldwide, while rheumatoid arthritis accounted for only 1.3% (11), however, highly effective treatments remain limited for gout (12), and existing medications have notable side effects (13). The kidneys are pivotal in the excretion of uric acid (14), as approximately 70% of it is eliminated through renal clearance (15), declining renal function (eGFR <60 mL/min/1.73 m²) results in increased serum urate levels (16) and studies demonstrate a significant increase in gout prevalence with declining renal function (17). Studies by Mats Dehlin and colleagues have demonstrated a bidirectional relationship between gout and chronic kidney disease(CKD), wherein CKD is a risk factor for gout, and gout further exacerbates the progression of CKD (5). Gout is prevalent among patients with chronic kidney disease yet it is often underrecognized and inadequately managed as a complication of chronic kidney disease (18). Prior studies have documented the global burden of gout, emphasizing its increasing prevalence and significant health impact. However, few studies have specifically focused on the burden attributable to kidney dysfunction, a critical yet underexplored area. The economic impact and diminished quality of life associated with gout further underscore the need for targeted public health strategies and clinical interventions (19). Therefore, this study aims to comprehensively assess the impact of impaired kidney function on the gout burden across global, regional, and national populations. By examining differences across regions, genders, and Socio-Demographic Index (SDI) levels, this study elucidates the relationship between impaired kidney function and gout, offering a scientific basis for targeted public health strategies and clinical interventions.

## Methods

### Data collection

In this study, we assessed the burden of gout attributable to kidney dysfunction using the Global Burden of Disease (GBD) 2021 study. The analysis followed the comparative risk assessment (CRA) framework. The risk–outcome pair was: kidney dysfunction → gout. Relative risks (RRs) were drawn from systematic reviews and meta-regression performed by GBD, as detailed in the kidney dysfunction risk factor methods paper. Exposure definition: kidney dysfunction was defined as an estimated glomerular filtration rate (eGFR) below 60 mL/min/1.73 m^2^ or an albumin-to-creatinine ratio (ACR) greater than or equal to 30 mg/g (4). The theoretical minimum risk exposure level (TMREL) was defined as eGFR ≥60 mL/min/1.73 m² and ACR <30 mg/g. Population attributable fractions (PAFs) were used as provided by the GBD study and were not recomputed independently.

The primary data source for our analysis was the Global Health Data Exchange (GHDx) (http://ghdx.healthdata.org/), a comprehensive repository of global health-related data. The data were sourced from the GBD 2021 study, which employs a comprehensive methodology to harmonize data across different coding systems. Specifically, the GBD study uses both ICD-10 codes and their corresponding ICD-9-CM codes (274.0 for gouty arthritis and 274.1 for other and unspecified gout) to ensure consistency and comparability of data over time and across regions. This harmonization process includes the use of correction factors and imputation techniques to account for any discrepancies that may arise due to coding changes, particularly during the transition period from ICD-9 to ICD-10. Using DisMod-MR 2.1, a Bayesian meta-regression tool, the non-fatal burden of gout was modeled, with the assumption that gout does not lead to increased mortality or complete remission. Estimates for prevalence and yearly incidence were produced based on age, gender, location, and year. The model incorporated a single country-specific covariate, the gout summary exposure value (SEV) scalar, which is a normalized measure of disease risks. For gout, the risks include kidney dysfunction and high BMI.

The data extraction process involved querying the GHDx database with the following parameters: cause of disease selected as “gout”; risk factor selected as “kidney dysfunction”; measurement indicators selected as “years lived with disability (YLDs), disability-adjusted life years (DALYs)”; geographical location selected as “all regions”; and time range selected as “1990-2021”. Data were standardized to ensure comparability across different regions and time periods. We adhered to the Guidelines for Accurate and Transparent Health Estimates Reporting (GATHER) to enhance the rigor and transparency of the data collection and analysis process.

### Data extraction

All estimates were obtained from the Global Health Data Exchange (GHDx, http://ghdx.healthdata.org/). We queried: cause = gout; risk factor = kidney dysfunction; measures = DALYs and YLDs; location = all 204 countries and territories; years = 1990–2021. Query IDs: GHDx-GBD2021-KD-Gout-20231012–001 to -003. Data were downloaded on October 12, 2024. Age groups followed the standard GBD 5-year intervals from 0–4 to ≥95 years. Rates were age-standardized using the GBD 2021 world standard population.

### Socio-demographic index

In this study, we utilized the Socio-Demographic Index (SDI) to classify countries and regions according to their level of development. The SDI is a composite measure developed by the Institute for Health Metrics and Evaluation (IHME) https://vizhub.healthdata.org/gbd-compare/ that integrates income per capita, average years of education, and total fertility rate (TFR) into a single metric. The Socio-demographic Index (SDI) serves as a composite indicator of development, incorporating a country’s total fertility rate among women under 25 years, educational attainment levels, and lag-distributed per capita income. The SDI is derived from three principal factors: per capita income, the average educational attainment of individuals aged 15 years and older, and fertility rates among women under the age of 25. The SDI ranges from 0 to 1, with higher values indicating higher levels of development. According to the 2021 SDI data, 204 countries and regions were categorized into five distinct groups: low SDI (<0.466), low-middle SDI (0.466–0.619), middle SDI (0.619–0.720), high-middle SDI (0.720–0.810), and high SDI (≥0.810) (20).

### Uncertainty handling

All estimates were reported with 95% uncertainty intervals (UIs), derived from the 1000 draws in the GBD posterior distribution. For derived metrics (e.g., age-standardized rates, EAPC), draws were propagated consistently, ensuring numerator (attributable DALYs/YLDs) and denominator (population) were matched. Statistical significance was judged only by whether the 95% CI of EAPC excluded zero; we avoided interpreting overlapping UIs as hypothesis tests.

### Statistical analysis

We used data from the Global Burden of Disease (GBD) study to describe the burden of gout attributable to kidney dysfunction at the global, regional, and national levels from 1990 to 2021. The primary analysis indicators were disability-adjusted life years (DALYs) and years lived with disability (YLDs). Because GBD does not attribute deaths to gout, YLLs were assumed to be zero; DALYs therefore equal YLDs. We state that age-standardized rates use the GBD 2021 world standard population. We calculated the age-standardized DALY rate (ASDR) and age-standardized YLD rate for gout attributable to kidney dysfunction in these regions and generated world maps to visually display their distribution. The annual percentage change (EAPC) was calculated using the formula 100×(exp(β)-1), where β represents the coefficient in the linear regression model. EAPC diagnostics: We specify the log-linear model, residual diagnostics, and Monte-Carlo uncertainty propagation. The 95% confidence interval (CI) was also derived from the linear regression model. We calculated the EAPC of ASDR and the EAPC of age-standardized YLD rate for gout attributable to impaired kidney function, at global, regional, and national scales. These estimates were mapped geospatially to depict temporal trends across countries and territories. World maps were produced in package sf using the Eckert IV equal-area projection and quantile classification (k = 7) for visual clarity; shapefiles were sourced from Natural Earth (v5.1.2). In addition, we compared the age-specific burden composition of gout attributable to kidney dysfunction in 1990 and 2021 and assessed changes in the age structure of the disease during the study period. All statistical analyses were completed using R software (version 4.4.3) with the assistance of packages including ggplot2 (v3.5.1), sf (v1.0-16), terra (v1.7-78), survey (v4.4), and mrbrt (v1.0). etc.

### Mapping and visualization

World maps were produced using the Eckert IV equal-area projection with quantile classification (k = 7) to visualize geographic heterogeneity. Because quantile breaks can overemphasize small differences, we also presented absolute numbers and uncertainty ribbons for temporal trends in **Supplementary Figures**. Maps were generated using the R package sf, and shapefiles were sourced from Natural Earth (v5.1.2). This approach allowed us to effectively communicate the spatial distribution of gout burden attributable to kidney dysfunction across different regions and time periods.

### Handling missing or sparse data

To address the issue of missing or sparse data, the GBD study employed a combination of methods. For countries with limited surveillance infrastructure, covariate-driven regression models were used to estimate disease burden. These models incorporated socioeconomic status, healthcare access, and lifestyle factors to improve accuracy. Imputation techniques were applied to fill gaps in data, based on information from neighboring countries or regions with similar profiles. Additionally, Bayesian statistical methods were used to integrate prior knowledge and quantify uncertainty in the estimates. Sensitivity analyses were conducted to assess the impact of missing data on the results. These approaches ensured that the estimates were robust and representative, even in regions with limited data availability.

## Results

### Global burden of gout attributable to kidney dysfunction from 1990 to 2021: temporal trends and gender disparities

**Figure 1:**
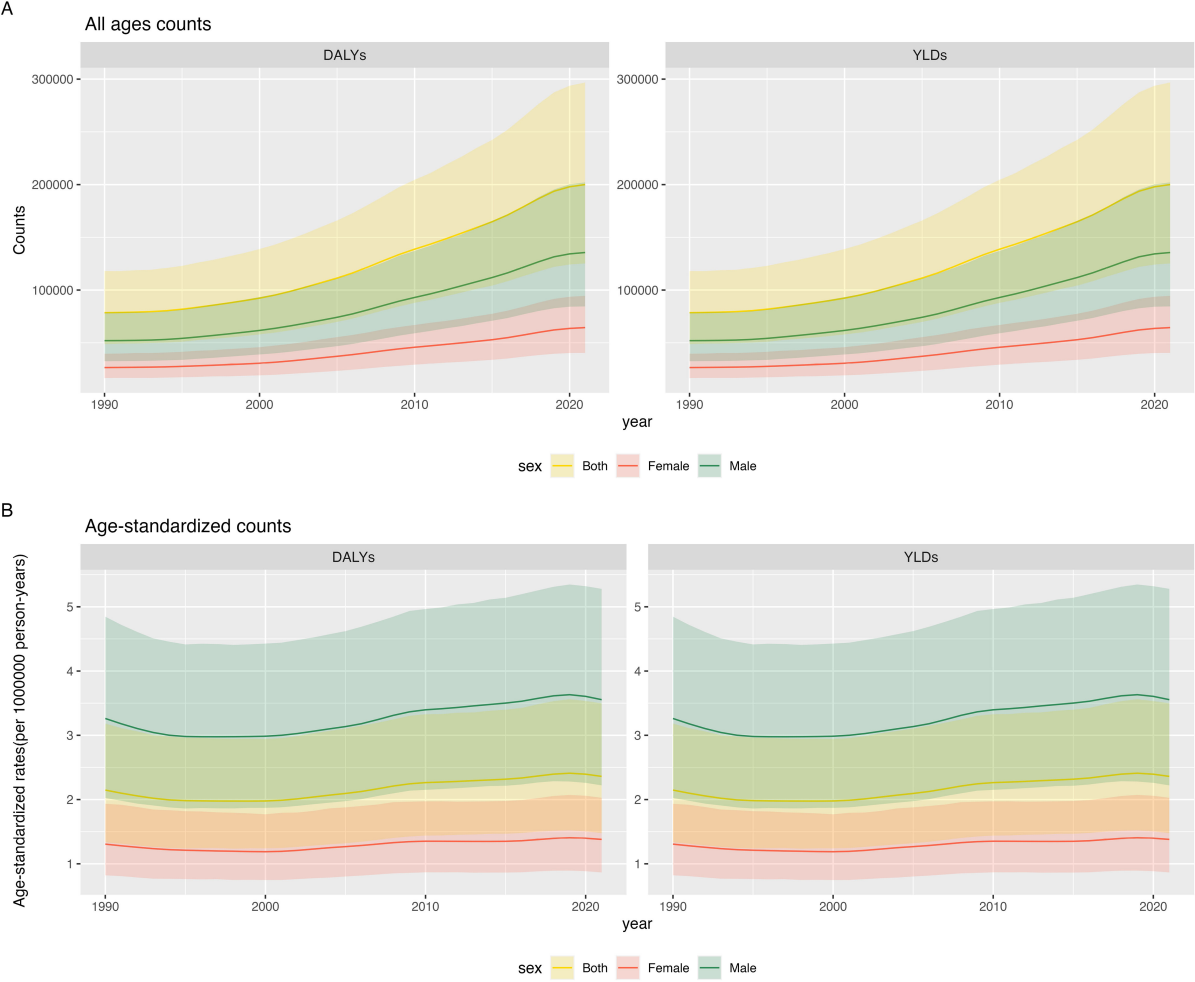
Global burden of gout attributable to kidney dysfunction from 1990 to 2021: temporal trends and sex differences: **(A)** Counts **(B)** Age-standardized rates.

From 1990 to 2021, global DALYs and YLDs attributable to gout due to kidney dysfunction increased significantly, rising from 78,564.8 years (95% uncertainty interval 48,687.9–118,061.9) to 200,033.3 years (125,245.0–296,812.4), a 2.5-fold increase. Non-overlapping 95% UIs indicate that this increase is statistically significant. DALYs and YLDs were consistently higher and grew more rapidly in males than females (**Figure 1A**, **Appendix Tables pp 1** in **Supplementary Material**). The age-standardized DALY rate (ASDR) and age-standardized YLD rate both increased, rising from 2.1 years per 100,000 population in 1990 (95% uncertainty interval 1.3–3.2) to 2.4 years per 100,000 population in 2021 (95% uncertainty interval 1.5–3.5)(Non-overlapping 95% UIs confirm a statistically significant temporal rise) (**Figure 1B**). In 2021, the ASDR and YLD rate in males were 2.6 times higher than those in females: 3.6 years per 100,000 population in males (95% uncertainty interval 2.2–5.3) and 1.4 years per 100,000 population in females (95% uncertainty interval 0.9–2.0) (Non-overlapping 95% UIs demonstrate a statistically significant sex disparity) (**Appendix Tables pp 1–2** in **Supplementary Material**).

### Region and sex trends of gout attributable to kidney dysfunction from 1990 to 2021

From 1990 to 2021, the burden of gout attributable to kidney dysfunction varied by region and development level. In high and high-middle SDI countries, DALYs and YLDs due to gout from kidney dysfunction increased, with the most significant rise in high SDI countries (EAPC of ASDR and age-standardized YLD rate: 1.61%, 95% CI 1.43–1.80; 95% CI excludes 0, indicating a statistically significant increase). The age-standardized DALY and YLD rates rose from 2.9 (1.8-4.2) per 100,000 in 1990 to 3.9 (2.5-5.8) per 100,000 in 2021. Conversely, low and low-middle SDI countries saw a decline in DALYs, with the most pronounced decrease in low SDI countries (EAPC of ASDR and age-standardized YLD rate: −0.09%, 95% CI −0.11 to −0.07; 95% CI excludes 0, indicating a statistically significant decrease). At the regional level, gout burden from kidney dysfunction showed divergent trends. High-income North America experienced the largest increase in age-standardized DALY and YLD rates (EAPC 2.93%, 95% CI 2.51–3.35), while South Asia saw the most significant decline (EAPC −0.21%, 95% CI −0.27 to −0.16). Notably, East Asia, Central Europe, and Oceania had minimal changes (EAPC: 0.13 [-0.1-0.35], 0.11 [0.04-0.17], and 0.24 [0.22-0.25], respectively, 95% CIs crossed 0, non-significant changes). Across all regions, male burden remained statistically significantly higher than female burden, supported by non-overlapping 95% UIs. (**Table 1**). These divergent trajectories likely reflect differential CKD detection capacity, uric-acid screening policies, and life expectancy: high-SDI settings benefit from heightened surveillance and longer survival with comorbid CKD, while low-SDI regions face under-diagnosis and younger population structures.

**Table 1:** Regional age-standardized DALY rate and age-standardized YLD rate of gout due to renal dysfunction from 1990 to 2021, and associated EAPC.

Location	Sex	DALY (95%UI)		DALY (95%UI)			YLDs (95%UI)		YLDs (95%UI)		
		Case, 1990	ASDR (per100000),	Case, 2021	ASDR (per100000),	EAPC of ASDR (95%CI),	Case, 1990	AS YLD rate (per100000),	Case, 2021	AS YLD rate (per100000),	EAPC of AS YLD rate (95%CI),
			1990		2021	1990-2021		1990		2021	1990-2021
Global	Both	78564.8 (48687.9-118061.9)	2.1 (1.3-3.2)	200033.3 (125245-296812.4)	2.4 (1.5-3.5)	0.67 (0.54-0.8)	78564.8 (48687.9-118061.9)	2.1 (1.3-3.2)	200033.3 (125245-296812.4)	2.4 (1.5-3.5)	0.67 (0.54-0.8)
	Male	52051.6 (32525.3-78861.1)	3.3 (2-4.8)	135608.1 (84507.3-202098.1)	3.6 (2.2-5.3)	0.66 (0.53-0.8)	52051.6 (32525.3-78861.1)	3.3 (2-4.8)	135608.1 (84507.3-202098.1)	3.6 (2.2-5.3)	0.66 (0.53-0.8)
	Female	26513.3 (16593.3-39608.6)	1.3 (0.8-1.9)	64425.3 (40432.1-94545.4)	1.4 (0.9-2)	0.49 (0.37-0.62)	26513.3 (16593.3-39608.6)	1.3 (0.8-1.9)	64425.3 (40432.1-94545.4)	1.4 (0.9-2)	0.49 (0.37-0.62)
High SDI	Both	32380.1 (19958.8-48176.1)	2.9 (1.8-4.2)	87694.9 (55433-129143.2)	3.9 (2.5-5.8)	1.61 (1.43-1.8)	32380.1 (19958.8-48176.1)	2.9 (1.8-4.2)	87694.9 (55433-129143.2)	3.9 (2.5-5.8)	1.61 (1.43-1.8)
	Male	20704 (12743.9-31353.1)	4.6 (2.9-6.9)	58744.6 (37472-87683.5)	6 (3.8-8.9)	1.47 (1.27-1.67)	20704 (12743.9-31353.1)	4.6 (2.9-6.9)	58744.6 (37472-87683.5)	6 (3.8-8.9)	1.47 (1.27-1.67)
	Female	11676.2 (7242.2-17405.1)	1.7 (1-2.5)	28950.3 (18251.1-42090.8)	2.2 (1.4-3.2)	1.39 (1.22-1.56)	11676.2 (7242.2-17405.1)	1.7 (1-2.5)	28950.3 (18251.1-42090.8)	2.2 (1.4-3.2)	1.39 (1.22-1.56)
High-middle SDI	Both	17078.5 (10597.2-25793.7)	1.8 (1.1-2.7)	37072 (22995.9-55599)	1.9 (1.2-2.8)	0.28 (0.18-0.38)	17078.5 (10597.2-25793.7)	1.8 (1.1-2.7)	37072 (22995.9-55599)	1.9 (1.2-2.8)	0.28 (0.18-0.38)
	Male	10852.3 (6726.6-16603)	2.9 (1.8-4.3)	24349.6 (14936.9-36627.5)	2.9 (1.8-4.3)	0.18 (0.07-0.29)	10852.3 (6726.6-16603)	2.9 (1.8-4.3)	24349.6 (14936.9-36627.5)	2.9 (1.8-4.3)	0.18 (0.07-0.29)
	Female	6226.2 (3852.4-9451.6)	1.1 (0.7-1.7)	12722.4 (7881.5-18879.6)	1.1 (0.7-1.7)	0.13 (0.04-0.23)	6226.2 (3852.4-9451.6)	1.1 (0.7-1.7)	12722.4 (7881.5-18879.6)	1.1 (0.7-1.7)	0.13 (0.04-0.23)
Middle SDI	Both	17405 (10849.9-26203.2)	1.9 (1.2-2.8)	47213.8 (29276-70757.4)	1.8 (1.1-2.8)	0.24 (0.1-0.38)	17405 (10849.9-26203.2)	1.9 (1.2-2.8)	47213.8 (29276-70757.4)	1.8 (1.1-2.8)	0.24 (0.1-0.38)
	Male	11939.1 (7395.5-18081.3)	2.7 (1.7-4.1)	32700.5 (20255.9-49010.9)	2.8 (1.7-4.1)	0.32 (0.18-0.46)	11939.1 (7395.5-18081.3)	2.7 (1.7-4.1)	32700.5 (20255.9-49010.9)	2.8 (1.7-4.1)	0.32 (0.18-0.46)
	Female	5465.9 (3408.1-8204.2)	1.1 (0.7-1.7)	14513.3 (9071-21939.6)	1.1 (0.7-1.6)	0.09 (-0.08-0.25)	5465.9 (3408.1-8204.2)	1.1 (0.7-1.7)	14513.3 (9071-21939.6)	1.1 (0.7-1.6)	0.09 (-0.08-0.25)
Low-middle SDI	Both	8588.7 (5424.4-12917.1)	1.6 (1-2.4)	21156.8 (13242.8-31826.2)	1.6 (1-2.4)	-0.01 (-0.04-0.02)	8588.7 (5424.4-12917.1)	1.6 (1-2.4)	21156.8 (13242.8-31826.2)	1.6 (1-2.4)	-0.01 (-0.04-0.02)
	Male	6244.2 (3945.9-9411.8)	2.3 (1.5-3.4)	14814.5 (9309.6-22275.8)	2.4 (1.5-3.6)	0.09 (0.06-0.12)	6244.2 (3945.9-9411.8)	2.3 (1.5-3.4)	14814.5 (9309.6-22275.8)	2.4 (1.5-3.6)	0.09 (0.06-0.12)
	Female	2344.5 (1469.9-3516.9)	0.9 (0.6-1.3)	6342.3 (4012.6-9514.4)	0.9 (0.6-1.4)	0.11 (0.06-0.16)	2344.5 (1469.9-3516.9)	0.9 (0.6-1.3)	6342.3 (4012.6-9514.4)	0.9 (0.6-1.4)	0.11 (0.06-0.16)
Low SDI	Both	3057.1 (1919.8-4610.1)	1.6 (1-2.3)	6781.9 (4255.5-10238.4)	1.5 (1-2.3)	-0.09 (-0.11--0.07)	3057.1 (1919.8-4610.1)	1.6 (1-2.3)	6781.9 (4255.5-10238.4)	1.5 (1-2.3)	-0.09 (-0.11--0.07)
	Male	2275.5 (1425.9-3448.4)	2.3 (1.5-3.4)	4922.8 (3084-7424.4)	2.3 (1.4-3.4)	-0.04 (-0.05--0.02)	2275.5 (1425.9-3448.4)	2.3 (1.5-3.4)	4922.8 (3084-7424.4)	2.3 (1.4-3.4)	-0.04 (-0.05--0.02)
	Female	781.6 (494.6-1173.5)	0.8 (0.5-1.2)	1859.1 (1185.1-2762.5)	0.8 (0.5-1.2)	0 (-0.03-0.03)	781.6 (494.6-1173.5)	0.8 (0.5-1.2)	1859.1 (1185.1-2762.5)	0.8 (0.5-1.2)	0 (-0.03-0.03)
Andean Latin America	Both	114.6 (72.7-172.5)	0.6 (0.4-0.9)	430.3 (265.9-652)	0.7 (0.5-1.1)	0.85 (0.81-0.9)	114.6 (72.7-172.5)	0.6 (0.4-0.9)	430.3 (265.9-652)	0.7 (0.5-1.1)	0.85 (0.81-0.9)
	Male	75.5 (47.8-111.7)	0.8 (0.5-1.2)	286.1 (176.8-430.3)	1.1 (0.6-1.6)	0.98 (0.92-1.04)	75.5 (47.8-111.7)	0.8 (0.5-1.2)	286.1 (176.8-430.3)	1.1 (0.6-1.6)	0.98 (0.92-1.04)
	Female	39.1 (24.2-59.5)	0.4 (0.2-0.6)	144.1 (87.7-222.6)	0.5 (0.3-0.7)	0.62 (0.59-0.64)	39.1 (24.2-59.5)	0.4 (0.2-0.6)	144.1 (87.7-222.6)	0.5 (0.3-0.7)	0.62 (0.59-0.64)
Australasia	Both	1002.5 (628.4-1505.1)	4.1 (2.6-6.2)	3050.5 (1854.2-4702.7)	5.3 (3.2-8.1)	0.9 (0.84-0.96)	1002.5 (628.4-1505.1)	4.1 (2.6-6.2)	3050.5 (1854.2-4702.7)	5.3 (3.2-8.1)	0.9 (0.84-0.96)
	Male	599.9 (384.3-910.8)	6 (3.8-9)	1955.6 (1203.1-3025.5)	7.3 (4.5-11.3)	0.84 (0.78-0.91)	599.9 (384.3-910.8)	6 (3.8-9)	1955.6 (1203.1-3025.5)	7.3 (4.5-11.3)	0.84 (0.78-0.91)
	Female	402.6 (243.7-608.1)	2.8 (1.7-4.3)	1094.9 (665.8-1645.8)	3.4 (2-5.1)	0.64 (0.58-0.71)	402.6 (243.7-608.1)	2.8 (1.7-4.3)	1094.9 (665.8-1645.8)	3.4 (2-5.1)	0.64 (0.58-0.71)
Caribbean	Both	155 (94.2-232.5)	0.6 (0.4-0.9)	413 (262-621.7)	0.8 (0.5-1.2)	0.67 (0.64-0.69)	155 (94.2-232.5)	0.6 (0.4-0.9)	413 (262-621.7)	0.8 (0.5-1.2)	0.67 (0.64-0.69)
	Male	108.1 (65.6-164.3)	0.9 (0.6-1.4)	281.9 (176.6-421.2)	1.1 (0.7-1.7)	0.69 (0.66-0.72)	108.1 (65.6-164.3)	0.9 (0.6-1.4)	281.9 (176.6-421.2)	1.1 (0.7-1.7)	0.69 (0.66-0.72)
	Female	46.9 (28.4-70.5)	0.4 (0.2-0.5)	131.1 (81.3-199.9)	0.4 (0.3-0.7)	0.71 (0.69-0.74)	46.9 (28.4-70.5)	0.4 (0.2-0.5)	131.1 (81.3-199.9)	0.4 (0.3-0.7)	0.71 (0.69-0.74)
Central Asia	Both	952.9 (599.9-1422.8)	2.2 (1.4-3.2)	1887.4 (1192.8-2833)	2.5 (1.6-3.7)	0.52 (0.5-0.54)	952.9 (599.9-1422.8)	2.2 (1.4-3.2)	1887.4 (1192.8-2833)	2.5 (1.6-3.7)	0.52 (0.5-0.54)
	Male	563.7 (358.6-843.9)	3.4 (2.1-5.1)	1191.3 (749.9-1796)	3.8 (2.4-5.6)	0.36 (0.33-0.39)	563.7 (358.6-843.9)	3.4 (2.1-5.1)	1191.3 (749.9-1796)	3.8 (2.4-5.6)	0.36 (0.33-0.39)
	Female	389.2 (248.4-581.6)	1.4 (0.9-2.1)	696.2 (440.1-1051.5)	1.6 (1-2.5)	0.41 (0.4-0.42)	389.2 (248.4-581.6)	1.4 (0.9-2.1)	696.2 (440.1-1051.5)	1.6 (1-2.5)	0.41 (0.4-0.42)
Central Europe	Both	1750.5 (1074.2-2604.9)	1.2 (0.8-1.8)	3035.1 (1883.2-4530.3)	1.3 (0.8-2)	0.16 (0.13-0.19)	1750.5 (1074.2-2604.9)	1.2 (0.8-1.8)	3035.1 (1883.2-4530.3)	1.3 (0.8-2)	0.16 (0.13-0.19)
	Male	1101.3 (673.7-1641.4)	1.9 (1.2-2.8)	1859.4 (1150.2-2748.5)	2 (1.2-2.9)	0.05 (0.02-0.07)	1101.3 (673.7-1641.4)	1.9 (1.2-2.8)	1859.4 (1150.2-2748.5)	2 (1.2-2.9)	0.05 (0.02-0.07)
	Female	649.2 (401.4-967)	0.8 (0.5-1.1)	1175.6 (733-1753.1)	0.8 (0.5-1.3)	0.22 (0.19-0.25)	649.2 (401.4-967)	0.8 (0.5-1.1)	1175.6 (733-1753.1)	0.8 (0.5-1.3)	0.22 (0.19-0.25)
Central Latin America	Both	473 (298.2-703.5)	0.6 (0.4-0.9)	1848.5 (1159.4-2793.9)	0.7 (0.5-1.1)	0.88 (0.8-0.97)	473 (298.2-703.5)	0.6 (0.4-0.9)	1848.5 (1159.4-2793.9)	0.7 (0.5-1.1)	0.88 (0.8-0.97)
	Male	281.7 (174.5-424)	0.7 (0.5-1.1)	1091 (679.6-1638.4)	1 (0.6-1.4)	1.02 (0.94-1.1)	281.7 (174.5-424)	0.7 (0.5-1.1)	1091 (679.6-1638.4)	1 (0.6-1.4)	1.02 (0.94-1.1)
	Female	191.3 (119.4-293.1)	0.5 (0.3-0.7)	757.5 (487.5-1174.4)	0.6 (0.4-0.9)	0.79 (0.69-0.88)	191.3 (119.4-293.1)	0.5 (0.3-0.7)	757.5 (487.5-1174.4)	0.6 (0.4-0.9)	0.79 (0.69-0.88)
Central Sub-Saharan Africa	Both	465.2 (295.3-695.9)	2.5 (1.6-3.6)	1173.2 (743.7-1752.3)	2.4 (1.5-3.6)	-0.11 (-0.16--0.05)	465.2 (295.3-695.9)	2.5 (1.6-3.6)	1173.2 (743.7-1752.3)	2.4 (1.5-3.6)	-0.11 (-0.16--0.05)
	Male	354.1 (223.4-527.6)	4 (2.5-5.8)	875.7 (547.6-1322.3)	4.2 (2.6-6.3)	0.09 (0.05-0.12)	354.1 (223.4-527.6)	4 (2.5-5.8)	875.7 (547.6-1322.3)	4.2 (2.6-6.3)	0.09 (0.05-0.12)
	Female	111 (68.9-171)	1.1 (0.7-1.7)	297.5 (189.4-447.9)	1.2 (0.7-1.7)	0.03 (-0.02-0.07)	111 (68.9-171)	1.1 (0.7-1.7)	297.5 (189.4-447.9)	1.2 (0.7-1.7)	0.03 (-0.02-0.07)
East Asia	Both	17153.6 (10626.4-25883.3)	2.2 (1.4-3.3)	42351.9 (25943.1-63986.4)	2 (1.2-3)	0.13 (-0.1-0.35)	17153.6 (10626.4-25883.3)	2.2 (1.4-3.3)	42351.9 (25943.1-63986.4)	2 (1.2-3)	0.13 (-0.1-0.35)
	Male	11410.5 (6993.9-17306.1)	3.2 (2-4.8)	29009 (17815.9-43823.1)	3 (1.8-4.4)	0.22 (0-0.45)	11410.5 (6993.9-17306.1)	3.2 (2-4.8)	29009 (17815.9-43823.1)	3 (1.8-4.4)	0.22 (0-0.45)
	Female	5743 (3524.4-8684.6)	1.4 (0.9-2.2)	13342.9 (8208.1-20242.9)	1.2 (0.7-1.8)	-0.15 (-0.41-0.11)	5743 (3524.4-8684.6)	1.4 (0.9-2.2)	13342.9 (8208.1-20242.9)	1.2 (0.7-1.8)	-0.15 (-0.41-0.11)
Eastern Europe	Both	3779.2 (2354.1-5702.4)	1.4 (0.9-2.1)	5660.7 (3571.5-8407.2)	1.6 (1-2.3)	0.44 (0.41-0.46)	3779.2 (2354.1-5702.4)	1.4 (0.9-2.1)	5660.7 (3571.5-8407.2)	1.6 (1-2.3)	0.44 (0.41-0.46)
	Male	1994.7 (1256.9-3012.3)	2.5 (1.5-3.6)	3085.4 (1920.5-4608.3)	2.5 (1.6-3.7)	0.13 (0.11-0.16)	1994.7 (1256.9-3012.3)	2.5 (1.5-3.6)	3085.4 (1920.5-4608.3)	2.5 (1.6-3.7)	0.13 (0.11-0.16)
	Female	1784.6 (1120.3-2683.2)	1 (0.6-1.5)	2575.3 (1633.3-3807.3)	1.1 (0.7-1.6)	0.45 (0.42-0.49)	1784.6 (1120.3-2683.2)	1 (0.6-1.5)	2575.3 (1633.3-3807.3)	1.1 (0.7-1.6)	0.45 (0.42-0.49)
Eastern Sub-Saharan Africa	Both	607.3 (381.3-931.9)	1 (0.6-1.5)	1350.4 (839.6-2047.6)	0.9 (0.6-1.4)	-0.02 (-0.07-0.03)	607.3 (381.3-931.9)	1 (0.6-1.5)	1350.4 (839.6-2047.6)	0.9 (0.6-1.4)	-0.02 (-0.07-0.03)
	Male	443.2 (277.3-683.5)	1.4 (0.9-2.2)	942.9 (582.7-1425.9)	1.4 (0.9-2.2)	0.08 (0.02-0.14)	443.2 (277.3-683.5)	1.4 (0.9-2.2)	942.9 (582.7-1425.9)	1.4 (0.9-2.2)	0.08 (0.02-0.14)
	Female	164.1 (102.1-250.1)	0.5 (0.3-0.8)	407.5 (253.7-621)	0.5 (0.3-0.8)	0.08 (0.03-0.13)	164.1 (102.1-250.1)	0.5 (0.3-0.8)	407.5 (253.7-621)	0.5 (0.3-0.8)	0.08 (0.03-0.13)
High-income Asia Pacific	Both	5494.2 (3389.8-8250.1)	2.8 (1.7-4.2)	15282.9 (9351.8-22822)	3 (1.8-4.5)	0.25 (0.22-0.28)	5494.2 (3389.8-8250.1)	2.8 (1.7-4.2)	15282.9 (9351.8-22822)	3 (1.8-4.5)	0.25 (0.22-0.28)
	Male	3776.9 (2336.5-5725.6)	4.6 (2.9-6.9)	10543.1 (6488.3-15779.7)	4.8 (3-7.3)	0.15 (0.12-0.19)	3776.9 (2336.5-5725.6)	4.6 (2.9-6.9)	10543.1 (6488.3-15779.7)	4.8 (3-7.3)	0.15 (0.12-0.19)
	Female	1717.3 (1076.7-2585.5)	1.5 (0.9-2.2)	4739.8 (2976.9-7161.4)	1.4 (0.9-2.1)	-0.07 (-0.09--0.05)	1717.3 (1076.7-2585.5)	1.5 (0.9-2.2)	4739.8 (2976.9-7161.4)	1.4 (0.9-2.1)	-0.07 (-0.09--0.05)
High-income North America	Both	15129 (9374.1-22498.3)	4.1 (2.6-6.1)	47760.1 (31073.7-69750.6)	6.9 (4.5-10.1)	2.93 (2.51-3.35)	15129 (9374.1-22498.3)	4.1 (2.6-6.1)	47760.1 (31073.7-69750.6)	6.9 (4.5-10.1)	2.93 (2.51-3.35)
	Male	9634.8 (5983.1-14630.6)	6.5 (4.1-9.8)	31758.1 (20706.6-46610.3)	10.4 (6.8-15.2)	2.82 (2.37-3.27)	9634.8 (5983.1-14630.6)	6.5 (4.1-9.8)	31758.1 (20706.6-46610.3)	10.4 (6.8-15.2)	2.82 (2.37-3.27)
	Female	5494.2 (3408.2-8144.4)	2.4 (1.5-3.6)	16002 (10185.7-23495.2)	4 (2.6-5.9)	2.71 (2.33-3.08)	5494.2 (3408.2-8144.4)	2.4 (1.5-3.6)	16002 (10185.7-23495.2)	4 (2.6-5.9)	2.71 (2.33-3.08)
North Africa and Middle East	Both	2585.7 (1616.2-3915.9)	1.7 (1.1-2.5)	8029.2 (5053.8-12070.8)	1.9 (1.2-2.9)	0.44 (0.4-0.47)	2585.7 (1616.2-3915.9)	1.7 (1.1-2.5)	8029.2 (5053.8-12070.8)	1.9 (1.2-2.9)	0.44 (0.4-0.47)
	Male	1841.8 (1153.9-2781.3)	2.4 (1.5-3.6)	5648.3 (3550.3-8495.8)	2.7 (1.7-4)	0.39 (0.35-0.43)	1841.8 (1153.9-2781.3)	2.4 (1.5-3.6)	5648.3 (3550.3-8495.8)	2.7 (1.7-4)	0.39 (0.35-0.43)
	Female	743.9 (462.7-1129.3)	1 (0.6-1.5)	2380.9 (1503.4-3570.3)	1.2 (0.7-1.7)	0.5 (0.47-0.53)	743.9 (462.7-1129.3)	1 (0.6-1.5)	2380.9 (1503.4-3570.3)	1.2 (0.7-1.7)	0.5 (0.47-0.53)
Oceania	Both	54.1 (34.6-81.9)	2.3 (1.4-3.4)	150.8 (93.8-227)	2.4 (1.5-3.6)	0.24 (0.22-0.25)	54.1 (34.6-81.9)	2.3 (1.4-3.4)	150.8 (93.8-227)	2.4 (1.5-3.6)	0.24 (0.22-0.25)
	Male	40.9 (25.8-61.7)	3.3 (2.1-4.9)	113.3 (68.9-173)	3.5 (2.2-5.3)	0.17 (0.15-0.2)	40.9 (25.8-61.7)	3.3 (2.1-4.9)	113.3 (68.9-173)	3.5 (2.2-5.3)	0.17 (0.15-0.2)
	Female	13.2 (8.3-20)	1.2 (0.8-1.8)	37.4 (23.5-56.1)	1.3 (0.8-1.9)	0.29 (0.26-0.32)	13.2 (8.3-20)	1.2 (0.8-1.8)	37.4 (23.5-56.1)	1.3 (0.8-1.9)	0.29 (0.26-0.32)
South Asia	Both	8131.1 (5155.3-12243.9)	1.7 (1-2.4)	20854.9 (13089.4-31522.8)	1.6 (1-2.3)	-0.21 (-0.27--0.16)	8131.1 (5155.3-12243.9)	1.7 (1-2.4)	20854.9 (13089.4-31522.8)	1.6 (1-2.3)	-0.21 (-0.27--0.16)
	Male	5972.4 (3783.2-9041.3)	2.3 (1.5-3.5)	14530.9 (9078.3-21959)	2.3 (1.4-3.4)	-0.1 (-0.15--0.05)	5972.4 (3783.2-9041.3)	2.3 (1.5-3.5)	14530.9 (9078.3-21959)	2.3 (1.4-3.4)	-0.1 (-0.15--0.05)
	Female	2158.7 (1366.1-3259.7)	0.9 (0.6-1.4)	6324 (3991.4-9453.1)	0.9 (0.6-1.4)	-0.01 (-0.07-0.05)	2158.7 (1366.1-3259.7)	0.9 (0.6-1.4)	6324 (3991.4-9453.1)	0.9 (0.6-1.4)	-0.01 (-0.07-0.05)
Southeast Asia	Both	4361.2 (2758.3-6487.9)	1.9 (1.2-2.9)	13924.2 (8713.6-20861.3)	2.3 (1.4-3.4)	0.7 (0.67-0.74)	4361.2 (2758.3-6487.9)	1.9 (1.2-2.9)	13924.2 (8713.6-20861.3)	2.3 (1.4-3.4)	0.7 (0.67-0.74)
	Male	3208.8 (2030.1-4776.5)	3.1 (1.9-4.6)	10245 (6363.4-15301.8)	3.8 (2.4-5.6)	0.8 (0.75-0.85)	3208.8 (2030.1-4776.5)	3.1 (1.9-4.6)	10245 (6363.4-15301.8)	3.8 (2.4-5.6)	0.8 (0.75-0.85)
	Female	1152.4 (729.8-1709.2)	1 (0.6-1.4)	3679.1 (2307.6-5521.4)	1.1 (0.7-1.7)	0.56 (0.54-0.59)	1152.4 (729.8-1709.2)	1 (0.6-1.4)	3679.1 (2307.6-5521.4)	1.1 (0.7-1.7)	0.56 (0.54-0.59)
Southern Latin America	Both	968.5 (592.5-1471.2)	2.2 (1.3-3.3)	2257.1 (1395.7-3365.8)	2.5 (1.6-3.7)	0.48 (0.46-0.51)	968.5 (592.5-1471.2)	2.2 (1.3-3.3)	2257.1 (1395.7-3365.8)	2.5 (1.6-3.7)	0.48 (0.46-0.51)
	Male	656.8 (403-1027.3)	3.4 (2.1-5.2)	1507.5 (934.2-2327.9)	4 (2.5-6.1)	0.47 (0.43-0.51)	656.8 (403-1027.3)	3.4 (2.1-5.2)	1507.5 (934.2-2327.9)	4 (2.5-6.1)	0.47 (0.43-0.51)
	Female	311.8 (188.1-478.4)	1.2 (0.7-1.9)	749.6 (457.3-1120.2)	1.4 (0.8-2)	0.46 (0.44-0.49)	311.8 (188.1-478.4)	1.2 (0.7-1.9)	749.6 (457.3-1120.2)	1.4 (0.8-2)	0.46 (0.44-0.49)
Southern Sub-Saharan Africa	Both	644.3 (411.5-964.7)	2.6 (1.6-3.9)	1498.8 (949.3-2255.5)	2.8 (1.8-4.2)	0.3 (0.26-0.34)	644.3 (411.5-964.7)	2.6 (1.6-3.9)	1498.8 (949.3-2255.5)	2.8 (1.8-4.2)	0.3 (0.26-0.34)
	Male	453.7 (286.9-675.5)	4.4 (2.8-6.5)	1027.4 (648.6-1545.7)	4.9 (3-7.3)	0.34 (0.32-0.37)	453.7 (286.9-675.5)	4.4 (2.8-6.5)	1027.4 (648.6-1545.7)	4.9 (3-7.3)	0.34 (0.32-0.37)
	Female	190.5 (120.2-286.8)	1.3 (0.8-2)	471.4 (294.7-701)	1.5 (0.9-2.2)	0.41 (0.39-0.42)	190.5 (120.2-286.8)	1.3 (0.8-2)	471.4 (294.7-701)	1.5 (0.9-2.2)	0.41 (0.39-0.42)
Tropical Latin America	Both	735.5 (460.8-1108.5)	0.9 (0.6-1.3)	2747.3 (1757.1-4120.3)	1.1 (0.7-1.6)	0.66 (0.61-0.7)	735.5 (460.8-1108.5)	0.9 (0.6-1.3)	2747.3 (1757.1-4120.3)	1.1 (0.7-1.6)	0.66 (0.61-0.7)
	Male	511.5 (322.8-767.6)	1.3 (0.9-2)	1876.8 (1192-2776.5)	1.7 (1.1-2.5)	0.72 (0.66-0.78)	511.5 (322.8-767.6)	1.3 (0.9-2)	1876.8 (1192-2776.5)	1.7 (1.1-2.5)	0.72 (0.66-0.78)
	Female	224 (141.3-336.5)	0.5 (0.3-0.8)	870.5 (553.5-1328.1)	0.6 (0.4-0.9)	0.66 (0.63-0.69)	224 (141.3-336.5)	0.5 (0.3-0.8)	870.5 (553.5-1328.1)	0.6 (0.4-0.9)	0.66 (0.63-0.69)
Western Europe	Both	12306 (7576-18500.2)	2 (1.2-3)	22717.5 (14015.5-34342.2)	2.2 (1.3-3.3)	0.4 (0.36-0.45)	12306 (7576-18500.2)	2 (1.2-3)	22717.5 (14015.5-34342.2)	2.2 (1.3-3.3)	0.4 (0.36-0.45)
	Male	7736.2 (4773-11676.3)	3.3 (2-4.9)	15142.5 (9290.9-23257)	3.4 (2.1-5.2)	0.26 (0.2-0.32)	7736.2 (4773-11676.3)	3.3 (2-4.9)	15142.5 (9290.9-23257)	3.4 (2.1-5.2)	0.26 (0.2-0.32)
	Female	4569.7 (2836.9-6839.5)	1.2 (0.7-1.7)	7575.1 (4724-11229.5)	1.2 (0.7-1.8)	0.16 (0.15-0.18)	4569.7 (2836.9-6839.5)	1.2 (0.7-1.7)	7575.1 (4724-11229.5)	1.2 (0.7-1.8)	0.16 (0.15-0.18)
Western Sub-Saharan Africa	Both	1701.5 (1082.8-2556.6)	2.1 (1.3-3.1)	3609.5 (2283.6-5437.3)	2 (1.3-3.1)	-0.1 (-0.16--0.03)	1701.5 (1082.8-2556.6)	2.1 (1.3-3.1)	3609.5 (2283.6-5437.3)	2 (1.3-3.1)	-0.1 (-0.16--0.03)
	Male	1285.1 (812.4-1933.8)	3.3 (2-4.8)	2636.8 (1657.8-3979.3)	3.2 (2-4.8)	-0.05 (-0.1-0)	1285.1 (812.4-1933.8)	3.3 (2-4.8)	2636.8 (1657.8-3979.3)	3.2 (2-4.8)	-0.05 (-0.1-0)
	Female	416.4 (263.7-621.2)	1.1 (0.7-1.6)	972.7 (617.2-1465.7)	1 (0.7-1.6)	-0.06 (-0.11--0.02)	416.4 (263.7-621.2)	1.1 (0.7-1.6)	972.7 (617.2-1465.7)	1 (0.7-1.6)	-0.06 (-0.11--0.02)

### Trends in gout burden attributable to kidney dysfunction across nations from 1990 to 2021

**Figure 2:**
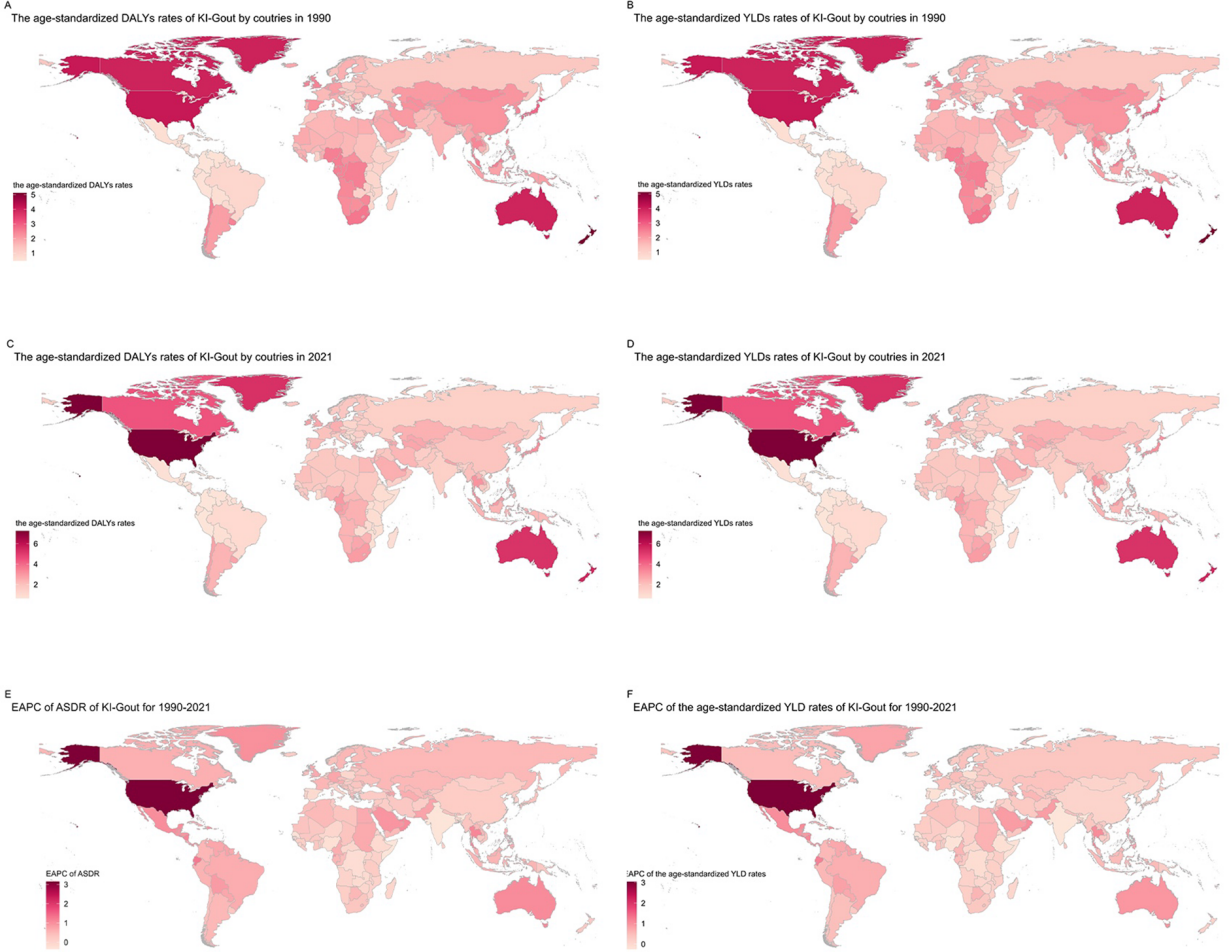
Trends in gout burden attributable to kidney dysfunction across nations from 1990 to 2021: **(A)** Age-standardized DALY rates; **(B)** Age-standardized YLD rates;**(C)** Age-standardized DALY rates; **(D)** Age-standardized YLD rates; **(E)** EAPC of ASDR; **(F)** EAPC of the age-standardized YLD rates.

From 1990 to 2021, the burden of gout attributable to kidney dysfunction exhibited varying trends across nations. In 1990, China, the United States of America, and India had the highest DALYs and YLDs due to gout from kidney dysfunction. New Zealand, the United States of America, and Greenland had the highest age-standardized DALY and YLD rates, while El Salvador, Panama, and Colombia had the lowest rates (**Figures 2A**, **B**, **Appendix Tables pp 3-71** in **Supplementary Material**). By 2021, the United States of America, China, and India remained the top three countries with the highest DALYs and YLDs. The age-standardized DALY and YLD rates were highest in the United States of America, New Zealand, and Greenland, and lowest in Colombia, Peru, and El Salvador (**Figures 2C**, **D**, **Appendix Tables pp 71-164** in **Supplementary Material**) (**Table 2**). During 1990–2021, 182 of 204 countries and territories experienced a statistically significant increase in age-standardized rates. The United States recorded the largest statistically significant increase (EAPC 3.15%, 95% CI 2.70–3.61), while India showed the largest statistically significant decrease (EAPC −0.36%, 95% CI −0.43 to −0.29). Ecuador (1.25%, 95% CI 1.14–1.36), El Salvador (1.15%, 95% CI 1.11–1.20), Somalia (−0.35%, 95% CI −0.39 to −0.31), and Senegal (−0.28%, 95% CI −0.35 to −0.21) all demonstrated statistically significant changes, as their 95% CIs excluded 0 (**Figures 2E**, **F**, **Appendix Tables pp 164-183** in **Supplementary Material**). National disparities mirror variations in CKD treatment access, dietary patterns, and uric-acid screening policies, alongside demographic aging (**Table 2**).

**Table 2:** Summary table for top and bottom 10 countries by ASDR in 1990 and 2021.

Top 10 countries by ASDR in 1990	
Location	ADSR_1990
New Zealand	5.1 (3.2-7.6)
United States of America	4.1 (2.6-6.1)
Greenland	4 (2.5-5.9)
Canada	4 (2.4-6.1)
Australia	3.9 (2.5-5.9)
Northern Mariana Islands	3.5 (2.1-5.2)
Taiwan (Province of China)	3.4 (2.2-4.9)
Brunei Darussalam	3.2 (2-4.8)
American Samoa	3.1 (1.9-4.7)
Nauru	3.1 (1.9-4.7)

Bottom 10 countries by ASDR in 1990	
Location	ADSR_1990
El Salvador	0.5 (0.3-0.7)
Colombia	0.5 (0.3-0.8)
Guatemala	0.5 (0.3-0.8)
Honduras	0.5 (0.3-0.8)
Panama	0.5 (0.3-0.8)
Peru	0.6 (0.3-0.8)
Antigua and Barbuda	0.6 (0.4-0.9)
Cuba	0.6 (0.4-0.9)
Dominican Republic	0.6 (0.4-0.9)
Haiti	0.6 (0.4-0.9)

Top 10 countries by ASDR in 2021	
Location	ASDR_2021
United States of America	7.3 (4.8-10.6)
New Zealand	5.4 (3.4-8.2)
Greenland	5.3 (3.3-7.9)
Australia	5.2 (3.2-8)
Canada	4.4 (2.7-6.6)
Northern Mariana Islands	3.6 (2.3-5.4)
Taiwan (Province of China)	3.5 (2.3-5.2)
Brunei Darussalam	3.5 (2.2-5.4)
American Samoa	3.5 (2.2-5.2)
Palau	3.4 (2.1-5)

Bottom 10 countries by ASDR in 2021	
Location	ASDR_2021
Peru	0.6 (0.4-1)
Colombia	0.6 (0.4-1)
Haiti	0.7 (0.4-1)
Panama	0.7 (0.4-1)
El Salvador	0.7 (0.4-1)
Venezuela (Bolivarian Republic of)	0.7 (0.4-1.1)
Guatemala	0.7 (0.4-1.1)
Honduras	0.7 (0.4-1.1)
Bermuda	0.7 (0.5-1.1)
Cuba	0.7 (0.5-1.1)

**Table 2** provides a summary of the top 10 and bottom 10 countries by age-standardized DALY rate (ASDR) in 1990 and 2021, facilitating comparative interpretation of the global burden of gout attributable to kidney dysfunction.

### Trends in gout burden attributable to kidney dysfunction across different age groups from 1990 to 2021

**Figure 3:**
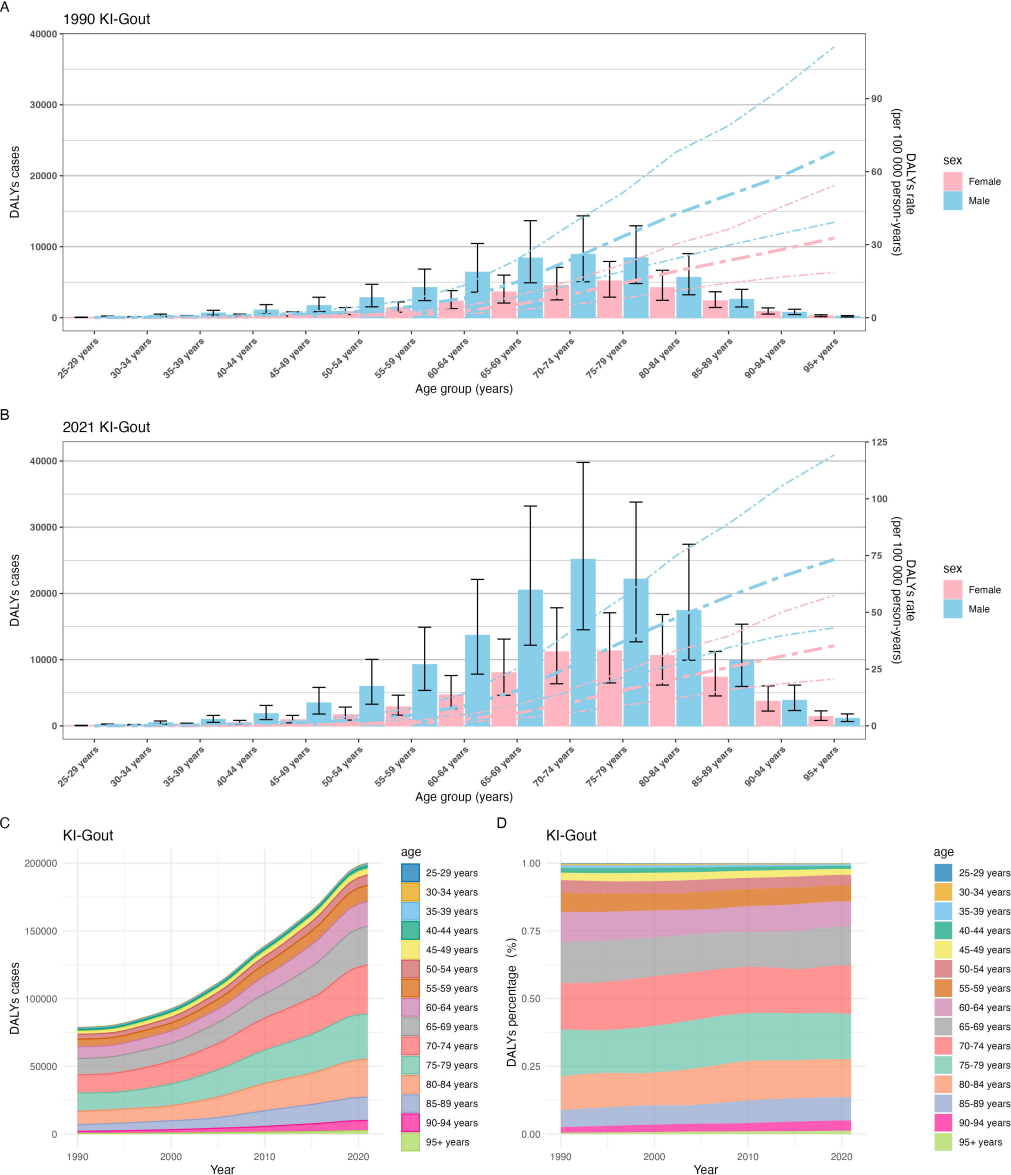
Trends in gout burden attributable to kidney dysfunction across different age groups from 1990 to 2021:**(A)** Global DALYs rate; **(B)** Global DALYs cases; **(C)** The trend in DALYs (per 100,000 population); **(D)** The change in the proportion of DALYs.

Age-specific analyses revealed progressively higher burden with advancing age, accentuated by population aging. From 1990 to 2021, the burden of gout attributable to kidney dysfunction increased with age, with the highest DALY rates observed in the oldest age groups. In 1990, the DALY rate was highest among those aged ≥95 years (41.73 [95% UI 23.85–68.99]); males had a statistically significantly higher rate (68.07 [39.18–111.28]) than females (32.69 [18.61–54.32]), as indicated by non-overlapping 95% UIs. The DALY cases were also highest in males, particularly in the 60+ age group, with the 75–79 age group having the highest number of DALY cases (13,566.82 [7691.88-20887.70]) (**Figure 3A**, **Appendix Tables pp 184-195** in **Supplementary Material**). By 2021, the DALY rate in the 95+ age group rose to 45.77 (26.91–74.94), again significantly higher in males (73.24 [43.17–113.17]) than females (35.23 [20.64–57.47]). The DALY rate in the 25–54 age group decreased compared to 1990, with the lowest rate observed in the 25–29 age group (0.04 [0.02-0.06]). However, the total number of DALY cases increased across all age groups, with the highest number of cases in the 70–74 age group (36,283.37 [20,805.80-57,020.28]), driven by a higher burden in males (22,143.90 [12,689.17-33,795.28]) compared to females (11,294.24 [6,484.24-17,079.50]) (With non-overlapping 95% UIs. Non-overlapping 95% UIs indicate that this increase is statistically significant.) (**Figure 3B**, **Appendix Tables pp 195-203** in **Supplementary Material**). Overall, the global number of DALY cases due to gout from kidney dysfunction increased significantly from 1990 to 2021. The proportion of DALYs in the 60+ age group rose from approximately 82% in 1990 to 86% in 2021, with the highest burden observed in the 65–84 age group (**Figures 3C**, **D**, **Appendix Tables pp 204-291** in **Supplementary Material**). The increase in DALYs among older age groups may partly reflect population aging, in addition to the increasing prevalence of chronic kidney disease (CKD) and hyperuricemia.

### The association between age-stratified trends in gout burden attributable to kidney dysfunction from 1990 to 2021 and the socio-demographic index

**Figure 4:**
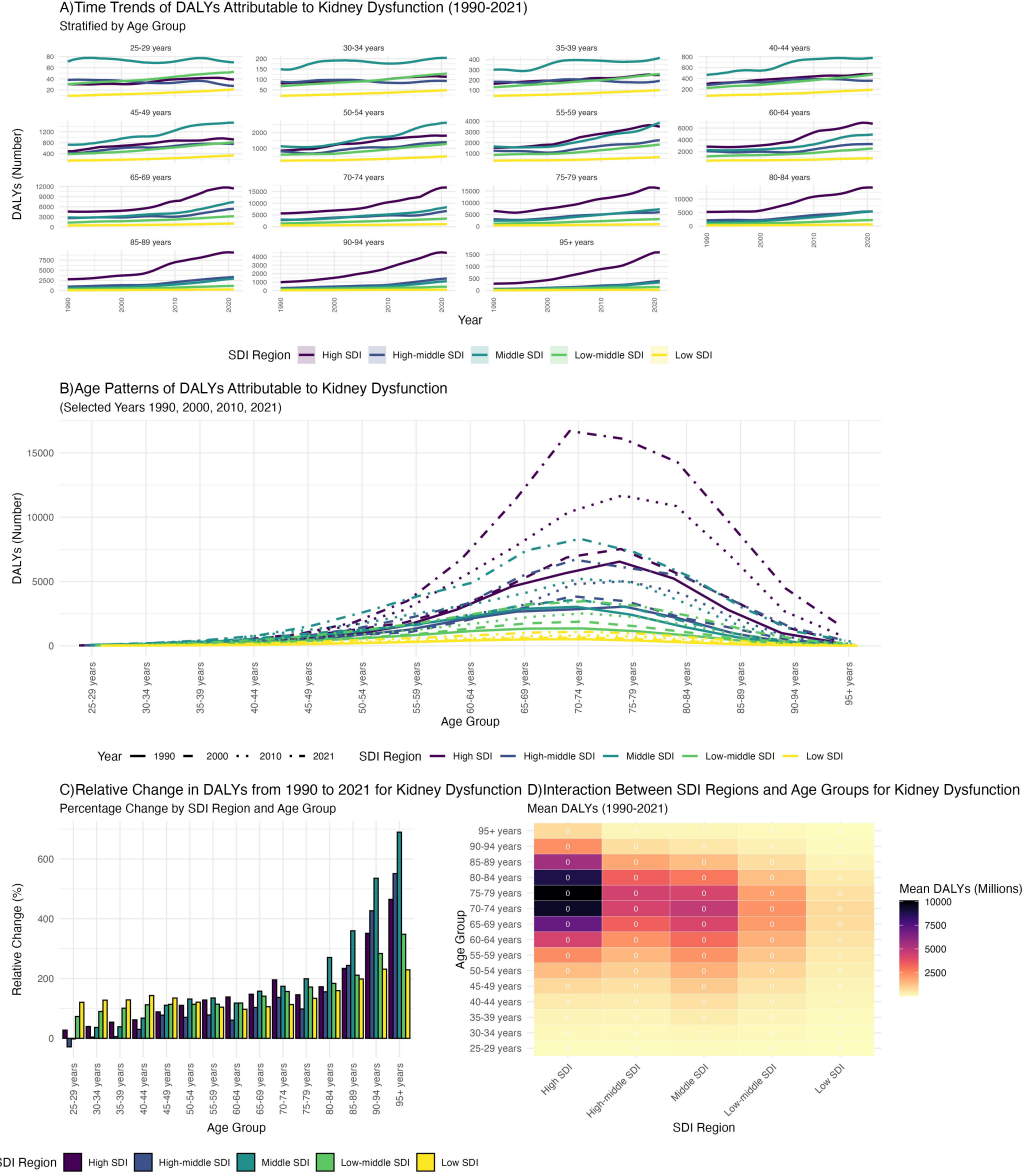
The association between age - stratified trends in gout burden attributable to kidney dysfunction from 1990 to 2021 and the Socio - demographic Index (SDI): **(A)** Time trends of DALYs; **(B)** Age patterns of DALYs; **(C)** Relative change in DALYs; **(D)** Mean DALYs.

Between 1990 and 2021, gout DALYs increased in most age groups across all five SDI regions, particularly among individuals aged 60 years and older. The increase was most pronounced in high SDI regions, whereas low SDI regions experienced a slower rise (**Figure 4A**). In 2021, the highest gout DALYs were observed in the 70–74 age group in high SDI regions, while in 1990, the highest burden was in the 75–79 age group in high SDI regions. In contrast, gout DALYs remained relatively low across all age groups in low SDI regions (**Figure 4B**). The relative change in DALYs due to gout from kidney dysfunction was most notable in the 85+ age group. The highest relative increase was seen in the 95+ age group in middle SDI regions, where DALYs in 2021 were eight times higher than in 1990, (The 8-fold relative increase in DALYs among individuals aged ≥95 years in middle-SDI regions was statistically significant, as 95% UIs for 1990 and 2021 did not overlap.) (**Figure 4C**). Overall, the mean DALYs increased with higher SDI levels, with the highest mean DALYs observed in the 75–79 age group in high SDI regions (**Figure 4D**, **Appendix Tables pp 291-463** in **Supplementary Material**). Longer life expectancy and improved diagnostics in high-SDI settings likely amplify observed burden, while under-diagnosis and resource constraints may mask true burden in low-SDI regions. DALYs increased most sharply in high-SDI regions, consistent with longer life expectancy, robust uric-acid screening programs, and broader access to CKD care. In middle-SDI regions, DALYs among ≥95 years rose 8-fold (non-overlapping 95% UIs), whereas low-SDI regions exhibited modest increases—likely attributable to under-diagnosis and limited treatment access.

## Discussion

Gout, an inflammatory arthritis caused by hyperuricemia (excess urate production or impaired excretion), is characterized by recurrent joint pain and swelling, potentially requiring hospitalization (21, 22), and significantly impacting quality of life (23). Uric acid, a purine metabolite primarily excreted by the kidneys (70%) (15), increases gout risk when renal function declines (24), as observed in chronic kidney disease (25). Kidney dysfunction is a major gout risk factor (26), but is often not actively managed in gout patients. Therefore, assessing the global, regional, and national burden of gout burden due to kidney dysfunction is essential for clinical practice and public health.

### Analysis of global trends

From 1990 to 2021, global DALYs and YLDs due to gout burden due to kidney dysfunction increased significantly. Over the past few decades, the prevalence of hyperuricemia and gout has increased globally (27). Uric acid (UA) is the end product of purine metabolism and is mainly excreted by the kidneys (28). Hyperuricemia can induce renal dysfunction by damaging renal cells and mediating inflammatory responses (29). It is also associated with an increased risk of kidney disease (30), and uric acid accumulation in chronic kidney disease can lead to gout (18). Renal function declines with age (31). The global aging population may have contributed to the significant increase in the prevalence of chronic kidney disease (CKD) (31, 32). Changes in modern lifestyle and dietary habits, such as high-sugar and high-fat diets, have been shown to cause glomerular injury (33) and hyperuricemia, which in turn can trigger gout (34, 35). Hyperuricemia has a strong genetic component (40%–70%): related genes include SNPs in SLC22A12, ABCG2, HNF1A, and HNF4A (36). Some genetic loci are shared between hyperuricemia and chronic kidney disease, indicating a possible common genetic basis (37), suggesting that gout due to kidney dysfunction also involves genetic and hereditary factors. Therefore, improving lifestyle, strengthening health education, optimizing the public health system, and conducting early screening and intervention for genetic factors can comprehensively address the increasing global burden of gout due to kidney dysfunction.

### Analysis of gender disparities

From 1990 to 2021, the burden of gout attributable to kidney dysfunction was universally higher in males than in females globally. This may be related to the protective effect of estrogen in females (estrogen can increase renal urate clearance and inhibit renal reabsorption) (38), the adverse effects of testosterone on renal damage in males (39), uric acid-related genes with sex-specific expression(such as SLC2A9 and ABCG2) (36),and higher intake of high-purine diet (40, 41), smoking (42), alcohol consumption (43), and sugary drink intake in males (44, 45). Enhanced monitoring of male populations and exploration of estrogen’s protective mechanisms are warranted for targeted prevention.

### Analysis of the drivers behind regional trends

High SDI countries saw the most significant increase in gout burden attributable to kidney dysfunction, driven by lifestyle factors (e.g., high-purine diet, alcohol intake) and advanced medical diagnostics (7, 46). Additionally, the higher DALYs in high-SDI regions may reflect better diagnostic capacity and more complete data reporting, which can inflate the measured burden relative to low-SDI countries where underdiagnosis and data limitations may lead to underestimation (20). Longer life expectancy in high-SDI countries also allows more time for individuals to live with chronic conditions such as gout and kidney dysfunction, further contributing to the observed increase in DALYs (47, 48). In contrast, Low SDI and Low-middle SDI countries experienced a decline, reflecting limited public health systems and medical resources (20). The declines in South Asia and East Africa may be attributed to lower intake of high-purine foods and younger population structures (5, 49, 50). High SDI regions should focus on optimizing medical resources and health education, while low SDI regions need increased public health investment to address resource shortages and improve chronic disease management. North America and Australasia showed rapid increases in DALYs, consistent with their high SDI levels (46). These regions should focus on optimizing medical resources and health education to address the increasing burden of gout attributable to kidney dysfunction. Meanwhile, low-SDI regions need increased public health investment to address resource shortages and improve chronic disease management, thereby reducing the global disparities in the burden of gout.

### Analysis of the reasons behind national trends

In 2021, the United States, China, and India had the highest total DALYs due to gout burden due to kidney dysfunction globally, with the United States experiencing the largest increase. Approximately 5% of adults in the United States are affected by gout, about 20% of adults have hyperuricemia, and the number of gout-related visits to the emergency department is increasing (51). The Western dietary habits in the United States (such as high intake of purine-rich foods and alcohol) are an important factor in the high incidence of gout (52). In China, a diet high in purines and a sedentary lifestyle have led to an increase in the incidence of gout (53). Although the burden of gout in India is relatively low (38), chronic kidney disease is one of the major health challenges in India (54), which indirectly leads to an increase in the burden of gout attributable to kidney dysfunction. The increase in burden in India is relatively low, with an EAPC of -0.36 for DALYs and YLDs, which may be related to limited public health resources and incomplete data collection. Public health intervention policies should be formulated for different countries to specifically reduce the burden of gout burden due to kidney dysfunction.

### Analysis of the reasons for trends in age-stratified data

Data from 1990 to 2021 show a significant increase in gout burden attributable to kidney dysfunction with age, particularly in individuals aged 60 and above. In 2021, the global DALY rate for those aged 95+ reached 45.77, with males having higher DALY cases than females. This trend reflects global aging (31), longer life expectancy, and rising CKD prevalence among the elderly (55, 56). The GBD (Global Burden of Disease) 2021 Gout Collaborators study highlights that worsening kidney dysfunction with age drives the increased gout burden (4). Future public health policies should prioritize management and early intervention of gout in the elderly.

### Analysis of the reasons for the correlation between age-stratified trends and trends in the socio-demographic index

From 1990 to 2021, the burden of gout attributable to kidney dysfunction increased significantly in most age groups across SDI regions, particularly in individuals aged 60 and above in high SDI areas. The relative percentage change in DALYs and overall average DALYs rose with increasing age and SDI, driven by declining kidney function in the elderly (47, 48).The higher burden in high-SDI regions may also be attributed to better diagnostic capacity and more complete data reporting, which can lead to more accurate identification and management of gout and its underlying risk factors (20). Additionally, longer life expectancy in high-SDI regions allows more time for individuals to develop and live with chronic conditions such as gout and kidney dysfunction (47, 48). Singh, J.A. et al. reported that in high SDI regions, the elderly are more prone to uric acid excretion disorders due to declining kidney function, which increases the incidence of gout (57). High SDI regions should focus on personalized health management for the elderly, emphasizing early detection and intervention for gout and kidney dysfunction. In contrast, low-SDI regions need to improve basic medical services and health management to better identify and manage these conditions. Strengthening health infrastructure and improving surveillance systems in low-SDI regions are essential steps to reduce the global disparities in the burden of gout attributable to kidney dysfunction.

## Limitations and future work

This study leverages data from the GBD database, which has several limitations. Modeling assumptions and ecological bias may influence the estimates. Underdiagnosis of gout and kidney dysfunction, particularly in low- and middle-income countries, could lead to underestimation of the true burden. Additionally, variations in coding practices across countries may affect the comparability of data. Future research should explicitly target the elderly, males, and populations in high SDI regions, and further conduct prospective cohort studies to clarify the temporal association between impaired kidney function and gout.

## Conclusion

This study comprehensively assessed the impact of impaired kidney function on the global burden of gout from 1990 to 2021, confirming that impaired kidney function is a significant risk factor for gout. Our findings highlight the substantial increase in the burden of gout attributable to kidney dysfunction, particularly among the elderly, males, and populations in high SDI regions. The observed trends are driven by multiple factors, including lifestyle and dietary habits, diagnostic capacity, and demographic shifts. Future efforts should focus on improving surveillance and access to care in low-SDI regions to address potential underestimation of the burden. High-SDI regions should prioritize personalized health management strategies for the elderly, emphasizing early detection and intervention for gout and kidney dysfunction. Additionally, further prospective studies and genetic research are needed to elucidate the underlying mechanisms and identify novel therapeutic targets. Through enhanced public health initiatives, improved healthcare systems, and international collaboration, we can address the global health challenge posed by gout attributable to kidney dysfunction and work towards reducing its burden in affected populations.

## Data Availability

The datasets presented in this study can be found in online repositories. The names of the repository/repositories and accession number(s) can be found in the article/**Supplementary Material**.
