# Supplementary material for: Trends in the global burden of gout attributable to kidney dysfunction, 1990-2021: a population-based analysis
Source: Front Endocrinol (Lausanne). 2025 Oct 21;16:1677340. doi: 10.3389/fendo.2025.1677340 (PMC12583099; doi:10.3389/fendo.2025.1677340)
Supplement: Supplementary file 1 [file DataSheet1.docx]

**Appendix 1: Global gout DALYs and YLDs attributed to renal dysfunction, 1990–2021**

| measure | location | sex | age | cause | rei | metric | year | val | upper | lower |
| --- | --- | --- | --- | --- | --- | --- | --- | --- | --- | --- |
| DALYs (Disability-Adjusted Life Years) | Global | Male | All ages | Gout | Kidney dysfunction | Number | 1990 | 52051.58308 | 78861.11353 | 32525.28975 |
| DALYs (Disability-Adjusted Life Years) | Global | Female | All ages | Gout | Kidney dysfunction | Number | 1990 | 26513.2507 | 39608.62678 | 16593.32812 |
| DALYs (Disability-Adjusted Life Years) | Global | Both | All ages | Gout | Kidney dysfunction | Number | 1990 | 78564.83378 | 118061.9027 | 48687.91227 |
| DALYs (Disability-Adjusted Life Years) | Global | Male | All ages | Gout | Kidney dysfunction | Number | 2021 | 135608.0635 | 202098.0839 | 84507.29422 |
| DALYs (Disability-Adjusted Life Years) | Global | Female | All ages | Gout | Kidney dysfunction | Number | 2021 | 64425.27429 | 94545.35799 | 40432.08576 |
| DALYs (Disability-Adjusted Life Years) | Global | Both | All ages | Gout | Kidney dysfunction | Number | 2021 | 200033.3378 | 296812.4115 | 125245.0319 |
| YLDs (Years Lived with Disability) | Global | Male | All ages | Gout | Kidney dysfunction | Number | 1990 | 52051.58308 | 78861.11353 | 32525.28975 |
| YLDs (Years Lived with Disability) | Global | Female | All ages | Gout | Kidney dysfunction | Number | 1990 | 26513.2507 | 39608.62678 | 16593.32812 |
| YLDs (Years Lived with Disability) | Global | Both | All ages | Gout | Kidney dysfunction | Number | 1990 | 78564.83378 | 118061.9027 | 48687.91227 |
| YLDs (Years Lived with Disability) | Global | Male | All ages | Gout | Kidney dysfunction | Number | 2021 | 135608.0635 | 202098.0839 | 84507.29422 |
| YLDs (Years Lived with Disability) | Global | Female | All ages | Gout | Kidney dysfunction | Number | 2021 | 64425.27429 | 94545.35799 | 40432.08576 |
| YLDs (Years Lived with Disability) | Global | Both | All ages | Gout | Kidney dysfunction | Number | 2021 | 200033.3378 | 296812.4115 | 125245.0319 |

**Appendix 2：Global age-standardized mortality rate (ASMR) and age-standardized YLD rate of gout due to renal dysfunction, 1990–2021**

| measure | location | sex | age | cause | rei | metric | year | val | upper | lower |
| --- | --- | --- | --- | --- | --- | --- | --- | --- | --- | --- |
| DALYs (Disability-Adjusted Life Years) | Global | Male | Age-standardized | Gout | Kidney dysfunction | Rate | 1990 | 3.26158249 | 4.846341276 | 2.024969974 |
| DALYs (Disability-Adjusted Life Years) | Global | Female | Age-standardized | Gout | Kidney dysfunction | Rate | 1990 | 1.306284489 | 1.934015877 | 0.821149122 |
| DALYs (Disability-Adjusted Life Years) | Global | Both | Age-standardized | Gout | Kidney dysfunction | Rate | 1990 | 2.147380033 | 3.182367821 | 1.335582731 |
| DALYs (Disability-Adjusted Life Years) | Global | Male | Age-standardized | Gout | Kidney dysfunction | Rate | 2021 | 3.554652915 | 5.279234814 | 2.220799278 |
| DALYs (Disability-Adjusted Life Years) | Global | Female | Age-standardized | Gout | Kidney dysfunction | Rate | 2021 | 1.379864126 | 2.027211938 | 0.865767331 |
| DALYs (Disability-Adjusted Life Years) | Global | Both | Age-standardized | Gout | Kidney dysfunction | Rate | 2021 | 2.360696724 | 3.497077676 | 1.477200837 |
| YLDs (Years Lived with Disability) | Global | Male | Age-standardized | Gout | Kidney dysfunction | Rate | 1990 | 3.26158249 | 4.846341276 | 2.024969974 |
| YLDs (Years Lived with Disability) | Global | Female | Age-standardized | Gout | Kidney dysfunction | Rate | 1990 | 1.306284489 | 1.934015877 | 0.821149122 |
| YLDs (Years Lived with Disability) | Global | Both | Age-standardized | Gout | Kidney dysfunction | Rate | 1990 | 2.147380033 | 3.182367821 | 1.335582731 |
| YLDs (Years Lived with Disability) | Global | Male | Age-standardized | Gout | Kidney dysfunction | Rate | 2021 | 3.554652915 | 5.279234814 | 2.220799278 |
| YLDs (Years Lived with Disability) | Global | Female | Age-standardized | Gout | Kidney dysfunction | Rate | 2021 | 1.379864126 | 2.027211938 | 0.865767331 |
| YLDs (Years Lived with Disability) | Global | Both | Age-standardized | Gout | Kidney dysfunction | Rate | 2021 | 2.360696724 | 3.497077676 | 1.477200837 |

**Appendix 3：In 1990, the DALYs and YLDs associated with gout due to renal dysfunction across 204 countries**

| measure | location | sex | age | cause | rei | metric | year | val | upper | lower |
| --- | --- | --- | --- | --- | --- | --- | --- | --- | --- | --- |
| DALYs (Disability-Adjusted Life Years) | China | Both | All ages | Gout | Kidney dysfunction | Number | 1990 | 16358.59038 | 24685.9197 | 10116.9779 |
| YLDs (Years Lived with Disability) | China | Both | All ages | Gout | Kidney dysfunction | Number | 1990 | 16358.59038 | 24685.9197 | 10116.9779 |
| DALYs (Disability-Adjusted Life Years) | United States of America | Both | All ages | Gout | Kidney dysfunction | Number | 1990 | 13807.55515 | 20518.9757 | 8538.595 |
| YLDs (Years Lived with Disability) | United States of America | Both | All ages | Gout | Kidney dysfunction | Number | 1990 | 13807.55515 | 20518.9757 | 8538.595 |
| DALYs (Disability-Adjusted Life Years) | India | Both | All ages | Gout | Kidney dysfunction | Number | 1990 | 6535.128062 | 9835.43375 | 4144.76143 |
| YLDs (Years Lived with Disability) | India | Both | All ages | Gout | Kidney dysfunction | Number | 1990 | 6535.128062 | 9835.43375 | 4144.76143 |
| DALYs (Disability-Adjusted Life Years) | Japan | Both | All ages | Gout | Kidney dysfunction | Number | 1990 | 4826.637673 | 7240.04485 | 2978.45302 |
| YLDs (Years Lived with Disability) | Japan | Both | All ages | Gout | Kidney dysfunction | Number | 1990 | 4826.637673 | 7240.04485 | 2978.45302 |
| DALYs (Disability-Adjusted Life Years) | Germany | Both | All ages | Gout | Kidney dysfunction | Number | 1990 | 2732.091607 | 4191.38222 | 1652.03393 |
| YLDs (Years Lived with Disability) | Germany | Both | All ages | Gout | Kidney dysfunction | Number | 1990 | 2732.091607 | 4191.38222 | 1652.03393 |
| DALYs (Disability-Adjusted Life Years) | United Kingdom | Both | All ages | Gout | Kidney dysfunction | Number | 1990 | 2402.350897 | 3605.7361 | 1473.71082 |
| YLDs (Years Lived with Disability) | United Kingdom | Both | All ages | Gout | Kidney dysfunction | Number | 1990 | 2402.350897 | 3605.7361 | 1473.71082 |
| DALYs (Disability-Adjusted Life Years) | Russian Federation | Both | All ages | Gout | Kidney dysfunction | Number | 1990 | 2264.677875 | 3409.87029 | 1408.99977 |
| YLDs (Years Lived with Disability) | Russian Federation | Both | All ages | Gout | Kidney dysfunction | Number | 1990 | 2264.677875 | 3409.87029 | 1408.99977 |
| DALYs (Disability-Adjusted Life Years) | Indonesia | Both | All ages | Gout | Kidney dysfunction | Number | 1990 | 1660.363066 | 2490.00884 | 1059.25597 |
| YLDs (Years Lived with Disability) | Indonesia | Both | All ages | Gout | Kidney dysfunction | Number | 1990 | 1660.363066 | 2490.00884 | 1059.25597 |
| DALYs (Disability-Adjusted Life Years) | Italy | Both | All ages | Gout | Kidney dysfunction | Number | 1990 | 1655.256568 | 2483.14829 | 1011.2338 |
| YLDs (Years Lived with Disability) | Italy | Both | All ages | Gout | Kidney dysfunction | Number | 1990 | 1655.256568 | 2483.14829 | 1011.2338 |
| DALYs (Disability-Adjusted Life Years) | France | Both | All ages | Gout | Kidney dysfunction | Number | 1990 | 1426.466294 | 2127.18163 | 873.255525 |
| YLDs (Years Lived with Disability) | France | Both | All ages | Gout | Kidney dysfunction | Number | 1990 | 1426.466294 | 2127.18163 | 873.255525 |
| DALYs (Disability-Adjusted Life Years) | Canada | Both | All ages | Gout | Kidney dysfunction | Number | 1990 | 1320.040681 | 2016.61248 | 806.477448 |
| YLDs (Years Lived with Disability) | Canada | Both | All ages | Gout | Kidney dysfunction | Number | 1990 | 1320.040681 | 2016.61248 | 806.477448 |
| DALYs (Disability-Adjusted Life Years) | Spain | Both | All ages | Gout | Kidney dysfunction | Number | 1990 | 1263.392021 | 1910.83653 | 778.17503 |
| YLDs (Years Lived with Disability) | Spain | Both | All ages | Gout | Kidney dysfunction | Number | 1990 | 1263.392021 | 1910.83653 | 778.17503 |
| DALYs (Disability-Adjusted Life Years) | Ukraine | Both | All ages | Gout | Kidney dysfunction | Number | 1990 | 1092.925139 | 1627.05616 | 679.10499 |
| YLDs (Years Lived with Disability) | Ukraine | Both | All ages | Gout | Kidney dysfunction | Number | 1990 | 1092.925139 | 1627.05616 | 679.10499 |
| DALYs (Disability-Adjusted Life Years) | Nigeria | Both | All ages | Gout | Kidney dysfunction | Number | 1990 | 1043.108887 | 1576.7781 | 667.497663 |
| YLDs (Years Lived with Disability) | Nigeria | Both | All ages | Gout | Kidney dysfunction | Number | 1990 | 1043.108887 | 1576.7781 | 667.497663 |
| DALYs (Disability-Adjusted Life Years) | Australia | Both | All ages | Gout | Kidney dysfunction | Number | 1990 | 794.5748883 | 1193.69312 | 497.658086 |
| YLDs (Years Lived with Disability) | Australia | Both | All ages | Gout | Kidney dysfunction | Number | 1990 | 794.5748883 | 1193.69312 | 497.658086 |
| DALYs (Disability-Adjusted Life Years) | Thailand | Both | All ages | Gout | Kidney dysfunction | Number | 1990 | 776.9526059 | 1157.40883 | 499.431618 |
| YLDs (Years Lived with Disability) | Thailand | Both | All ages | Gout | Kidney dysfunction | Number | 1990 | 776.9526059 | 1157.40883 | 499.431618 |
| DALYs (Disability-Adjusted Life Years) | Pakistan | Both | All ages | Gout | Kidney dysfunction | Number | 1990 | 762.2108374 | 1134.47931 | 477.222634 |
| YLDs (Years Lived with Disability) | Pakistan | Both | All ages | Gout | Kidney dysfunction | Number | 1990 | 762.2108374 | 1134.47931 | 477.222634 |
| DALYs (Disability-Adjusted Life Years) | Brazil | Both | All ages | Gout | Kidney dysfunction | Number | 1990 | 717.7602858 | 1083.70828 | 450.129974 |
| YLDs (Years Lived with Disability) | Brazil | Both | All ages | Gout | Kidney dysfunction | Number | 1990 | 717.7602858 | 1083.70828 | 450.129974 |
| DALYs (Disability-Adjusted Life Years) | Argentina | Both | All ages | Gout | Kidney dysfunction | Number | 1990 | 642.5399808 | 985.193624 | 390.025921 |
| YLDs (Years Lived with Disability) | Argentina | Both | All ages | Gout | Kidney dysfunction | Number | 1990 | 642.5399808 | 985.193624 | 390.025921 |
| DALYs (Disability-Adjusted Life Years) | Bangladesh | Both | All ages | Gout | Kidney dysfunction | Number | 1990 | 634.5988245 | 952.812502 | 389.32439 |
| YLDs (Years Lived with Disability) | Bangladesh | Both | All ages | Gout | Kidney dysfunction | Number | 1990 | 634.5988245 | 952.812502 | 389.32439 |
| DALYs (Disability-Adjusted Life Years) | Republic of Korea | Both | All ages | Gout | Kidney dysfunction | Number | 1990 | 608.9142552 | 923.78661 | 374.259326 |
| YLDs (Years Lived with Disability) | Republic of Korea | Both | All ages | Gout | Kidney dysfunction | Number | 1990 | 608.9142552 | 923.78661 | 374.259326 |
| DALYs (Disability-Adjusted Life Years) | Poland | Both | All ages | Gout | Kidney dysfunction | Number | 1990 | 540.4087387 | 818.476277 | 337.399874 |
| YLDs (Years Lived with Disability) | Poland | Both | All ages | Gout | Kidney dysfunction | Number | 1990 | 540.4087387 | 818.476277 | 337.399874 |
| DALYs (Disability-Adjusted Life Years) | Turkey | Both | All ages | Gout | Kidney dysfunction | Number | 1990 | 535.7533681 | 808.924791 | 326.032839 |
| YLDs (Years Lived with Disability) | Turkey | Both | All ages | Gout | Kidney dysfunction | Number | 1990 | 535.7533681 | 808.924791 | 326.032839 |
| DALYs (Disability-Adjusted Life Years) | Philippines | Both | All ages | Gout | Kidney dysfunction | Number | 1990 | 511.8987673 | 771.204641 | 319.223083 |
| YLDs (Years Lived with Disability) | Philippines | Both | All ages | Gout | Kidney dysfunction | Number | 1990 | 511.8987673 | 771.204641 | 319.223083 |
| DALYs (Disability-Adjusted Life Years) | South Africa | Both | All ages | Gout | Kidney dysfunction | Number | 1990 | 508.5724078 | 763.259854 | 324.594709 |
| YLDs (Years Lived with Disability) | South Africa | Both | All ages | Gout | Kidney dysfunction | Number | 1990 | 508.5724078 | 763.259854 | 324.594709 |
| DALYs (Disability-Adjusted Life Years) | Iran (Islamic Republic of) | Both | All ages | Gout | Kidney dysfunction | Number | 1990 | 495.3838744 | 754.927546 | 307.650918 |
| YLDs (Years Lived with Disability) | Iran (Islamic Republic of) | Both | All ages | Gout | Kidney dysfunction | Number | 1990 | 495.3838744 | 754.927546 | 307.650918 |
| DALYs (Disability-Adjusted Life Years) | Taiwan (Province of China) | Both | All ages | Gout | Kidney dysfunction | Number | 1990 | 484.8567629 | 711.964302 | 311.446409 |
| YLDs (Years Lived with Disability) | Taiwan (Province of China) | Both | All ages | Gout | Kidney dysfunction | Number | 1990 | 484.8567629 | 711.964302 | 311.446409 |
| DALYs (Disability-Adjusted Life Years) | Viet Nam | Both | All ages | Gout | Kidney dysfunction | Number | 1990 | 467.7181931 | 687.659048 | 283.927471 |
| YLDs (Years Lived with Disability) | Viet Nam | Both | All ages | Gout | Kidney dysfunction | Number | 1990 | 467.7181931 | 687.659048 | 283.927471 |
| DALYs (Disability-Adjusted Life Years) | Netherlands | Both | All ages | Gout | Kidney dysfunction | Number | 1990 | 424.3450519 | 630.030695 | 257.363112 |
| YLDs (Years Lived with Disability) | Netherlands | Both | All ages | Gout | Kidney dysfunction | Number | 1990 | 424.3450519 | 630.030695 | 257.363112 |
| DALYs (Disability-Adjusted Life Years) | Egypt | Both | All ages | Gout | Kidney dysfunction | Number | 1990 | 409.7350289 | 609.468027 | 258.713335 |
| YLDs (Years Lived with Disability) | Egypt | Both | All ages | Gout | Kidney dysfunction | Number | 1990 | 409.7350289 | 609.468027 | 258.713335 |
| DALYs (Disability-Adjusted Life Years) | Myanmar | Both | All ages | Gout | Kidney dysfunction | Number | 1990 | 388.4383501 | 587.780827 | 241.23046 |
| YLDs (Years Lived with Disability) | Myanmar | Both | All ages | Gout | Kidney dysfunction | Number | 1990 | 388.4383501 | 587.780827 | 241.23046 |
| DALYs (Disability-Adjusted Life Years) | Greece | Both | All ages | Gout | Kidney dysfunction | Number | 1990 | 378.3416595 | 587.413175 | 238.449155 |
| YLDs (Years Lived with Disability) | Greece | Both | All ages | Gout | Kidney dysfunction | Number | 1990 | 378.3416595 | 587.413175 | 238.449155 |
| DALYs (Disability-Adjusted Life Years) | Romania | Both | All ages | Gout | Kidney dysfunction | Number | 1990 | 345.8260997 | 520.245931 | 210.713447 |
| YLDs (Years Lived with Disability) | Romania | Both | All ages | Gout | Kidney dysfunction | Number | 1990 | 345.8260997 | 520.245931 | 210.713447 |
| DALYs (Disability-Adjusted Life Years) | Belgium | Both | All ages | Gout | Kidney dysfunction | Number | 1990 | 338.1098256 | 510.230278 | 213.702986 |
| YLDs (Years Lived with Disability) | Belgium | Both | All ages | Gout | Kidney dysfunction | Number | 1990 | 338.1098256 | 510.230278 | 213.702986 |
| DALYs (Disability-Adjusted Life Years) | Democratic Republic of the Congo | Both | All ages | Gout | Kidney dysfunction | Number | 1990 | 325.0093819 | 477.898591 | 203.570611 |
| YLDs (Years Lived with Disability) | Democratic Republic of the Congo | Both | All ages | Gout | Kidney dysfunction | Number | 1990 | 325.0093819 | 477.898591 | 203.570611 |
| DALYs (Disability-Adjusted Life Years) | Democratic People's Republic of Korea | Both | All ages | Gout | Kidney dysfunction | Number | 1990 | 310.1093318 | 466.125658 | 192.863048 |
| YLDs (Years Lived with Disability) | Democratic People's Republic of Korea | Both | All ages | Gout | Kidney dysfunction | Number | 1990 | 310.1093318 | 466.125658 | 192.863048 |
| DALYs (Disability-Adjusted Life Years) | Sweden | Both | All ages | Gout | Kidney dysfunction | Number | 1990 | 297.6065023 | 455.813018 | 179.415261 |
| YLDs (Years Lived with Disability) | Sweden | Both | All ages | Gout | Kidney dysfunction | Number | 1990 | 297.6065023 | 455.813018 | 179.415261 |
| DALYs (Disability-Adjusted Life Years) | Mexico | Both | All ages | Gout | Kidney dysfunction | Number | 1990 | 261.6700831 | 393.84227 | 164.926743 |
| YLDs (Years Lived with Disability) | Mexico | Both | All ages | Gout | Kidney dysfunction | Number | 1990 | 261.6700831 | 393.84227 | 164.926743 |
| DALYs (Disability-Adjusted Life Years) | Uzbekistan | Both | All ages | Gout | Kidney dysfunction | Number | 1990 | 253.3781146 | 372.142221 | 157.130729 |
| YLDs (Years Lived with Disability) | Uzbekistan | Both | All ages | Gout | Kidney dysfunction | Number | 1990 | 253.3781146 | 372.142221 | 157.130729 |
| DALYs (Disability-Adjusted Life Years) | Kazakhstan | Both | All ages | Gout | Kidney dysfunction | Number | 1990 | 250.0418405 | 378.379319 | 157.547821 |
| YLDs (Years Lived with Disability) | Kazakhstan | Both | All ages | Gout | Kidney dysfunction | Number | 1990 | 250.0418405 | 378.379319 | 157.547821 |
| DALYs (Disability-Adjusted Life Years) | Austria | Both | All ages | Gout | Kidney dysfunction | Number | 1990 | 242.1350419 | 365.073819 | 147.97262 |
| YLDs (Years Lived with Disability) | Austria | Both | All ages | Gout | Kidney dysfunction | Number | 1990 | 242.1350419 | 365.073819 | 147.97262 |
| DALYs (Disability-Adjusted Life Years) | Portugal | Both | All ages | Gout | Kidney dysfunction | Number | 1990 | 232.2673663 | 363.012851 | 140.076195 |
| YLDs (Years Lived with Disability) | Portugal | Both | All ages | Gout | Kidney dysfunction | Number | 1990 | 232.2673663 | 363.012851 | 140.076195 |
| DALYs (Disability-Adjusted Life Years) | Switzerland | Both | All ages | Gout | Kidney dysfunction | Number | 1990 | 226.9619988 | 347.773328 | 140.529358 |
| YLDs (Years Lived with Disability) | Switzerland | Both | All ages | Gout | Kidney dysfunction | Number | 1990 | 226.9619988 | 347.773328 | 140.529358 |
| DALYs (Disability-Adjusted Life Years) | Chile | Both | All ages | Gout | Kidney dysfunction | Number | 1990 | 226.3869214 | 343.102499 | 138.241948 |
| YLDs (Years Lived with Disability) | Chile | Both | All ages | Gout | Kidney dysfunction | Number | 1990 | 226.3869214 | 343.102499 | 138.241948 |
| DALYs (Disability-Adjusted Life Years) | Malaysia | Both | All ages | Gout | Kidney dysfunction | Number | 1990 | 213.3582848 | 321.108249 | 134.403951 |
| YLDs (Years Lived with Disability) | Malaysia | Both | All ages | Gout | Kidney dysfunction | Number | 1990 | 213.3582848 | 321.108249 | 134.403951 |
| DALYs (Disability-Adjusted Life Years) | New Zealand | Both | All ages | Gout | Kidney dysfunction | Number | 1990 | 207.9473683 | 310.223885 | 128.014883 |
| YLDs (Years Lived with Disability) | New Zealand | Both | All ages | Gout | Kidney dysfunction | Number | 1990 | 207.9473683 | 310.223885 | 128.014883 |
| DALYs (Disability-Adjusted Life Years) | Sri Lanka | Both | All ages | Gout | Kidney dysfunction | Number | 1990 | 206.160952 | 312.940488 | 129.642383 |
| YLDs (Years Lived with Disability) | Sri Lanka | Both | All ages | Gout | Kidney dysfunction | Number | 1990 | 206.160952 | 312.940488 | 129.642383 |
| DALYs (Disability-Adjusted Life Years) | Nepal | Both | All ages | Gout | Kidney dysfunction | Number | 1990 | 195.5833512 | 295.197453 | 124.153482 |
| YLDs (Years Lived with Disability) | Nepal | Both | All ages | Gout | Kidney dysfunction | Number | 1990 | 195.5833512 | 295.197453 | 124.153482 |
| DALYs (Disability-Adjusted Life Years) | Belarus | Both | All ages | Gout | Kidney dysfunction | Number | 1990 | 188.6637398 | 281.206153 | 117.976236 |
| YLDs (Years Lived with Disability) | Belarus | Both | All ages | Gout | Kidney dysfunction | Number | 1990 | 188.6637398 | 281.206153 | 117.976236 |
| DALYs (Disability-Adjusted Life Years) | Algeria | Both | All ages | Gout | Kidney dysfunction | Number | 1990 | 179.7019836 | 275.5095 | 109.580888 |
| YLDs (Years Lived with Disability) | Algeria | Both | All ages | Gout | Kidney dysfunction | Number | 1990 | 179.7019836 | 275.5095 | 109.580888 |
| DALYs (Disability-Adjusted Life Years) | Denmark | Both | All ages | Gout | Kidney dysfunction | Number | 1990 | 173.3828825 | 266.113023 | 105.924098 |
| YLDs (Years Lived with Disability) | Denmark | Both | All ages | Gout | Kidney dysfunction | Number | 1990 | 173.3828825 | 266.113023 | 105.924098 |
| DALYs (Disability-Adjusted Life Years) | Hungary | Both | All ages | Gout | Kidney dysfunction | Number | 1990 | 169.2596365 | 251.956117 | 102.540662 |
| YLDs (Years Lived with Disability) | Hungary | Both | All ages | Gout | Kidney dysfunction | Number | 1990 | 169.2596365 | 251.956117 | 102.540662 |
| DALYs (Disability-Adjusted Life Years) | Ethiopia | Both | All ages | Gout | Kidney dysfunction | Number | 1990 | 167.136884 | 256.700303 | 104.360601 |
| YLDs (Years Lived with Disability) | Ethiopia | Both | All ages | Gout | Kidney dysfunction | Number | 1990 | 167.136884 | 256.700303 | 104.360601 |
| DALYs (Disability-Adjusted Life Years) | Morocco | Both | All ages | Gout | Kidney dysfunction | Number | 1990 | 162.0597923 | 241.679663 | 98.7344991 |
| YLDs (Years Lived with Disability) | Morocco | Both | All ages | Gout | Kidney dysfunction | Number | 1990 | 162.0597923 | 241.679663 | 98.7344991 |
| DALYs (Disability-Adjusted Life Years) | Czechia | Both | All ages | Gout | Kidney dysfunction | Number | 1990 | 155.7481281 | 233.937764 | 94.4433216 |
| YLDs (Years Lived with Disability) | Czechia | Both | All ages | Gout | Kidney dysfunction | Number | 1990 | 155.7481281 | 233.937764 | 94.4433216 |
| DALYs (Disability-Adjusted Life Years) | Bulgaria | Both | All ages | Gout | Kidney dysfunction | Number | 1990 | 150.6623116 | 224.50979 | 90.5428574 |
| YLDs (Years Lived with Disability) | Bulgaria | Both | All ages | Gout | Kidney dysfunction | Number | 1990 | 150.6623116 | 224.50979 | 90.5428574 |
| DALYs (Disability-Adjusted Life Years) | Iraq | Both | All ages | Gout | Kidney dysfunction | Number | 1990 | 137.9376422 | 208.790419 | 83.9851261 |
| YLDs (Years Lived with Disability) | Iraq | Both | All ages | Gout | Kidney dysfunction | Number | 1990 | 137.9376422 | 208.790419 | 83.9851261 |
| DALYs (Disability-Adjusted Life Years) | Georgia | Both | All ages | Gout | Kidney dysfunction | Number | 1990 | 129.6982514 | 194.573827 | 83.6437697 |
| YLDs (Years Lived with Disability) | Georgia | Both | All ages | Gout | Kidney dysfunction | Number | 1990 | 129.6982514 | 194.573827 | 83.6437697 |
| DALYs (Disability-Adjusted Life Years) | Sudan | Both | All ages | Gout | Kidney dysfunction | Number | 1990 | 124.4019948 | 190.331437 | 76.115225 |
| YLDs (Years Lived with Disability) | Sudan | Both | All ages | Gout | Kidney dysfunction | Number | 1990 | 124.4019948 | 190.331437 | 76.115225 |
| DALYs (Disability-Adjusted Life Years) | Israel | Both | All ages | Gout | Kidney dysfunction | Number | 1990 | 120.779493 | 184.859515 | 72.4465214 |
| YLDs (Years Lived with Disability) | Israel | Both | All ages | Gout | Kidney dysfunction | Number | 1990 | 120.779493 | 184.859515 | 72.4465214 |
| DALYs (Disability-Adjusted Life Years) | Finland | Both | All ages | Gout | Kidney dysfunction | Number | 1990 | 120.4642103 | 186.431783 | 73.2673051 |
| YLDs (Years Lived with Disability) | Finland | Both | All ages | Gout | Kidney dysfunction | Number | 1990 | 120.4642103 | 186.431783 | 73.2673051 |
| DALYs (Disability-Adjusted Life Years) | Norway | Both | All ages | Gout | Kidney dysfunction | Number | 1990 | 108.5139954 | 163.241858 | 66.7760335 |
| YLDs (Years Lived with Disability) | Norway | Both | All ages | Gout | Kidney dysfunction | Number | 1990 | 108.5139954 | 163.241858 | 66.7760335 |
| DALYs (Disability-Adjusted Life Years) | Ireland | Both | All ages | Gout | Kidney dysfunction | Number | 1990 | 105.0444386 | 162.850885 | 65.5277849 |
| YLDs (Years Lived with Disability) | Ireland | Both | All ages | Gout | Kidney dysfunction | Number | 1990 | 105.0444386 | 162.850885 | 65.5277849 |
| DALYs (Disability-Adjusted Life Years) | Serbia | Both | All ages | Gout | Kidney dysfunction | Number | 1990 | 101.4468261 | 153.670674 | 60.9605556 |
| YLDs (Years Lived with Disability) | Serbia | Both | All ages | Gout | Kidney dysfunction | Number | 1990 | 101.4468261 | 153.670674 | 60.9605556 |
| DALYs (Disability-Adjusted Life Years) | Cameroon | Both | All ages | Gout | Kidney dysfunction | Number | 1990 | 100.7573434 | 151.031653 | 63.0419861 |
| YLDs (Years Lived with Disability) | Cameroon | Both | All ages | Gout | Kidney dysfunction | Number | 1990 | 100.7573434 | 151.031653 | 63.0419861 |
| DALYs (Disability-Adjusted Life Years) | Saudi Arabia | Both | All ages | Gout | Kidney dysfunction | Number | 1990 | 100.0646423 | 149.752042 | 62.8186774 |
| YLDs (Years Lived with Disability) | Saudi Arabia | Both | All ages | Gout | Kidney dysfunction | Number | 1990 | 100.0646423 | 149.752042 | 62.8186774 |
| DALYs (Disability-Adjusted Life Years) | Uruguay | Both | All ages | Gout | Kidney dysfunction | Number | 1990 | 99.56518773 | 156.916064 | 61.930213 |
| YLDs (Years Lived with Disability) | Uruguay | Both | All ages | Gout | Kidney dysfunction | Number | 1990 | 99.56518773 | 156.916064 | 61.930213 |
| DALYs (Disability-Adjusted Life Years) | Azerbaijan | Both | All ages | Gout | Kidney dysfunction | Number | 1990 | 98.75819574 | 145.851297 | 61.4354378 |
| YLDs (Years Lived with Disability) | Azerbaijan | Both | All ages | Gout | Kidney dysfunction | Number | 1990 | 98.75819574 | 145.851297 | 61.4354378 |
| DALYs (Disability-Adjusted Life Years) | Afghanistan | Both | All ages | Gout | Kidney dysfunction | Number | 1990 | 98.45012647 | 149.711645 | 59.8885887 |
| YLDs (Years Lived with Disability) | Afghanistan | Both | All ages | Gout | Kidney dysfunction | Number | 1990 | 98.45012647 | 149.711645 | 59.8885887 |
| DALYs (Disability-Adjusted Life Years) | Zimbabwe | Both | All ages | Gout | Kidney dysfunction | Number | 1990 | 87.97624471 | 133.826025 | 54.318446 |
| YLDs (Years Lived with Disability) | Zimbabwe | Both | All ages | Gout | Kidney dysfunction | Number | 1990 | 87.97624471 | 133.826025 | 54.318446 |
| DALYs (Disability-Adjusted Life Years) | Colombia | Both | All ages | Gout | Kidney dysfunction | Number | 1990 | 86.94710638 | 130.639965 | 53.9481827 |
| YLDs (Years Lived with Disability) | Colombia | Both | All ages | Gout | Kidney dysfunction | Number | 1990 | 86.94710638 | 130.639965 | 53.9481827 |
| DALYs (Disability-Adjusted Life Years) | Syrian Arab Republic | Both | All ages | Gout | Kidney dysfunction | Number | 1990 | 82.73253335 | 126.617675 | 51.8773643 |
| YLDs (Years Lived with Disability) | Syrian Arab Republic | Both | All ages | Gout | Kidney dysfunction | Number | 1990 | 82.73253335 | 126.617675 | 51.8773643 |
| DALYs (Disability-Adjusted Life Years) | United Republic of Tanzania | Both | All ages | Gout | Kidney dysfunction | Number | 1990 | 82.29047364 | 126.911375 | 50.2483078 |
| YLDs (Years Lived with Disability) | United Republic of Tanzania | Both | All ages | Gout | Kidney dysfunction | Number | 1990 | 82.29047364 | 126.911375 | 50.2483078 |
| DALYs (Disability-Adjusted Life Years) | Angola | Both | All ages | Gout | Kidney dysfunction | Number | 1990 | 77.86382371 | 117.317859 | 48.4874349 |
| YLDs (Years Lived with Disability) | Angola | Both | All ages | Gout | Kidney dysfunction | Number | 1990 | 77.86382371 | 117.317859 | 48.4874349 |
| DALYs (Disability-Adjusted Life Years) | Republic of Moldova | Both | All ages | Gout | Kidney dysfunction | Number | 1990 | 74.62548417 | 111.848692 | 47.5466155 |
| YLDs (Years Lived with Disability) | Republic of Moldova | Both | All ages | Gout | Kidney dysfunction | Number | 1990 | 74.62548417 | 111.848692 | 47.5466155 |
| DALYs (Disability-Adjusted Life Years) | Tunisia | Both | All ages | Gout | Kidney dysfunction | Number | 1990 | 72.7553578 | 109.385412 | 43.9154213 |
| YLDs (Years Lived with Disability) | Tunisia | Both | All ages | Gout | Kidney dysfunction | Number | 1990 | 72.7553578 | 109.385412 | 43.9154213 |
| DALYs (Disability-Adjusted Life Years) | Lithuania | Both | All ages | Gout | Kidney dysfunction | Number | 1990 | 71.71669241 | 107.563724 | 44.3311758 |
| YLDs (Years Lived with Disability) | Lithuania | Both | All ages | Gout | Kidney dysfunction | Number | 1990 | 71.71669241 | 107.563724 | 44.3311758 |
| DALYs (Disability-Adjusted Life Years) | Slovakia | Both | All ages | Gout | Kidney dysfunction | Number | 1990 | 70.79397669 | 107.18264 | 44.1454813 |
| YLDs (Years Lived with Disability) | Slovakia | Both | All ages | Gout | Kidney dysfunction | Number | 1990 | 70.79397669 | 107.18264 | 44.1454813 |
| DALYs (Disability-Adjusted Life Years) | Kenya | Both | All ages | Gout | Kidney dysfunction | Number | 1990 | 69.97521908 | 106.064358 | 43.2421745 |
| YLDs (Years Lived with Disability) | Kenya | Both | All ages | Gout | Kidney dysfunction | Number | 1990 | 69.97521908 | 106.064358 | 43.2421745 |
| DALYs (Disability-Adjusted Life Years) | Cambodia | Both | All ages | Gout | Kidney dysfunction | Number | 1990 | 68.12796839 | 103.433309 | 43.0569858 |
| YLDs (Years Lived with Disability) | Cambodia | Both | All ages | Gout | Kidney dysfunction | Number | 1990 | 68.12796839 | 103.433309 | 43.0569858 |
| DALYs (Disability-Adjusted Life Years) | Croatia | Both | All ages | Gout | Kidney dysfunction | Number | 1990 | 64.32508082 | 97.4949785 | 40.22154 |
| YLDs (Years Lived with Disability) | Croatia | Both | All ages | Gout | Kidney dysfunction | Number | 1990 | 64.32508082 | 97.4949785 | 40.22154 |
| DALYs (Disability-Adjusted Life Years) | Ivory Coast | Both | All ages | Gout | Kidney dysfunction | Number | 1990 | 64.10038263 | 99.9474773 | 39.9513094 |
| YLDs (Years Lived with Disability) | Ivory Coast | Both | All ages | Gout | Kidney dysfunction | Number | 1990 | 64.10038263 | 99.9474773 | 39.9513094 |
| DALYs (Disability-Adjusted Life Years) | Peru | Both | All ages | Gout | Kidney dysfunction | Number | 1990 | 62.69819308 | 93.7061497 | 39.8708473 |
| YLDs (Years Lived with Disability) | Peru | Both | All ages | Gout | Kidney dysfunction | Number | 1990 | 62.69819308 | 93.7061497 | 39.8708473 |
| DALYs (Disability-Adjusted Life Years) | Burkina Faso | Both | All ages | Gout | Kidney dysfunction | Number | 1990 | 61.87587789 | 91.7953785 | 38.4143944 |
| YLDs (Years Lived with Disability) | Burkina Faso | Both | All ages | Gout | Kidney dysfunction | Number | 1990 | 61.87587789 | 91.7953785 | 38.4143944 |
| DALYs (Disability-Adjusted Life Years) | Kyrgyzstan | Both | All ages | Gout | Kidney dysfunction | Number | 1990 | 60.41790063 | 89.0040127 | 37.8421893 |
| YLDs (Years Lived with Disability) | Kyrgyzstan | Both | All ages | Gout | Kidney dysfunction | Number | 1990 | 60.41790063 | 89.0040127 | 37.8421893 |
| DALYs (Disability-Adjusted Life Years) | Cuba | Both | All ages | Gout | Kidney dysfunction | Number | 1990 | 59.99895465 | 89.9929839 | 35.3983951 |
| YLDs (Years Lived with Disability) | Cuba | Both | All ages | Gout | Kidney dysfunction | Number | 1990 | 59.99895465 | 89.9929839 | 35.3983951 |
| DALYs (Disability-Adjusted Life Years) | Mali | Both | All ages | Gout | Kidney dysfunction | Number | 1990 | 59.0994357 | 88.4636502 | 37.2448477 |
| YLDs (Years Lived with Disability) | Mali | Both | All ages | Gout | Kidney dysfunction | Number | 1990 | 59.0994357 | 88.4636502 | 37.2448477 |
| DALYs (Disability-Adjusted Life Years) | Uganda | Both | All ages | Gout | Kidney dysfunction | Number | 1990 | 56.26711584 | 88.9186264 | 34.6212837 |
| YLDs (Years Lived with Disability) | Uganda | Both | All ages | Gout | Kidney dysfunction | Number | 1990 | 56.26711584 | 88.9186264 | 34.6212837 |
| DALYs (Disability-Adjusted Life Years) | Yemen | Both | All ages | Gout | Kidney dysfunction | Number | 1990 | 55.84612358 | 84.9167562 | 34.8686485 |
| YLDs (Years Lived with Disability) | Yemen | Both | All ages | Gout | Kidney dysfunction | Number | 1990 | 55.84612358 | 84.9167562 | 34.8686485 |
| DALYs (Disability-Adjusted Life Years) | Singapore | Both | All ages | Gout | Kidney dysfunction | Number | 1990 | 55.78931956 | 84.120111 | 34.3708337 |
| YLDs (Years Lived with Disability) | Singapore | Both | All ages | Gout | Kidney dysfunction | Number | 1990 | 55.78931956 | 84.120111 | 34.3708337 |
| DALYs (Disability-Adjusted Life Years) | Latvia | Both | All ages | Gout | Kidney dysfunction | Number | 1990 | 54.82342698 | 83.2028897 | 33.5852318 |
| YLDs (Years Lived with Disability) | Latvia | Both | All ages | Gout | Kidney dysfunction | Number | 1990 | 54.82342698 | 83.2028897 | 33.5852318 |
| DALYs (Disability-Adjusted Life Years) | Ghana | Both | All ages | Gout | Kidney dysfunction | Number | 1990 | 54.75305236 | 83.1850081 | 33.2448151 |
| YLDs (Years Lived with Disability) | Ghana | Both | All ages | Gout | Kidney dysfunction | Number | 1990 | 54.75305236 | 83.1850081 | 33.2448151 |
| DALYs (Disability-Adjusted Life Years) | Guinea | Both | All ages | Gout | Kidney dysfunction | Number | 1990 | 53.81302237 | 79.5748688 | 34.0834356 |
| YLDs (Years Lived with Disability) | Guinea | Both | All ages | Gout | Kidney dysfunction | Number | 1990 | 53.81302237 | 79.5748688 | 34.0834356 |
| DALYs (Disability-Adjusted Life Years) | Venezuela (Bolivarian Republic of) | Both | All ages | Gout | Kidney dysfunction | Number | 1990 | 52.16758685 | 77.434304 | 32.5652315 |
| YLDs (Years Lived with Disability) | Venezuela (Bolivarian Republic of) | Both | All ages | Gout | Kidney dysfunction | Number | 1990 | 52.16758685 | 77.434304 | 32.5652315 |
| DALYs (Disability-Adjusted Life Years) | Armenia | Both | All ages | Gout | Kidney dysfunction | Number | 1990 | 50.54460635 | 76.4458789 | 32.0888604 |
| YLDs (Years Lived with Disability) | Armenia | Both | All ages | Gout | Kidney dysfunction | Number | 1990 | 50.54460635 | 76.4458789 | 32.0888604 |
| DALYs (Disability-Adjusted Life Years) | Tajikistan | Both | All ages | Gout | Kidney dysfunction | Number | 1990 | 47.68761265 | 71.6245435 | 29.8170367 |
| YLDs (Years Lived with Disability) | Tajikistan | Both | All ages | Gout | Kidney dysfunction | Number | 1990 | 47.68761265 | 71.6245435 | 29.8170367 |
| DALYs (Disability-Adjusted Life Years) | Mozambique | Both | All ages | Gout | Kidney dysfunction | Number | 1990 | 44.21737487 | 66.9700237 | 26.9865643 |
| YLDs (Years Lived with Disability) | Mozambique | Both | All ages | Gout | Kidney dysfunction | Number | 1990 | 44.21737487 | 66.9700237 | 26.9865643 |
| DALYs (Disability-Adjusted Life Years) | Senegal | Both | All ages | Gout | Kidney dysfunction | Number | 1990 | 43.19776569 | 65.7569608 | 26.8318321 |
| YLDs (Years Lived with Disability) | Senegal | Both | All ages | Gout | Kidney dysfunction | Number | 1990 | 43.19776569 | 65.7569608 | 26.8318321 |
| DALYs (Disability-Adjusted Life Years) | Chad | Both | All ages | Gout | Kidney dysfunction | Number | 1990 | 42.73866385 | 64.5288882 | 26.1241593 |
| YLDs (Years Lived with Disability) | Chad | Both | All ages | Gout | Kidney dysfunction | Number | 1990 | 42.73866385 | 64.5288882 | 26.1241593 |
| DALYs (Disability-Adjusted Life Years) | Niger | Both | All ages | Gout | Kidney dysfunction | Number | 1990 | 40.81008942 | 61.435092 | 25.0983009 |
| YLDs (Years Lived with Disability) | Niger | Both | All ages | Gout | Kidney dysfunction | Number | 1990 | 40.81008942 | 61.435092 | 25.0983009 |
| DALYs (Disability-Adjusted Life Years) | Bosnia and Herzegovina | Both | All ages | Gout | Kidney dysfunction | Number | 1990 | 40.74069123 | 60.6482803 | 25.0557953 |
| YLDs (Years Lived with Disability) | Bosnia and Herzegovina | Both | All ages | Gout | Kidney dysfunction | Number | 1990 | 40.74069123 | 60.6482803 | 25.0557953 |
| DALYs (Disability-Adjusted Life Years) | Madagascar | Both | All ages | Gout | Kidney dysfunction | Number | 1990 | 40.32745418 | 61.3477734 | 24.7924235 |
| YLDs (Years Lived with Disability) | Madagascar | Both | All ages | Gout | Kidney dysfunction | Number | 1990 | 40.32745418 | 61.3477734 | 24.7924235 |
| DALYs (Disability-Adjusted Life Years) | Turkmenistan | Both | All ages | Gout | Kidney dysfunction | Number | 1990 | 39.157741 | 59.088398 | 24.6741362 |
| YLDs (Years Lived with Disability) | Turkmenistan | Both | All ages | Gout | Kidney dysfunction | Number | 1990 | 39.157741 | 59.088398 | 24.6741362 |
| DALYs (Disability-Adjusted Life Years) | Lao People's Democratic Republic | Both | All ages | Gout | Kidney dysfunction | Number | 1990 | 38.57688715 | 58.9908739 | 23.6184245 |
| YLDs (Years Lived with Disability) | Lao People's Democratic Republic | Both | All ages | Gout | Kidney dysfunction | Number | 1990 | 38.57688715 | 58.9908739 | 23.6184245 |
| DALYs (Disability-Adjusted Life Years) | Ecuador | Both | All ages | Gout | Kidney dysfunction | Number | 1990 | 33.06532554 | 50.0017738 | 20.4077925 |
| YLDs (Years Lived with Disability) | Ecuador | Both | All ages | Gout | Kidney dysfunction | Number | 1990 | 33.06532554 | 50.0017738 | 20.4077925 |
| DALYs (Disability-Adjusted Life Years) | Sierra Leone | Both | All ages | Gout | Kidney dysfunction | Number | 1990 | 32.76088381 | 49.639382 | 20.0511211 |
| YLDs (Years Lived with Disability) | Sierra Leone | Both | All ages | Gout | Kidney dysfunction | Number | 1990 | 32.76088381 | 49.639382 | 20.0511211 |
| DALYs (Disability-Adjusted Life Years) | Benin | Both | All ages | Gout | Kidney dysfunction | Number | 1990 | 32.49487728 | 48.2019369 | 20.4105128 |
| YLDs (Years Lived with Disability) | Benin | Both | All ages | Gout | Kidney dysfunction | Number | 1990 | 32.49487728 | 48.2019369 | 20.4105128 |
| DALYs (Disability-Adjusted Life Years) | Estonia | Both | All ages | Gout | Kidney dysfunction | Number | 1990 | 31.81741096 | 47.3581593 | 19.1355781 |
| YLDs (Years Lived with Disability) | Estonia | Both | All ages | Gout | Kidney dysfunction | Number | 1990 | 31.81741096 | 47.3581593 | 19.1355781 |
| DALYs (Disability-Adjusted Life Years) | Lebanon | Both | All ages | Gout | Kidney dysfunction | Number | 1990 | 31.21109216 | 47.5082641 | 19.7825497 |
| YLDs (Years Lived with Disability) | Lebanon | Both | All ages | Gout | Kidney dysfunction | Number | 1990 | 31.21109216 | 47.5082641 | 19.7825497 |
| DALYs (Disability-Adjusted Life Years) | Libya | Both | All ages | Gout | Kidney dysfunction | Number | 1990 | 30.10481543 | 45.6126456 | 18.8476747 |
| YLDs (Years Lived with Disability) | Libya | Both | All ages | Gout | Kidney dysfunction | Number | 1990 | 30.10481543 | 45.6126456 | 18.8476747 |
| DALYs (Disability-Adjusted Life Years) | Malawi | Both | All ages | Gout | Kidney dysfunction | Number | 1990 | 29.9504803 | 45.5252307 | 18.4869847 |
| YLDs (Years Lived with Disability) | Malawi | Both | All ages | Gout | Kidney dysfunction | Number | 1990 | 29.9504803 | 45.5252307 | 18.4869847 |
| DALYs (Disability-Adjusted Life Years) | Papua New Guinea | Both | All ages | Gout | Kidney dysfunction | Number | 1990 | 29.17680829 | 44.2096542 | 18.0398304 |
| YLDs (Years Lived with Disability) | Papua New Guinea | Both | All ages | Gout | Kidney dysfunction | Number | 1990 | 29.17680829 | 44.2096542 | 18.0398304 |
| DALYs (Disability-Adjusted Life Years) | Slovenia | Both | All ages | Gout | Kidney dysfunction | Number | 1990 | 26.66613611 | 40.6572741 | 16.0606745 |
| YLDs (Years Lived with Disability) | Slovenia | Both | All ages | Gout | Kidney dysfunction | Number | 1990 | 26.66613611 | 40.6572741 | 16.0606745 |
| DALYs (Disability-Adjusted Life Years) | North Macedonia | Both | All ages | Gout | Kidney dysfunction | Number | 1990 | 26.34905481 | 39.6777872 | 16.3967554 |
| YLDs (Years Lived with Disability) | North Macedonia | Both | All ages | Gout | Kidney dysfunction | Number | 1990 | 26.34905481 | 39.6777872 | 16.3967554 |
| DALYs (Disability-Adjusted Life Years) | Puerto Rico | Both | All ages | Gout | Kidney dysfunction | Number | 1990 | 25.35273243 | 38.0884104 | 15.6170134 |
| YLDs (Years Lived with Disability) | Puerto Rico | Both | All ages | Gout | Kidney dysfunction | Number | 1990 | 25.35273243 | 38.0884104 | 15.6170134 |
| DALYs (Disability-Adjusted Life Years) | Zambia | Both | All ages | Gout | Kidney dysfunction | Number | 1990 | 24.96039177 | 38.51872 | 15.3938455 |
| YLDs (Years Lived with Disability) | Zambia | Both | All ages | Gout | Kidney dysfunction | Number | 1990 | 24.96039177 | 38.51872 | 15.3938455 |
| DALYs (Disability-Adjusted Life Years) | South Sudan | Both | All ages | Gout | Kidney dysfunction | Number | 1990 | 23.50024267 | 35.4353421 | 14.1538744 |
| YLDs (Years Lived with Disability) | South Sudan | Both | All ages | Gout | Kidney dysfunction | Number | 1990 | 23.50024267 | 35.4353421 | 14.1538744 |
| DALYs (Disability-Adjusted Life Years) | Mongolia | Both | All ages | Gout | Kidney dysfunction | Number | 1990 | 23.21562243 | 33.8339359 | 14.7629315 |
| YLDs (Years Lived with Disability) | Mongolia | Both | All ages | Gout | Kidney dysfunction | Number | 1990 | 23.21562243 | 33.8339359 | 14.7629315 |
| DALYs (Disability-Adjusted Life Years) | Congo | Both | All ages | Gout | Kidney dysfunction | Number | 1990 | 22.89092567 | 34.7246664 | 14.1036298 |
| YLDs (Years Lived with Disability) | Congo | Both | All ages | Gout | Kidney dysfunction | Number | 1990 | 22.89092567 | 34.7246664 | 14.1036298 |
| DALYs (Disability-Adjusted Life Years) | Albania | Both | All ages | Gout | Kidney dysfunction | Number | 1990 | 22.16032367 | 33.9215624 | 13.8436371 |
| YLDs (Years Lived with Disability) | Albania | Both | All ages | Gout | Kidney dysfunction | Number | 1990 | 22.16032367 | 33.9215624 | 13.8436371 |
| DALYs (Disability-Adjusted Life Years) | Central African Republic | Both | All ages | Gout | Kidney dysfunction | Number | 1990 | 22.13541535 | 33.4408787 | 13.7657511 |
| YLDs (Years Lived with Disability) | Central African Republic | Both | All ages | Gout | Kidney dysfunction | Number | 1990 | 22.13541535 | 33.4408787 | 13.7657511 |
| DALYs (Disability-Adjusted Life Years) | Rwanda | Both | All ages | Gout | Kidney dysfunction | Number | 1990 | 22.04170653 | 34.0974126 | 13.6382415 |
| YLDs (Years Lived with Disability) | Rwanda | Both | All ages | Gout | Kidney dysfunction | Number | 1990 | 22.04170653 | 34.0974126 | 13.6382415 |
| DALYs (Disability-Adjusted Life Years) | Jordan | Both | All ages | Gout | Kidney dysfunction | Number | 1990 | 21.22396998 | 32.414207 | 13.3800893 |
| YLDs (Years Lived with Disability) | Jordan | Both | All ages | Gout | Kidney dysfunction | Number | 1990 | 21.22396998 | 32.414207 | 13.3800893 |
| DALYs (Disability-Adjusted Life Years) | Liberia | Both | All ages | Gout | Kidney dysfunction | Number | 1990 | 20.33779464 | 30.8423803 | 12.8031172 |
| YLDs (Years Lived with Disability) | Liberia | Both | All ages | Gout | Kidney dysfunction | Number | 1990 | 20.33779464 | 30.8423803 | 12.8031172 |
| DALYs (Disability-Adjusted Life Years) | Dominican Republic | Both | All ages | Gout | Kidney dysfunction | Number | 1990 | 19.87559239 | 30.9435288 | 11.8414204 |
| YLDs (Years Lived with Disability) | Dominican Republic | Both | All ages | Gout | Kidney dysfunction | Number | 1990 | 19.87559239 | 30.9435288 | 11.8414204 |
| DALYs (Disability-Adjusted Life Years) | Burundi | Both | All ages | Gout | Kidney dysfunction | Number | 1990 | 18.99607596 | 28.9639672 | 11.7662234 |
| YLDs (Years Lived with Disability) | Burundi | Both | All ages | Gout | Kidney dysfunction | Number | 1990 | 18.99607596 | 28.9639672 | 11.7662234 |
| DALYs (Disability-Adjusted Life Years) | Bolivia (Plurinational State of) | Both | All ages | Gout | Kidney dysfunction | Number | 1990 | 18.8388261 | 28.5455724 | 11.8487788 |
| YLDs (Years Lived with Disability) | Bolivia (Plurinational State of) | Both | All ages | Gout | Kidney dysfunction | Number | 1990 | 18.8388261 | 28.5455724 | 11.8487788 |
| DALYs (Disability-Adjusted Life Years) | Togo | Both | All ages | Gout | Kidney dysfunction | Number | 1990 | 18.02325491 | 27.0620026 | 11.1202573 |
| YLDs (Years Lived with Disability) | Togo | Both | All ages | Gout | Kidney dysfunction | Number | 1990 | 18.02325491 | 27.0620026 | 11.1202573 |
| DALYs (Disability-Adjusted Life Years) | Cyprus | Both | All ages | Gout | Kidney dysfunction | Number | 1990 | 17.9985722 | 27.6948106 | 10.8779202 |
| YLDs (Years Lived with Disability) | Cyprus | Both | All ages | Gout | Kidney dysfunction | Number | 1990 | 17.9985722 | 27.6948106 | 10.8779202 |
| DALYs (Disability-Adjusted Life Years) | Paraguay | Both | All ages | Gout | Kidney dysfunction | Number | 1990 | 17.72572076 | 25.8992771 | 11.1334649 |
| YLDs (Years Lived with Disability) | Paraguay | Both | All ages | Gout | Kidney dysfunction | Number | 1990 | 17.72572076 | 25.8992771 | 11.1334649 |
| DALYs (Disability-Adjusted Life Years) | Somalia | Both | All ages | Gout | Kidney dysfunction | Number | 1990 | 17.39040529 | 26.287177 | 10.7276273 |
| YLDs (Years Lived with Disability) | Somalia | Both | All ages | Gout | Kidney dysfunction | Number | 1990 | 17.39040529 | 26.287177 | 10.7276273 |
| DALYs (Disability-Adjusted Life Years) | Mauritania | Both | All ages | Gout | Kidney dysfunction | Number | 1990 | 17.12145401 | 26.1727659 | 10.864591 |
| YLDs (Years Lived with Disability) | Mauritania | Both | All ages | Gout | Kidney dysfunction | Number | 1990 | 17.12145401 | 26.1727659 | 10.864591 |
| DALYs (Disability-Adjusted Life Years) | Guatemala | Both | All ages | Gout | Kidney dysfunction | Number | 1990 | 16.89233189 | 26.4820692 | 10.5213721 |
| YLDs (Years Lived with Disability) | Guatemala | Both | All ages | Gout | Kidney dysfunction | Number | 1990 | 16.89233189 | 26.4820692 | 10.5213721 |
| DALYs (Disability-Adjusted Life Years) | Haiti | Both | All ages | Gout | Kidney dysfunction | Number | 1990 | 16.5599266 | 24.9364412 | 10.1769181 |
| YLDs (Years Lived with Disability) | Haiti | Both | All ages | Gout | Kidney dysfunction | Number | 1990 | 16.5599266 | 24.9364412 | 10.1769181 |
| DALYs (Disability-Adjusted Life Years) | Lesotho | Both | All ages | Gout | Kidney dysfunction | Number | 1990 | 16.38090356 | 24.8964775 | 10.416935 |
| YLDs (Years Lived with Disability) | Lesotho | Both | All ages | Gout | Kidney dysfunction | Number | 1990 | 16.38090356 | 24.8964775 | 10.416935 |
| DALYs (Disability-Adjusted Life Years) | Mauritius | Both | All ages | Gout | Kidney dysfunction | Number | 1990 | 15.67072338 | 23.6668176 | 9.91522994 |
| YLDs (Years Lived with Disability) | Mauritius | Both | All ages | Gout | Kidney dysfunction | Number | 1990 | 15.67072338 | 23.6668176 | 9.91522994 |
| DALYs (Disability-Adjusted Life Years) | El Salvador | Both | All ages | Gout | Kidney dysfunction | Number | 1990 | 13.8564931 | 21.0102332 | 8.48050555 |
| YLDs (Years Lived with Disability) | El Salvador | Both | All ages | Gout | Kidney dysfunction | Number | 1990 | 13.8564931 | 21.0102332 | 8.48050555 |
| DALYs (Disability-Adjusted Life Years) | Gabon | Both | All ages | Gout | Kidney dysfunction | Number | 1990 | 13.52054641 | 20.6945736 | 8.47573691 |
| YLDs (Years Lived with Disability) | Gabon | Both | All ages | Gout | Kidney dysfunction | Number | 1990 | 13.52054641 | 20.6945736 | 8.47573691 |
| DALYs (Disability-Adjusted Life Years) | Namibia | Both | All ages | Gout | Kidney dysfunction | Number | 1990 | 13.33102359 | 19.9836432 | 8.20742548 |
| YLDs (Years Lived with Disability) | Namibia | Both | All ages | Gout | Kidney dysfunction | Number | 1990 | 13.33102359 | 19.9836432 | 8.20742548 |
| DALYs (Disability-Adjusted Life Years) | Palestine | Both | All ages | Gout | Kidney dysfunction | Number | 1990 | 13.27564848 | 20.3154221 | 8.3968405 |
| YLDs (Years Lived with Disability) | Palestine | Both | All ages | Gout | Kidney dysfunction | Number | 1990 | 13.27564848 | 20.3154221 | 8.3968405 |
| DALYs (Disability-Adjusted Life Years) | Costa Rica | Both | All ages | Gout | Kidney dysfunction | Number | 1990 | 12.83847111 | 19.0186743 | 7.97350806 |
| YLDs (Years Lived with Disability) | Costa Rica | Both | All ages | Gout | Kidney dysfunction | Number | 1990 | 12.83847111 | 19.0186743 | 7.97350806 |
| DALYs (Disability-Adjusted Life Years) | Luxembourg | Both | All ages | Gout | Kidney dysfunction | Number | 1990 | 12.0207481 | 18.1785419 | 7.31477118 |
| YLDs (Years Lived with Disability) | Luxembourg | Both | All ages | Gout | Kidney dysfunction | Number | 1990 | 12.0207481 | 18.1785419 | 7.31477118 |
| DALYs (Disability-Adjusted Life Years) | Jamaica | Both | All ages | Gout | Kidney dysfunction | Number | 1990 | 11.98217552 | 17.8227631 | 7.18775167 |
| YLDs (Years Lived with Disability) | Jamaica | Both | All ages | Gout | Kidney dysfunction | Number | 1990 | 11.98217552 | 17.8227631 | 7.18775167 |
| DALYs (Disability-Adjusted Life Years) | Botswana | Both | All ages | Gout | Kidney dysfunction | Number | 1990 | 11.23224435 | 16.7798253 | 6.76548363 |
| YLDs (Years Lived with Disability) | Botswana | Both | All ages | Gout | Kidney dysfunction | Number | 1990 | 11.23224435 | 16.7798253 | 6.76548363 |
| DALYs (Disability-Adjusted Life Years) | Nicaragua | Both | All ages | Gout | Kidney dysfunction | Number | 1990 | 10.56622226 | 16.5469551 | 6.52052524 |
| YLDs (Years Lived with Disability) | Nicaragua | Both | All ages | Gout | Kidney dysfunction | Number | 1990 | 10.56622226 | 16.5469551 | 6.52052524 |
| DALYs (Disability-Adjusted Life Years) | Honduras | Both | All ages | Gout | Kidney dysfunction | Number | 1990 | 10.53073718 | 15.9545704 | 6.50774186 |
| YLDs (Years Lived with Disability) | Honduras | Both | All ages | Gout | Kidney dysfunction | Number | 1990 | 10.53073718 | 15.9545704 | 6.50774186 |
| DALYs (Disability-Adjusted Life Years) | Kuwait | Both | All ages | Gout | Kidney dysfunction | Number | 1990 | 10.39181461 | 15.4894967 | 6.63066588 |
| YLDs (Years Lived with Disability) | Kuwait | Both | All ages | Gout | Kidney dysfunction | Number | 1990 | 10.39181461 | 15.4894967 | 6.63066588 |
| DALYs (Disability-Adjusted Life Years) | United Arab Emirates | Both | All ages | Gout | Kidney dysfunction | Number | 1990 | 9.582401082 | 14.319948 | 5.77230026 |
| YLDs (Years Lived with Disability) | United Arab Emirates | Both | All ages | Gout | Kidney dysfunction | Number | 1990 | 9.582401082 | 14.319948 | 5.77230026 |
| DALYs (Disability-Adjusted Life Years) | Malta | Both | All ages | Gout | Kidney dysfunction | Number | 1990 | 8.899253253 | 13.5208495 | 5.48274426 |
| YLDs (Years Lived with Disability) | Malta | Both | All ages | Gout | Kidney dysfunction | Number | 1990 | 8.899253253 | 13.5208495 | 5.48274426 |
| DALYs (Disability-Adjusted Life Years) | Oman | Both | All ages | Gout | Kidney dysfunction | Number | 1990 | 8.678252152 | 13.0180564 | 5.32209685 |
| YLDs (Years Lived with Disability) | Oman | Both | All ages | Gout | Kidney dysfunction | Number | 1990 | 8.678252152 | 13.0180564 | 5.32209685 |
| DALYs (Disability-Adjusted Life Years) | Fiji | Both | All ages | Gout | Kidney dysfunction | Number | 1990 | 8.109741448 | 12.208315 | 5.08403642 |
| YLDs (Years Lived with Disability) | Fiji | Both | All ages | Gout | Kidney dysfunction | Number | 1990 | 8.109741448 | 12.208315 | 5.08403642 |
| DALYs (Disability-Adjusted Life Years) | Montenegro | Both | All ages | Gout | Kidney dysfunction | Number | 1990 | 8.090787482 | 12.1653926 | 4.98053438 |
| YLDs (Years Lived with Disability) | Montenegro | Both | All ages | Gout | Kidney dysfunction | Number | 1990 | 8.090787482 | 12.1653926 | 4.98053438 |
| DALYs (Disability-Adjusted Life Years) | Panama | Both | All ages | Gout | Kidney dysfunction | Number | 1990 | 7.546717298 | 11.3769736 | 4.63504995 |
| YLDs (Years Lived with Disability) | Panama | Both | All ages | Gout | Kidney dysfunction | Number | 1990 | 7.546717298 | 11.3769736 | 4.63504995 |
| DALYs (Disability-Adjusted Life Years) | Eritrea | Both | All ages | Gout | Kidney dysfunction | Number | 1990 | 7.243595387 | 11.246125 | 4.35106352 |
| YLDs (Years Lived with Disability) | Eritrea | Both | All ages | Gout | Kidney dysfunction | Number | 1990 | 7.243595387 | 11.246125 | 4.35106352 |
| DALYs (Disability-Adjusted Life Years) | Eswatini | Both | All ages | Gout | Kidney dysfunction | Number | 1990 | 6.76043253 | 10.4471971 | 4.16627673 |
| YLDs (Years Lived with Disability) | Eswatini | Both | All ages | Gout | Kidney dysfunction | Number | 1990 | 6.76043253 | 10.4471971 | 4.16627673 |
| DALYs (Disability-Adjusted Life Years) | Guinea-Bissau | Both | All ages | Gout | Kidney dysfunction | Number | 1990 | 6.148302143 | 9.4130106 | 3.81665997 |
| YLDs (Years Lived with Disability) | Guinea-Bissau | Both | All ages | Gout | Kidney dysfunction | Number | 1990 | 6.148302143 | 9.4130106 | 3.81665997 |
| DALYs (Disability-Adjusted Life Years) | Iceland | Both | All ages | Gout | Kidney dysfunction | Number | 1990 | 5.671319953 | 8.60075206 | 3.46601619 |
| YLDs (Years Lived with Disability) | Iceland | Both | All ages | Gout | Kidney dysfunction | Number | 1990 | 5.671319953 | 8.60075206 | 3.46601619 |
| DALYs (Disability-Adjusted Life Years) | Gambia | Both | All ages | Gout | Kidney dysfunction | Number | 1990 | 5.480285998 | 8.37812173 | 3.43074796 |
| YLDs (Years Lived with Disability) | Gambia | Both | All ages | Gout | Kidney dysfunction | Number | 1990 | 5.480285998 | 8.37812173 | 3.43074796 |
| DALYs (Disability-Adjusted Life Years) | Trinidad and Tobago | Both | All ages | Gout | Kidney dysfunction | Number | 1990 | 5.355257322 | 7.97457241 | 3.25369806 |
| YLDs (Years Lived with Disability) | Trinidad and Tobago | Both | All ages | Gout | Kidney dysfunction | Number | 1990 | 5.355257322 | 7.97457241 | 3.25369806 |
| DALYs (Disability-Adjusted Life Years) | Timor-Leste | Both | All ages | Gout | Kidney dysfunction | Number | 1990 | 4.546532217 | 6.79548057 | 2.86085767 |
| YLDs (Years Lived with Disability) | Timor-Leste | Both | All ages | Gout | Kidney dysfunction | Number | 1990 | 4.546532217 | 6.79548057 | 2.86085767 |
| DALYs (Disability-Adjusted Life Years) | Equatorial Guinea | Both | All ages | Gout | Kidney dysfunction | Number | 1990 | 3.732683445 | 5.55680972 | 2.36235059 |
| YLDs (Years Lived with Disability) | Equatorial Guinea | Both | All ages | Gout | Kidney dysfunction | Number | 1990 | 3.732683445 | 5.55680972 | 2.36235059 |
| DALYs (Disability-Adjusted Life Years) | Cabo Verde | Both | All ages | Gout | Kidney dysfunction | Number | 1990 | 3.675183776 | 5.54091009 | 2.26907572 |
| YLDs (Years Lived with Disability) | Cabo Verde | Both | All ages | Gout | Kidney dysfunction | Number | 1990 | 3.675183776 | 5.54091009 | 2.26907572 |
| DALYs (Disability-Adjusted Life Years) | Bhutan | Both | All ages | Gout | Kidney dysfunction | Number | 1990 | 3.563473531 | 5.37424024 | 2.23336893 |
| YLDs (Years Lived with Disability) | Bhutan | Both | All ages | Gout | Kidney dysfunction | Number | 1990 | 3.563473531 | 5.37424024 | 2.23336893 |
| DALYs (Disability-Adjusted Life Years) | Brunei Darussalam | Both | All ages | Gout | Kidney dysfunction | Number | 1990 | 2.90013553 | 4.31937087 | 1.79297212 |
| YLDs (Years Lived with Disability) | Brunei Darussalam | Both | All ages | Gout | Kidney dysfunction | Number | 1990 | 2.90013553 | 4.31937087 | 1.79297212 |
| DALYs (Disability-Adjusted Life Years) | Bahrain | Both | All ages | Gout | Kidney dysfunction | Number | 1990 | 2.790659754 | 4.24841897 | 1.7160716 |
| YLDs (Years Lived with Disability) | Bahrain | Both | All ages | Gout | Kidney dysfunction | Number | 1990 | 2.790659754 | 4.24841897 | 1.7160716 |
| DALYs (Disability-Adjusted Life Years) | Solomon Islands | Both | All ages | Gout | Kidney dysfunction | Number | 1990 | 2.776963223 | 4.13229839 | 1.75241769 |
| YLDs (Years Lived with Disability) | Solomon Islands | Both | All ages | Gout | Kidney dysfunction | Number | 1990 | 2.776963223 | 4.13229839 | 1.75241769 |
| DALYs (Disability-Adjusted Life Years) | Guyana | Both | All ages | Gout | Kidney dysfunction | Number | 1990 | 2.197558605 | 3.25824305 | 1.35328577 |
| YLDs (Years Lived with Disability) | Guyana | Both | All ages | Gout | Kidney dysfunction | Number | 1990 | 2.197558605 | 3.25824305 | 1.35328577 |
| DALYs (Disability-Adjusted Life Years) | Qatar | Both | All ages | Gout | Kidney dysfunction | Number | 1990 | 2.167912382 | 3.20879459 | 1.30580302 |
| YLDs (Years Lived with Disability) | Qatar | Both | All ages | Gout | Kidney dysfunction | Number | 1990 | 2.167912382 | 3.20879459 | 1.30580302 |
| DALYs (Disability-Adjusted Life Years) | Samoa | Both | All ages | Gout | Kidney dysfunction | Number | 1990 | 2.132098348 | 3.20645718 | 1.35521051 |
| YLDs (Years Lived with Disability) | Samoa | Both | All ages | Gout | Kidney dysfunction | Number | 1990 | 2.132098348 | 3.20645718 | 1.35521051 |
| DALYs (Disability-Adjusted Life Years) | Barbados | Both | All ages | Gout | Kidney dysfunction | Number | 1990 | 1.918432416 | 2.88390703 | 1.1713247 |
| YLDs (Years Lived with Disability) | Barbados | Both | All ages | Gout | Kidney dysfunction | Number | 1990 | 1.918432416 | 2.88390703 | 1.1713247 |
| DALYs (Disability-Adjusted Life Years) | Maldives | Both | All ages | Gout | Kidney dysfunction | Number | 1990 | 1.794632519 | 2.78173105 | 1.13391247 |
| YLDs (Years Lived with Disability) | Maldives | Both | All ages | Gout | Kidney dysfunction | Number | 1990 | 1.794632519 | 2.78173105 | 1.13391247 |
| DALYs (Disability-Adjusted Life Years) | Monaco | Both | All ages | Gout | Kidney dysfunction | Number | 1990 | 1.690910441 | 2.5027156 | 1.00765225 |
| YLDs (Years Lived with Disability) | Monaco | Both | All ages | Gout | Kidney dysfunction | Number | 1990 | 1.690910441 | 2.5027156 | 1.00765225 |
| DALYs (Disability-Adjusted Life Years) | Comoros | Both | All ages | Gout | Kidney dysfunction | Number | 1990 | 1.628858762 | 2.59461036 | 0.94979854 |
| YLDs (Years Lived with Disability) | Comoros | Both | All ages | Gout | Kidney dysfunction | Number | 1990 | 1.628858762 | 2.59461036 | 0.94979854 |
| DALYs (Disability-Adjusted Life Years) | Suriname | Both | All ages | Gout | Kidney dysfunction | Number | 1990 | 1.626186375 | 2.49052278 | 1.00366848 |
| YLDs (Years Lived with Disability) | Suriname | Both | All ages | Gout | Kidney dysfunction | Number | 1990 | 1.626186375 | 2.49052278 | 1.00366848 |
| DALYs (Disability-Adjusted Life Years) | Guam | Both | All ages | Gout | Kidney dysfunction | Number | 1990 | 1.600723994 | 2.43662306 | 0.99793382 |
| YLDs (Years Lived with Disability) | Guam | Both | All ages | Gout | Kidney dysfunction | Number | 1990 | 1.600723994 | 2.43662306 | 0.99793382 |
| DALYs (Disability-Adjusted Life Years) | Tonga | Both | All ages | Gout | Kidney dysfunction | Number | 1990 | 1.322922779 | 1.96338525 | 0.81200607 |
| YLDs (Years Lived with Disability) | Tonga | Both | All ages | Gout | Kidney dysfunction | Number | 1990 | 1.322922779 | 1.96338525 | 0.81200607 |
| DALYs (Disability-Adjusted Life Years) | Andorra | Both | All ages | Gout | Kidney dysfunction | Number | 1990 | 1.291729757 | 1.94815308 | 0.78791873 |
| YLDs (Years Lived with Disability) | Andorra | Both | All ages | Gout | Kidney dysfunction | Number | 1990 | 1.291729757 | 1.94815308 | 0.78791873 |
| DALYs (Disability-Adjusted Life Years) | Seychelles | Both | All ages | Gout | Kidney dysfunction | Number | 1990 | 1.260798501 | 1.94550368 | 0.76865823 |
| YLDs (Years Lived with Disability) | Seychelles | Both | All ages | Gout | Kidney dysfunction | Number | 1990 | 1.260798501 | 1.94550368 | 0.76865823 |
| DALYs (Disability-Adjusted Life Years) | Vanuatu | Both | All ages | Gout | Kidney dysfunction | Number | 1990 | 1.258748334 | 1.95469934 | 0.77680158 |
| YLDs (Years Lived with Disability) | Vanuatu | Both | All ages | Gout | Kidney dysfunction | Number | 1990 | 1.258748334 | 1.95469934 | 0.77680158 |
| DALYs (Disability-Adjusted Life Years) | Micronesia (Federated States of) | Both | All ages | Gout | Kidney dysfunction | Number | 1990 | 1.188644252 | 1.81148817 | 0.74709593 |
| YLDs (Years Lived with Disability) | Micronesia (Federated States of) | Both | All ages | Gout | Kidney dysfunction | Number | 1990 | 1.188644252 | 1.81148817 | 0.74709593 |
| DALYs (Disability-Adjusted Life Years) | Sao Tome and Principe | Both | All ages | Gout | Kidney dysfunction | Number | 1990 | 1.109983014 | 1.68221492 | 0.69759149 |
| YLDs (Years Lived with Disability) | Sao Tome and Principe | Both | All ages | Gout | Kidney dysfunction | Number | 1990 | 1.109983014 | 1.68221492 | 0.69759149 |
| DALYs (Disability-Adjusted Life Years) | Greenland | Both | All ages | Gout | Kidney dysfunction | Number | 1990 | 1.069241241 | 1.6152039 | 0.67199651 |
| YLDs (Years Lived with Disability) | Greenland | Both | All ages | Gout | Kidney dysfunction | Number | 1990 | 1.069241241 | 1.6152039 | 0.67199651 |
| DALYs (Disability-Adjusted Life Years) | Djibouti | Both | All ages | Gout | Kidney dysfunction | Number | 1990 | 0.983012024 | 1.5199651 | 0.60538224 |
| YLDs (Years Lived with Disability) | Djibouti | Both | All ages | Gout | Kidney dysfunction | Number | 1990 | 0.983012024 | 1.5199651 | 0.60538224 |
| DALYs (Disability-Adjusted Life Years) | Bahamas | Both | All ages | Gout | Kidney dysfunction | Number | 1990 | 0.958469417 | 1.45282054 | 0.60817404 |
| YLDs (Years Lived with Disability) | Bahamas | Both | All ages | Gout | Kidney dysfunction | Number | 1990 | 0.958469417 | 1.45282054 | 0.60817404 |
| DALYs (Disability-Adjusted Life Years) | San Marino | Both | All ages | Gout | Kidney dysfunction | Number | 1990 | 0.766090785 | 1.17535511 | 0.4684062 |
| YLDs (Years Lived with Disability) | San Marino | Both | All ages | Gout | Kidney dysfunction | Number | 1990 | 0.766090785 | 1.17535511 | 0.4684062 |
| DALYs (Disability-Adjusted Life Years) | Kiribati | Both | All ages | Gout | Kidney dysfunction | Number | 1990 | 0.750950999 | 1.15770117 | 0.47450479 |
| YLDs (Years Lived with Disability) | Kiribati | Both | All ages | Gout | Kidney dysfunction | Number | 1990 | 0.750950999 | 1.15770117 | 0.47450479 |
| DALYs (Disability-Adjusted Life Years) | Belize | Both | All ages | Gout | Kidney dysfunction | Number | 1990 | 0.621020331 | 0.92737696 | 0.38637443 |
| YLDs (Years Lived with Disability) | Belize | Both | All ages | Gout | Kidney dysfunction | Number | 1990 | 0.621020331 | 0.92737696 | 0.38637443 |
| DALYs (Disability-Adjusted Life Years) | American Samoa | Both | All ages | Gout | Kidney dysfunction | Number | 1990 | 0.609297947 | 0.92960573 | 0.3834263 |
| YLDs (Years Lived with Disability) | American Samoa | Both | All ages | Gout | Kidney dysfunction | Number | 1990 | 0.609297947 | 0.92960573 | 0.3834263 |
| DALYs (Disability-Adjusted Life Years) | Saint Lucia | Both | All ages | Gout | Kidney dysfunction | Number | 1990 | 0.531859694 | 0.8218845 | 0.32043213 |
| YLDs (Years Lived with Disability) | Saint Lucia | Both | All ages | Gout | Kidney dysfunction | Number | 1990 | 0.531859694 | 0.8218845 | 0.32043213 |
| DALYs (Disability-Adjusted Life Years) | Northern Mariana Islands | Both | All ages | Gout | Kidney dysfunction | Number | 1990 | 0.529118316 | 0.79377465 | 0.31924485 |
| YLDs (Years Lived with Disability) | Northern Mariana Islands | Both | All ages | Gout | Kidney dysfunction | Number | 1990 | 0.529118316 | 0.79377465 | 0.31924485 |
| DALYs (Disability-Adjusted Life Years) | United States Virgin Islands | Both | All ages | Gout | Kidney dysfunction | Number | 1990 | 0.520555963 | 0.79246173 | 0.31840637 |
| YLDs (Years Lived with Disability) | United States Virgin Islands | Both | All ages | Gout | Kidney dysfunction | Number | 1990 | 0.520555963 | 0.79246173 | 0.31840637 |
| DALYs (Disability-Adjusted Life Years) | Grenada | Both | All ages | Gout | Kidney dysfunction | Number | 1990 | 0.475150321 | 0.73370514 | 0.29622347 |
| YLDs (Years Lived with Disability) | Grenada | Both | All ages | Gout | Kidney dysfunction | Number | 1990 | 0.475150321 | 0.73370514 | 0.29622347 |
| DALYs (Disability-Adjusted Life Years) | Saint Vincent and the Grenadines | Both | All ages | Gout | Kidney dysfunction | Number | 1990 | 0.411311488 | 0.62556856 | 0.25682015 |
| YLDs (Years Lived with Disability) | Saint Vincent and the Grenadines | Both | All ages | Gout | Kidney dysfunction | Number | 1990 | 0.411311488 | 0.62556856 | 0.25682015 |
| DALYs (Disability-Adjusted Life Years) | Dominica | Both | All ages | Gout | Kidney dysfunction | Number | 1990 | 0.391774827 | 0.58918279 | 0.23742911 |
| YLDs (Years Lived with Disability) | Dominica | Both | All ages | Gout | Kidney dysfunction | Number | 1990 | 0.391774827 | 0.58918279 | 0.23742911 |
| DALYs (Disability-Adjusted Life Years) | Bermuda | Both | All ages | Gout | Kidney dysfunction | Number | 1990 | 0.378987141 | 0.57491941 | 0.23455734 |
| YLDs (Years Lived with Disability) | Bermuda | Both | All ages | Gout | Kidney dysfunction | Number | 1990 | 0.378987141 | 0.57491941 | 0.23455734 |
| DALYs (Disability-Adjusted Life Years) | Antigua and Barbuda | Both | All ages | Gout | Kidney dysfunction | Number | 1990 | 0.34399082 | 0.52282187 | 0.20654671 |
| YLDs (Years Lived with Disability) | Antigua and Barbuda | Both | All ages | Gout | Kidney dysfunction | Number | 1990 | 0.34399082 | 0.52282187 | 0.20654671 |
| DALYs (Disability-Adjusted Life Years) | Marshall Islands | Both | All ages | Gout | Kidney dysfunction | Number | 1990 | 0.333865359 | 0.50588609 | 0.20919898 |
| YLDs (Years Lived with Disability) | Marshall Islands | Both | All ages | Gout | Kidney dysfunction | Number | 1990 | 0.333865359 | 0.50588609 | 0.20919898 |
| DALYs (Disability-Adjusted Life Years) | Cook Islands | Both | All ages | Gout | Kidney dysfunction | Number | 1990 | 0.300437828 | 0.45330608 | 0.18968342 |
| YLDs (Years Lived with Disability) | Cook Islands | Both | All ages | Gout | Kidney dysfunction | Number | 1990 | 0.300437828 | 0.45330608 | 0.18968342 |
| DALYs (Disability-Adjusted Life Years) | Saint Kitts and Nevis | Both | All ages | Gout | Kidney dysfunction | Number | 1990 | 0.25654092 | 0.38919373 | 0.15210588 |
| YLDs (Years Lived with Disability) | Saint Kitts and Nevis | Both | All ages | Gout | Kidney dysfunction | Number | 1990 | 0.25654092 | 0.38919373 | 0.15210588 |
| DALYs (Disability-Adjusted Life Years) | Palau | Both | All ages | Gout | Kidney dysfunction | Number | 1990 | 0.2556515 | 0.39052146 | 0.1589338 |
| YLDs (Years Lived with Disability) | Palau | Both | All ages | Gout | Kidney dysfunction | Number | 1990 | 0.2556515 | 0.39052146 | 0.1589338 |
| DALYs (Disability-Adjusted Life Years) | Tuvalu | Both | All ages | Gout | Kidney dysfunction | Number | 1990 | 0.132184221 | 0.20031953 | 0.08213522 |
| YLDs (Years Lived with Disability) | Tuvalu | Both | All ages | Gout | Kidney dysfunction | Number | 1990 | 0.132184221 | 0.20031953 | 0.08213522 |
| DALYs (Disability-Adjusted Life Years) | Nauru | Both | All ages | Gout | Kidney dysfunction | Number | 1990 | 0.113993616 | 0.17616496 | 0.07155269 |
| YLDs (Years Lived with Disability) | Nauru | Both | All ages | Gout | Kidney dysfunction | Number | 1990 | 0.113993616 | 0.17616496 | 0.07155269 |
| DALYs (Disability-Adjusted Life Years) | Niue | Both | All ages | Gout | Kidney dysfunction | Number | 1990 | 0.058120041 | 0.08722483 | 0.03505707 |
| YLDs (Years Lived with Disability) | Niue | Both | All ages | Gout | Kidney dysfunction | Number | 1990 | 0.058120041 | 0.08722483 | 0.03505707 |
| DALYs (Disability-Adjusted Life Years) | Tokelau | Both | All ages | Gout | Kidney dysfunction | Number | 1990 | 0.030582215 | 0.04632952 | 0.01916977 |
| YLDs (Years Lived with Disability) | Tokelau | Both | All ages | Gout | Kidney dysfunction | Number | 1990 | 0.030582215 | 0.04632952 | 0.01916977 |

**Appendix 4：The age-standardized mortality rate (ASMR) and age-standardized YLD rate of gout attributable to renal dysfunction across 204 countries in 1990**

| measure | location | sex | age | cause | rei | metric | year | val | upper | lower |
| --- | --- | --- | --- | --- | --- | --- | --- | --- | --- | --- |
| DALYs (Disability-Adjusted Life Years) | New Zealand | Both | Age-standardized | Gout | Kidney dysfunction | Rate | 1990 | 5.125126093 | 7.63899762 | 3.1513745 | |
| YLDs (Years Lived with Disability) | New Zealand | Both | Age-standardized | Gout | Kidney dysfunction | Rate | 1990 | 5.125126093 | 7.63899762 | 3.1513745 |
| DALYs (Disability-Adjusted Life Years) | United States of America | Both | Age-standardized | Gout | Kidney dysfunction | Rate | 1990 | 4.121291708 | 6.09135411 | 2.55672678 |
| YLDs (Years Lived with Disability) | United States of America | Both | Age-standardized | Gout | Kidney dysfunction | Rate | 1990 | 4.121291708 | 6.09135411 | 2.55672678 |
| DALYs (Disability-Adjusted Life Years) | Greenland | Both | Age-standardized | Gout | Kidney dysfunction | Rate | 1990 | 3.984278847 | 5.94366289 | 2.50500859 |
| YLDs (Years Lived with Disability) | Greenland | Both | Age-standardized | Gout | Kidney dysfunction | Rate | 1990 | 3.984278847 | 5.94366289 | 2.50500859 |
| DALYs (Disability-Adjusted Life Years) | Canada | Both | Age-standardized | Gout | Kidney dysfunction | Rate | 1990 | 3.975781728 | 6.083529 | 2.43762641 |
| YLDs (Years Lived with Disability) | Canada | Both | Age-standardized | Gout | Kidney dysfunction | Rate | 1990 | 3.975781728 | 6.083529 | 2.43762641 |
| DALYs (Disability-Adjusted Life Years) | Australia | Both | Age-standardized | Gout | Kidney dysfunction | Rate | 1990 | 3.946599837 | 5.94800419 | 2.48915321 |
| YLDs (Years Lived with Disability) | Australia | Both | Age-standardized | Gout | Kidney dysfunction | Rate | 1990 | 3.946599837 | 5.94800419 | 2.48915321 |
| DALYs (Disability-Adjusted Life Years) | Northern Mariana Islands | Both | Age-standardized | Gout | Kidney dysfunction | Rate | 1990 | 3.506062325 | 5.18593503 | 2.11248926 |
| YLDs (Years Lived with Disability) | Northern Mariana Islands | Both | Age-standardized | Gout | Kidney dysfunction | Rate | 1990 | 3.506062325 | 5.18593503 | 2.11248926 |
| DALYs (Disability-Adjusted Life Years) | Taiwan (Province of China) | Both | Age-standardized | Gout | Kidney dysfunction | Rate | 1990 | 3.376466071 | 4.9451658 | 2.18479254 |
| YLDs (Years Lived with Disability) | Taiwan (Province of China) | Both | Age-standardized | Gout | Kidney dysfunction | Rate | 1990 | 3.376466071 | 4.9451658 | 2.18479254 |
| DALYs (Disability-Adjusted Life Years) | Brunei Darussalam | Both | Age-standardized | Gout | Kidney dysfunction | Rate | 1990 | 3.193019722 | 4.82492039 | 1.99095281 |
| YLDs (Years Lived with Disability) | Brunei Darussalam | Both | Age-standardized | Gout | Kidney dysfunction | Rate | 1990 | 3.193019722 | 4.82492039 | 1.99095281 |
| DALYs (Disability-Adjusted Life Years) | American Samoa | Both | Age-standardized | Gout | Kidney dysfunction | Rate | 1990 | 3.110604237 | 4.67163325 | 1.92917503 |
| YLDs (Years Lived with Disability) | American Samoa | Both | Age-standardized | Gout | Kidney dysfunction | Rate | 1990 | 3.110604237 | 4.67163325 | 1.92917503 |
| DALYs (Disability-Adjusted Life Years) | Nauru | Both | Age-standardized | Gout | Kidney dysfunction | Rate | 1990 | 3.057482257 | 4.68694528 | 1.8833159 |
| YLDs (Years Lived with Disability) | Nauru | Both | Age-standardized | Gout | Kidney dysfunction | Rate | 1990 | 3.057482257 | 4.68694528 | 1.8833159 |
| DALYs (Disability-Adjusted Life Years) | Japan | Both | Age-standardized | Gout | Kidney dysfunction | Rate | 1990 | 2.858797977 | 4.27903195 | 1.77729471 |
| YLDs (Years Lived with Disability) | Japan | Both | Age-standardized | Gout | Kidney dysfunction | Rate | 1990 | 2.858797977 | 4.27903195 | 1.77729471 |
| DALYs (Disability-Adjusted Life Years) | Palau | Both | Age-standardized | Gout | Kidney dysfunction | Rate | 1990 | 2.84532761 | 4.29508807 | 1.76798095 |
| YLDs (Years Lived with Disability) | Palau | Both | Age-standardized | Gout | Kidney dysfunction | Rate | 1990 | 2.84532761 | 4.29508807 | 1.76798095 |
| DALYs (Disability-Adjusted Life Years) | Singapore | Both | Age-standardized | Gout | Kidney dysfunction | Rate | 1990 | 2.827318332 | 4.2569104 | 1.73694377 |
| YLDs (Years Lived with Disability) | Singapore | Both | Age-standardized | Gout | Kidney dysfunction | Rate | 1990 | 2.827318332 | 4.2569104 | 1.73694377 |
| DALYs (Disability-Adjusted Life Years) | Samoa | Both | Age-standardized | Gout | Kidney dysfunction | Rate | 1990 | 2.751936862 | 4.08269092 | 1.74053611 |
| YLDs (Years Lived with Disability) | Samoa | Both | Age-standardized | Gout | Kidney dysfunction | Rate | 1990 | 2.751936862 | 4.08269092 | 1.74053611 |
| DALYs (Disability-Adjusted Life Years) | South Africa | Both | Age-standardized | Gout | Kidney dysfunction | Rate | 1990 | 2.655521714 | 3.95810962 | 1.66819782 |
| YLDs (Years Lived with Disability) | South Africa | Both | Age-standardized | Gout | Kidney dysfunction | Rate | 1990 | 2.655521714 | 3.95810962 | 1.66819782 |
| DALYs (Disability-Adjusted Life Years) | Cook Islands | Both | Age-standardized | Gout | Kidney dysfunction | Rate | 1990 | 2.655452898 | 3.94735179 | 1.66104706 |
| YLDs (Years Lived with Disability) | Cook Islands | Both | Age-standardized | Gout | Kidney dysfunction | Rate | 1990 | 2.655452898 | 3.94735179 | 1.66104706 |
| DALYs (Disability-Adjusted Life Years) | Micronesia (Federated States of) | Both | Age-standardized | Gout | Kidney dysfunction | Rate | 1990 | 2.654364847 | 4.02747717 | 1.65227365 |
| YLDs (Years Lived with Disability) | Micronesia (Federated States of) | Both | Age-standardized | Gout | Kidney dysfunction | Rate | 1990 | 2.654364847 | 4.02747717 | 1.65227365 |
| DALYs (Disability-Adjusted Life Years) | Fiji | Both | Age-standardized | Gout | Kidney dysfunction | Rate | 1990 | 2.637724473 | 3.85803403 | 1.62791961 |
| YLDs (Years Lived with Disability) | Fiji | Both | Age-standardized | Gout | Kidney dysfunction | Rate | 1990 | 2.637724473 | 3.85803403 | 1.62791961 |
| DALYs (Disability-Adjusted Life Years) | Eswatini | Both | Age-standardized | Gout | Kidney dysfunction | Rate | 1990 | 2.637578265 | 3.96875384 | 1.60808766 |
| YLDs (Years Lived with Disability) | Eswatini | Both | Age-standardized | Gout | Kidney dysfunction | Rate | 1990 | 2.637578265 | 3.96875384 | 1.60808766 |
| DALYs (Disability-Adjusted Life Years) | Tonga | Both | Age-standardized | Gout | Kidney dysfunction | Rate | 1990 | 2.627552045 | 3.895906 | 1.61303702 |
| YLDs (Years Lived with Disability) | Tonga | Both | Age-standardized | Gout | Kidney dysfunction | Rate | 1990 | 2.627552045 | 3.895906 | 1.61303702 |
| DALYs (Disability-Adjusted Life Years) | Niue | Both | Age-standardized | Gout | Kidney dysfunction | Rate | 1990 | 2.541457474 | 3.84728741 | 1.54168502 |
| YLDs (Years Lived with Disability) | Niue | Both | Age-standardized | Gout | Kidney dysfunction | Rate | 1990 | 2.541457474 | 3.84728741 | 1.54168502 |
| DALYs (Disability-Adjusted Life Years) | Gabon | Both | Age-standardized | Gout | Kidney dysfunction | Rate | 1990 | 2.527607165 | 3.82452114 | 1.59352937 |
| YLDs (Years Lived with Disability) | Gabon | Both | Age-standardized | Gout | Kidney dysfunction | Rate | 1990 | 2.527607165 | 3.82452114 | 1.59352937 |
| DALYs (Disability-Adjusted Life Years) | Cameroon | Both | Age-standardized | Gout | Kidney dysfunction | Rate | 1990 | 2.527036284 | 3.76406739 | 1.57002559 |
| YLDs (Years Lived with Disability) | Cameroon | Both | Age-standardized | Gout | Kidney dysfunction | Rate | 1990 | 2.527036284 | 3.76406739 | 1.57002559 |
| DALYs (Disability-Adjusted Life Years) | Nigeria | Both | Age-standardized | Gout | Kidney dysfunction | Rate | 1990 | 2.523368522 | 3.74792938 | 1.58732728 |
| YLDs (Years Lived with Disability) | Nigeria | Both | Age-standardized | Gout | Kidney dysfunction | Rate | 1990 | 2.523368522 | 3.74792938 | 1.58732728 |
| DALYs (Disability-Adjusted Life Years) | Malaysia | Both | Age-standardized | Gout | Kidney dysfunction | Rate | 1990 | 2.517063605 | 3.77435966 | 1.59934477 |
| YLDs (Years Lived with Disability) | Malaysia | Both | Age-standardized | Gout | Kidney dysfunction | Rate | 1990 | 2.517063605 | 3.77435966 | 1.59934477 |
| DALYs (Disability-Adjusted Life Years) | Democratic Republic of the Congo | Both | Age-standardized | Gout | Kidney dysfunction | Rate | 1990 | 2.516171271 | 3.63913323 | 1.58893348 |
| YLDs (Years Lived with Disability) | Democratic Republic of the Congo | Both | Age-standardized | Gout | Kidney dysfunction | Rate | 1990 | 2.516171271 | 3.63913323 | 1.58893348 |
| DALYs (Disability-Adjusted Life Years) | Guam | Both | Age-standardized | Gout | Kidney dysfunction | Rate | 1990 | 2.508054028 | 3.78576609 | 1.56415313 |
| YLDs (Years Lived with Disability) | Guam | Both | Age-standardized | Gout | Kidney dysfunction | Rate | 1990 | 2.508054028 | 3.78576609 | 1.56415313 |
| DALYs (Disability-Adjusted Life Years) | Maldives | Both | Age-standardized | Gout | Kidney dysfunction | Rate | 1990 | 2.49419572 | 3.81083543 | 1.54592724 |
| YLDs (Years Lived with Disability) | Maldives | Both | Age-standardized | Gout | Kidney dysfunction | Rate | 1990 | 2.49419572 | 3.81083543 | 1.54592724 |
| DALYs (Disability-Adjusted Life Years) | Solomon Islands | Both | Age-standardized | Gout | Kidney dysfunction | Rate | 1990 | 2.489293501 | 3.66498042 | 1.56520554 |
| YLDs (Years Lived with Disability) | Solomon Islands | Both | Age-standardized | Gout | Kidney dysfunction | Rate | 1990 | 2.489293501 | 3.66498042 | 1.56520554 |
| DALYs (Disability-Adjusted Life Years) | Uruguay | Both | Age-standardized | Gout | Kidney dysfunction | Rate | 1990 | 2.477494578 | 3.8833631 | 1.55643024 |
| YLDs (Years Lived with Disability) | Uruguay | Both | Age-standardized | Gout | Kidney dysfunction | Rate | 1990 | 2.477494578 | 3.8833631 | 1.55643024 |
| DALYs (Disability-Adjusted Life Years) | United Kingdom | Both | Age-standardized | Gout | Kidney dysfunction | Rate | 1990 | 2.473900377 | 3.71727312 | 1.52749373 |
| YLDs (Years Lived with Disability) | United Kingdom | Both | Age-standardized | Gout | Kidney dysfunction | Rate | 1990 | 2.473900377 | 3.71727312 | 1.52749373 |
| DALYs (Disability-Adjusted Life Years) | Ireland | Both | Age-standardized | Gout | Kidney dysfunction | Rate | 1990 | 2.465776611 | 3.77792432 | 1.54888766 |
| YLDs (Years Lived with Disability) | Ireland | Both | Age-standardized | Gout | Kidney dysfunction | Rate | 1990 | 2.465776611 | 3.77792432 | 1.54888766 |
| DALYs (Disability-Adjusted Life Years) | Congo | Both | Age-standardized | Gout | Kidney dysfunction | Rate | 1990 | 2.46056854 | 3.62466972 | 1.54674343 |
| YLDs (Years Lived with Disability) | Congo | Both | Age-standardized | Gout | Kidney dysfunction | Rate | 1990 | 2.46056854 | 3.62466972 | 1.54674343 |
| DALYs (Disability-Adjusted Life Years) | Vanuatu | Both | Age-standardized | Gout | Kidney dysfunction | Rate | 1990 | 2.448695533 | 3.72726633 | 1.50632042 |
| YLDs (Years Lived with Disability) | Vanuatu | Both | Age-standardized | Gout | Kidney dysfunction | Rate | 1990 | 2.448695533 | 3.72726633 | 1.50632042 |
| DALYs (Disability-Adjusted Life Years) | Israel | Both | Age-standardized | Gout | Kidney dysfunction | Rate | 1990 | 2.43093606 | 3.66493085 | 1.46723722 |
| YLDs (Years Lived with Disability) | Israel | Both | Age-standardized | Gout | Kidney dysfunction | Rate | 1990 | 2.43093606 | 3.66493085 | 1.46723722 |
| DALYs (Disability-Adjusted Life Years) | Greece | Both | Age-standardized | Gout | Kidney dysfunction | Rate | 1990 | 2.419699459 | 3.74964061 | 1.53832058 |
| YLDs (Years Lived with Disability) | Greece | Both | Age-standardized | Gout | Kidney dysfunction | Rate | 1990 | 2.419699459 | 3.74964061 | 1.53832058 |
| DALYs (Disability-Adjusted Life Years) | Thailand | Both | Age-standardized | Gout | Kidney dysfunction | Rate | 1990 | 2.417256322 | 3.52871584 | 1.56043211 |
| YLDs (Years Lived with Disability) | Thailand | Both | Age-standardized | Gout | Kidney dysfunction | Rate | 1990 | 2.417256322 | 3.52871584 | 1.56043211 |
| DALYs (Disability-Adjusted Life Years) | Chile | Both | Age-standardized | Gout | Kidney dysfunction | Rate | 1990 | 2.412568948 | 3.64047229 | 1.46383097 |
| YLDs (Years Lived with Disability) | Chile | Both | Age-standardized | Gout | Kidney dysfunction | Rate | 1990 | 2.412568948 | 3.64047229 | 1.46383097 |
| DALYs (Disability-Adjusted Life Years) | Zimbabwe | Both | Age-standardized | Gout | Kidney dysfunction | Rate | 1990 | 2.403912376 | 3.58995757 | 1.48237576 |
| YLDs (Years Lived with Disability) | Zimbabwe | Both | Age-standardized | Gout | Kidney dysfunction | Rate | 1990 | 2.403912376 | 3.58995757 | 1.48237576 |
| DALYs (Disability-Adjusted Life Years) | Republic of Korea | Both | Age-standardized | Gout | Kidney dysfunction | Rate | 1990 | 2.396734371 | 3.64526016 | 1.49863729 |
| YLDs (Years Lived with Disability) | Republic of Korea | Both | Age-standardized | Gout | Kidney dysfunction | Rate | 1990 | 2.396734371 | 3.64526016 | 1.49863729 |
| DALYs (Disability-Adjusted Life Years) | Angola | Both | Age-standardized | Gout | Kidney dysfunction | Rate | 1990 | 2.395604869 | 3.53469512 | 1.50423021 |
| YLDs (Years Lived with Disability) | Angola | Both | Age-standardized | Gout | Kidney dysfunction | Rate | 1990 | 2.395604869 | 3.53469512 | 1.50423021 |
| DALYs (Disability-Adjusted Life Years) | Nepal | Both | Age-standardized | Gout | Kidney dysfunction | Rate | 1990 | 2.385985087 | 3.57106409 | 1.51936303 |
| YLDs (Years Lived with Disability) | Nepal | Both | Age-standardized | Gout | Kidney dysfunction | Rate | 1990 | 2.385985087 | 3.57106409 | 1.51936303 |
| DALYs (Disability-Adjusted Life Years) | Kiribati | Both | Age-standardized | Gout | Kidney dysfunction | Rate | 1990 | 2.366834964 | 3.55916394 | 1.48376946 |
| YLDs (Years Lived with Disability) | Kiribati | Both | Age-standardized | Gout | Kidney dysfunction | Rate | 1990 | 2.366834964 | 3.55916394 | 1.48376946 |
| DALYs (Disability-Adjusted Life Years) | Mongolia | Both | Age-standardized | Gout | Kidney dysfunction | Rate | 1990 | 2.355213672 | 3.42683917 | 1.51132072 |
| YLDs (Years Lived with Disability) | Mongolia | Both | Age-standardized | Gout | Kidney dysfunction | Rate | 1990 | 2.355213672 | 3.42683917 | 1.51132072 |
| DALYs (Disability-Adjusted Life Years) | Tokelau | Both | Age-standardized | Gout | Kidney dysfunction | Rate | 1990 | 2.348522505 | 3.53952496 | 1.47675533 |
| YLDs (Years Lived with Disability) | Tokelau | Both | Age-standardized | Gout | Kidney dysfunction | Rate | 1990 | 2.348522505 | 3.53952496 | 1.47675533 |
| DALYs (Disability-Adjusted Life Years) | Namibia | Both | Age-standardized | Gout | Kidney dysfunction | Rate | 1990 | 2.330568611 | 3.43209004 | 1.41839423 |
| YLDs (Years Lived with Disability) | Namibia | Both | Age-standardized | Gout | Kidney dysfunction | Rate | 1990 | 2.330568611 | 3.43209004 | 1.41839423 |
| DALYs (Disability-Adjusted Life Years) | Andorra | Both | Age-standardized | Gout | Kidney dysfunction | Rate | 1990 | 2.327511035 | 3.50076401 | 1.42780476 |
| YLDs (Years Lived with Disability) | Andorra | Both | Age-standardized | Gout | Kidney dysfunction | Rate | 1990 | 2.327511035 | 3.50076401 | 1.42780476 |
| DALYs (Disability-Adjusted Life Years) | Mauritius | Both | Age-standardized | Gout | Kidney dysfunction | Rate | 1990 | 2.307543612 | 3.46247068 | 1.44776029 |
| YLDs (Years Lived with Disability) | Mauritius | Both | Age-standardized | Gout | Kidney dysfunction | Rate | 1990 | 2.307543612 | 3.46247068 | 1.44776029 |
| DALYs (Disability-Adjusted Life Years) | Marshall Islands | Both | Age-standardized | Gout | Kidney dysfunction | Rate | 1990 | 2.305848875 | 3.50026367 | 1.40917049 |
| YLDs (Years Lived with Disability) | Marshall Islands | Both | Age-standardized | Gout | Kidney dysfunction | Rate | 1990 | 2.305848875 | 3.50026367 | 1.40917049 |
| DALYs (Disability-Adjusted Life Years) | Uzbekistan | Both | Age-standardized | Gout | Kidney dysfunction | Rate | 1990 | 2.29371466 | 3.36949038 | 1.41863175 |
| YLDs (Years Lived with Disability) | Uzbekistan | Both | Age-standardized | Gout | Kidney dysfunction | Rate | 1990 | 2.29371466 | 3.36949038 | 1.41863175 |
| DALYs (Disability-Adjusted Life Years) | Central African Republic | Both | Age-standardized | Gout | Kidney dysfunction | Rate | 1990 | 2.28661244 | 3.38472077 | 1.40307427 |
| YLDs (Years Lived with Disability) | Central African Republic | Both | Age-standardized | Gout | Kidney dysfunction | Rate | 1990 | 2.28661244 | 3.38472077 | 1.40307427 |
| DALYs (Disability-Adjusted Life Years) | Botswana | Both | Age-standardized | Gout | Kidney dysfunction | Rate | 1990 | 2.280216032 | 3.36326801 | 1.37652892 |
| YLDs (Years Lived with Disability) | Botswana | Both | Age-standardized | Gout | Kidney dysfunction | Rate | 1990 | 2.280216032 | 3.36326801 | 1.37652892 |
| DALYs (Disability-Adjusted Life Years) | Turkmenistan | Both | Age-standardized | Gout | Kidney dysfunction | Rate | 1990 | 2.24828868 | 3.3977677 | 1.42338333 |
| YLDs (Years Lived with Disability) | Turkmenistan | Both | Age-standardized | Gout | Kidney dysfunction | Rate | 1990 | 2.24828868 | 3.3977677 | 1.42338333 |
| DALYs (Disability-Adjusted Life Years) | Tuvalu | Both | Age-standardized | Gout | Kidney dysfunction | Rate | 1990 | 2.238610805 | 3.31498534 | 1.3947104 |
| YLDs (Years Lived with Disability) | Tuvalu | Both | Age-standardized | Gout | Kidney dysfunction | Rate | 1990 | 2.238610805 | 3.31498534 | 1.3947104 |
| DALYs (Disability-Adjusted Life Years) | Equatorial Guinea | Both | Age-standardized | Gout | Kidney dysfunction | Rate | 1990 | 2.22965161 | 3.25930603 | 1.42285644 |
| YLDs (Years Lived with Disability) | Equatorial Guinea | Both | Age-standardized | Gout | Kidney dysfunction | Rate | 1990 | 2.22965161 | 3.25930603 | 1.42285644 |
| DALYs (Disability-Adjusted Life Years) | Cyprus | Both | Age-standardized | Gout | Kidney dysfunction | Rate | 1990 | 2.225895384 | 3.38824588 | 1.37059434 |
| YLDs (Years Lived with Disability) | Cyprus | Both | Age-standardized | Gout | Kidney dysfunction | Rate | 1990 | 2.225895384 | 3.38824588 | 1.37059434 |
| DALYs (Disability-Adjusted Life Years) | Spain | Both | Age-standardized | Gout | Kidney dysfunction | Rate | 1990 | 2.225576672 | 3.36079763 | 1.37727408 |
| YLDs (Years Lived with Disability) | Spain | Both | Age-standardized | Gout | Kidney dysfunction | Rate | 1990 | 2.225576672 | 3.36079763 | 1.37727408 |
| DALYs (Disability-Adjusted Life Years) | Seychelles | Both | Age-standardized | Gout | Kidney dysfunction | Rate | 1990 | 2.224158653 | 3.42905357 | 1.36134797 |
| YLDs (Years Lived with Disability) | Seychelles | Both | Age-standardized | Gout | Kidney dysfunction | Rate | 1990 | 2.224158653 | 3.42905357 | 1.36134797 |
| DALYs (Disability-Adjusted Life Years) | Democratic People's Republic of Korea | Both | Age-standardized | Gout | Kidney dysfunction | Rate | 1990 | 2.217538469 | 3.27490459 | 1.38168971 |
| YLDs (Years Lived with Disability) | Democratic People's Republic of Korea | Both | Age-standardized | Gout | Kidney dysfunction | Rate | 1990 | 2.217538469 | 3.27490459 | 1.38168971 |
| DALYs (Disability-Adjusted Life Years) | United Arab Emirates | Both | Age-standardized | Gout | Kidney dysfunction | Rate | 1990 | 2.211463907 | 3.37735046 | 1.3740273 |
| YLDs (Years Lived with Disability) | United Arab Emirates | Both | Age-standardized | Gout | Kidney dysfunction | Rate | 1990 | 2.211463907 | 3.37735046 | 1.3740273 |
| DALYs (Disability-Adjusted Life Years) | China | Both | Age-standardized | Gout | Kidney dysfunction | Rate | 1990 | 2.195535148 | 3.29120381 | 1.3509616 |
| YLDs (Years Lived with Disability) | China | Both | Age-standardized | Gout | Kidney dysfunction | Rate | 1990 | 2.195535148 | 3.29120381 | 1.3509616 |
| DALYs (Disability-Adjusted Life Years) | Sri Lanka | Both | Age-standardized | Gout | Kidney dysfunction | Rate | 1990 | 2.153678164 | 3.21221737 | 1.34242048 |
| YLDs (Years Lived with Disability) | Sri Lanka | Both | Age-standardized | Gout | Kidney dysfunction | Rate | 1990 | 2.153678164 | 3.21221737 | 1.34242048 |
| DALYs (Disability-Adjusted Life Years) | Monaco | Both | Age-standardized | Gout | Kidney dysfunction | Rate | 1990 | 2.14052428 | 3.19836459 | 1.28504636 |
| YLDs (Years Lived with Disability) | Monaco | Both | Age-standardized | Gout | Kidney dysfunction | Rate | 1990 | 2.14052428 | 3.19836459 | 1.28504636 |
| DALYs (Disability-Adjusted Life Years) | Georgia | Both | Age-standardized | Gout | Kidney dysfunction | Rate | 1990 | 2.134007541 | 3.20212967 | 1.38072141 |
| YLDs (Years Lived with Disability) | Georgia | Both | Age-standardized | Gout | Kidney dysfunction | Rate | 1990 | 2.134007541 | 3.20212967 | 1.38072141 |
| DALYs (Disability-Adjusted Life Years) | Iran (Islamic Republic of) | Both | Age-standardized | Gout | Kidney dysfunction | Rate | 1990 | 2.130930711 | 3.20810269 | 1.31173727 |
| YLDs (Years Lived with Disability) | Iran (Islamic Republic of) | Both | Age-standardized | Gout | Kidney dysfunction | Rate | 1990 | 2.130930711 | 3.20810269 | 1.31173727 |
| DALYs (Disability-Adjusted Life Years) | Kyrgyzstan | Both | Age-standardized | Gout | Kidney dysfunction | Rate | 1990 | 2.12717331 | 3.1273469 | 1.3351582 |
| YLDs (Years Lived with Disability) | Kyrgyzstan | Both | Age-standardized | Gout | Kidney dysfunction | Rate | 1990 | 2.12717331 | 3.1273469 | 1.3351582 |
| DALYs (Disability-Adjusted Life Years) | Azerbaijan | Both | Age-standardized | Gout | Kidney dysfunction | Rate | 1990 | 2.126675954 | 3.11647701 | 1.32031346 |
| YLDs (Years Lived with Disability) | Azerbaijan | Both | Age-standardized | Gout | Kidney dysfunction | Rate | 1990 | 2.126675954 | 3.11647701 | 1.32031346 |
| DALYs (Disability-Adjusted Life Years) | Luxembourg | Both | Age-standardized | Gout | Kidney dysfunction | Rate | 1990 | 2.124302895 | 3.22016143 | 1.29814404 |
| YLDs (Years Lived with Disability) | Luxembourg | Both | Age-standardized | Gout | Kidney dysfunction | Rate | 1990 | 2.124302895 | 3.22016143 | 1.29814404 |
| DALYs (Disability-Adjusted Life Years) | Kazakhstan | Both | Age-standardized | Gout | Kidney dysfunction | Rate | 1990 | 2.111850502 | 3.20027226 | 1.32507761 |
| YLDs (Years Lived with Disability) | Kazakhstan | Both | Age-standardized | Gout | Kidney dysfunction | Rate | 1990 | 2.111850502 | 3.20027226 | 1.32507761 |
| DALYs (Disability-Adjusted Life Years) | Malta | Both | Age-standardized | Gout | Kidney dysfunction | Rate | 1990 | 2.109202752 | 3.14865562 | 1.30252608 |
| YLDs (Years Lived with Disability) | Malta | Both | Age-standardized | Gout | Kidney dysfunction | Rate | 1990 | 2.109202752 | 3.14865562 | 1.30252608 |
| DALYs (Disability-Adjusted Life Years) | Lao People's Democratic Republic | Both | Age-standardized | Gout | Kidney dysfunction | Rate | 1990 | 2.103279536 | 3.148871 | 1.28972348 |
| YLDs (Years Lived with Disability) | Lao People's Democratic Republic | Both | Age-standardized | Gout | Kidney dysfunction | Rate | 1990 | 2.103279536 | 3.148871 | 1.28972348 |
| DALYs (Disability-Adjusted Life Years) | Qatar | Both | Age-standardized | Gout | Kidney dysfunction | Rate | 1990 | 2.098864644 | 3.19749814 | 1.27778544 |
| YLDs (Years Lived with Disability) | Qatar | Both | Age-standardized | Gout | Kidney dysfunction | Rate | 1990 | 2.098864644 | 3.19749814 | 1.27778544 |
| DALYs (Disability-Adjusted Life Years) | Belgium | Both | Age-standardized | Gout | Kidney dysfunction | Rate | 1990 | 2.083729023 | 3.16789317 | 1.32671406 |
| YLDs (Years Lived with Disability) | Belgium | Both | Age-standardized | Gout | Kidney dysfunction | Rate | 1990 | 2.083729023 | 3.16789317 | 1.32671406 |
| DALYs (Disability-Adjusted Life Years) | Lesotho | Both | Age-standardized | Gout | Kidney dysfunction | Rate | 1990 | 2.06949466 | 3.10538449 | 1.29997642 |
| YLDs (Years Lived with Disability) | Lesotho | Both | Age-standardized | Gout | Kidney dysfunction | Rate | 1990 | 2.06949466 | 3.10538449 | 1.29997642 |
| DALYs (Disability-Adjusted Life Years) | San Marino | Both | Age-standardized | Gout | Kidney dysfunction | Rate | 1990 | 2.050663126 | 3.14415094 | 1.26248409 |
| YLDs (Years Lived with Disability) | San Marino | Both | Age-standardized | Gout | Kidney dysfunction | Rate | 1990 | 2.050663126 | 3.14415094 | 1.26248409 |
| DALYs (Disability-Adjusted Life Years) | Papua New Guinea | Both | Age-standardized | Gout | Kidney dysfunction | Rate | 1990 | 2.050006732 | 3.06170506 | 1.28420922 |
| YLDs (Years Lived with Disability) | Papua New Guinea | Both | Age-standardized | Gout | Kidney dysfunction | Rate | 1990 | 2.050006732 | 3.06170506 | 1.28420922 |
| DALYs (Disability-Adjusted Life Years) | Argentina | Both | Age-standardized | Gout | Kidney dysfunction | Rate | 1990 | 2.041725126 | 3.10090711 | 1.24449966 |
| YLDs (Years Lived with Disability) | Argentina | Both | Age-standardized | Gout | Kidney dysfunction | Rate | 1990 | 2.041725126 | 3.10090711 | 1.24449966 |
| DALYs (Disability-Adjusted Life Years) | Germany | Both | Age-standardized | Gout | Kidney dysfunction | Rate | 1990 | 2.037491261 | 3.14896611 | 1.23597551 |
| YLDs (Years Lived with Disability) | Germany | Both | Age-standardized | Gout | Kidney dysfunction | Rate | 1990 | 2.037491261 | 3.14896611 | 1.23597551 |
| DALYs (Disability-Adjusted Life Years) | Netherlands | Both | Age-standardized | Gout | Kidney dysfunction | Rate | 1990 | 2.033039032 | 3.02296344 | 1.24829852 |
| YLDs (Years Lived with Disability) | Netherlands | Both | Age-standardized | Gout | Kidney dysfunction | Rate | 1990 | 2.033039032 | 3.02296344 | 1.24829852 |
| DALYs (Disability-Adjusted Life Years) | Switzerland | Both | Age-standardized | Gout | Kidney dysfunction | Rate | 1990 | 2.026332909 | 3.09324975 | 1.25441744 |
| YLDs (Years Lived with Disability) | Switzerland | Both | Age-standardized | Gout | Kidney dysfunction | Rate | 1990 | 2.026332909 | 3.09324975 | 1.25441744 |
| DALYs (Disability-Adjusted Life Years) | Armenia | Both | Age-standardized | Gout | Kidney dysfunction | Rate | 1990 | 2.016196336 | 3.05253337 | 1.2535619 |
| YLDs (Years Lived with Disability) | Armenia | Both | Age-standardized | Gout | Kidney dysfunction | Rate | 1990 | 2.016196336 | 3.05253337 | 1.2535619 |
| DALYs (Disability-Adjusted Life Years) | Timor-Leste | Both | Age-standardized | Gout | Kidney dysfunction | Rate | 1990 | 2.015965325 | 3.01922325 | 1.23676283 |
| YLDs (Years Lived with Disability) | Timor-Leste | Both | Age-standardized | Gout | Kidney dysfunction | Rate | 1990 | 2.015965325 | 3.01922325 | 1.23676283 |
| DALYs (Disability-Adjusted Life Years) | Philippines | Both | Age-standardized | Gout | Kidney dysfunction | Rate | 1990 | 1.990705873 | 2.93994682 | 1.24687537 |
| YLDs (Years Lived with Disability) | Philippines | Both | Age-standardized | Gout | Kidney dysfunction | Rate | 1990 | 1.990705873 | 2.93994682 | 1.24687537 |
| DALYs (Disability-Adjusted Life Years) | Indonesia | Both | Age-standardized | Gout | Kidney dysfunction | Rate | 1990 | 1.98409742 | 2.94284798 | 1.2352302 |
| YLDs (Years Lived with Disability) | Indonesia | Both | Age-standardized | Gout | Kidney dysfunction | Rate | 1990 | 1.98409742 | 2.94284798 | 1.2352302 |
| DALYs (Disability-Adjusted Life Years) | Denmark | Both | Age-standardized | Gout | Kidney dysfunction | Rate | 1990 | 1.982463247 | 3.03835958 | 1.22712298 |
| YLDs (Years Lived with Disability) | Denmark | Both | Age-standardized | Gout | Kidney dysfunction | Rate | 1990 | 1.982463247 | 3.03835958 | 1.22712298 |
| DALYs (Disability-Adjusted Life Years) | Kuwait | Both | Age-standardized | Gout | Kidney dysfunction | Rate | 1990 | 1.932681782 | 2.91950341 | 1.21577149 |
| YLDs (Years Lived with Disability) | Kuwait | Both | Age-standardized | Gout | Kidney dysfunction | Rate | 1990 | 1.932681782 | 2.91950341 | 1.21577149 |
| DALYs (Disability-Adjusted Life Years) | Liberia | Both | Age-standardized | Gout | Kidney dysfunction | Rate | 1990 | 1.931473227 | 2.89473029 | 1.21393088 |
| YLDs (Years Lived with Disability) | Liberia | Both | Age-standardized | Gout | Kidney dysfunction | Rate | 1990 | 1.931473227 | 2.89473029 | 1.21393088 |
| DALYs (Disability-Adjusted Life Years) | Myanmar | Both | Age-standardized | Gout | Kidney dysfunction | Rate | 1990 | 1.92165059 | 2.88905966 | 1.19197339 |
| YLDs (Years Lived with Disability) | Myanmar | Both | Age-standardized | Gout | Kidney dysfunction | Rate | 1990 | 1.92165059 | 2.88905966 | 1.19197339 |
| DALYs (Disability-Adjusted Life Years) | Austria | Both | Age-standardized | Gout | Kidney dysfunction | Rate | 1990 | 1.910503809 | 2.90085405 | 1.17318229 |
| YLDs (Years Lived with Disability) | Austria | Both | Age-standardized | Gout | Kidney dysfunction | Rate | 1990 | 1.910503809 | 2.90085405 | 1.17318229 |
| DALYs (Disability-Adjusted Life Years) | Ivory Coast | Both | Age-standardized | Gout | Kidney dysfunction | Rate | 1990 | 1.888257311 | 2.87379066 | 1.19819839 |
| YLDs (Years Lived with Disability) | Ivory Coast | Both | Age-standardized | Gout | Kidney dysfunction | Rate | 1990 | 1.888257311 | 2.87379066 | 1.19819839 |
| DALYs (Disability-Adjusted Life Years) | Iceland | Both | Age-standardized | Gout | Kidney dysfunction | Rate | 1990 | 1.888156874 | 2.85253727 | 1.16225786 |
| YLDs (Years Lived with Disability) | Iceland | Both | Age-standardized | Gout | Kidney dysfunction | Rate | 1990 | 1.888156874 | 2.85253727 | 1.16225786 |
| DALYs (Disability-Adjusted Life Years) | Saudi Arabia | Both | Age-standardized | Gout | Kidney dysfunction | Rate | 1990 | 1.884677585 | 2.7798373 | 1.19749468 |
| YLDs (Years Lived with Disability) | Saudi Arabia | Both | Age-standardized | Gout | Kidney dysfunction | Rate | 1990 | 1.884677585 | 2.7798373 | 1.19749468 |
| DALYs (Disability-Adjusted Life Years) | Tajikistan | Both | Age-standardized | Gout | Kidney dysfunction | Rate | 1990 | 1.879683193 | 2.7910299 | 1.16973359 |
| YLDs (Years Lived with Disability) | Tajikistan | Both | Age-standardized | Gout | Kidney dysfunction | Rate | 1990 | 1.879683193 | 2.7910299 | 1.16973359 |
| DALYs (Disability-Adjusted Life Years) | Mauritania | Both | Age-standardized | Gout | Kidney dysfunction | Rate | 1990 | 1.853260042 | 2.78756744 | 1.19021518 |
| YLDs (Years Lived with Disability) | Mauritania | Both | Age-standardized | Gout | Kidney dysfunction | Rate | 1990 | 1.853260042 | 2.78756744 | 1.19021518 |
| DALYs (Disability-Adjusted Life Years) | Iraq | Both | Age-standardized | Gout | Kidney dysfunction | Rate | 1990 | 1.843823996 | 2.79667241 | 1.1106499 |
| YLDs (Years Lived with Disability) | Iraq | Both | Age-standardized | Gout | Kidney dysfunction | Rate | 1990 | 1.843823996 | 2.79667241 | 1.1106499 |
| DALYs (Disability-Adjusted Life Years) | Republic of Moldova | Both | Age-standardized | Gout | Kidney dysfunction | Rate | 1990 | 1.835350077 | 2.70608681 | 1.17917063 |
| YLDs (Years Lived with Disability) | Republic of Moldova | Both | Age-standardized | Gout | Kidney dysfunction | Rate | 1990 | 1.835350077 | 2.70608681 | 1.17917063 |
| DALYs (Disability-Adjusted Life Years) | Jordan | Both | Age-standardized | Gout | Kidney dysfunction | Rate | 1990 | 1.821247062 | 2.74586376 | 1.14321942 |
| YLDs (Years Lived with Disability) | Jordan | Both | Age-standardized | Gout | Kidney dysfunction | Rate | 1990 | 1.821247062 | 2.74586376 | 1.14321942 |
| DALYs (Disability-Adjusted Life Years) | Bahrain | Both | Age-standardized | Gout | Kidney dysfunction | Rate | 1990 | 1.804395011 | 2.66640436 | 1.12578496 |
| YLDs (Years Lived with Disability) | Bahrain | Both | Age-standardized | Gout | Kidney dysfunction | Rate | 1990 | 1.804395011 | 2.66640436 | 1.12578496 |
| DALYs (Disability-Adjusted Life Years) | Egypt | Both | Age-standardized | Gout | Kidney dysfunction | Rate | 1990 | 1.789490835 | 2.64639734 | 1.1088841 |
| YLDs (Years Lived with Disability) | Egypt | Both | Age-standardized | Gout | Kidney dysfunction | Rate | 1990 | 1.789490835 | 2.64639734 | 1.1088841 |
| DALYs (Disability-Adjusted Life Years) | Sao Tome and Principe | Both | Age-standardized | Gout | Kidney dysfunction | Rate | 1990 | 1.784149852 | 2.6900736 | 1.11179163 |
| YLDs (Years Lived with Disability) | Sao Tome and Principe | Both | Age-standardized | Gout | Kidney dysfunction | Rate | 1990 | 1.784149852 | 2.6900736 | 1.11179163 |
| DALYs (Disability-Adjusted Life Years) | Italy | Both | Age-standardized | Gout | Kidney dysfunction | Rate | 1990 | 1.783819803 | 2.67349842 | 1.10008376 |
| YLDs (Years Lived with Disability) | Italy | Both | Age-standardized | Gout | Kidney dysfunction | Rate | 1990 | 1.783819803 | 2.67349842 | 1.10008376 |
| DALYs (Disability-Adjusted Life Years) | Guinea-Bissau | Both | Age-standardized | Gout | Kidney dysfunction | Rate | 1990 | 1.779398046 | 2.70995735 | 1.1056913 |
| YLDs (Years Lived with Disability) | Guinea-Bissau | Both | Age-standardized | Gout | Kidney dysfunction | Rate | 1990 | 1.779398046 | 2.70995735 | 1.1056913 |
| DALYs (Disability-Adjusted Life Years) | Sweden | Both | Age-standardized | Gout | Kidney dysfunction | Rate | 1990 | 1.766320481 | 2.6915147 | 1.07469481 |
| YLDs (Years Lived with Disability) | Sweden | Both | Age-standardized | Gout | Kidney dysfunction | Rate | 1990 | 1.766320481 | 2.6915147 | 1.07469481 |
| DALYs (Disability-Adjusted Life Years) | Cambodia | Both | Age-standardized | Gout | Kidney dysfunction | Rate | 1990 | 1.764698488 | 2.6690979 | 1.10683872 |
| YLDs (Years Lived with Disability) | Cambodia | Both | Age-standardized | Gout | Kidney dysfunction | Rate | 1990 | 1.764698488 | 2.6690979 | 1.10683872 |
| DALYs (Disability-Adjusted Life Years) | Guinea | Both | Age-standardized | Gout | Kidney dysfunction | Rate | 1990 | 1.76411838 | 2.5771771 | 1.1111605 |
| YLDs (Years Lived with Disability) | Guinea | Both | Age-standardized | Gout | Kidney dysfunction | Rate | 1990 | 1.76411838 | 2.5771771 | 1.1111605 |
| DALYs (Disability-Adjusted Life Years) | Benin | Both | Age-standardized | Gout | Kidney dysfunction | Rate | 1990 | 1.754532745 | 2.59595145 | 1.10198081 |
| YLDs (Years Lived with Disability) | Benin | Both | Age-standardized | Gout | Kidney dysfunction | Rate | 1990 | 1.754532745 | 2.59595145 | 1.10198081 |
| DALYs (Disability-Adjusted Life Years) | Gambia | Both | Age-standardized | Gout | Kidney dysfunction | Rate | 1990 | 1.754383689 | 2.64131374 | 1.07220336 |
| YLDs (Years Lived with Disability) | Gambia | Both | Age-standardized | Gout | Kidney dysfunction | Rate | 1990 | 1.754383689 | 2.64131374 | 1.07220336 |
| DALYs (Disability-Adjusted Life Years) | Bhutan | Both | Age-standardized | Gout | Kidney dysfunction | Rate | 1990 | 1.746387109 | 2.634236 | 1.0877193 |
| YLDs (Years Lived with Disability) | Bhutan | Both | Age-standardized | Gout | Kidney dysfunction | Rate | 1990 | 1.746387109 | 2.634236 | 1.0877193 |
| DALYs (Disability-Adjusted Life Years) | Syrian Arab Republic | Both | Age-standardized | Gout | Kidney dysfunction | Rate | 1990 | 1.745808819 | 2.63715577 | 1.07034233 |
| YLDs (Years Lived with Disability) | Syrian Arab Republic | Both | Age-standardized | Gout | Kidney dysfunction | Rate | 1990 | 1.745808819 | 2.63715577 | 1.07034233 |
| DALYs (Disability-Adjusted Life Years) | Libya | Both | Age-standardized | Gout | Kidney dysfunction | Rate | 1990 | 1.726320651 | 2.62287579 | 1.09036298 |
| YLDs (Years Lived with Disability) | Libya | Both | Age-standardized | Gout | Kidney dysfunction | Rate | 1990 | 1.726320651 | 2.62287579 | 1.09036298 |
| DALYs (Disability-Adjusted Life Years) | Sierra Leone | Both | Age-standardized | Gout | Kidney dysfunction | Rate | 1990 | 1.720289615 | 2.57591596 | 1.0598231 |
| YLDs (Years Lived with Disability) | Sierra Leone | Both | Age-standardized | Gout | Kidney dysfunction | Rate | 1990 | 1.720289615 | 2.57591596 | 1.0598231 |
| DALYs (Disability-Adjusted Life Years) | Mali | Both | Age-standardized | Gout | Kidney dysfunction | Rate | 1990 | 1.713044489 | 2.5804561 | 1.06858908 |
| YLDs (Years Lived with Disability) | Mali | Both | Age-standardized | Gout | Kidney dysfunction | Rate | 1990 | 1.713044489 | 2.5804561 | 1.06858908 |
| DALYs (Disability-Adjusted Life Years) | Niger | Both | Age-standardized | Gout | Kidney dysfunction | Rate | 1990 | 1.70638424 | 2.55343406 | 1.06446855 |
| YLDs (Years Lived with Disability) | Niger | Both | Age-standardized | Gout | Kidney dysfunction | Rate | 1990 | 1.70638424 | 2.55343406 | 1.06446855 |
| DALYs (Disability-Adjusted Life Years) | Togo | Both | Age-standardized | Gout | Kidney dysfunction | Rate | 1990 | 1.682866829 | 2.55012049 | 1.04589638 |
| YLDs (Years Lived with Disability) | Togo | Both | Age-standardized | Gout | Kidney dysfunction | Rate | 1990 | 1.682866829 | 2.55012049 | 1.04589638 |
| DALYs (Disability-Adjusted Life Years) | India | Both | Age-standardized | Gout | Kidney dysfunction | Rate | 1990 | 1.67365596 | 2.4778993 | 1.05136887 |
| YLDs (Years Lived with Disability) | India | Both | Age-standardized | Gout | Kidney dysfunction | Rate | 1990 | 1.67365596 | 2.4778993 | 1.05136887 |
| DALYs (Disability-Adjusted Life Years) | Turkey | Both | Age-standardized | Gout | Kidney dysfunction | Rate | 1990 | 1.672284863 | 2.52496851 | 1.02065392 |
| YLDs (Years Lived with Disability) | Turkey | Both | Age-standardized | Gout | Kidney dysfunction | Rate | 1990 | 1.672284863 | 2.52496851 | 1.02065392 |
| DALYs (Disability-Adjusted Life Years) | Portugal | Both | Age-standardized | Gout | Kidney dysfunction | Rate | 1990 | 1.642377732 | 2.54412004 | 1.01169785 |
| YLDs (Years Lived with Disability) | Portugal | Both | Age-standardized | Gout | Kidney dysfunction | Rate | 1990 | 1.642377732 | 2.54412004 | 1.01169785 |
| DALYs (Disability-Adjusted Life Years) | Algeria | Both | Age-standardized | Gout | Kidney dysfunction | Rate | 1990 | 1.641001746 | 2.45707344 | 1.01964578 |
| YLDs (Years Lived with Disability) | Algeria | Both | Age-standardized | Gout | Kidney dysfunction | Rate | 1990 | 1.641001746 | 2.45707344 | 1.01964578 |
| DALYs (Disability-Adjusted Life Years) | Palestine | Both | Age-standardized | Gout | Kidney dysfunction | Rate | 1990 | 1.636416179 | 2.47618553 | 1.02362419 |
| YLDs (Years Lived with Disability) | Palestine | Both | Age-standardized | Gout | Kidney dysfunction | Rate | 1990 | 1.636416179 | 2.47618553 | 1.02362419 |
| DALYs (Disability-Adjusted Life Years) | Chad | Both | Age-standardized | Gout | Kidney dysfunction | Rate | 1990 | 1.625109541 | 2.42230846 | 1.00765001 |
| YLDs (Years Lived with Disability) | Chad | Both | Age-standardized | Gout | Kidney dysfunction | Rate | 1990 | 1.625109541 | 2.42230846 | 1.00765001 |
| DALYs (Disability-Adjusted Life Years) | Finland | Both | Age-standardized | Gout | Kidney dysfunction | Rate | 1990 | 1.617956628 | 2.50976124 | 0.99146924 |
| YLDs (Years Lived with Disability) | Finland | Both | Age-standardized | Gout | Kidney dysfunction | Rate | 1990 | 1.617956628 | 2.50976124 | 0.99146924 |
| DALYs (Disability-Adjusted Life Years) | Burkina Faso | Both | Age-standardized | Gout | Kidney dysfunction | Rate | 1990 | 1.613839557 | 2.37650506 | 1.01478299 |
| YLDs (Years Lived with Disability) | Burkina Faso | Both | Age-standardized | Gout | Kidney dysfunction | Rate | 1990 | 1.613839557 | 2.37650506 | 1.01478299 |
| DALYs (Disability-Adjusted Life Years) | France | Both | Age-standardized | Gout | Kidney dysfunction | Rate | 1990 | 1.59759804 | 2.42341708 | 0.98676825 |
| YLDs (Years Lived with Disability) | France | Both | Age-standardized | Gout | Kidney dysfunction | Rate | 1990 | 1.59759804 | 2.42341708 | 0.98676825 |
| DALYs (Disability-Adjusted Life Years) | Lithuania | Both | Age-standardized | Gout | Kidney dysfunction | Rate | 1990 | 1.589351781 | 2.36692788 | 0.98686855 |
| YLDs (Years Lived with Disability) | Lithuania | Both | Age-standardized | Gout | Kidney dysfunction | Rate | 1990 | 1.589351781 | 2.36692788 | 0.98686855 |
| DALYs (Disability-Adjusted Life Years) | Afghanistan | Both | Age-standardized | Gout | Kidney dysfunction | Rate | 1990 | 1.573125315 | 2.34568692 | 0.95567917 |
| YLDs (Years Lived with Disability) | Afghanistan | Both | Age-standardized | Gout | Kidney dysfunction | Rate | 1990 | 1.573125315 | 2.34568692 | 0.95567917 |
| DALYs (Disability-Adjusted Life Years) | Tunisia | Both | Age-standardized | Gout | Kidney dysfunction | Rate | 1990 | 1.571681153 | 2.34367823 | 0.9665645 |
| YLDs (Years Lived with Disability) | Tunisia | Both | Age-standardized | Gout | Kidney dysfunction | Rate | 1990 | 1.571681153 | 2.34367823 | 0.9665645 |
| DALYs (Disability-Adjusted Life Years) | Ukraine | Both | Age-standardized | Gout | Kidney dysfunction | Rate | 1990 | 1.567012307 | 2.31607154 | 0.97584242 |
| YLDs (Years Lived with Disability) | Ukraine | Both | Age-standardized | Gout | Kidney dysfunction | Rate | 1990 | 1.567012307 | 2.31607154 | 0.97584242 |
| DALYs (Disability-Adjusted Life Years) | Cabo Verde | Both | Age-standardized | Gout | Kidney dysfunction | Rate | 1990 | 1.560707564 | 2.34735706 | 0.97391715 |
| YLDs (Years Lived with Disability) | Cabo Verde | Both | Age-standardized | Gout | Kidney dysfunction | Rate | 1990 | 1.560707564 | 2.34735706 | 0.97391715 |
| DALYs (Disability-Adjusted Life Years) | Lebanon | Both | Age-standardized | Gout | Kidney dysfunction | Rate | 1990 | 1.557951568 | 2.34365245 | 0.99004197 |
| YLDs (Years Lived with Disability) | Lebanon | Both | Age-standardized | Gout | Kidney dysfunction | Rate | 1990 | 1.557951568 | 2.34365245 | 0.99004197 |
| DALYs (Disability-Adjusted Life Years) | Estonia | Both | Age-standardized | Gout | Kidney dysfunction | Rate | 1990 | 1.55177062 | 2.30160091 | 0.94628651 |
| YLDs (Years Lived with Disability) | Estonia | Both | Age-standardized | Gout | Kidney dysfunction | Rate | 1990 | 1.55177062 | 2.30160091 | 0.94628651 |
| DALYs (Disability-Adjusted Life Years) | North Macedonia | Both | Age-standardized | Gout | Kidney dysfunction | Rate | 1990 | 1.528579573 | 2.29221607 | 0.94836188 |
| YLDs (Years Lived with Disability) | North Macedonia | Both | Age-standardized | Gout | Kidney dysfunction | Rate | 1990 | 1.528579573 | 2.29221607 | 0.94836188 |
| DALYs (Disability-Adjusted Life Years) | Bangladesh | Both | Age-standardized | Gout | Kidney dysfunction | Rate | 1990 | 1.528459864 | 2.2822574 | 0.93642497 |
| YLDs (Years Lived with Disability) | Bangladesh | Both | Age-standardized | Gout | Kidney dysfunction | Rate | 1990 | 1.528459864 | 2.2822574 | 0.93642497 |
| DALYs (Disability-Adjusted Life Years) | Latvia | Both | Age-standardized | Gout | Kidney dysfunction | Rate | 1990 | 1.526102621 | 2.309933 | 0.95124585 |
| YLDs (Years Lived with Disability) | Latvia | Both | Age-standardized | Gout | Kidney dysfunction | Rate | 1990 | 1.526102621 | 2.309933 | 0.95124585 |
| DALYs (Disability-Adjusted Life Years) | Pakistan | Both | Age-standardized | Gout | Kidney dysfunction | Rate | 1990 | 1.497959687 | 2.21650311 | 0.94434548 |
| YLDs (Years Lived with Disability) | Pakistan | Both | Age-standardized | Gout | Kidney dysfunction | Rate | 1990 | 1.497959687 | 2.21650311 | 0.94434548 |
| DALYs (Disability-Adjusted Life Years) | Senegal | Both | Age-standardized | Gout | Kidney dysfunction | Rate | 1990 | 1.49030297 | 2.22758943 | 0.92260263 |
| YLDs (Years Lived with Disability) | Senegal | Both | Age-standardized | Gout | Kidney dysfunction | Rate | 1990 | 1.49030297 | 2.22758943 | 0.92260263 |
| DALYs (Disability-Adjusted Life Years) | Belarus | Both | Age-standardized | Gout | Kidney dysfunction | Rate | 1990 | 1.485313985 | 2.2210846 | 0.91855769 |
| YLDs (Years Lived with Disability) | Belarus | Both | Age-standardized | Gout | Kidney dysfunction | Rate | 1990 | 1.485313985 | 2.2210846 | 0.91855769 |
| DALYs (Disability-Adjusted Life Years) | Sudan | Both | Age-standardized | Gout | Kidney dysfunction | Rate | 1990 | 1.476409404 | 2.24588539 | 0.92250898 |
| YLDs (Years Lived with Disability) | Sudan | Both | Age-standardized | Gout | Kidney dysfunction | Rate | 1990 | 1.476409404 | 2.24588539 | 0.92250898 |
| DALYs (Disability-Adjusted Life Years) | Norway | Both | Age-standardized | Gout | Kidney dysfunction | Rate | 1990 | 1.446391752 | 2.17933629 | 0.88840105 |
| YLDs (Years Lived with Disability) | Norway | Both | Age-standardized | Gout | Kidney dysfunction | Rate | 1990 | 1.446391752 | 2.17933629 | 0.88840105 |
| DALYs (Disability-Adjusted Life Years) | Oman | Both | Age-standardized | Gout | Kidney dysfunction | Rate | 1990 | 1.425162515 | 2.15107955 | 0.8742583 |
| YLDs (Years Lived with Disability) | Oman | Both | Age-standardized | Gout | Kidney dysfunction | Rate | 1990 | 1.425162515 | 2.15107955 | 0.8742583 |
| DALYs (Disability-Adjusted Life Years) | Montenegro | Both | Age-standardized | Gout | Kidney dysfunction | Rate | 1990 | 1.35491074 | 2.03167212 | 0.83716587 |
| YLDs (Years Lived with Disability) | Montenegro | Both | Age-standardized | Gout | Kidney dysfunction | Rate | 1990 | 1.35491074 | 2.03167212 | 0.83716587 |
| DALYs (Disability-Adjusted Life Years) | Russian Federation | Both | Age-standardized | Gout | Kidney dysfunction | Rate | 1990 | 1.318425859 | 1.96782267 | 0.82109063 |
| YLDs (Years Lived with Disability) | Russian Federation | Both | Age-standardized | Gout | Kidney dysfunction | Rate | 1990 | 1.318425859 | 1.96782267 | 0.82109063 |
| DALYs (Disability-Adjusted Life Years) | Romania | Both | Age-standardized | Gout | Kidney dysfunction | Rate | 1990 | 1.316381769 | 1.96197121 | 0.80639467 |
| YLDs (Years Lived with Disability) | Romania | Both | Age-standardized | Gout | Kidney dysfunction | Rate | 1990 | 1.316381769 | 1.96197121 | 0.80639467 |
| DALYs (Disability-Adjusted Life Years) | Bulgaria | Both | Age-standardized | Gout | Kidney dysfunction | Rate | 1990 | 1.300693261 | 1.9209805 | 0.7866092 |
| YLDs (Years Lived with Disability) | Bulgaria | Both | Age-standardized | Gout | Kidney dysfunction | Rate | 1990 | 1.300693261 | 1.9209805 | 0.7866092 |
| DALYs (Disability-Adjusted Life Years) | Yemen | Both | Age-standardized | Gout | Kidney dysfunction | Rate | 1990 | 1.294704183 | 1.95439912 | 0.7885035 |
| YLDs (Years Lived with Disability) | Yemen | Both | Age-standardized | Gout | Kidney dysfunction | Rate | 1990 | 1.294704183 | 1.95439912 | 0.7885035 |
| DALYs (Disability-Adjusted Life Years) | Poland | Both | Age-standardized | Gout | Kidney dysfunction | Rate | 1990 | 1.26764141 | 1.90861489 | 0.79443114 |
| YLDs (Years Lived with Disability) | Poland | Both | Age-standardized | Gout | Kidney dysfunction | Rate | 1990 | 1.26764141 | 1.90861489 | 0.79443114 |
| DALYs (Disability-Adjusted Life Years) | Viet Nam | Both | Age-standardized | Gout | Kidney dysfunction | Rate | 1990 | 1.24340541 | 1.82449658 | 0.75624283 |
| YLDs (Years Lived with Disability) | Viet Nam | Both | Age-standardized | Gout | Kidney dysfunction | Rate | 1990 | 1.24340541 | 1.82449658 | 0.75624283 |
| DALYs (Disability-Adjusted Life Years) | Morocco | Both | Age-standardized | Gout | Kidney dysfunction | Rate | 1990 | 1.224882389 | 1.83599815 | 0.74384496 |
| YLDs (Years Lived with Disability) | Morocco | Both | Age-standardized | Gout | Kidney dysfunction | Rate | 1990 | 1.224882389 | 1.83599815 | 0.74384496 |
| DALYs (Disability-Adjusted Life Years) | Slovakia | Both | Age-standardized | Gout | Kidney dysfunction | Rate | 1990 | 1.200450765 | 1.81855971 | 0.75139025 |
| YLDs (Years Lived with Disability) | Slovakia | Both | Age-standardized | Gout | Kidney dysfunction | Rate | 1990 | 1.200450765 | 1.81855971 | 0.75139025 |
| DALYs (Disability-Adjusted Life Years) | Albania | Both | Age-standardized | Gout | Kidney dysfunction | Rate | 1990 | 1.176179388 | 1.77274089 | 0.7351236 |
| YLDs (Years Lived with Disability) | Albania | Both | Age-standardized | Gout | Kidney dysfunction | Rate | 1990 | 1.176179388 | 1.77274089 | 0.7351236 |
| DALYs (Disability-Adjusted Life Years) | Hungary | Both | Age-standardized | Gout | Kidney dysfunction | Rate | 1990 | 1.154768232 | 1.70219835 | 0.70981711 |
| YLDs (Years Lived with Disability) | Hungary | Both | Age-standardized | Gout | Kidney dysfunction | Rate | 1990 | 1.154768232 | 1.70219835 | 0.70981711 |
| DALYs (Disability-Adjusted Life Years) | Bosnia and Herzegovina | Both | Age-standardized | Gout | Kidney dysfunction | Rate | 1990 | 1.127603494 | 1.66839391 | 0.69370026 |
| YLDs (Years Lived with Disability) | Bosnia and Herzegovina | Both | Age-standardized | Gout | Kidney dysfunction | Rate | 1990 | 1.127603494 | 1.66839391 | 0.69370026 |
| DALYs (Disability-Adjusted Life Years) | Croatia | Both | Age-standardized | Gout | Kidney dysfunction | Rate | 1990 | 1.126145654 | 1.68958133 | 0.70819349 |
| YLDs (Years Lived with Disability) | Croatia | Both | Age-standardized | Gout | Kidney dysfunction | Rate | 1990 | 1.126145654 | 1.68958133 | 0.70819349 |
| DALYs (Disability-Adjusted Life Years) | Czechia | Both | Age-standardized | Gout | Kidney dysfunction | Rate | 1990 | 1.126102551 | 1.68090833 | 0.69026527 |
| YLDs (Years Lived with Disability) | Czechia | Both | Age-standardized | Gout | Kidney dysfunction | Rate | 1990 | 1.126102551 | 1.68090833 | 0.69026527 |
| DALYs (Disability-Adjusted Life Years) | Slovenia | Both | Age-standardized | Gout | Kidney dysfunction | Rate | 1990 | 1.092738943 | 1.66252268 | 0.66792693 |
| YLDs (Years Lived with Disability) | Slovenia | Both | Age-standardized | Gout | Kidney dysfunction | Rate | 1990 | 1.092738943 | 1.66252268 | 0.66792693 |
| DALYs (Disability-Adjusted Life Years) | Zambia | Both | Age-standardized | Gout | Kidney dysfunction | Rate | 1990 | 1.050443969 | 1.58946417 | 0.64376651 |
| YLDs (Years Lived with Disability) | Zambia | Both | Age-standardized | Gout | Kidney dysfunction | Rate | 1990 | 1.050443969 | 1.58946417 | 0.64376651 |
| DALYs (Disability-Adjusted Life Years) | Ghana | Both | Age-standardized | Gout | Kidney dysfunction | Rate | 1990 | 1.044784681 | 1.59018351 | 0.6396217 |
| YLDs (Years Lived with Disability) | Ghana | Both | Age-standardized | Gout | Kidney dysfunction | Rate | 1990 | 1.044784681 | 1.59018351 | 0.6396217 |
| DALYs (Disability-Adjusted Life Years) | South Sudan | Both | Age-standardized | Gout | Kidney dysfunction | Rate | 1990 | 1.031108271 | 1.54410895 | 0.63067201 |
| YLDs (Years Lived with Disability) | South Sudan | Both | Age-standardized | Gout | Kidney dysfunction | Rate | 1990 | 1.031108271 | 1.54410895 | 0.63067201 |
| DALYs (Disability-Adjusted Life Years) | Ethiopia | Both | Age-standardized | Gout | Kidney dysfunction | Rate | 1990 | 1.025059288 | 1.5374655 | 0.63674852 |
| YLDs (Years Lived with Disability) | Ethiopia | Both | Age-standardized | Gout | Kidney dysfunction | Rate | 1990 | 1.025059288 | 1.5374655 | 0.63674852 |
| DALYs (Disability-Adjusted Life Years) | Uganda | Both | Age-standardized | Gout | Kidney dysfunction | Rate | 1990 | 1.012015093 | 1.56787537 | 0.62508125 |
| YLDs (Years Lived with Disability) | Uganda | Both | Age-standardized | Gout | Kidney dysfunction | Rate | 1990 | 1.012015093 | 1.56787537 | 0.62508125 |
| DALYs (Disability-Adjusted Life Years) | Kenya | Both | Age-standardized | Gout | Kidney dysfunction | Rate | 1990 | 0.994520782 | 1.49385028 | 0.60032886 |
| YLDs (Years Lived with Disability) | Kenya | Both | Age-standardized | Gout | Kidney dysfunction | Rate | 1990 | 0.994520782 | 1.49385028 | 0.60032886 |
| DALYs (Disability-Adjusted Life Years) | Serbia | Both | Age-standardized | Gout | Kidney dysfunction | Rate | 1990 | 0.993644541 | 1.48088113 | 0.60580871 |
| YLDs (Years Lived with Disability) | Serbia | Both | Age-standardized | Gout | Kidney dysfunction | Rate | 1990 | 0.993644541 | 1.48088113 | 0.60580871 |
| DALYs (Disability-Adjusted Life Years) | Comoros | Both | Age-standardized | Gout | Kidney dysfunction | Rate | 1990 | 0.980123064 | 1.5116954 | 0.57902643 |
| YLDs (Years Lived with Disability) | Comoros | Both | Age-standardized | Gout | Kidney dysfunction | Rate | 1990 | 0.980123064 | 1.5116954 | 0.57902643 |
| DALYs (Disability-Adjusted Life Years) | Madagascar | Both | Age-standardized | Gout | Kidney dysfunction | Rate | 1990 | 0.948303563 | 1.42706212 | 0.57860724 |
| YLDs (Years Lived with Disability) | Madagascar | Both | Age-standardized | Gout | Kidney dysfunction | Rate | 1990 | 0.948303563 | 1.42706212 | 0.57860724 |
| DALYs (Disability-Adjusted Life Years) | Malawi | Both | Age-standardized | Gout | Kidney dysfunction | Rate | 1990 | 0.94077047 | 1.4101083 | 0.58292509 |
| YLDs (Years Lived with Disability) | Malawi | Both | Age-standardized | Gout | Kidney dysfunction | Rate | 1990 | 0.94077047 | 1.4101083 | 0.58292509 |
| DALYs (Disability-Adjusted Life Years) | Somalia | Both | Age-standardized | Gout | Kidney dysfunction | Rate | 1990 | 0.940628317 | 1.39154661 | 0.58540595 |
| YLDs (Years Lived with Disability) | Somalia | Both | Age-standardized | Gout | Kidney dysfunction | Rate | 1990 | 0.940628317 | 1.39154661 | 0.58540595 |
| DALYs (Disability-Adjusted Life Years) | Rwanda | Both | Age-standardized | Gout | Kidney dysfunction | Rate | 1990 | 0.939426314 | 1.40713382 | 0.57963005 |
| YLDs (Years Lived with Disability) | Rwanda | Both | Age-standardized | Gout | Kidney dysfunction | Rate | 1990 | 0.939426314 | 1.40713382 | 0.57963005 |
| DALYs (Disability-Adjusted Life Years) | Burundi | Both | Age-standardized | Gout | Kidney dysfunction | Rate | 1990 | 0.933963864 | 1.41729178 | 0.57829159 |
| YLDs (Years Lived with Disability) | Burundi | Both | Age-standardized | Gout | Kidney dysfunction | Rate | 1990 | 0.933963864 | 1.41729178 | 0.57829159 |
| DALYs (Disability-Adjusted Life Years) | Djibouti | Both | Age-standardized | Gout | Kidney dysfunction | Rate | 1990 | 0.914625481 | 1.39053435 | 0.55476426 |
| YLDs (Years Lived with Disability) | Djibouti | Both | Age-standardized | Gout | Kidney dysfunction | Rate | 1990 | 0.914625481 | 1.39053435 | 0.55476426 |
| DALYs (Disability-Adjusted Life Years) | Mozambique | Both | Age-standardized | Gout | Kidney dysfunction | Rate | 1990 | 0.889535313 | 1.32698326 | 0.54047954 |
| YLDs (Years Lived with Disability) | Mozambique | Both | Age-standardized | Gout | Kidney dysfunction | Rate | 1990 | 0.889535313 | 1.32698326 | 0.54047954 |
| DALYs (Disability-Adjusted Life Years) | United Republic of Tanzania | Both | Age-standardized | Gout | Kidney dysfunction | Rate | 1990 | 0.882679699 | 1.35696389 | 0.53614433 |
| YLDs (Years Lived with Disability) | United Republic of Tanzania | Both | Age-standardized | Gout | Kidney dysfunction | Rate | 1990 | 0.882679699 | 1.35696389 | 0.53614433 |
| DALYs (Disability-Adjusted Life Years) | Brazil | Both | Age-standardized | Gout | Kidney dysfunction | Rate | 1990 | 0.882050356 | 1.31542347 | 0.55784616 |
| YLDs (Years Lived with Disability) | Brazil | Both | Age-standardized | Gout | Kidney dysfunction | Rate | 1990 | 0.882050356 | 1.31542347 | 0.55784616 |
| DALYs (Disability-Adjusted Life Years) | Eritrea | Both | Age-standardized | Gout | Kidney dysfunction | Rate | 1990 | 0.839633269 | 1.2767144 | 0.50869208 |
| YLDs (Years Lived with Disability) | Eritrea | Both | Age-standardized | Gout | Kidney dysfunction | Rate | 1990 | 0.839633269 | 1.2767144 | 0.50869208 |
| DALYs (Disability-Adjusted Life Years) | Paraguay | Both | Age-standardized | Gout | Kidney dysfunction | Rate | 1990 | 0.838310376 | 1.22889242 | 0.52285257 |
| YLDs (Years Lived with Disability) | Paraguay | Both | Age-standardized | Gout | Kidney dysfunction | Rate | 1990 | 0.838310376 | 1.22889242 | 0.52285257 |
| DALYs (Disability-Adjusted Life Years) | Costa Rica | Both | Age-standardized | Gout | Kidney dysfunction | Rate | 1990 | 0.752163817 | 1.11848329 | 0.46709551 |
| YLDs (Years Lived with Disability) | Costa Rica | Both | Age-standardized | Gout | Kidney dysfunction | Rate | 1990 | 0.752163817 | 1.11848329 | 0.46709551 |
| DALYs (Disability-Adjusted Life Years) | Puerto Rico | Both | Age-standardized | Gout | Kidney dysfunction | Rate | 1990 | 0.708587486 | 1.07753833 | 0.44026713 |
| YLDs (Years Lived with Disability) | Puerto Rico | Both | Age-standardized | Gout | Kidney dysfunction | Rate | 1990 | 0.708587486 | 1.07753833 | 0.44026713 |
| DALYs (Disability-Adjusted Life Years) | Nicaragua | Both | Age-standardized | Gout | Kidney dysfunction | Rate | 1990 | 0.705243813 | 1.09964786 | 0.43841161 |
| YLDs (Years Lived with Disability) | Nicaragua | Both | Age-standardized | Gout | Kidney dysfunction | Rate | 1990 | 0.705243813 | 1.09964786 | 0.43841161 |
| DALYs (Disability-Adjusted Life Years) | Saint Kitts and Nevis | Both | Age-standardized | Gout | Kidney dysfunction | Rate | 1990 | 0.682599562 | 1.02405926 | 0.4221736 |
| YLDs (Years Lived with Disability) | Saint Kitts and Nevis | Both | Age-standardized | Gout | Kidney dysfunction | Rate | 1990 | 0.682599562 | 1.02405926 | 0.4221736 |
| DALYs (Disability-Adjusted Life Years) | Suriname | Both | Age-standardized | Gout | Kidney dysfunction | Rate | 1990 | 0.673806458 | 1.02786493 | 0.41656521 |
| YLDs (Years Lived with Disability) | Suriname | Both | Age-standardized | Gout | Kidney dysfunction | Rate | 1990 | 0.673806458 | 1.02786493 | 0.41656521 |
| DALYs (Disability-Adjusted Life Years) | United States Virgin Islands | Both | Age-standardized | Gout | Kidney dysfunction | Rate | 1990 | 0.672179373 | 1.01322594 | 0.41554016 |
| YLDs (Years Lived with Disability) | United States Virgin Islands | Both | Age-standardized | Gout | Kidney dysfunction | Rate | 1990 | 0.672179373 | 1.01322594 | 0.41554016 |
| DALYs (Disability-Adjusted Life Years) | Belize | Both | Age-standardized | Gout | Kidney dysfunction | Rate | 1990 | 0.671410236 | 1.00292903 | 0.41256583 |
| YLDs (Years Lived with Disability) | Belize | Both | Age-standardized | Gout | Kidney dysfunction | Rate | 1990 | 0.671410236 | 1.00292903 | 0.41256583 |
| DALYs (Disability-Adjusted Life Years) | Trinidad and Tobago | Both | Age-standardized | Gout | Kidney dysfunction | Rate | 1990 | 0.669728496 | 0.98971797 | 0.41618494 |
| YLDs (Years Lived with Disability) | Trinidad and Tobago | Both | Age-standardized | Gout | Kidney dysfunction | Rate | 1990 | 0.669728496 | 0.98971797 | 0.41618494 |
| DALYs (Disability-Adjusted Life Years) | Ecuador | Both | Age-standardized | Gout | Kidney dysfunction | Rate | 1990 | 0.666454601 | 1.00903471 | 0.40978399 |
| YLDs (Years Lived with Disability) | Ecuador | Both | Age-standardized | Gout | Kidney dysfunction | Rate | 1990 | 0.666454601 | 1.00903471 | 0.40978399 |
| DALYs (Disability-Adjusted Life Years) | Dominica | Both | Age-standardized | Gout | Kidney dysfunction | Rate | 1990 | 0.665988571 | 1.00603791 | 0.41085038 |
| YLDs (Years Lived with Disability) | Dominica | Both | Age-standardized | Gout | Kidney dysfunction | Rate | 1990 | 0.665988571 | 1.00603791 | 0.41085038 |
| DALYs (Disability-Adjusted Life Years) | Bahamas | Both | Age-standardized | Gout | Kidney dysfunction | Rate | 1990 | 0.657967839 | 0.99985068 | 0.41419489 |
| YLDs (Years Lived with Disability) | Bahamas | Both | Age-standardized | Gout | Kidney dysfunction | Rate | 1990 | 0.657967839 | 0.99985068 | 0.41419489 |
| DALYs (Disability-Adjusted Life Years) | Jamaica | Both | Age-standardized | Gout | Kidney dysfunction | Rate | 1990 | 0.657676767 | 0.98048055 | 0.39453195 |
| YLDs (Years Lived with Disability) | Jamaica | Both | Age-standardized | Gout | Kidney dysfunction | Rate | 1990 | 0.657676767 | 0.98048055 | 0.39453195 |
| DALYs (Disability-Adjusted Life Years) | Mexico | Both | Age-standardized | Gout | Kidney dysfunction | Rate | 1990 | 0.656475144 | 0.99156698 | 0.41333838 |
| YLDs (Years Lived with Disability) | Mexico | Both | Age-standardized | Gout | Kidney dysfunction | Rate | 1990 | 0.656475144 | 0.99156698 | 0.41333838 |
| DALYs (Disability-Adjusted Life Years) | Bolivia (Plurinational State of) | Both | Age-standardized | Gout | Kidney dysfunction | Rate | 1990 | 0.651230965 | 0.9722265 | 0.40896599 |
| YLDs (Years Lived with Disability) | Bolivia (Plurinational State of) | Both | Age-standardized | Gout | Kidney dysfunction | Rate | 1990 | 0.651230965 | 0.9722265 | 0.40896599 |
| DALYs (Disability-Adjusted Life Years) | Guyana | Both | Age-standardized | Gout | Kidney dysfunction | Rate | 1990 | 0.639116476 | 0.95470253 | 0.39608685 |
| YLDs (Years Lived with Disability) | Guyana | Both | Age-standardized | Gout | Kidney dysfunction | Rate | 1990 | 0.639116476 | 0.95470253 | 0.39608685 |
| DALYs (Disability-Adjusted Life Years) | Bermuda | Both | Age-standardized | Gout | Kidney dysfunction | Rate | 1990 | 0.631195849 | 0.95690062 | 0.39045895 |
| YLDs (Years Lived with Disability) | Bermuda | Both | Age-standardized | Gout | Kidney dysfunction | Rate | 1990 | 0.631195849 | 0.95690062 | 0.39045895 |
| DALYs (Disability-Adjusted Life Years) | Barbados | Both | Age-standardized | Gout | Kidney dysfunction | Rate | 1990 | 0.629812461 | 0.95458067 | 0.38921006 |
| YLDs (Years Lived with Disability) | Barbados | Both | Age-standardized | Gout | Kidney dysfunction | Rate | 1990 | 0.629812461 | 0.95458067 | 0.38921006 |
| DALYs (Disability-Adjusted Life Years) | Saint Lucia | Both | Age-standardized | Gout | Kidney dysfunction | Rate | 1990 | 0.628688491 | 0.96334889 | 0.38157861 |
| YLDs (Years Lived with Disability) | Saint Lucia | Both | Age-standardized | Gout | Kidney dysfunction | Rate | 1990 | 0.628688491 | 0.96334889 | 0.38157861 |
| DALYs (Disability-Adjusted Life Years) | Grenada | Both | Age-standardized | Gout | Kidney dysfunction | Rate | 1990 | 0.625140722 | 0.96389684 | 0.39306926 |
| YLDs (Years Lived with Disability) | Grenada | Both | Age-standardized | Gout | Kidney dysfunction | Rate | 1990 | 0.625140722 | 0.96389684 | 0.39306926 |
| DALYs (Disability-Adjusted Life Years) | Antigua and Barbuda | Both | Age-standardized | Gout | Kidney dysfunction | Rate | 1990 | 0.619709073 | 0.94690719 | 0.37815827 |
| YLDs (Years Lived with Disability) | Antigua and Barbuda | Both | Age-standardized | Gout | Kidney dysfunction | Rate | 1990 | 0.619709073 | 0.94690719 | 0.37815827 |
| DALYs (Disability-Adjusted Life Years) | Cuba | Both | Age-standardized | Gout | Kidney dysfunction | Rate | 1990 | 0.601677314 | 0.89418333 | 0.35638506 |
| YLDs (Years Lived with Disability) | Cuba | Both | Age-standardized | Gout | Kidney dysfunction | Rate | 1990 | 0.601677314 | 0.89418333 | 0.35638506 |
| DALYs (Disability-Adjusted Life Years) | Haiti | Both | Age-standardized | Gout | Kidney dysfunction | Rate | 1990 | 0.598926051 | 0.91539004 | 0.37186738 |
| YLDs (Years Lived with Disability) | Haiti | Both | Age-standardized | Gout | Kidney dysfunction | Rate | 1990 | 0.598926051 | 0.91539004 | 0.37186738 |
| DALYs (Disability-Adjusted Life Years) | Dominican Republic | Both | Age-standardized | Gout | Kidney dysfunction | Rate | 1990 | 0.59277558 | 0.92397163 | 0.35021561 |
| YLDs (Years Lived with Disability) | Dominican Republic | Both | Age-standardized | Gout | Kidney dysfunction | Rate | 1990 | 0.59277558 | 0.92397163 | 0.35021561 |
| DALYs (Disability-Adjusted Life Years) | Saint Vincent and the Grenadines | Both | Age-standardized | Gout | Kidney dysfunction | Rate | 1990 | 0.588557542 | 0.8809715 | 0.36629395 |
| YLDs (Years Lived with Disability) | Saint Vincent and the Grenadines | Both | Age-standardized | Gout | Kidney dysfunction | Rate | 1990 | 0.588557542 | 0.8809715 | 0.36629395 |
| DALYs (Disability-Adjusted Life Years) | Venezuela (Bolivarian Republic of) | Both | Age-standardized | Gout | Kidney dysfunction | Rate | 1990 | 0.569664369 | 0.85303258 | 0.35393222 |
| YLDs (Years Lived with Disability) | Venezuela (Bolivarian Republic of) | Both | Age-standardized | Gout | Kidney dysfunction | Rate | 1990 | 0.569664369 | 0.85303258 | 0.35393222 |
| DALYs (Disability-Adjusted Life Years) | Peru | Both | Age-standardized | Gout | Kidney dysfunction | Rate | 1990 | 0.554260318 | 0.83349242 | 0.3480248 |
| YLDs (Years Lived with Disability) | Peru | Both | Age-standardized | Gout | Kidney dysfunction | Rate | 1990 | 0.554260318 | 0.83349242 | 0.3480248 |
| DALYs (Disability-Adjusted Life Years) | Honduras | Both | Age-standardized | Gout | Kidney dysfunction | Rate | 1990 | 0.549315769 | 0.83151642 | 0.34438379 |
| YLDs (Years Lived with Disability) | Honduras | Both | Age-standardized | Gout | Kidney dysfunction | Rate | 1990 | 0.549315769 | 0.83151642 | 0.34438379 |
| DALYs (Disability-Adjusted Life Years) | Guatemala | Both | Age-standardized | Gout | Kidney dysfunction | Rate | 1990 | 0.537228348 | 0.84174905 | 0.34428653 |
| YLDs (Years Lived with Disability) | Guatemala | Both | Age-standardized | Gout | Kidney dysfunction | Rate | 1990 | 0.537228348 | 0.84174905 | 0.34428653 |
| DALYs (Disability-Adjusted Life Years) | Colombia | Both | Age-standardized | Gout | Kidney dysfunction | Rate | 1990 | 0.527702858 | 0.79902671 | 0.32690138 |
| YLDs (Years Lived with Disability) | Colombia | Both | Age-standardized | Gout | Kidney dysfunction | Rate | 1990 | 0.527702858 | 0.79902671 | 0.32690138 |
| DALYs (Disability-Adjusted Life Years) | Panama | Both | Age-standardized | Gout | Kidney dysfunction | Rate | 1990 | 0.522650149 | 0.78709902 | 0.32133009 |
| YLDs (Years Lived with Disability) | Panama | Both | Age-standardized | Gout | Kidney dysfunction | Rate | 1990 | 0.522650149 | 0.78709902 | 0.32133009 |
| DALYs (Disability-Adjusted Life Years) | El Salvador | Both | Age-standardized | Gout | Kidney dysfunction | Rate | 1990 | 0.4767337 | 0.72580078 | 0.28781894 |
| YLDs (Years Lived with Disability) | El Salvador | Both | Age-standardized | Gout | Kidney dysfunction | Rate | 1990 | 0.4767337 | 0.72580078 | 0.28781894 |

**Appendix 5：In 2021, the DALYs and YLDs associated with gout due to renal dysfunction across 204 countries**

| **measure** | **location** | **sex** | **age** | **cause** | **rei** | **metric** | **year** | **val** | **upper** | **lower** |
| --- | --- | --- | --- | --- | --- | --- | --- | --- | --- | --- |
| **DALYs (Disability-Adjusted Life Years)** | **United States of America** | **Both** | **All ages** | **Gout** | **Kidney dysfunction** | **Number** | **2021** | **44353.56347** | **64911.95** | **28907.7087** |
| **YLDs (Years Lived with Disability)** | **United States of America** | **Both** | **All ages** | **Gout** | **Kidney dysfunction** | **Number** | **2021** | **44353.56347** | **64911.95** | **28907.7087** |
| **DALYs (Disability-Adjusted Life Years)** | **China** | **Both** | **All ages** | **Gout** | **Kidney dysfunction** | **Number** | **2021** | **40089.39588** | **60711.1646** | **24449.101** |
| **YLDs (Years Lived with Disability)** | **China** | **Both** | **All ages** | **Gout** | **Kidney dysfunction** | **Number** | **2021** | **40089.39588** | **60711.1646** | **24449.101** |
| **DALYs (Disability-Adjusted Life Years)** | **India** | **Both** | **All ages** | **Gout** | **Kidney dysfunction** | **Number** | **2021** | **16364.14097** | **24635.3214** | **10265.8886** |
| **YLDs (Years Lived with Disability)** | **India** | **Both** | **All ages** | **Gout** | **Kidney dysfunction** | **Number** | **2021** | **16364.14097** | **24635.3214** | **10265.8886** |
| **DALYs (Disability-Adjusted Life Years)** | **Japan** | **Both** | **All ages** | **Gout** | **Kidney dysfunction** | **Number** | **2021** | **12615.75965** | **18799.2537** | **7772.89902** |
| **YLDs (Years Lived with Disability)** | **Japan** | **Both** | **All ages** | **Gout** | **Kidney dysfunction** | **Number** | **2021** | **12615.75965** | **18799.2537** | **7772.89902** |
| **DALYs (Disability-Adjusted Life Years)** | **Germany** | **Both** | **All ages** | **Gout** | **Kidney dysfunction** | **Number** | **2021** | **5362.089164** | **8082.96656** | **3217.17617** |
| **YLDs (Years Lived with Disability)** | **Germany** | **Both** | **All ages** | **Gout** | **Kidney dysfunction** | **Number** | **2021** | **5362.089164** | **8082.96656** | **3217.17617** |
| **DALYs (Disability-Adjusted Life Years)** | **Indonesia** | **Both** | **All ages** | **Gout** | **Kidney dysfunction** | **Number** | **2021** | **4906.926751** | **7434.09375** | **3083.41226** |
| **YLDs (Years Lived with Disability)** | **Indonesia** | **Both** | **All ages** | **Gout** | **Kidney dysfunction** | **Number** | **2021** | **4906.926751** | **7434.09375** | **3083.41226** |
| **DALYs (Disability-Adjusted Life Years)** | **United Kingdom** | **Both** | **All ages** | **Gout** | **Kidney dysfunction** | **Number** | **2021** | **3720.688864** | **5560.47036** | **2293.41479** |
| **YLDs (Years Lived with Disability)** | **United Kingdom** | **Both** | **All ages** | **Gout** | **Kidney dysfunction** | **Number** | **2021** | **3720.688864** | **5560.47036** | **2293.41479** |
| **DALYs (Disability-Adjusted Life Years)** | **Russian Federation** | **Both** | **All ages** | **Gout** | **Kidney dysfunction** | **Number** | **2021** | **3656.692213** | **5444.56915** | **2295.74401** |
| **YLDs (Years Lived with Disability)** | **Russian Federation** | **Both** | **All ages** | **Gout** | **Kidney dysfunction** | **Number** | **2021** | **3656.692213** | **5444.56915** | **2295.74401** |
| **DALYs (Disability-Adjusted Life Years)** | **Canada** | **Both** | **All ages** | **Gout** | **Kidney dysfunction** | **Number** | **2021** | **3402.572997** | **5069.76263** | **2127.30047** |
| **YLDs (Years Lived with Disability)** | **Canada** | **Both** | **All ages** | **Gout** | **Kidney dysfunction** | **Number** | **2021** | **3402.572997** | **5069.76263** | **2127.30047** |
| **DALYs (Disability-Adjusted Life Years)** | **Thailand** | **Both** | **All ages** | **Gout** | **Kidney dysfunction** | **Number** | **2021** | **3370.900451** | **5058.82519** | **2114.77991** |
| **YLDs (Years Lived with Disability)** | **Thailand** | **Both** | **All ages** | **Gout** | **Kidney dysfunction** | **Number** | **2021** | **3370.900451** | **5058.82519** | **2114.77991** |
| **DALYs (Disability-Adjusted Life Years)** | **France** | **Both** | **All ages** | **Gout** | **Kidney dysfunction** | **Number** | **2021** | **3143.056222** | **4866.69332** | **1962.61854** |
| **YLDs (Years Lived with Disability)** | **France** | **Both** | **All ages** | **Gout** | **Kidney dysfunction** | **Number** | **2021** | **3143.056222** | **4866.69332** | **1962.61854** |
| **DALYs (Disability-Adjusted Life Years)** | **Italy** | **Both** | **All ages** | **Gout** | **Kidney dysfunction** | **Number** | **2021** | **2743.742295** | **4127.07821** | **1702.57064** |
| **YLDs (Years Lived with Disability)** | **Italy** | **Both** | **All ages** | **Gout** | **Kidney dysfunction** | **Number** | **2021** | **2743.742295** | **4127.07821** | **1702.57064** |
| **DALYs (Disability-Adjusted Life Years)** | **Brazil** | **Both** | **All ages** | **Gout** | **Kidney dysfunction** | **Number** | **2021** | **2688.371203** | **4033.40598** | **1718.64387** |
| **YLDs (Years Lived with Disability)** | **Brazil** | **Both** | **All ages** | **Gout** | **Kidney dysfunction** | **Number** | **2021** | **2688.371203** | **4033.40598** | **1718.64387** |
| **DALYs (Disability-Adjusted Life Years)** | **Australia** | **Both** | **All ages** | **Gout** | **Kidney dysfunction** | **Number** | **2021** | **2563.70635** | **3942.86** | **1557.74932** |
| **YLDs (Years Lived with Disability)** | **Australia** | **Both** | **All ages** | **Gout** | **Kidney dysfunction** | **Number** | **2021** | **2563.70635** | **3942.86** | **1557.74932** |
| **DALYs (Disability-Adjusted Life Years)** | **Republic of Korea** | **Both** | **All ages** | **Gout** | **Kidney dysfunction** | **Number** | **2021** | **2389.497713** | **3561.84708** | **1482.74407** |
| **YLDs (Years Lived with Disability)** | **Republic of Korea** | **Both** | **All ages** | **Gout** | **Kidney dysfunction** | **Number** | **2021** | **2389.497713** | **3561.84708** | **1482.74407** |
| **DALYs (Disability-Adjusted Life Years)** | **Spain** | **Both** | **All ages** | **Gout** | **Kidney dysfunction** | **Number** | **2021** | **2343.986085** | **3576.88599** | **1470.32949** |
| **YLDs (Years Lived with Disability)** | **Spain** | **Both** | **All ages** | **Gout** | **Kidney dysfunction** | **Number** | **2021** | **2343.986085** | **3576.88599** | **1470.32949** |
| **DALYs (Disability-Adjusted Life Years)** | **Bangladesh** | **Both** | **All ages** | **Gout** | **Kidney dysfunction** | **Number** | **2021** | **1956.01266** | **2928.86305** | **1220.49283** |
| **YLDs (Years Lived with Disability)** | **Bangladesh** | **Both** | **All ages** | **Gout** | **Kidney dysfunction** | **Number** | **2021** | **1956.01266** | **2928.86305** | **1220.49283** |
| **DALYs (Disability-Adjusted Life Years)** | **Pakistan** | **Both** | **All ages** | **Gout** | **Kidney dysfunction** | **Number** | **2021** | **1933.547129** | **2930.55699** | **1207.72537** |
| **YLDs (Years Lived with Disability)** | **Pakistan** | **Both** | **All ages** | **Gout** | **Kidney dysfunction** | **Number** | **2021** | **1933.547129** | **2930.55699** | **1207.72537** |
| **DALYs (Disability-Adjusted Life Years)** | **Nigeria** | **Both** | **All ages** | **Gout** | **Kidney dysfunction** | **Number** | **2021** | **1913.782937** | **2898.09934** | **1220.71308** |
| **YLDs (Years Lived with Disability)** | **Nigeria** | **Both** | **All ages** | **Gout** | **Kidney dysfunction** | **Number** | **2021** | **1913.782937** | **2898.09934** | **1220.71308** |
| **DALYs (Disability-Adjusted Life Years)** | **Philippines** | **Both** | **All ages** | **Gout** | **Kidney dysfunction** | **Number** | **2021** | **1820.063194** | **2732.3495** | **1146.72177** |
| **YLDs (Years Lived with Disability)** | **Philippines** | **Both** | **All ages** | **Gout** | **Kidney dysfunction** | **Number** | **2021** | **1820.063194** | **2732.3495** | **1146.72177** |
| **DALYs (Disability-Adjusted Life Years)** | **Iran (Islamic Republic of)** | **Both** | **All ages** | **Gout** | **Kidney dysfunction** | **Number** | **2021** | **1684.450488** | **2535.2955** | **1050.96102** |
| **YLDs (Years Lived with Disability)** | **Iran (Islamic Republic of)** | **Both** | **All ages** | **Gout** | **Kidney dysfunction** | **Number** | **2021** | **1684.450488** | **2535.2955** | **1050.96102** |
| **DALYs (Disability-Adjusted Life Years)** | **Turkey** | **Both** | **All ages** | **Gout** | **Kidney dysfunction** | **Number** | **2021** | **1589.692969** | **2420.07335** | **1001.16928** |
| **YLDs (Years Lived with Disability)** | **Turkey** | **Both** | **All ages** | **Gout** | **Kidney dysfunction** | **Number** | **2021** | **1589.692969** | **2420.07335** | **1001.16928** |
| **DALYs (Disability-Adjusted Life Years)** | **Taiwan (Province of China)** | **Both** | **All ages** | **Gout** | **Kidney dysfunction** | **Number** | **2021** | **1529.837375** | **2248.79909** | **983.811644** |
| **YLDs (Years Lived with Disability)** | **Taiwan (Province of China)** | **Both** | **All ages** | **Gout** | **Kidney dysfunction** | **Number** | **2021** | **1529.837375** | **2248.79909** | **983.811644** |
| **DALYs (Disability-Adjusted Life Years)** | **Argentina** | **Both** | **All ages** | **Gout** | **Kidney dysfunction** | **Number** | **2021** | **1377.439987** | **2092.82774** | **855.014224** |
| **YLDs (Years Lived with Disability)** | **Argentina** | **Both** | **All ages** | **Gout** | **Kidney dysfunction** | **Number** | **2021** | **1377.439987** | **2092.82774** | **855.014224** |
| **DALYs (Disability-Adjusted Life Years)** | **Ukraine** | **Both** | **All ages** | **Gout** | **Kidney dysfunction** | **Number** | **2021** | **1376.792359** | **2080.0573** | **854.990448** |
| **YLDs (Years Lived with Disability)** | **Ukraine** | **Both** | **All ages** | **Gout** | **Kidney dysfunction** | **Number** | **2021** | **1376.792359** | **2080.0573** | **854.990448** |
| **DALYs (Disability-Adjusted Life Years)** | **South Africa** | **Both** | **All ages** | **Gout** | **Kidney dysfunction** | **Number** | **2021** | **1256.31766** | **1888.2201** | **794.377217** |
| **YLDs (Years Lived with Disability)** | **South Africa** | **Both** | **All ages** | **Gout** | **Kidney dysfunction** | **Number** | **2021** | **1256.31766** | **1888.2201** | **794.377217** |
| **DALYs (Disability-Adjusted Life Years)** | **Egypt** | **Both** | **All ages** | **Gout** | **Kidney dysfunction** | **Number** | **2021** | **1189.972648** | **1856.26879** | **740.72942** |
| **YLDs (Years Lived with Disability)** | **Egypt** | **Both** | **All ages** | **Gout** | **Kidney dysfunction** | **Number** | **2021** | **1189.972648** | **1856.26879** | **740.72942** |
| **DALYs (Disability-Adjusted Life Years)** | **Viet Nam** | **Both** | **All ages** | **Gout** | **Kidney dysfunction** | **Number** | **2021** | **1144.677125** | **1707.34088** | **712.673739** |
| **YLDs (Years Lived with Disability)** | **Viet Nam** | **Both** | **All ages** | **Gout** | **Kidney dysfunction** | **Number** | **2021** | **1144.677125** | **1707.34088** | **712.673739** |
| **DALYs (Disability-Adjusted Life Years)** | **Mexico** | **Both** | **All ages** | **Gout** | **Kidney dysfunction** | **Number** | **2021** | **996.0510245** | **1502.60144** | **627.375146** |
| **YLDs (Years Lived with Disability)** | **Mexico** | **Both** | **All ages** | **Gout** | **Kidney dysfunction** | **Number** | **2021** | **996.0510245** | **1502.60144** | **627.375146** |
| **DALYs (Disability-Adjusted Life Years)** | **Poland** | **Both** | **All ages** | **Gout** | **Kidney dysfunction** | **Number** | **2021** | **975.6625579** | **1465.41002** | **610.870081** |
| **YLDs (Years Lived with Disability)** | **Poland** | **Both** | **All ages** | **Gout** | **Kidney dysfunction** | **Number** | **2021** | **975.6625579** | **1465.41002** | **610.870081** |
| **DALYs (Disability-Adjusted Life Years)** | **Myanmar** | **Both** | **All ages** | **Gout** | **Kidney dysfunction** | **Number** | **2021** | **918.2307745** | **1369.02869** | **573.502596** |
| **YLDs (Years Lived with Disability)** | **Myanmar** | **Both** | **All ages** | **Gout** | **Kidney dysfunction** | **Number** | **2021** | **918.2307745** | **1369.02869** | **573.502596** |
| **DALYs (Disability-Adjusted Life Years)** | **Netherlands** | **Both** | **All ages** | **Gout** | **Kidney dysfunction** | **Number** | **2021** | **869.4219641** | **1335.92798** | **542.943813** |
| **YLDs (Years Lived with Disability)** | **Netherlands** | **Both** | **All ages** | **Gout** | **Kidney dysfunction** | **Number** | **2021** | **869.4219641** | **1335.92798** | **542.943813** |
| **DALYs (Disability-Adjusted Life Years)** | **Democratic Republic of the Congo** | **Both** | **All ages** | **Gout** | **Kidney dysfunction** | **Number** | **2021** | **789.928845** | **1177.06827** | **501.905971** |
| **YLDs (Years Lived with Disability)** | **Democratic Republic of the Congo** | **Both** | **All ages** | **Gout** | **Kidney dysfunction** | **Number** | **2021** | **789.928845** | **1177.06827** | **501.905971** |
| **DALYs (Disability-Adjusted Life Years)** | **Malaysia** | **Both** | **All ages** | **Gout** | **Kidney dysfunction** | **Number** | **2021** | **772.4318763** | **1163.81347** | **476.321606** |
| **YLDs (Years Lived with Disability)** | **Malaysia** | **Both** | **All ages** | **Gout** | **Kidney dysfunction** | **Number** | **2021** | **772.4318763** | **1163.81347** | **476.321606** |
| **DALYs (Disability-Adjusted Life Years)** | **Democratic People's Republic of Korea** | **Both** | **All ages** | **Gout** | **Kidney dysfunction** | **Number** | **2021** | **732.6700475** | **1105.372** | **448.030409** |
| **YLDs (Years Lived with Disability)** | **Democratic People's Republic of Korea** | **Both** | **All ages** | **Gout** | **Kidney dysfunction** | **Number** | **2021** | **732.6700475** | **1105.372** | **448.030409** |
| **DALYs (Disability-Adjusted Life Years)** | **Chile** | **Both** | **All ages** | **Gout** | **Kidney dysfunction** | **Number** | **2021** | **714.6957617** | **1093.7582** | **442.702748** |
| **YLDs (Years Lived with Disability)** | **Chile** | **Both** | **All ages** | **Gout** | **Kidney dysfunction** | **Number** | **2021** | **714.6957617** | **1093.7582** | **442.702748** |
| **DALYs (Disability-Adjusted Life Years)** | **Greece** | **Both** | **All ages** | **Gout** | **Kidney dysfunction** | **Number** | **2021** | **682.1752884** | **1033.26308** | **423.817704** |
| **YLDs (Years Lived with Disability)** | **Greece** | **Both** | **All ages** | **Gout** | **Kidney dysfunction** | **Number** | **2021** | **682.1752884** | **1033.26308** | **423.817704** |
| **DALYs (Disability-Adjusted Life Years)** | **Uzbekistan** | **Both** | **All ages** | **Gout** | **Kidney dysfunction** | **Number** | **2021** | **631.0906197** | **961.229898** | **396.483599** |
| **YLDs (Years Lived with Disability)** | **Uzbekistan** | **Both** | **All ages** | **Gout** | **Kidney dysfunction** | **Number** | **2021** | **631.0906197** | **961.229898** | **396.483599** |
| **DALYs (Disability-Adjusted Life Years)** | **Algeria** | **Both** | **All ages** | **Gout** | **Kidney dysfunction** | **Number** | **2021** | **601.5501693** | **905.440067** | **369.425413** |
| **YLDs (Years Lived with Disability)** | **Algeria** | **Both** | **All ages** | **Gout** | **Kidney dysfunction** | **Number** | **2021** | **601.5501693** | **905.440067** | **369.425413** |
| **DALYs (Disability-Adjusted Life Years)** | **Romania** | **Both** | **All ages** | **Gout** | **Kidney dysfunction** | **Number** | **2021** | **597.7837794** | **879.449688** | **377.117067** |
| **YLDs (Years Lived with Disability)** | **Romania** | **Both** | **All ages** | **Gout** | **Kidney dysfunction** | **Number** | **2021** | **597.7837794** | **879.449688** | **377.117067** |
| **DALYs (Disability-Adjusted Life Years)** | **Nepal** | **Both** | **All ages** | **Gout** | **Kidney dysfunction** | **Number** | **2021** | **589.7959258** | **881.582498** | **376.92625** |
| **YLDs (Years Lived with Disability)** | **Nepal** | **Both** | **All ages** | **Gout** | **Kidney dysfunction** | **Number** | **2021** | **589.7959258** | **881.582498** | **376.92625** |
| **DALYs (Disability-Adjusted Life Years)** | **Sri Lanka** | **Both** | **All ages** | **Gout** | **Kidney dysfunction** | **Number** | **2021** | **583.7395277** | **882.72647** | **361.986759** |
| **YLDs (Years Lived with Disability)** | **Sri Lanka** | **Both** | **All ages** | **Gout** | **Kidney dysfunction** | **Number** | **2021** | **583.7395277** | **882.72647** | **361.986759** |
| **DALYs (Disability-Adjusted Life Years)** | **Belgium** | **Both** | **All ages** | **Gout** | **Kidney dysfunction** | **Number** | **2021** | **564.7413361** | **845.200931** | **345.660267** |
| **YLDs (Years Lived with Disability)** | **Belgium** | **Both** | **All ages** | **Gout** | **Kidney dysfunction** | **Number** | **2021** | **564.7413361** | **845.200931** | **345.660267** |
| **DALYs (Disability-Adjusted Life Years)** | **New Zealand** | **Both** | **All ages** | **Gout** | **Kidney dysfunction** | **Number** | **2021** | **486.7637335** | **732.063308** | **301.690445** |
| **YLDs (Years Lived with Disability)** | **New Zealand** | **Both** | **All ages** | **Gout** | **Kidney dysfunction** | **Number** | **2021** | **486.7637335** | **732.063308** | **301.690445** |
| **DALYs (Disability-Adjusted Life Years)** | **Sweden** | **Both** | **All ages** | **Gout** | **Kidney dysfunction** | **Number** | **2021** | **468.021059** | **710.779162** | **286.304931** |
| **YLDs (Years Lived with Disability)** | **Sweden** | **Both** | **All ages** | **Gout** | **Kidney dysfunction** | **Number** | **2021** | **468.021059** | **710.779162** | **286.304931** |
| **DALYs (Disability-Adjusted Life Years)** | **Portugal** | **Both** | **All ages** | **Gout** | **Kidney dysfunction** | **Number** | **2021** | **461.0306172** | **703.180517** | **282.338403** |
| **YLDs (Years Lived with Disability)** | **Portugal** | **Both** | **All ages** | **Gout** | **Kidney dysfunction** | **Number** | **2021** | **461.0306172** | **703.180517** | **282.338403** |
| **DALYs (Disability-Adjusted Life Years)** | **Austria** | **Both** | **All ages** | **Gout** | **Kidney dysfunction** | **Number** | **2021** | **450.4088413** | **675.476593** | **278.022138** |
| **YLDs (Years Lived with Disability)** | **Austria** | **Both** | **All ages** | **Gout** | **Kidney dysfunction** | **Number** | **2021** | **450.4088413** | **675.476593** | **278.022138** |
| **DALYs (Disability-Adjusted Life Years)** | **Switzerland** | **Both** | **All ages** | **Gout** | **Kidney dysfunction** | **Number** | **2021** | **448.4797519** | **673.154987** | **283.282685** |
| **YLDs (Years Lived with Disability)** | **Switzerland** | **Both** | **All ages** | **Gout** | **Kidney dysfunction** | **Number** | **2021** | **448.4797519** | **673.154987** | **283.282685** |
| **DALYs (Disability-Adjusted Life Years)** | **Morocco** | **Both** | **All ages** | **Gout** | **Kidney dysfunction** | **Number** | **2021** | **440.3066549** | **646.252891** | **278.511925** |
| **YLDs (Years Lived with Disability)** | **Morocco** | **Both** | **All ages** | **Gout** | **Kidney dysfunction** | **Number** | **2021** | **440.3066549** | **646.252891** | **278.511925** |
| **DALYs (Disability-Adjusted Life Years)** | **Saudi Arabia** | **Both** | **All ages** | **Gout** | **Kidney dysfunction** | **Number** | **2021** | **422.7580482** | **641.570626** | **255.497406** |
| **YLDs (Years Lived with Disability)** | **Saudi Arabia** | **Both** | **All ages** | **Gout** | **Kidney dysfunction** | **Number** | **2021** | **422.7580482** | **641.570626** | **255.497406** |
| **DALYs (Disability-Adjusted Life Years)** | **Kazakhstan** | **Both** | **All ages** | **Gout** | **Kidney dysfunction** | **Number** | **2021** | **408.7078946** | **607.446681** | **254.221965** |
| **YLDs (Years Lived with Disability)** | **Kazakhstan** | **Both** | **All ages** | **Gout** | **Kidney dysfunction** | **Number** | **2021** | **408.7078946** | **607.446681** | **254.221965** |
| **DALYs (Disability-Adjusted Life Years)** | **Iraq** | **Both** | **All ages** | **Gout** | **Kidney dysfunction** | **Number** | **2021** | **407.7031883** | **616.523251** | **258.305386** |
| **YLDs (Years Lived with Disability)** | **Iraq** | **Both** | **All ages** | **Gout** | **Kidney dysfunction** | **Number** | **2021** | **407.7031883** | **616.523251** | **258.305386** |
| **DALYs (Disability-Adjusted Life Years)** | **Ethiopia** | **Both** | **All ages** | **Gout** | **Kidney dysfunction** | **Number** | **2021** | **366.0154133** | **564.122579** | **226.888223** |
| **YLDs (Years Lived with Disability)** | **Ethiopia** | **Both** | **All ages** | **Gout** | **Kidney dysfunction** | **Number** | **2021** | **366.0154133** | **564.122579** | **226.888223** |
| **DALYs (Disability-Adjusted Life Years)** | **Colombia** | **Both** | **All ages** | **Gout** | **Kidney dysfunction** | **Number** | **2021** | **352.4229939** | **537.783986** | **218.80882** |
| **YLDs (Years Lived with Disability)** | **Colombia** | **Both** | **All ages** | **Gout** | **Kidney dysfunction** | **Number** | **2021** | **352.4229939** | **537.783986** | **218.80882** |
| **DALYs (Disability-Adjusted Life Years)** | **Cameroon** | **Both** | **All ages** | **Gout** | **Kidney dysfunction** | **Number** | **2021** | **347.6508465** | **534.038412** | **216.527484** |
| **YLDs (Years Lived with Disability)** | **Cameroon** | **Both** | **All ages** | **Gout** | **Kidney dysfunction** | **Number** | **2021** | **347.6508465** | **534.038412** | **216.527484** |
| **DALYs (Disability-Adjusted Life Years)** | **Israel** | **Both** | **All ages** | **Gout** | **Kidney dysfunction** | **Number** | **2021** | **335.9172876** | **515.04394** | **210.715725** |
| **YLDs (Years Lived with Disability)** | **Israel** | **Both** | **All ages** | **Gout** | **Kidney dysfunction** | **Number** | **2021** | **335.9172876** | **515.04394** | **210.715725** |
| **DALYs (Disability-Adjusted Life Years)** | **Sudan** | **Both** | **All ages** | **Gout** | **Kidney dysfunction** | **Number** | **2021** | **307.3634209** | **470.238763** | **189.054672** |
| **YLDs (Years Lived with Disability)** | **Sudan** | **Both** | **All ages** | **Gout** | **Kidney dysfunction** | **Number** | **2021** | **307.3634209** | **470.238763** | **189.054672** |
| **DALYs (Disability-Adjusted Life Years)** | **Denmark** | **Both** | **All ages** | **Gout** | **Kidney dysfunction** | **Number** | **2021** | **295.6530989** | **459.674414** | **180.807497** |
| **YLDs (Years Lived with Disability)** | **Denmark** | **Both** | **All ages** | **Gout** | **Kidney dysfunction** | **Number** | **2021** | **295.6530989** | **459.674414** | **180.807497** |
| **DALYs (Disability-Adjusted Life Years)** | **Czechia** | **Both** | **All ages** | **Gout** | **Kidney dysfunction** | **Number** | **2021** | **278.3065335** | **416.091124** | **169.147996** |
| **YLDs (Years Lived with Disability)** | **Czechia** | **Both** | **All ages** | **Gout** | **Kidney dysfunction** | **Number** | **2021** | **278.3065335** | **416.091124** | **169.147996** |
| **DALYs (Disability-Adjusted Life Years)** | **Finland** | **Both** | **All ages** | **Gout** | **Kidney dysfunction** | **Number** | **2021** | **270.027139** | **413.311301** | **167.588638** |
| **YLDs (Years Lived with Disability)** | **Finland** | **Both** | **All ages** | **Gout** | **Kidney dysfunction** | **Number** | **2021** | **270.027139** | **413.311301** | **167.588638** |
| **DALYs (Disability-Adjusted Life Years)** | **Singapore** | **Both** | **All ages** | **Gout** | **Kidney dysfunction** | **Number** | **2021** | **266.794825** | **407.770481** | **168.927512** |
| **YLDs (Years Lived with Disability)** | **Singapore** | **Both** | **All ages** | **Gout** | **Kidney dysfunction** | **Number** | **2021** | **266.794825** | **407.770481** | **168.927512** |
| **DALYs (Disability-Adjusted Life Years)** | **Belarus** | **Both** | **All ages** | **Gout** | **Kidney dysfunction** | **Number** | **2021** | **264.1435521** | **391.209603** | **164.263861** |
| **YLDs (Years Lived with Disability)** | **Belarus** | **Both** | **All ages** | **Gout** | **Kidney dysfunction** | **Number** | **2021** | **264.1435521** | **391.209603** | **164.263861** |
| **DALYs (Disability-Adjusted Life Years)** | **Hungary** | **Both** | **All ages** | **Gout** | **Kidney dysfunction** | **Number** | **2021** | **258.9999917** | **385.950786** | **158.490305** |
| **YLDs (Years Lived with Disability)** | **Hungary** | **Both** | **All ages** | **Gout** | **Kidney dysfunction** | **Number** | **2021** | **258.9999917** | **385.950786** | **158.490305** |
| **DALYs (Disability-Adjusted Life Years)** | **Azerbaijan** | **Both** | **All ages** | **Gout** | **Kidney dysfunction** | **Number** | **2021** | **241.6878604** | **363.512466** | **150.147032** |
| **YLDs (Years Lived with Disability)** | **Azerbaijan** | **Both** | **All ages** | **Gout** | **Kidney dysfunction** | **Number** | **2021** | **241.6878604** | **363.512466** | **150.147032** |
| **DALYs (Disability-Adjusted Life Years)** | **Angola** | **Both** | **All ages** | **Gout** | **Kidney dysfunction** | **Number** | **2021** | **236.6026461** | **354.204853** | **149.623792** |
| **YLDs (Years Lived with Disability)** | **Angola** | **Both** | **All ages** | **Gout** | **Kidney dysfunction** | **Number** | **2021** | **236.6026461** | **354.204853** | **149.623792** |
| **DALYs (Disability-Adjusted Life Years)** | **Ireland** | **Both** | **All ages** | **Gout** | **Kidney dysfunction** | **Number** | **2021** | **232.3482004** | **353.422845** | **148.89497** |
| **YLDs (Years Lived with Disability)** | **Ireland** | **Both** | **All ages** | **Gout** | **Kidney dysfunction** | **Number** | **2021** | **232.3482004** | **353.422845** | **148.89497** |
| **DALYs (Disability-Adjusted Life Years)** | **Tunisia** | **Both** | **All ages** | **Gout** | **Kidney dysfunction** | **Number** | **2021** | **217.9448396** | **328.31362** | **133.470513** |
| **YLDs (Years Lived with Disability)** | **Tunisia** | **Both** | **All ages** | **Gout** | **Kidney dysfunction** | **Number** | **2021** | **217.9448396** | **328.31362** | **133.470513** |
| **DALYs (Disability-Adjusted Life Years)** | **Syrian Arab Republic** | **Both** | **All ages** | **Gout** | **Kidney dysfunction** | **Number** | **2021** | **217.5707551** | **325.44959** | **134.876398** |
| **YLDs (Years Lived with Disability)** | **Syrian Arab Republic** | **Both** | **All ages** | **Gout** | **Kidney dysfunction** | **Number** | **2021** | **217.5707551** | **325.44959** | **134.876398** |
| **DALYs (Disability-Adjusted Life Years)** | **Peru** | **Both** | **All ages** | **Gout** | **Kidney dysfunction** | **Number** | **2021** | **215.9859674** | **322.593522** | **131.719927** |
| **YLDs (Years Lived with Disability)** | **Peru** | **Both** | **All ages** | **Gout** | **Kidney dysfunction** | **Number** | **2021** | **215.9859674** | **322.593522** | **131.719927** |
| **DALYs (Disability-Adjusted Life Years)** | **Venezuela (Bolivarian Republic of)** | **Both** | **All ages** | **Gout** | **Kidney dysfunction** | **Number** | **2021** | **208.2526213** | **317.776862** | **132.364015** |
| **YLDs (Years Lived with Disability)** | **Venezuela (Bolivarian Republic of)** | **Both** | **All ages** | **Gout** | **Kidney dysfunction** | **Number** | **2021** | **208.2526213** | **317.776862** | **132.364015** |
| **DALYs (Disability-Adjusted Life Years)** | **Cambodia** | **Both** | **All ages** | **Gout** | **Kidney dysfunction** | **Number** | **2021** | **206.6868785** | **313.515091** | **127.715118** |
| **YLDs (Years Lived with Disability)** | **Cambodia** | **Both** | **All ages** | **Gout** | **Kidney dysfunction** | **Number** | **2021** | **206.6868785** | **313.515091** | **127.715118** |
| **DALYs (Disability-Adjusted Life Years)** | **United Republic of Tanzania** | **Both** | **All ages** | **Gout** | **Kidney dysfunction** | **Number** | **2021** | **204.9304808** | **302.671** | **128.433315** |
| **YLDs (Years Lived with Disability)** | **United Republic of Tanzania** | **Both** | **All ages** | **Gout** | **Kidney dysfunction** | **Number** | **2021** | **204.9304808** | **302.671** | **128.433315** |
| **DALYs (Disability-Adjusted Life Years)** | **Bulgaria** | **Both** | **All ages** | **Gout** | **Kidney dysfunction** | **Number** | **2021** | **198.6936406** | **308.085246** | **121.088144** |
| **YLDs (Years Lived with Disability)** | **Bulgaria** | **Both** | **All ages** | **Gout** | **Kidney dysfunction** | **Number** | **2021** | **198.6936406** | **308.085246** | **121.088144** |
| **DALYs (Disability-Adjusted Life Years)** | **Kenya** | **Both** | **All ages** | **Gout** | **Kidney dysfunction** | **Number** | **2021** | **194.4257738** | **296.682571** | **121.695758** |
| **YLDs (Years Lived with Disability)** | **Kenya** | **Both** | **All ages** | **Gout** | **Kidney dysfunction** | **Number** | **2021** | **194.4257738** | **296.682571** | **121.695758** |
| **DALYs (Disability-Adjusted Life Years)** | **Ivory Coast** | **Both** | **All ages** | **Gout** | **Kidney dysfunction** | **Number** | **2021** | **190.2141254** | **284.243325** | **120.347122** |
| **YLDs (Years Lived with Disability)** | **Ivory Coast** | **Both** | **All ages** | **Gout** | **Kidney dysfunction** | **Number** | **2021** | **190.2141254** | **284.243325** | **120.347122** |
| **DALYs (Disability-Adjusted Life Years)** | **Norway** | **Both** | **All ages** | **Gout** | **Kidney dysfunction** | **Number** | **2021** | **185.7017033** | **276.688224** | **115.689581** |
| **YLDs (Years Lived with Disability)** | **Norway** | **Both** | **All ages** | **Gout** | **Kidney dysfunction** | **Number** | **2021** | **185.7017033** | **276.688224** | **115.689581** |
| **DALYs (Disability-Adjusted Life Years)** | **Serbia** | **Both** | **All ages** | **Gout** | **Kidney dysfunction** | **Number** | **2021** | **182.7578474** | **274.333477** | **109.532449** |
| **YLDs (Years Lived with Disability)** | **Serbia** | **Both** | **All ages** | **Gout** | **Kidney dysfunction** | **Number** | **2021** | **182.7578474** | **274.333477** | **109.532449** |
| **DALYs (Disability-Adjusted Life Years)** | **Yemen** | **Both** | **All ages** | **Gout** | **Kidney dysfunction** | **Number** | **2021** | **179.6896661** | **269.976975** | **110.440817** |
| **YLDs (Years Lived with Disability)** | **Yemen** | **Both** | **All ages** | **Gout** | **Kidney dysfunction** | **Number** | **2021** | **179.6896661** | **269.976975** | **110.440817** |
| **DALYs (Disability-Adjusted Life Years)** | **Uruguay** | **Both** | **All ages** | **Gout** | **Kidney dysfunction** | **Number** | **2021** | **164.8139476** | **247.513601** | **101.258622** |
| **YLDs (Years Lived with Disability)** | **Uruguay** | **Both** | **All ages** | **Gout** | **Kidney dysfunction** | **Number** | **2021** | **164.8139476** | **247.513601** | **101.258622** |
| **DALYs (Disability-Adjusted Life Years)** | **Ghana** | **Both** | **All ages** | **Gout** | **Kidney dysfunction** | **Number** | **2021** | **162.8771524** | **247.030454** | **101.429605** |
| **YLDs (Years Lived with Disability)** | **Ghana** | **Both** | **All ages** | **Gout** | **Kidney dysfunction** | **Number** | **2021** | **162.8771524** | **247.030454** | **101.429605** |
| **DALYs (Disability-Adjusted Life Years)** | **Georgia** | **Both** | **All ages** | **Gout** | **Kidney dysfunction** | **Number** | **2021** | **144.1450066** | **216.715562** | **90.8863118** |
| **YLDs (Years Lived with Disability)** | **Georgia** | **Both** | **All ages** | **Gout** | **Kidney dysfunction** | **Number** | **2021** | **144.1450066** | **216.715562** | **90.8863118** |
| **DALYs (Disability-Adjusted Life Years)** | **Cuba** | **Both** | **All ages** | **Gout** | **Kidney dysfunction** | **Number** | **2021** | **144.0332751** | **220.873468** | **90.1378185** |
| **YLDs (Years Lived with Disability)** | **Cuba** | **Both** | **All ages** | **Gout** | **Kidney dysfunction** | **Number** | **2021** | **144.0332751** | **220.873468** | **90.1378185** |
| **DALYs (Disability-Adjusted Life Years)** | **Ecuador** | **Both** | **All ages** | **Gout** | **Kidney dysfunction** | **Number** | **2021** | **142.6145767** | **216.407366** | **88.2571268** |
| **YLDs (Years Lived with Disability)** | **Ecuador** | **Both** | **All ages** | **Gout** | **Kidney dysfunction** | **Number** | **2021** | **142.6145767** | **216.407366** | **88.2571268** |
| **DALYs (Disability-Adjusted Life Years)** | **Burkina Faso** | **Both** | **All ages** | **Gout** | **Kidney dysfunction** | **Number** | **2021** | **142.5784444** | **217.175117** | **88.7820017** |
| **YLDs (Years Lived with Disability)** | **Burkina Faso** | **Both** | **All ages** | **Gout** | **Kidney dysfunction** | **Number** | **2021** | **142.5784444** | **217.175117** | **88.7820017** |
| **DALYs (Disability-Adjusted Life Years)** | **Jordan** | **Both** | **All ages** | **Gout** | **Kidney dysfunction** | **Number** | **2021** | **142.0576776** | **213.170035** | **87.6865477** |
| **YLDs (Years Lived with Disability)** | **Jordan** | **Both** | **All ages** | **Gout** | **Kidney dysfunction** | **Number** | **2021** | **142.0576776** | **213.170035** | **87.6865477** |
| **DALYs (Disability-Adjusted Life Years)** | **Mali** | **Both** | **All ages** | **Gout** | **Kidney dysfunction** | **Number** | **2021** | **140.9989728** | **206.962763** | **88.0294082** |
| **YLDs (Years Lived with Disability)** | **Mali** | **Both** | **All ages** | **Gout** | **Kidney dysfunction** | **Number** | **2021** | **140.9989728** | **206.962763** | **88.0294082** |
| **DALYs (Disability-Adjusted Life Years)** | **Zimbabwe** | **Both** | **All ages** | **Gout** | **Kidney dysfunction** | **Number** | **2021** | **140.3578377** | **217.44783** | **85.2337875** |
| **YLDs (Years Lived with Disability)** | **Zimbabwe** | **Both** | **All ages** | **Gout** | **Kidney dysfunction** | **Number** | **2021** | **140.3578377** | **217.44783** | **85.2337875** |
| **DALYs (Disability-Adjusted Life Years)** | **Afghanistan** | **Both** | **All ages** | **Gout** | **Kidney dysfunction** | **Number** | **2021** | **130.9171152** | **195.345177** | **81.8808384** |
| **YLDs (Years Lived with Disability)** | **Afghanistan** | **Both** | **All ages** | **Gout** | **Kidney dysfunction** | **Number** | **2021** | **130.9171152** | **195.345177** | **81.8808384** |
| **DALYs (Disability-Adjusted Life Years)** | **United Arab Emirates** | **Both** | **All ages** | **Gout** | **Kidney dysfunction** | **Number** | **2021** | **128.3182635** | **200.020266** | **76.9551356** |
| **YLDs (Years Lived with Disability)** | **United Arab Emirates** | **Both** | **All ages** | **Gout** | **Kidney dysfunction** | **Number** | **2021** | **128.3182635** | **200.020266** | **76.9551356** |
| **DALYs (Disability-Adjusted Life Years)** | **Republic of Moldova** | **Both** | **All ages** | **Gout** | **Kidney dysfunction** | **Number** | **2021** | **128.2311743** | **193.04428** | **80.7888835** |
| **YLDs (Years Lived with Disability)** | **Republic of Moldova** | **Both** | **All ages** | **Gout** | **Kidney dysfunction** | **Number** | **2021** | **128.2311743** | **193.04428** | **80.7888835** |
| **DALYs (Disability-Adjusted Life Years)** | **Uganda** | **Both** | **All ages** | **Gout** | **Kidney dysfunction** | **Number** | **2021** | **122.8651332** | **186.543454** | **75.8427471** |
| **YLDs (Years Lived with Disability)** | **Uganda** | **Both** | **All ages** | **Gout** | **Kidney dysfunction** | **Number** | **2021** | **122.8651332** | **186.543454** | **75.8427471** |
| **DALYs (Disability-Adjusted Life Years)** | **Slovakia** | **Both** | **All ages** | **Gout** | **Kidney dysfunction** | **Number** | **2021** | **119.9687296** | **181.851473** | **75.7119637** |
| **YLDs (Years Lived with Disability)** | **Slovakia** | **Both** | **All ages** | **Gout** | **Kidney dysfunction** | **Number** | **2021** | **119.9687296** | **181.851473** | **75.7119637** |
| **DALYs (Disability-Adjusted Life Years)** | **Croatia** | **Both** | **All ages** | **Gout** | **Kidney dysfunction** | **Number** | **2021** | **119.8736459** | **180.781975** | **73.6896525** |
| **YLDs (Years Lived with Disability)** | **Croatia** | **Both** | **All ages** | **Gout** | **Kidney dysfunction** | **Number** | **2021** | **119.8736459** | **180.781975** | **73.6896525** |
| **DALYs (Disability-Adjusted Life Years)** | **Niger** | **Both** | **All ages** | **Gout** | **Kidney dysfunction** | **Number** | **2021** | **118.6595063** | **181.910736** | **73.7579307** |
| **YLDs (Years Lived with Disability)** | **Niger** | **Both** | **All ages** | **Gout** | **Kidney dysfunction** | **Number** | **2021** | **118.6595063** | **181.910736** | **73.7579307** |
| **DALYs (Disability-Adjusted Life Years)** | **Armenia** | **Both** | **All ages** | **Gout** | **Kidney dysfunction** | **Number** | **2021** | **107.0089586** | **160.54492** | **68.0739794** |
| **YLDs (Years Lived with Disability)** | **Armenia** | **Both** | **All ages** | **Gout** | **Kidney dysfunction** | **Number** | **2021** | **107.0089586** | **160.54492** | **68.0739794** |
| **DALYs (Disability-Adjusted Life Years)** | **Lebanon** | **Both** | **All ages** | **Gout** | **Kidney dysfunction** | **Number** | **2021** | **106.1806542** | **158.622065** | **65.1407543** |
| **YLDs (Years Lived with Disability)** | **Lebanon** | **Both** | **All ages** | **Gout** | **Kidney dysfunction** | **Number** | **2021** | **106.1806542** | **158.622065** | **65.1407543** |
| **DALYs (Disability-Adjusted Life Years)** | **Tajikistan** | **Both** | **All ages** | **Gout** | **Kidney dysfunction** | **Number** | **2021** | **105.3208647** | **162.920706** | **64.890446** |
| **YLDs (Years Lived with Disability)** | **Tajikistan** | **Both** | **All ages** | **Gout** | **Kidney dysfunction** | **Number** | **2021** | **105.3208647** | **162.920706** | **64.890446** |
| **DALYs (Disability-Adjusted Life Years)** | **Lithuania** | **Both** | **All ages** | **Gout** | **Kidney dysfunction** | **Number** | **2021** | **105.1696648** | **160.438884** | **66.7004451** |
| **YLDs (Years Lived with Disability)** | **Lithuania** | **Both** | **All ages** | **Gout** | **Kidney dysfunction** | **Number** | **2021** | **105.1696648** | **160.438884** | **66.7004451** |
| **DALYs (Disability-Adjusted Life Years)** | **Kyrgyzstan** | **Both** | **All ages** | **Gout** | **Kidney dysfunction** | **Number** | **2021** | **101.6171776** | **153.885073** | **63.5592505** |
| **YLDs (Years Lived with Disability)** | **Kyrgyzstan** | **Both** | **All ages** | **Gout** | **Kidney dysfunction** | **Number** | **2021** | **101.6171776** | **153.885073** | **63.5592505** |
| **DALYs (Disability-Adjusted Life Years)** | **Turkmenistan** | **Both** | **All ages** | **Gout** | **Kidney dysfunction** | **Number** | **2021** | **98.82348487** | **148.429232** | **60.6453217** |
| **YLDs (Years Lived with Disability)** | **Turkmenistan** | **Both** | **All ages** | **Gout** | **Kidney dysfunction** | **Number** | **2021** | **98.82348487** | **148.429232** | **60.6453217** |
| **DALYs (Disability-Adjusted Life Years)** | **Lao People's Democratic Republic** | **Both** | **All ages** | **Gout** | **Kidney dysfunction** | **Number** | **2021** | **98.70843796** | **147.530568** | **62.230665** |
| **YLDs (Years Lived with Disability)** | **Lao People's Democratic Republic** | **Both** | **All ages** | **Gout** | **Kidney dysfunction** | **Number** | **2021** | **98.70843796** | **147.530568** | **62.230665** |
| **DALYs (Disability-Adjusted Life Years)** | **Senegal** | **Both** | **All ages** | **Gout** | **Kidney dysfunction** | **Number** | **2021** | **95.94722233** | **148.66678** | **58.8109579** |
| **YLDs (Years Lived with Disability)** | **Senegal** | **Both** | **All ages** | **Gout** | **Kidney dysfunction** | **Number** | **2021** | **95.94722233** | **148.66678** | **58.8109579** |
| **DALYs (Disability-Adjusted Life Years)** | **Guinea** | **Both** | **All ages** | **Gout** | **Kidney dysfunction** | **Number** | **2021** | **93.29595509** | **140.49684** | **57.3619586** |
| **YLDs (Years Lived with Disability)** | **Guinea** | **Both** | **All ages** | **Gout** | **Kidney dysfunction** | **Number** | **2021** | **93.29595509** | **140.49684** | **57.3619586** |
| **DALYs (Disability-Adjusted Life Years)** | **Papua New Guinea** | **Both** | **All ages** | **Gout** | **Kidney dysfunction** | **Number** | **2021** | **90.5834424** | **136.953332** | **54.5330424** |
| **YLDs (Years Lived with Disability)** | **Papua New Guinea** | **Both** | **All ages** | **Gout** | **Kidney dysfunction** | **Number** | **2021** | **90.5834424** | **136.953332** | **54.5330424** |
| **DALYs (Disability-Adjusted Life Years)** | **Chad** | **Both** | **All ages** | **Gout** | **Kidney dysfunction** | **Number** | **2021** | **89.81369197** | **136.610491** | **55.5300883** |
| **YLDs (Years Lived with Disability)** | **Chad** | **Both** | **All ages** | **Gout** | **Kidney dysfunction** | **Number** | **2021** | **89.81369197** | **136.610491** | **55.5300883** |
| **DALYs (Disability-Adjusted Life Years)** | **Libya** | **Both** | **All ages** | **Gout** | **Kidney dysfunction** | **Number** | **2021** | **88.85827801** | **132.21375** | **55.42731** |
| **YLDs (Years Lived with Disability)** | **Libya** | **Both** | **All ages** | **Gout** | **Kidney dysfunction** | **Number** | **2021** | **88.85827801** | **132.21375** | **55.42731** |
| **DALYs (Disability-Adjusted Life Years)** | **Benin** | **Both** | **All ages** | **Gout** | **Kidney dysfunction** | **Number** | **2021** | **86.90346528** | **131.969824** | **54.6354476** |
| **YLDs (Years Lived with Disability)** | **Benin** | **Both** | **All ages** | **Gout** | **Kidney dysfunction** | **Number** | **2021** | **86.90346528** | **131.969824** | **54.6354476** |
| **DALYs (Disability-Adjusted Life Years)** | **Mozambique** | **Both** | **All ages** | **Gout** | **Kidney dysfunction** | **Number** | **2021** | **81.49133126** | **121.577554** | **50.7076202** |
| **YLDs (Years Lived with Disability)** | **Mozambique** | **Both** | **All ages** | **Gout** | **Kidney dysfunction** | **Number** | **2021** | **81.49133126** | **121.577554** | **50.7076202** |
| **DALYs (Disability-Adjusted Life Years)** | **Bosnia and Herzegovina** | **Both** | **All ages** | **Gout** | **Kidney dysfunction** | **Number** | **2021** | **80.00012132** | **121.301513** | **49.3482403** |
| **YLDs (Years Lived with Disability)** | **Bosnia and Herzegovina** | **Both** | **All ages** | **Gout** | **Kidney dysfunction** | **Number** | **2021** | **80.00012132** | **121.301513** | **49.3482403** |
| **DALYs (Disability-Adjusted Life Years)** | **Madagascar** | **Both** | **All ages** | **Gout** | **Kidney dysfunction** | **Number** | **2021** | **79.27304738** | **118.958411** | **48.9397997** |
| **YLDs (Years Lived with Disability)** | **Madagascar** | **Both** | **All ages** | **Gout** | **Kidney dysfunction** | **Number** | **2021** | **79.27304738** | **118.958411** | **48.9397997** |
| **DALYs (Disability-Adjusted Life Years)** | **Guatemala** | **Both** | **All ages** | **Gout** | **Kidney dysfunction** | **Number** | **2021** | **76.70749014** | **115.930495** | **48.4908568** |
| **YLDs (Years Lived with Disability)** | **Guatemala** | **Both** | **All ages** | **Gout** | **Kidney dysfunction** | **Number** | **2021** | **76.70749014** | **115.930495** | **48.4908568** |
| **DALYs (Disability-Adjusted Life Years)** | **Latvia** | **Both** | **All ages** | **Gout** | **Kidney dysfunction** | **Number** | **2021** | **75.17656067** | **109.6453** | **46.148233** |
| **YLDs (Years Lived with Disability)** | **Latvia** | **Both** | **All ages** | **Gout** | **Kidney dysfunction** | **Number** | **2021** | **75.17656067** | **109.6453** | **46.148233** |
| **DALYs (Disability-Adjusted Life Years)** | **Dominican Republic** | **Both** | **All ages** | **Gout** | **Kidney dysfunction** | **Number** | **2021** | **74.47375206** | **112.415992** | **46.4196094** |
| **YLDs (Years Lived with Disability)** | **Dominican Republic** | **Both** | **All ages** | **Gout** | **Kidney dysfunction** | **Number** | **2021** | **74.47375206** | **112.415992** | **46.4196094** |
| **DALYs (Disability-Adjusted Life Years)** | **Bolivia (Plurinational State of)** | **Both** | **All ages** | **Gout** | **Kidney dysfunction** | **Number** | **2021** | **71.68101225** | **106.426601** | **43.8520452** |
| **YLDs (Years Lived with Disability)** | **Bolivia (Plurinational State of)** | **Both** | **All ages** | **Gout** | **Kidney dysfunction** | **Number** | **2021** | **71.68101225** | **106.426601** | **43.8520452** |
| **DALYs (Disability-Adjusted Life Years)** | **Puerto Rico** | **Both** | **All ages** | **Gout** | **Kidney dysfunction** | **Number** | **2021** | **66.20846478** | **101.874224** | **40.8628282** |
| **YLDs (Years Lived with Disability)** | **Puerto Rico** | **Both** | **All ages** | **Gout** | **Kidney dysfunction** | **Number** | **2021** | **66.20846478** | **101.874224** | **40.8628282** |
| **DALYs (Disability-Adjusted Life Years)** | **Congo** | **Both** | **All ages** | **Gout** | **Kidney dysfunction** | **Number** | **2021** | **64.65394976** | **99.7679096** | **40.4289557** |
| **YLDs (Years Lived with Disability)** | **Congo** | **Both** | **All ages** | **Gout** | **Kidney dysfunction** | **Number** | **2021** | **64.65394976** | **99.7679096** | **40.4289557** |
| **DALYs (Disability-Adjusted Life Years)** | **Paraguay** | **Both** | **All ages** | **Gout** | **Kidney dysfunction** | **Number** | **2021** | **58.94428306** | **87.8337638** | **37.2301745** |
| **YLDs (Years Lived with Disability)** | **Paraguay** | **Both** | **All ages** | **Gout** | **Kidney dysfunction** | **Number** | **2021** | **58.94428306** | **87.8337638** | **37.2301745** |
| **DALYs (Disability-Adjusted Life Years)** | **Sierra Leone** | **Both** | **All ages** | **Gout** | **Kidney dysfunction** | **Number** | **2021** | **58.50720056** | **89.4426867** | **36.1092758** |
| **YLDs (Years Lived with Disability)** | **Sierra Leone** | **Both** | **All ages** | **Gout** | **Kidney dysfunction** | **Number** | **2021** | **58.50720056** | **89.4426867** | **36.1092758** |
| **DALYs (Disability-Adjusted Life Years)** | **Malawi** | **Both** | **All ages** | **Gout** | **Kidney dysfunction** | **Number** | **2021** | **58.18490547** | **88.0462195** | **36.5346579** |
| **YLDs (Years Lived with Disability)** | **Malawi** | **Both** | **All ages** | **Gout** | **Kidney dysfunction** | **Number** | **2021** | **58.18490547** | **88.0462195** | **36.5346579** |
| **DALYs (Disability-Adjusted Life Years)** | **Zambia** | **Both** | **All ages** | **Gout** | **Kidney dysfunction** | **Number** | **2021** | **58.09182168** | **89.1577637** | **36.1689611** |
| **YLDs (Years Lived with Disability)** | **Zambia** | **Both** | **All ages** | **Gout** | **Kidney dysfunction** | **Number** | **2021** | **58.09182168** | **89.1577637** | **36.1689611** |
| **DALYs (Disability-Adjusted Life Years)** | **Slovenia** | **Both** | **All ages** | **Gout** | **Kidney dysfunction** | **Number** | **2021** | **56.90567094** | **85.9229611** | **34.8490824** |
| **YLDs (Years Lived with Disability)** | **Slovenia** | **Both** | **All ages** | **Gout** | **Kidney dysfunction** | **Number** | **2021** | **56.90567094** | **85.9229611** | **34.8490824** |
| **DALYs (Disability-Adjusted Life Years)** | **Albania** | **Both** | **All ages** | **Gout** | **Kidney dysfunction** | **Number** | **2021** | **55.97588708** | **84.2551363** | **34.7336744** |
| **YLDs (Years Lived with Disability)** | **Albania** | **Both** | **All ages** | **Gout** | **Kidney dysfunction** | **Number** | **2021** | **55.97588708** | **84.2551363** | **34.7336744** |
| **DALYs (Disability-Adjusted Life Years)** | **Togo** | **Both** | **All ages** | **Gout** | **Kidney dysfunction** | **Number** | **2021** | **55.18637033** | **82.226802** | **34.9126811** |
| **YLDs (Years Lived with Disability)** | **Togo** | **Both** | **All ages** | **Gout** | **Kidney dysfunction** | **Number** | **2021** | **55.18637033** | **82.226802** | **34.9126811** |
| **DALYs (Disability-Adjusted Life Years)** | **Mauritius** | **Both** | **All ages** | **Gout** | **Kidney dysfunction** | **Number** | **2021** | **54.65319162** | **82.8947649** | **34.8552313** |
| **YLDs (Years Lived with Disability)** | **Mauritius** | **Both** | **All ages** | **Gout** | **Kidney dysfunction** | **Number** | **2021** | **54.65319162** | **82.8947649** | **34.8552313** |
| **DALYs (Disability-Adjusted Life Years)** | **Estonia** | **Both** | **All ages** | **Gout** | **Kidney dysfunction** | **Number** | **2021** | **54.54205531** | **83.6586843** | **33.1051608** |
| **YLDs (Years Lived with Disability)** | **Estonia** | **Both** | **All ages** | **Gout** | **Kidney dysfunction** | **Number** | **2021** | **54.54205531** | **83.6586843** | **33.1051608** |
| **DALYs (Disability-Adjusted Life Years)** | **Kuwait** | **Both** | **All ages** | **Gout** | **Kidney dysfunction** | **Number** | **2021** | **52.48587065** | **78.5434813** | **32.5643114** |
| **YLDs (Years Lived with Disability)** | **Kuwait** | **Both** | **All ages** | **Gout** | **Kidney dysfunction** | **Number** | **2021** | **52.48587065** | **78.5434813** | **32.5643114** |
| **DALYs (Disability-Adjusted Life Years)** | **North Macedonia** | **Both** | **All ages** | **Gout** | **Kidney dysfunction** | **Number** | **2021** | **52.36419724** | **80.5529188** | **31.2406436** |
| **YLDs (Years Lived with Disability)** | **North Macedonia** | **Both** | **All ages** | **Gout** | **Kidney dysfunction** | **Number** | **2021** | **52.36419724** | **80.5529188** | **31.2406436** |
| **DALYs (Disability-Adjusted Life Years)** | **Cyprus** | **Both** | **All ages** | **Gout** | **Kidney dysfunction** | **Number** | **2021** | **51.12416499** | **78.7736929** | **32.3967576** |
| **YLDs (Years Lived with Disability)** | **Cyprus** | **Both** | **All ages** | **Gout** | **Kidney dysfunction** | **Number** | **2021** | **51.12416499** | **78.7736929** | **32.3967576** |
| **DALYs (Disability-Adjusted Life Years)** | **Nicaragua** | **Both** | **All ages** | **Gout** | **Kidney dysfunction** | **Number** | **2021** | **49.71101771** | **72.0931398** | **31.089617** |
| **YLDs (Years Lived with Disability)** | **Nicaragua** | **Both** | **All ages** | **Gout** | **Kidney dysfunction** | **Number** | **2021** | **49.71101771** | **72.0931398** | **31.089617** |
| **DALYs (Disability-Adjusted Life Years)** | **Costa Rica** | **Both** | **All ages** | **Gout** | **Kidney dysfunction** | **Number** | **2021** | **49.49587044** | **75.2102489** | **30.6760905** |
| **YLDs (Years Lived with Disability)** | **Costa Rica** | **Both** | **All ages** | **Gout** | **Kidney dysfunction** | **Number** | **2021** | **49.49587044** | **75.2102489** | **30.6760905** |
| **DALYs (Disability-Adjusted Life Years)** | **Mongolia** | **Both** | **All ages** | **Gout** | **Kidney dysfunction** | **Number** | **2021** | **49.04600433** | **74.8949498** | **30.563981** |
| **YLDs (Years Lived with Disability)** | **Mongolia** | **Both** | **All ages** | **Gout** | **Kidney dysfunction** | **Number** | **2021** | **49.04600433** | **74.8949498** | **30.563981** |
| **DALYs (Disability-Adjusted Life Years)** | **Rwanda** | **Both** | **All ages** | **Gout** | **Kidney dysfunction** | **Number** | **2021** | **46.3525755** | **69.2025994** | **28.1304136** |
| **YLDs (Years Lived with Disability)** | **Rwanda** | **Both** | **All ages** | **Gout** | **Kidney dysfunction** | **Number** | **2021** | **46.3525755** | **69.2025994** | **28.1304136** |
| **DALYs (Disability-Adjusted Life Years)** | **Honduras** | **Both** | **All ages** | **Gout** | **Kidney dysfunction** | **Number** | **2021** | **43.36796455** | **68.2155421** | **26.4827539** |
| **YLDs (Years Lived with Disability)** | **Honduras** | **Both** | **All ages** | **Gout** | **Kidney dysfunction** | **Number** | **2021** | **43.36796455** | **68.2155421** | **26.4827539** |
| **DALYs (Disability-Adjusted Life Years)** | **Haiti** | **Both** | **All ages** | **Gout** | **Kidney dysfunction** | **Number** | **2021** | **43.30230456** | **64.7501129** | **26.390659** |
| **YLDs (Years Lived with Disability)** | **Haiti** | **Both** | **All ages** | **Gout** | **Kidney dysfunction** | **Number** | **2021** | **43.30230456** | **64.7501129** | **26.390659** |
| **DALYs (Disability-Adjusted Life Years)** | **Central African Republic** | **Both** | **All ages** | **Gout** | **Kidney dysfunction** | **Number** | **2021** | **41.89123993** | **66.3524511** | **25.5956289** |
| **YLDs (Years Lived with Disability)** | **Central African Republic** | **Both** | **All ages** | **Gout** | **Kidney dysfunction** | **Number** | **2021** | **41.89123993** | **66.3524511** | **25.5956289** |
| **DALYs (Disability-Adjusted Life Years)** | **Palestine** | **Both** | **All ages** | **Gout** | **Kidney dysfunction** | **Number** | **2021** | **41.42819653** | **63.0914562** | **25.6741798** |
| **YLDs (Years Lived with Disability)** | **Palestine** | **Both** | **All ages** | **Gout** | **Kidney dysfunction** | **Number** | **2021** | **41.42819653** | **63.0914562** | **25.6741798** |
| **DALYs (Disability-Adjusted Life Years)** | **El Salvador** | **Both** | **All ages** | **Gout** | **Kidney dysfunction** | **Number** | **2021** | **41.17824933** | **61.8562826** | **25.7353616** |
| **YLDs (Years Lived with Disability)** | **El Salvador** | **Both** | **All ages** | **Gout** | **Kidney dysfunction** | **Number** | **2021** | **41.17824933** | **61.8562826** | **25.7353616** |
| **DALYs (Disability-Adjusted Life Years)** | **Mauritania** | **Both** | **All ages** | **Gout** | **Kidney dysfunction** | **Number** | **2021** | **40.51695023** | **62.8072309** | **26.0178147** |
| **YLDs (Years Lived with Disability)** | **Mauritania** | **Both** | **All ages** | **Gout** | **Kidney dysfunction** | **Number** | **2021** | **40.51695023** | **62.8072309** | **26.0178147** |
| **DALYs (Disability-Adjusted Life Years)** | **Somalia** | **Both** | **All ages** | **Gout** | **Kidney dysfunction** | **Number** | **2021** | **40.24476344** | **62.0806052** | **24.3830999** |
| **YLDs (Years Lived with Disability)** | **Somalia** | **Both** | **All ages** | **Gout** | **Kidney dysfunction** | **Number** | **2021** | **40.24476344** | **62.0806052** | **24.3830999** |
| **DALYs (Disability-Adjusted Life Years)** | **Burundi** | **Both** | **All ages** | **Gout** | **Kidney dysfunction** | **Number** | **2021** | **37.55860354** | **57.6884242** | **23.1521961** |
| **YLDs (Years Lived with Disability)** | **Burundi** | **Both** | **All ages** | **Gout** | **Kidney dysfunction** | **Number** | **2021** | **37.55860354** | **57.6884242** | **23.1521961** |
| **DALYs (Disability-Adjusted Life Years)** | **Liberia** | **Both** | **All ages** | **Gout** | **Kidney dysfunction** | **Number** | **2021** | **36.23640744** | **54.3698374** | **23.0552945** |
| **YLDs (Years Lived with Disability)** | **Liberia** | **Both** | **All ages** | **Gout** | **Kidney dysfunction** | **Number** | **2021** | **36.23640744** | **54.3698374** | **23.0552945** |
| **DALYs (Disability-Adjusted Life Years)** | **Botswana** | **Both** | **All ages** | **Gout** | **Kidney dysfunction** | **Number** | **2021** | **35.05769621** | **53.5502141** | **22.0717649** |
| **YLDs (Years Lived with Disability)** | **Botswana** | **Both** | **All ages** | **Gout** | **Kidney dysfunction** | **Number** | **2021** | **35.05769621** | **53.5502141** | **22.0717649** |
| **DALYs (Disability-Adjusted Life Years)** | **Oman** | **Both** | **All ages** | **Gout** | **Kidney dysfunction** | **Number** | **2021** | **34.89008781** | **52.3110027** | **21.6021124** |
| **YLDs (Years Lived with Disability)** | **Oman** | **Both** | **All ages** | **Gout** | **Kidney dysfunction** | **Number** | **2021** | **34.89008781** | **52.3110027** | **21.6021124** |
| **DALYs (Disability-Adjusted Life Years)** | **South Sudan** | **Both** | **All ages** | **Gout** | **Kidney dysfunction** | **Number** | **2021** | **31.51572012** | **47.3623716** | **19.0829698** |
| **YLDs (Years Lived with Disability)** | **South Sudan** | **Both** | **All ages** | **Gout** | **Kidney dysfunction** | **Number** | **2021** | **31.51572012** | **47.3623716** | **19.0829698** |
| **DALYs (Disability-Adjusted Life Years)** | **Panama** | **Both** | **All ages** | **Gout** | **Kidney dysfunction** | **Number** | **2021** | **31.35712952** | **46.7052692** | **19.9852052** |
| **YLDs (Years Lived with Disability)** | **Panama** | **Both** | **All ages** | **Gout** | **Kidney dysfunction** | **Number** | **2021** | **31.35712952** | **46.7052692** | **19.9852052** |
| **DALYs (Disability-Adjusted Life Years)** | **Namibia** | **Both** | **All ages** | **Gout** | **Kidney dysfunction** | **Number** | **2021** | **29.68444715** | **44.4262053** | **18.3281291** |
| **YLDs (Years Lived with Disability)** | **Namibia** | **Both** | **All ages** | **Gout** | **Kidney dysfunction** | **Number** | **2021** | **29.68444715** | **44.4262053** | **18.3281291** |
| **DALYs (Disability-Adjusted Life Years)** | **Gabon** | **Both** | **All ages** | **Gout** | **Kidney dysfunction** | **Number** | **2021** | **27.91359006** | **42.8271314** | **17.7569705** |
| **YLDs (Years Lived with Disability)** | **Gabon** | **Both** | **All ages** | **Gout** | **Kidney dysfunction** | **Number** | **2021** | **27.91359006** | **42.8271314** | **17.7569705** |
| **DALYs (Disability-Adjusted Life Years)** | **Jamaica** | **Both** | **All ages** | **Gout** | **Kidney dysfunction** | **Number** | **2021** | **26.67197623** | **40.3367829** | **16.6151209** |
| **YLDs (Years Lived with Disability)** | **Jamaica** | **Both** | **All ages** | **Gout** | **Kidney dysfunction** | **Number** | **2021** | **26.67197623** | **40.3367829** | **16.6151209** |
| **DALYs (Disability-Adjusted Life Years)** | **Malta** | **Both** | **All ages** | **Gout** | **Kidney dysfunction** | **Number** | **2021** | **24.9744851** | **37.6619018** | **15.4136101** |
| **YLDs (Years Lived with Disability)** | **Malta** | **Both** | **All ages** | **Gout** | **Kidney dysfunction** | **Number** | **2021** | **24.9744851** | **37.6619018** | **15.4136101** |
| **DALYs (Disability-Adjusted Life Years)** | **Luxembourg** | **Both** | **All ages** | **Gout** | **Kidney dysfunction** | **Number** | **2021** | **24.00636729** | **37.7904344** | **14.6920272** |
| **YLDs (Years Lived with Disability)** | **Luxembourg** | **Both** | **All ages** | **Gout** | **Kidney dysfunction** | **Number** | **2021** | **24.00636729** | **37.7904344** | **14.6920272** |
| **DALYs (Disability-Adjusted Life Years)** | **Lesotho** | **Both** | **All ages** | **Gout** | **Kidney dysfunction** | **Number** | **2021** | **23.46468251** | **35.2402942** | **14.641594** |
| **YLDs (Years Lived with Disability)** | **Lesotho** | **Both** | **All ages** | **Gout** | **Kidney dysfunction** | **Number** | **2021** | **23.46468251** | **35.2402942** | **14.641594** |
| **DALYs (Disability-Adjusted Life Years)** | **Qatar** | **Both** | **All ages** | **Gout** | **Kidney dysfunction** | **Number** | **2021** | **20.92920156** | **30.8711385** | **12.837013** |
| **YLDs (Years Lived with Disability)** | **Qatar** | **Both** | **All ages** | **Gout** | **Kidney dysfunction** | **Number** | **2021** | **20.92920156** | **30.8711385** | **12.837013** |
| **DALYs (Disability-Adjusted Life Years)** | **Fiji** | **Both** | **All ages** | **Gout** | **Kidney dysfunction** | **Number** | **2021** | **20.07318935** | **30.0988191** | **12.4870536** |
| **YLDs (Years Lived with Disability)** | **Fiji** | **Both** | **All ages** | **Gout** | **Kidney dysfunction** | **Number** | **2021** | **20.07318935** | **30.0988191** | **12.4870536** |
| **DALYs (Disability-Adjusted Life Years)** | **Eritrea** | **Both** | **All ages** | **Gout** | **Kidney dysfunction** | **Number** | **2021** | **18.94111928** | **28.9780668** | **11.8721342** |
| **YLDs (Years Lived with Disability)** | **Eritrea** | **Both** | **All ages** | **Gout** | **Kidney dysfunction** | **Number** | **2021** | **18.94111928** | **28.9780668** | **11.8721342** |
| **DALYs (Disability-Adjusted Life Years)** | **Timor-Leste** | **Both** | **All ages** | **Gout** | **Kidney dysfunction** | **Number** | **2021** | **17.05708391** | **25.8966466** | **10.6457007** |
| **YLDs (Years Lived with Disability)** | **Timor-Leste** | **Both** | **All ages** | **Gout** | **Kidney dysfunction** | **Number** | **2021** | **17.05708391** | **25.8966466** | **10.6457007** |
| **DALYs (Disability-Adjusted Life Years)** | **Bahrain** | **Both** | **All ages** | **Gout** | **Kidney dysfunction** | **Number** | **2021** | **16.63903048** | **26.0274468** | **10.241549** |
| **YLDs (Years Lived with Disability)** | **Bahrain** | **Both** | **All ages** | **Gout** | **Kidney dysfunction** | **Number** | **2021** | **16.63903048** | **26.0274468** | **10.241549** |
| **DALYs (Disability-Adjusted Life Years)** | **Gambia** | **Both** | **All ages** | **Gout** | **Kidney dysfunction** | **Number** | **2021** | **16.20707666** | **24.2978896** | **10.2306497** |
| **YLDs (Years Lived with Disability)** | **Gambia** | **Both** | **All ages** | **Gout** | **Kidney dysfunction** | **Number** | **2021** | **16.20707666** | **24.2978896** | **10.2306497** |
| **DALYs (Disability-Adjusted Life Years)** | **Trinidad and Tobago** | **Both** | **All ages** | **Gout** | **Kidney dysfunction** | **Number** | **2021** | **16.15910501** | **24.4619553** | **9.83966005** |
| **YLDs (Years Lived with Disability)** | **Trinidad and Tobago** | **Both** | **All ages** | **Gout** | **Kidney dysfunction** | **Number** | **2021** | **16.15910501** | **24.4619553** | **9.83966005** |
| **DALYs (Disability-Adjusted Life Years)** | **Eswatini** | **Both** | **All ages** | **Gout** | **Kidney dysfunction** | **Number** | **2021** | **13.8830087** | **21.0508767** | **8.93689892** |
| **YLDs (Years Lived with Disability)** | **Eswatini** | **Both** | **All ages** | **Gout** | **Kidney dysfunction** | **Number** | **2021** | **13.8830087** | **21.0508767** | **8.93689892** |
| **DALYs (Disability-Adjusted Life Years)** | **Montenegro** | **Both** | **All ages** | **Gout** | **Kidney dysfunction** | **Number** | **2021** | **13.59258972** | **20.2268673** | **8.44030937** |
| **YLDs (Years Lived with Disability)** | **Montenegro** | **Both** | **All ages** | **Gout** | **Kidney dysfunction** | **Number** | **2021** | **13.59258972** | **20.2268673** | **8.44030937** |
| **DALYs (Disability-Adjusted Life Years)** | **Equatorial Guinea** | **Both** | **All ages** | **Gout** | **Kidney dysfunction** | **Number** | **2021** | **12.23528818** | **18.5776013** | **7.66497272** |
| **YLDs (Years Lived with Disability)** | **Equatorial Guinea** | **Both** | **All ages** | **Gout** | **Kidney dysfunction** | **Number** | **2021** | **12.23528818** | **18.5776013** | **7.66497272** |
| **DALYs (Disability-Adjusted Life Years)** | **Iceland** | **Both** | **All ages** | **Gout** | **Kidney dysfunction** | **Number** | **2021** | **11.77697086** | **17.7813812** | **7.33015951** |
| **YLDs (Years Lived with Disability)** | **Iceland** | **Both** | **All ages** | **Gout** | **Kidney dysfunction** | **Number** | **2021** | **11.77697086** | **17.7813812** | **7.33015951** |
| **DALYs (Disability-Adjusted Life Years)** | **Bhutan** | **Both** | **All ages** | **Gout** | **Kidney dysfunction** | **Number** | **2021** | **11.42127567** | **16.7791286** | **6.99075442** |
| **YLDs (Years Lived with Disability)** | **Bhutan** | **Both** | **All ages** | **Gout** | **Kidney dysfunction** | **Number** | **2021** | **11.42127567** | **16.7791286** | **6.99075442** |
| **DALYs (Disability-Adjusted Life Years)** | **Brunei Darussalam** | **Both** | **All ages** | **Gout** | **Kidney dysfunction** | **Number** | **2021** | **10.81278627** | **16.6140707** | **6.73276914** |
| **YLDs (Years Lived with Disability)** | **Brunei Darussalam** | **Both** | **All ages** | **Gout** | **Kidney dysfunction** | **Number** | **2021** | **10.81278627** | **16.6140707** | **6.73276914** |
| **DALYs (Disability-Adjusted Life Years)** | **Guinea-Bissau** | **Both** | **All ages** | **Gout** | **Kidney dysfunction** | **Number** | **2021** | **10.67242714** | **16.0900882** | **6.78506803** |
| **YLDs (Years Lived with Disability)** | **Guinea-Bissau** | **Both** | **All ages** | **Gout** | **Kidney dysfunction** | **Number** | **2021** | **10.67242714** | **16.0900882** | **6.78506803** |
| **DALYs (Disability-Adjusted Life Years)** | **Maldives** | **Both** | **All ages** | **Gout** | **Kidney dysfunction** | **Number** | **2021** | **7.692874476** | **11.6278254** | **4.80760645** |
| **YLDs (Years Lived with Disability)** | **Maldives** | **Both** | **All ages** | **Gout** | **Kidney dysfunction** | **Number** | **2021** | **7.692874476** | **11.6278254** | **4.80760645** |
| **DALYs (Disability-Adjusted Life Years)** | **Solomon Islands** | **Both** | **All ages** | **Gout** | **Kidney dysfunction** | **Number** | **2021** | **7.468938861** | **11.1180642** | **4.74117861** |
| **YLDs (Years Lived with Disability)** | **Solomon Islands** | **Both** | **All ages** | **Gout** | **Kidney dysfunction** | **Number** | **2021** | **7.468938861** | **11.1180642** | **4.74117861** |
| **DALYs (Disability-Adjusted Life Years)** | **Cabo Verde** | **Both** | **All ages** | **Gout** | **Kidney dysfunction** | **Number** | **2021** | **7.325401756** | **10.9747241** | **4.5577557** |
| **YLDs (Years Lived with Disability)** | **Cabo Verde** | **Both** | **All ages** | **Gout** | **Kidney dysfunction** | **Number** | **2021** | **7.325401756** | **10.9747241** | **4.5577557** |
| **DALYs (Disability-Adjusted Life Years)** | **Guam** | **Both** | **All ages** | **Gout** | **Kidney dysfunction** | **Number** | **2021** | **6.062432056** | **9.11949878** | **3.80927053** |
| **YLDs (Years Lived with Disability)** | **Guam** | **Both** | **All ages** | **Gout** | **Kidney dysfunction** | **Number** | **2021** | **6.062432056** | **9.11949878** | **3.80927053** |
| **DALYs (Disability-Adjusted Life Years)** | **Djibouti** | **Both** | **All ages** | **Gout** | **Kidney dysfunction** | **Number** | **2021** | **5.233105584** | **7.99878804** | **3.17529658** |
| **YLDs (Years Lived with Disability)** | **Djibouti** | **Both** | **All ages** | **Gout** | **Kidney dysfunction** | **Number** | **2021** | **5.233105584** | **7.99878804** | **3.17529658** |
| **DALYs (Disability-Adjusted Life Years)** | **Suriname** | **Both** | **All ages** | **Gout** | **Kidney dysfunction** | **Number** | **2021** | **5.06859709** | **7.63038115** | **3.1763447** |
| **YLDs (Years Lived with Disability)** | **Suriname** | **Both** | **All ages** | **Gout** | **Kidney dysfunction** | **Number** | **2021** | **5.06859709** | **7.63038115** | **3.1763447** |
| **DALYs (Disability-Adjusted Life Years)** | **Guyana** | **Both** | **All ages** | **Gout** | **Kidney dysfunction** | **Number** | **2021** | **4.779388136** | **7.16658297** | **3.04915247** |
| **YLDs (Years Lived with Disability)** | **Guyana** | **Both** | **All ages** | **Gout** | **Kidney dysfunction** | **Number** | **2021** | **4.779388136** | **7.16658297** | **3.04915247** |
| **DALYs (Disability-Adjusted Life Years)** | **Comoros** | **Both** | **All ages** | **Gout** | **Kidney dysfunction** | **Number** | **2021** | **4.057855416** | **5.96478005** | **2.52886455** |
| **YLDs (Years Lived with Disability)** | **Comoros** | **Both** | **All ages** | **Gout** | **Kidney dysfunction** | **Number** | **2021** | **4.057855416** | **5.96478005** | **2.52886455** |
| **DALYs (Disability-Adjusted Life Years)** | **Barbados** | **Both** | **All ages** | **Gout** | **Kidney dysfunction** | **Number** | **2021** | **4.034063565** | **5.9983005** | **2.46329057** |
| **YLDs (Years Lived with Disability)** | **Barbados** | **Both** | **All ages** | **Gout** | **Kidney dysfunction** | **Number** | **2021** | **4.034063565** | **5.9983005** | **2.46329057** |
| **DALYs (Disability-Adjusted Life Years)** | **Samoa** | **Both** | **All ages** | **Gout** | **Kidney dysfunction** | **Number** | **2021** | **3.975226699** | **5.98452461** | **2.4804545** |
| **YLDs (Years Lived with Disability)** | **Samoa** | **Both** | **All ages** | **Gout** | **Kidney dysfunction** | **Number** | **2021** | **3.975226699** | **5.98452461** | **2.4804545** |
| **DALYs (Disability-Adjusted Life Years)** | **Vanuatu** | **Both** | **All ages** | **Gout** | **Kidney dysfunction** | **Number** | **2021** | **3.966452746** | **5.98008136** | **2.43100749** |
| **YLDs (Years Lived with Disability)** | **Vanuatu** | **Both** | **All ages** | **Gout** | **Kidney dysfunction** | **Number** | **2021** | **3.966452746** | **5.98008136** | **2.43100749** |
| **DALYs (Disability-Adjusted Life Years)** | **Andorra** | **Both** | **All ages** | **Gout** | **Kidney dysfunction** | **Number** | **2021** | **3.735386955** | **5.8049184** | **2.31936001** |
| **YLDs (Years Lived with Disability)** | **Andorra** | **Both** | **All ages** | **Gout** | **Kidney dysfunction** | **Number** | **2021** | **3.735386955** | **5.8049184** | **2.31936001** |
| **DALYs (Disability-Adjusted Life Years)** | **Greenland** | **Both** | **All ages** | **Gout** | **Kidney dysfunction** | **Number** | **2021** | **3.232867222** | **4.84770676** | **2.02934161** |
| **YLDs (Years Lived with Disability)** | **Greenland** | **Both** | **All ages** | **Gout** | **Kidney dysfunction** | **Number** | **2021** | **3.232867222** | **4.84770676** | **2.02934161** |
| **DALYs (Disability-Adjusted Life Years)** | **Bahamas** | **Both** | **All ages** | **Gout** | **Kidney dysfunction** | **Number** | **2021** | **3.041662164** | **4.66664505** | **1.87814209** |
| **YLDs (Years Lived with Disability)** | **Bahamas** | **Both** | **All ages** | **Gout** | **Kidney dysfunction** | **Number** | **2021** | **3.041662164** | **4.66664505** | **1.87814209** |
| **DALYs (Disability-Adjusted Life Years)** | **Seychelles** | **Both** | **All ages** | **Gout** | **Kidney dysfunction** | **Number** | **2021** | **2.976927912** | **4.49577414** | **1.8516025** |
| **YLDs (Years Lived with Disability)** | **Seychelles** | **Both** | **All ages** | **Gout** | **Kidney dysfunction** | **Number** | **2021** | **2.976927912** | **4.49577414** | **1.8516025** |
| **DALYs (Disability-Adjusted Life Years)** | **Belize** | **Both** | **All ages** | **Gout** | **Kidney dysfunction** | **Number** | **2021** | **2.608301749** | **3.87095954** | **1.60935497** |
| **YLDs (Years Lived with Disability)** | **Belize** | **Both** | **All ages** | **Gout** | **Kidney dysfunction** | **Number** | **2021** | **2.608301749** | **3.87095954** | **1.60935497** |
| **DALYs (Disability-Adjusted Life Years)** | **Monaco** | **Both** | **All ages** | **Gout** | **Kidney dysfunction** | **Number** | **2021** | **2.556861358** | **3.92828064** | **1.572371** |
| **YLDs (Years Lived with Disability)** | **Monaco** | **Both** | **All ages** | **Gout** | **Kidney dysfunction** | **Number** | **2021** | **2.556861358** | **3.92828064** | **1.572371** |
| **DALYs (Disability-Adjusted Life Years)** | **Tonga** | **Both** | **All ages** | **Gout** | **Kidney dysfunction** | **Number** | **2021** | **2.315895402** | **3.46738625** | **1.44968421** |
| **YLDs (Years Lived with Disability)** | **Tonga** | **Both** | **All ages** | **Gout** | **Kidney dysfunction** | **Number** | **2021** | **2.315895402** | **3.46738625** | **1.44968421** |
| **DALYs (Disability-Adjusted Life Years)** | **Sao Tome and Principe** | **Both** | **All ages** | **Gout** | **Kidney dysfunction** | **Number** | **2021** | **2.130289756** | **3.19086675** | **1.3728116** |
| **YLDs (Years Lived with Disability)** | **Sao Tome and Principe** | **Both** | **All ages** | **Gout** | **Kidney dysfunction** | **Number** | **2021** | **2.130289756** | **3.19086675** | **1.3728116** |
| **DALYs (Disability-Adjusted Life Years)** | **Micronesia (Federated States of)** | **Both** | **All ages** | **Gout** | **Kidney dysfunction** | **Number** | **2021** | **1.97589498** | **2.98455845** | **1.23988602** |
| **YLDs (Years Lived with Disability)** | **Micronesia (Federated States of)** | **Both** | **All ages** | **Gout** | **Kidney dysfunction** | **Number** | **2021** | **1.97589498** | **2.98455845** | **1.23988602** |
| **DALYs (Disability-Adjusted Life Years)** | **Saint Lucia** | **Both** | **All ages** | **Gout** | **Kidney dysfunction** | **Number** | **2021** | **1.916998393** | **2.88676752** | **1.19157128** |
| **YLDs (Years Lived with Disability)** | **Saint Lucia** | **Both** | **All ages** | **Gout** | **Kidney dysfunction** | **Number** | **2021** | **1.916998393** | **2.88676752** | **1.19157128** |
| **DALYs (Disability-Adjusted Life Years)** | **San Marino** | **Both** | **All ages** | **Gout** | **Kidney dysfunction** | **Number** | **2021** | **1.875942395** | **2.87402204** | **1.17749596** |
| **YLDs (Years Lived with Disability)** | **San Marino** | **Both** | **All ages** | **Gout** | **Kidney dysfunction** | **Number** | **2021** | **1.875942395** | **2.87402204** | **1.17749596** |
| **DALYs (Disability-Adjusted Life Years)** | **Northern Mariana Islands** | **Both** | **All ages** | **Gout** | **Kidney dysfunction** | **Number** | **2021** | **1.687042283** | **2.56421794** | **1.07377918** |
| **YLDs (Years Lived with Disability)** | **Northern Mariana Islands** | **Both** | **All ages** | **Gout** | **Kidney dysfunction** | **Number** | **2021** | **1.687042283** | **2.56421794** | **1.07377918** |
| **DALYs (Disability-Adjusted Life Years)** | **United States Virgin Islands** | **Both** | **All ages** | **Gout** | **Kidney dysfunction** | **Number** | **2021** | **1.601549781** | **2.45369613** | **0.96485277** |
| **YLDs (Years Lived with Disability)** | **United States Virgin Islands** | **Both** | **All ages** | **Gout** | **Kidney dysfunction** | **Number** | **2021** | **1.601549781** | **2.45369613** | **0.96485277** |
| **DALYs (Disability-Adjusted Life Years)** | **Kiribati** | **Both** | **All ages** | **Gout** | **Kidney dysfunction** | **Number** | **2021** | **1.577056447** | **2.3997193** | **0.99130828** |
| **YLDs (Years Lived with Disability)** | **Kiribati** | **Both** | **All ages** | **Gout** | **Kidney dysfunction** | **Number** | **2021** | **1.577056447** | **2.3997193** | **0.99130828** |
| **DALYs (Disability-Adjusted Life Years)** | **American Samoa** | **Both** | **All ages** | **Gout** | **Kidney dysfunction** | **Number** | **2021** | **1.573298905** | **2.34644993** | **1.00045648** |
| **YLDs (Years Lived with Disability)** | **American Samoa** | **Both** | **All ages** | **Gout** | **Kidney dysfunction** | **Number** | **2021** | **1.573298905** | **2.34644993** | **1.00045648** |
| **DALYs (Disability-Adjusted Life Years)** | **Saint Vincent and the Grenadines** | **Both** | **All ages** | **Gout** | **Kidney dysfunction** | **Number** | **2021** | **1.13999391** | **1.72834357** | **0.69667545** |
| **YLDs (Years Lived with Disability)** | **Saint Vincent and the Grenadines** | **Both** | **All ages** | **Gout** | **Kidney dysfunction** | **Number** | **2021** | **1.13999391** | **1.72834357** | **0.69667545** |
| **DALYs (Disability-Adjusted Life Years)** | **Bermuda** | **Both** | **All ages** | **Gout** | **Kidney dysfunction** | **Number** | **2021** | **1.052428465** | **1.55576109** | **0.65225345** |
| **YLDs (Years Lived with Disability)** | **Bermuda** | **Both** | **All ages** | **Gout** | **Kidney dysfunction** | **Number** | **2021** | **1.052428465** | **1.55576109** | **0.65225345** |
| **DALYs (Disability-Adjusted Life Years)** | **Grenada** | **Both** | **All ages** | **Gout** | **Kidney dysfunction** | **Number** | **2021** | **0.886789986** | **1.36329217** | **0.53521344** |
| **YLDs (Years Lived with Disability)** | **Grenada** | **Both** | **All ages** | **Gout** | **Kidney dysfunction** | **Number** | **2021** | **0.886789986** | **1.36329217** | **0.53521344** |
| **DALYs (Disability-Adjusted Life Years)** | **Antigua and Barbuda** | **Both** | **All ages** | **Gout** | **Kidney dysfunction** | **Number** | **2021** | **0.822606899** | **1.22545598** | **0.51434158** |
| **YLDs (Years Lived with Disability)** | **Antigua and Barbuda** | **Both** | **All ages** | **Gout** | **Kidney dysfunction** | **Number** | **2021** | **0.822606899** | **1.22545598** | **0.51434158** |
| **DALYs (Disability-Adjusted Life Years)** | **Marshall Islands** | **Both** | **All ages** | **Gout** | **Kidney dysfunction** | **Number** | **2021** | **0.791551628** | **1.21805572** | **0.49654016** |
| **YLDs (Years Lived with Disability)** | **Marshall Islands** | **Both** | **All ages** | **Gout** | **Kidney dysfunction** | **Number** | **2021** | **0.791551628** | **1.21805572** | **0.49654016** |
| **DALYs (Disability-Adjusted Life Years)** | **Cook Islands** | **Both** | **All ages** | **Gout** | **Kidney dysfunction** | **Number** | **2021** | **0.766992708** | **1.1676905** | **0.47758448** |
| **YLDs (Years Lived with Disability)** | **Cook Islands** | **Both** | **All ages** | **Gout** | **Kidney dysfunction** | **Number** | **2021** | **0.766992708** | **1.1676905** | **0.47758448** |
| **DALYs (Disability-Adjusted Life Years)** | **Dominica** | **Both** | **All ages** | **Gout** | **Kidney dysfunction** | **Number** | **2021** | **0.716075224** | **1.12751471** | **0.43524172** |
| **YLDs (Years Lived with Disability)** | **Dominica** | **Both** | **All ages** | **Gout** | **Kidney dysfunction** | **Number** | **2021** | **0.716075224** | **1.12751471** | **0.43524172** |
| **DALYs (Disability-Adjusted Life Years)** | **Palau** | **Both** | **All ages** | **Gout** | **Kidney dysfunction** | **Number** | **2021** | **0.676305202** | **1.01707638** | **0.4229335** |
| **YLDs (Years Lived with Disability)** | **Palau** | **Both** | **All ages** | **Gout** | **Kidney dysfunction** | **Number** | **2021** | **0.676305202** | **1.01707638** | **0.4229335** |
| **DALYs (Disability-Adjusted Life Years)** | **Saint Kitts and Nevis** | **Both** | **All ages** | **Gout** | **Kidney dysfunction** | **Number** | **2021** | **0.521510828** | **0.79335455** | **0.32598724** |
| **YLDs (Years Lived with Disability)** | **Saint Kitts and Nevis** | **Both** | **All ages** | **Gout** | **Kidney dysfunction** | **Number** | **2021** | **0.521510828** | **0.79335455** | **0.32598724** |
| **DALYs (Disability-Adjusted Life Years)** | **Tuvalu** | **Both** | **All ages** | **Gout** | **Kidney dysfunction** | **Number** | **2021** | **0.262432917** | **0.39512077** | **0.16233249** |
| **YLDs (Years Lived with Disability)** | **Tuvalu** | **Both** | **All ages** | **Gout** | **Kidney dysfunction** | **Number** | **2021** | **0.262432917** | **0.39512077** | **0.16233249** |
| **DALYs (Disability-Adjusted Life Years)** | **Nauru** | **Both** | **All ages** | **Gout** | **Kidney dysfunction** | **Number** | **2021** | **0.145968229** | **0.21555594** | **0.0918049** |
| **YLDs (Years Lived with Disability)** | **Nauru** | **Both** | **All ages** | **Gout** | **Kidney dysfunction** | **Number** | **2021** | **0.145968229** | **0.21555594** | **0.0918049** |
| **DALYs (Disability-Adjusted Life Years)** | **Niue** | **Both** | **All ages** | **Gout** | **Kidney dysfunction** | **Number** | **2021** | **0.063929541** | **0.09741243** | **0.03960407** |
| **YLDs (Years Lived with Disability)** | **Niue** | **Both** | **All ages** | **Gout** | **Kidney dysfunction** | **Number** | **2021** | **0.063929541** | **0.09741243** | **0.03960407** |
| **DALYs (Disability-Adjusted Life Years)** | **Tokelau** | **Both** | **All ages** | **Gout** | **Kidney dysfunction** | **Number** | **2021** | **0.041156532** | **0.061912** | **0.02594382** |
| **YLDs (Years Lived with Disability)** | **Tokelau** | **Both** | **All ages** | **Gout** | **Kidney dysfunction** | **Number** | **2021** | **0.041156532** | **0.061912** | **0.02594382** |

**Appendix 6：The age-standardized DALY rate and age-standardized YLD rate of gout attributable to kidney dysfunction across 204 countries in 2021.**

| **measure** | **location** | **sex** | **age** | **cause** | **rei** | **metric** | **year** | **val** | **upper** | **lower** |
| --- | --- | --- | --- | --- | --- | --- | --- | --- | --- | --- |
| **DALYs (Disability-Adjusted Life Years)** | **United States of America** | **Both** | **Age-standardized** | **Gout** | **Kidney dysfunction** | **Rate** | **2021** | **7.255463865** | **10.6201942** | **4.75889028** |
| **YLDs (Years Lived with Disability)** | **United States of America** | **Both** | **Age-standardized** | **Gout** | **Kidney dysfunction** | **Rate** | **2021** | **7.255463865** | **10.6201942** | **4.75889028** |
| **DALYs (Disability-Adjusted Life Years)** | **New Zealand** | **Both** | **Age-standardized** | **Gout** | **Kidney dysfunction** | **Rate** | **2021** | **5.438418464** | **8.20033509** | **3.38228587** |
| **YLDs (Years Lived with Disability)** | **New Zealand** | **Both** | **Age-standardized** | **Gout** | **Kidney dysfunction** | **Rate** | **2021** | **5.438418464** | **8.20033509** | **3.38228587** |
| **DALYs (Disability-Adjusted Life Years)** | **Greenland** | **Both** | **Age-standardized** | **Gout** | **Kidney dysfunction** | **Rate** | **2021** | **5.283550863** | **7.87512016** | **3.34125002** |
| **YLDs (Years Lived with Disability)** | **Greenland** | **Both** | **Age-standardized** | **Gout** | **Kidney dysfunction** | **Rate** | **2021** | **5.283550863** | **7.87512016** | **3.34125002** |
| **DALYs (Disability-Adjusted Life Years)** | **Australia** | **Both** | **Age-standardized** | **Gout** | **Kidney dysfunction** | **Rate** | **2021** | **5.219079969** | **8.01904172** | **3.16325049** |
| **YLDs (Years Lived with Disability)** | **Australia** | **Both** | **Age-standardized** | **Gout** | **Kidney dysfunction** | **Rate** | **2021** | **5.219079969** | **8.01904172** | **3.16325049** |
| **DALYs (Disability-Adjusted Life Years)** | **Canada** | **Both** | **Age-standardized** | **Gout** | **Kidney dysfunction** | **Rate** | **2021** | **4.428704276** | **6.58112587** | **2.74247978** |
| **YLDs (Years Lived with Disability)** | **Canada** | **Both** | **Age-standardized** | **Gout** | **Kidney dysfunction** | **Rate** | **2021** | **4.428704276** | **6.58112587** | **2.74247978** |
| **DALYs (Disability-Adjusted Life Years)** | **Northern Mariana Islands** | **Both** | **Age-standardized** | **Gout** | **Kidney dysfunction** | **Rate** | **2021** | **3.567196868** | **5.41873399** | **2.25788868** |
| **YLDs (Years Lived with Disability)** | **Northern Mariana Islands** | **Both** | **Age-standardized** | **Gout** | **Kidney dysfunction** | **Rate** | **2021** | **3.567196868** | **5.41873399** | **2.25788868** |
| **DALYs (Disability-Adjusted Life Years)** | **Taiwan (Province of China)** | **Both** | **Age-standardized** | **Gout** | **Kidney dysfunction** | **Rate** | **2021** | **3.549424556** | **5.21658706** | **2.28011097** |
| **YLDs (Years Lived with Disability)** | **Taiwan (Province of China)** | **Both** | **Age-standardized** | **Gout** | **Kidney dysfunction** | **Rate** | **2021** | **3.549424556** | **5.21658706** | **2.28011097** |
| **DALYs (Disability-Adjusted Life Years)** | **American Samoa** | **Both** | **Age-standardized** | **Gout** | **Kidney dysfunction** | **Rate** | **2021** | **3.546701439** | **5.18899466** | **2.21877062** |
| **YLDs (Years Lived with Disability)** | **American Samoa** | **Both** | **Age-standardized** | **Gout** | **Kidney dysfunction** | **Rate** | **2021** | **3.546701439** | **5.18899466** | **2.21877062** |
| **DALYs (Disability-Adjusted Life Years)** | **Brunei Darussalam** | **Both** | **Age-standardized** | **Gout** | **Kidney dysfunction** | **Rate** | **2021** | **3.545459563** | **5.39013224** | **2.22873827** |
| **YLDs (Years Lived with Disability)** | **Brunei Darussalam** | **Both** | **Age-standardized** | **Gout** | **Kidney dysfunction** | **Rate** | **2021** | **3.545459563** | **5.39013224** | **2.22873827** |
| **DALYs (Disability-Adjusted Life Years)** | **Palau** | **Both** | **Age-standardized** | **Gout** | **Kidney dysfunction** | **Rate** | **2021** | **3.36958955** | **4.97296265** | **2.10020295** |
| **YLDs (Years Lived with Disability)** | **Palau** | **Both** | **Age-standardized** | **Gout** | **Kidney dysfunction** | **Rate** | **2021** | **3.36958955** | **4.97296265** | **2.10020295** |
| **DALYs (Disability-Adjusted Life Years)** | **Singapore** | **Both** | **Age-standardized** | **Gout** | **Kidney dysfunction** | **Rate** | **2021** | **3.147264308** | **4.78912503** | **1.97761264** |
| **YLDs (Years Lived with Disability)** | **Singapore** | **Both** | **Age-standardized** | **Gout** | **Kidney dysfunction** | **Rate** | **2021** | **3.147264308** | **4.78912503** | **1.97761264** |
| **DALYs (Disability-Adjusted Life Years)** | **Japan** | **Both** | **Age-standardized** | **Gout** | **Kidney dysfunction** | **Rate** | **2021** | **3.13131039** | **4.7226476** | **1.91060739** |
| **YLDs (Years Lived with Disability)** | **Japan** | **Both** | **Age-standardized** | **Gout** | **Kidney dysfunction** | **Rate** | **2021** | **3.13131039** | **4.7226476** | **1.91060739** |
| **DALYs (Disability-Adjusted Life Years)** | **Thailand** | **Both** | **Age-standardized** | **Gout** | **Kidney dysfunction** | **Rate** | **2021** | **3.090560788** | **4.62825701** | **1.94262504** |
| **YLDs (Years Lived with Disability)** | **Thailand** | **Both** | **Age-standardized** | **Gout** | **Kidney dysfunction** | **Rate** | **2021** | **3.090560788** | **4.62825701** | **1.94262504** |
| **DALYs (Disability-Adjusted Life Years)** | **Micronesia (Federated States of)** | **Both** | **Age-standardized** | **Gout** | **Kidney dysfunction** | **Rate** | **2021** | **3.033343818** | **4.48474773** | **1.89218485** |
| **YLDs (Years Lived with Disability)** | **Micronesia (Federated States of)** | **Both** | **Age-standardized** | **Gout** | **Kidney dysfunction** | **Rate** | **2021** | **3.033343818** | **4.48474773** | **1.89218485** |
| **DALYs (Disability-Adjusted Life Years)** | **Niue** | **Both** | **Age-standardized** | **Gout** | **Kidney dysfunction** | **Rate** | **2021** | **2.998669013** | **4.55309695** | **1.86488795** |
| **YLDs (Years Lived with Disability)** | **Niue** | **Both** | **Age-standardized** | **Gout** | **Kidney dysfunction** | **Rate** | **2021** | **2.998669013** | **4.55309695** | **1.86488795** |
| **DALYs (Disability-Adjusted Life Years)** | **Cameroon** | **Both** | **Age-standardized** | **Gout** | **Kidney dysfunction** | **Rate** | **2021** | **2.992404987** | **4.5863573** | **1.89415047** |
| **YLDs (Years Lived with Disability)** | **Cameroon** | **Both** | **Age-standardized** | **Gout** | **Kidney dysfunction** | **Rate** | **2021** | **2.992404987** | **4.5863573** | **1.89415047** |
| **DALYs (Disability-Adjusted Life Years)** | **Mauritius** | **Both** | **Age-standardized** | **Gout** | **Kidney dysfunction** | **Rate** | **2021** | **2.990834969** | **4.53781664** | **1.91946252** |
| **YLDs (Years Lived with Disability)** | **Mauritius** | **Both** | **Age-standardized** | **Gout** | **Kidney dysfunction** | **Rate** | **2021** | **2.990834969** | **4.53781664** | **1.91946252** |
| **DALYs (Disability-Adjusted Life Years)** | **Samoa** | **Both** | **Age-standardized** | **Gout** | **Kidney dysfunction** | **Rate** | **2021** | **2.982173684** | **4.48366585** | **1.87491036** |
| **YLDs (Years Lived with Disability)** | **Samoa** | **Both** | **Age-standardized** | **Gout** | **Kidney dysfunction** | **Rate** | **2021** | **2.982173684** | **4.48366585** | **1.87491036** |
| **DALYs (Disability-Adjusted Life Years)** | **Tonga** | **Both** | **Age-standardized** | **Gout** | **Kidney dysfunction** | **Rate** | **2021** | **2.966513399** | **4.46780128** | **1.86884231** |
| **YLDs (Years Lived with Disability)** | **Tonga** | **Both** | **Age-standardized** | **Gout** | **Kidney dysfunction** | **Rate** | **2021** | **2.966513399** | **4.46780128** | **1.86884231** |
| **DALYs (Disability-Adjusted Life Years)** | **United Arab Emirates** | **Both** | **Age-standardized** | **Gout** | **Kidney dysfunction** | **Rate** | **2021** | **2.958066703** | **4.6031071** | **1.81760144** |
| **YLDs (Years Lived with Disability)** | **United Arab Emirates** | **Both** | **Age-standardized** | **Gout** | **Kidney dysfunction** | **Rate** | **2021** | **2.958066703** | **4.6031071** | **1.81760144** |
| **DALYs (Disability-Adjusted Life Years)** | **Cook Islands** | **Both** | **Age-standardized** | **Gout** | **Kidney dysfunction** | **Rate** | **2021** | **2.944044138** | **4.45179451** | **1.84400822** |
| **YLDs (Years Lived with Disability)** | **Cook Islands** | **Both** | **Age-standardized** | **Gout** | **Kidney dysfunction** | **Rate** | **2021** | **2.944044138** | **4.45179451** | **1.84400822** |
| **DALYs (Disability-Adjusted Life Years)** | **Malaysia** | **Both** | **Age-standardized** | **Gout** | **Kidney dysfunction** | **Rate** | **2021** | **2.918595581** | **4.35424898** | **1.79768229** |
| **YLDs (Years Lived with Disability)** | **Malaysia** | **Both** | **Age-standardized** | **Gout** | **Kidney dysfunction** | **Rate** | **2021** | **2.918595581** | **4.35424898** | **1.79768229** |
| **DALYs (Disability-Adjusted Life Years)** | **Gabon** | **Both** | **Age-standardized** | **Gout** | **Kidney dysfunction** | **Rate** | **2021** | **2.916796397** | **4.38281143** | **1.86583096** |
| **YLDs (Years Lived with Disability)** | **Gabon** | **Both** | **Age-standardized** | **Gout** | **Kidney dysfunction** | **Rate** | **2021** | **2.916796397** | **4.38281143** | **1.86583096** |
| **DALYs (Disability-Adjusted Life Years)** | **Nauru** | **Both** | **Age-standardized** | **Gout** | **Kidney dysfunction** | **Rate** | **2021** | **2.908946451** | **4.30595667** | **1.81956515** |
| **YLDs (Years Lived with Disability)** | **Nauru** | **Both** | **Age-standardized** | **Gout** | **Kidney dysfunction** | **Rate** | **2021** | **2.908946451** | **4.30595667** | **1.81956515** |
| **DALYs (Disability-Adjusted Life Years)** | **South Africa** | **Both** | **Age-standardized** | **Gout** | **Kidney dysfunction** | **Rate** | **2021** | **2.900674511** | **4.31303015** | **1.82611739** |
| **YLDs (Years Lived with Disability)** | **South Africa** | **Both** | **Age-standardized** | **Gout** | **Kidney dysfunction** | **Rate** | **2021** | **2.900674511** | **4.31303015** | **1.82611739** |
| **DALYs (Disability-Adjusted Life Years)** | **Fiji** | **Both** | **Age-standardized** | **Gout** | **Kidney dysfunction** | **Rate** | **2021** | **2.889051474** | **4.31430492** | **1.81061555** |
| **YLDs (Years Lived with Disability)** | **Fiji** | **Both** | **Age-standardized** | **Gout** | **Kidney dysfunction** | **Rate** | **2021** | **2.889051474** | **4.31430492** | **1.81061555** |
| **DALYs (Disability-Adjusted Life Years)** | **Guam** | **Both** | **Age-standardized** | **Gout** | **Kidney dysfunction** | **Rate** | **2021** | **2.835749621** | **4.25344553** | **1.77478537** |
| **YLDs (Years Lived with Disability)** | **Guam** | **Both** | **Age-standardized** | **Gout** | **Kidney dysfunction** | **Rate** | **2021** | **2.835749621** | **4.25344553** | **1.77478537** |
| **DALYs (Disability-Adjusted Life Years)** | **Ireland** | **Both** | **Age-standardized** | **Gout** | **Kidney dysfunction** | **Rate** | **2021** | **2.799051166** | **4.25595943** | **1.78281888** |
| **YLDs (Years Lived with Disability)** | **Ireland** | **Both** | **Age-standardized** | **Gout** | **Kidney dysfunction** | **Rate** | **2021** | **2.799051166** | **4.25595943** | **1.78281888** |
| **DALYs (Disability-Adjusted Life Years)** | **Marshall Islands** | **Both** | **Age-standardized** | **Gout** | **Kidney dysfunction** | **Rate** | **2021** | **2.757139445** | **4.14809761** | **1.72470032** |
| **YLDs (Years Lived with Disability)** | **Marshall Islands** | **Both** | **Age-standardized** | **Gout** | **Kidney dysfunction** | **Rate** | **2021** | **2.757139445** | **4.14809761** | **1.72470032** |
| **DALYs (Disability-Adjusted Life Years)** | **Congo** | **Both** | **Age-standardized** | **Gout** | **Kidney dysfunction** | **Rate** | **2021** | **2.756268661** | **4.16473212** | **1.72015191** |
| **YLDs (Years Lived with Disability)** | **Congo** | **Both** | **Age-standardized** | **Gout** | **Kidney dysfunction** | **Rate** | **2021** | **2.756268661** | **4.16473212** | **1.72015191** |
| **DALYs (Disability-Adjusted Life Years)** | **Uruguay** | **Both** | **Age-standardized** | **Gout** | **Kidney dysfunction** | **Rate** | **2021** | **2.751377811** | **4.21477032** | **1.7088649** |
| **YLDs (Years Lived with Disability)** | **Uruguay** | **Both** | **Age-standardized** | **Gout** | **Kidney dysfunction** | **Rate** | **2021** | **2.751377811** | **4.21477032** | **1.7088649** |
| **DALYs (Disability-Adjusted Life Years)** | **Eswatini** | **Both** | **Age-standardized** | **Gout** | **Kidney dysfunction** | **Rate** | **2021** | **2.751074451** | **4.09175892** | **1.74415947** |
| **YLDs (Years Lived with Disability)** | **Eswatini** | **Both** | **Age-standardized** | **Gout** | **Kidney dysfunction** | **Rate** | **2021** | **2.751074451** | **4.09175892** | **1.74415947** |
| **DALYs (Disability-Adjusted Life Years)** | **Equatorial Guinea** | **Both** | **Age-standardized** | **Gout** | **Kidney dysfunction** | **Rate** | **2021** | **2.747904376** | **4.21692599** | **1.73196314** |
| **YLDs (Years Lived with Disability)** | **Equatorial Guinea** | **Both** | **Age-standardized** | **Gout** | **Kidney dysfunction** | **Rate** | **2021** | **2.747904376** | **4.21692599** | **1.73196314** |
| **DALYs (Disability-Adjusted Life Years)** | **Tokelau** | **Both** | **Age-standardized** | **Gout** | **Kidney dysfunction** | **Rate** | **2021** | **2.741164636** | **4.11548313** | **1.73764116** |
| **YLDs (Years Lived with Disability)** | **Tokelau** | **Both** | **Age-standardized** | **Gout** | **Kidney dysfunction** | **Rate** | **2021** | **2.741164636** | **4.11548313** | **1.73764116** |
| **DALYs (Disability-Adjusted Life Years)** | **Chile** | **Both** | **Age-standardized** | **Gout** | **Kidney dysfunction** | **Rate** | **2021** | **2.729557414** | **4.1648413** | **1.69086904** |
| **YLDs (Years Lived with Disability)** | **Chile** | **Both** | **Age-standardized** | **Gout** | **Kidney dysfunction** | **Rate** | **2021** | **2.729557414** | **4.1648413** | **1.69086904** |
| **DALYs (Disability-Adjusted Life Years)** | **Tuvalu** | **Both** | **Age-standardized** | **Gout** | **Kidney dysfunction** | **Rate** | **2021** | **2.710378566** | **4.01426136** | **1.68844058** |
| **YLDs (Years Lived with Disability)** | **Tuvalu** | **Both** | **Age-standardized** | **Gout** | **Kidney dysfunction** | **Rate** | **2021** | **2.710378566** | **4.01426136** | **1.68844058** |
| **DALYs (Disability-Adjusted Life Years)** | **Nepal** | **Both** | **Age-standardized** | **Gout** | **Kidney dysfunction** | **Rate** | **2021** | **2.693533498** | **4.00778894** | **1.71292674** |
| **YLDs (Years Lived with Disability)** | **Nepal** | **Both** | **Age-standardized** | **Gout** | **Kidney dysfunction** | **Rate** | **2021** | **2.693533498** | **4.00778894** | **1.71292674** |
| **DALYs (Disability-Adjusted Life Years)** | **Turkmenistan** | **Both** | **Age-standardized** | **Gout** | **Kidney dysfunction** | **Rate** | **2021** | **2.678111292** | **3.99695576** | **1.6912155** |
| **YLDs (Years Lived with Disability)** | **Turkmenistan** | **Both** | **Age-standardized** | **Gout** | **Kidney dysfunction** | **Rate** | **2021** | **2.678111292** | **3.99695576** | **1.6912155** |
| **DALYs (Disability-Adjusted Life Years)** | **Seychelles** | **Both** | **Age-standardized** | **Gout** | **Kidney dysfunction** | **Rate** | **2021** | **2.676811637** | **3.99550947** | **1.67076015** |
| **YLDs (Years Lived with Disability)** | **Seychelles** | **Both** | **Age-standardized** | **Gout** | **Kidney dysfunction** | **Rate** | **2021** | **2.676811637** | **3.99550947** | **1.67076015** |
| **DALYs (Disability-Adjusted Life Years)** | **Vanuatu** | **Both** | **Age-standardized** | **Gout** | **Kidney dysfunction** | **Rate** | **2021** | **2.655828423** | **3.91625686** | **1.61618339** |
| **YLDs (Years Lived with Disability)** | **Vanuatu** | **Both** | **Age-standardized** | **Gout** | **Kidney dysfunction** | **Rate** | **2021** | **2.655828423** | **3.91625686** | **1.61618339** |
| **DALYs (Disability-Adjusted Life Years)** | **Uzbekistan** | **Both** | **Age-standardized** | **Gout** | **Kidney dysfunction** | **Rate** | **2021** | **2.643756898** | **4.01730272** | **1.65141738** |
| **YLDs (Years Lived with Disability)** | **Uzbekistan** | **Both** | **Age-standardized** | **Gout** | **Kidney dysfunction** | **Rate** | **2021** | **2.643756898** | **4.01730272** | **1.65141738** |
| **DALYs (Disability-Adjusted Life Years)** | **Botswana** | **Both** | **Age-standardized** | **Gout** | **Kidney dysfunction** | **Rate** | **2021** | **2.643250102** | **3.9351367** | **1.66194959** |
| **YLDs (Years Lived with Disability)** | **Botswana** | **Both** | **Age-standardized** | **Gout** | **Kidney dysfunction** | **Rate** | **2021** | **2.643250102** | **3.9351367** | **1.66194959** |
| **DALYs (Disability-Adjusted Life Years)** | **United Kingdom** | **Both** | **Age-standardized** | **Gout** | **Kidney dysfunction** | **Rate** | **2021** | **2.638979212** | **3.96521761** | **1.63550014** |
| **YLDs (Years Lived with Disability)** | **United Kingdom** | **Both** | **Age-standardized** | **Gout** | **Kidney dysfunction** | **Rate** | **2021** | **2.638979212** | **3.96521761** | **1.63550014** |
| **DALYs (Disability-Adjusted Life Years)** | **Israel** | **Both** | **Age-standardized** | **Gout** | **Kidney dysfunction** | **Rate** | **2021** | **2.587793967** | **3.94933725** | **1.63621947** |
| **YLDs (Years Lived with Disability)** | **Israel** | **Both** | **Age-standardized** | **Gout** | **Kidney dysfunction** | **Rate** | **2021** | **2.587793967** | **3.94933725** | **1.63621947** |
| **DALYs (Disability-Adjusted Life Years)** | **Azerbaijan** | **Both** | **Age-standardized** | **Gout** | **Kidney dysfunction** | **Rate** | **2021** | **2.585202117** | **3.91432695** | **1.60299847** |
| **YLDs (Years Lived with Disability)** | **Azerbaijan** | **Both** | **Age-standardized** | **Gout** | **Kidney dysfunction** | **Rate** | **2021** | **2.585202117** | **3.91432695** | **1.60299847** |
| **DALYs (Disability-Adjusted Life Years)** | **Solomon Islands** | **Both** | **Age-standardized** | **Gout** | **Kidney dysfunction** | **Rate** | **2021** | **2.550550884** | **3.77681496** | **1.57132168** |
| **YLDs (Years Lived with Disability)** | **Solomon Islands** | **Both** | **Age-standardized** | **Gout** | **Kidney dysfunction** | **Rate** | **2021** | **2.550550884** | **3.77681496** | **1.57132168** |
| **DALYs (Disability-Adjusted Life Years)** | **Kiribati** | **Both** | **Age-standardized** | **Gout** | **Kidney dysfunction** | **Rate** | **2021** | **2.532207844** | **3.79463792** | **1.61299153** |
| **YLDs (Years Lived with Disability)** | **Kiribati** | **Both** | **Age-standardized** | **Gout** | **Kidney dysfunction** | **Rate** | **2021** | **2.532207844** | **3.79463792** | **1.61299153** |
| **DALYs (Disability-Adjusted Life Years)** | **Greece** | **Both** | **Age-standardized** | **Gout** | **Kidney dysfunction** | **Rate** | **2021** | **2.523079762** | **3.83067061** | **1.58621844** |
| **YLDs (Years Lived with Disability)** | **Greece** | **Both** | **Age-standardized** | **Gout** | **Kidney dysfunction** | **Rate** | **2021** | **2.523079762** | **3.83067061** | **1.58621844** |
| **DALYs (Disability-Adjusted Life Years)** | **Maldives** | **Both** | **Age-standardized** | **Gout** | **Kidney dysfunction** | **Rate** | **2021** | **2.516775385** | **3.77660693** | **1.55754985** |
| **YLDs (Years Lived with Disability)** | **Maldives** | **Both** | **Age-standardized** | **Gout** | **Kidney dysfunction** | **Rate** | **2021** | **2.516775385** | **3.77660693** | **1.55754985** |
| **DALYs (Disability-Adjusted Life Years)** | **Republic of Korea** | **Both** | **Age-standardized** | **Gout** | **Kidney dysfunction** | **Rate** | **2021** | **2.503062811** | **3.72250885** | **1.55880848** |
| **YLDs (Years Lived with Disability)** | **Republic of Korea** | **Both** | **Age-standardized** | **Gout** | **Kidney dysfunction** | **Rate** | **2021** | **2.503062811** | **3.72250885** | **1.55880848** |
| **DALYs (Disability-Adjusted Life Years)** | **Saudi Arabia** | **Both** | **Age-standardized** | **Gout** | **Kidney dysfunction** | **Rate** | **2021** | **2.493069738** | **3.62474014** | **1.55016664** |
| **YLDs (Years Lived with Disability)** | **Saudi Arabia** | **Both** | **Age-standardized** | **Gout** | **Kidney dysfunction** | **Rate** | **2021** | **2.493069738** | **3.62474014** | **1.55016664** |
| **DALYs (Disability-Adjusted Life Years)** | **Germany** | **Both** | **Age-standardized** | **Gout** | **Kidney dysfunction** | **Rate** | **2021** | **2.477624079** | **3.81004066** | **1.48510284** |
| **YLDs (Years Lived with Disability)** | **Germany** | **Both** | **Age-standardized** | **Gout** | **Kidney dysfunction** | **Rate** | **2021** | **2.477624079** | **3.81004066** | **1.48510284** |
| **DALYs (Disability-Adjusted Life Years)** | **Armenia** | **Both** | **Age-standardized** | **Gout** | **Kidney dysfunction** | **Rate** | **2021** | **2.452232976** | **3.68180616** | **1.5685506** |
| **YLDs (Years Lived with Disability)** | **Armenia** | **Both** | **Age-standardized** | **Gout** | **Kidney dysfunction** | **Rate** | **2021** | **2.452232976** | **3.68180616** | **1.5685506** |
| **DALYs (Disability-Adjusted Life Years)** | **Democratic Republic of the Congo** | **Both** | **Age-standardized** | **Gout** | **Kidney dysfunction** | **Rate** | **2021** | **2.439870615** | **3.62455603** | **1.5393152** |
| **YLDs (Years Lived with Disability)** | **Democratic Republic of the Congo** | **Both** | **Age-standardized** | **Gout** | **Kidney dysfunction** | **Rate** | **2021** | **2.439870615** | **3.62455603** | **1.5393152** |
| **DALYs (Disability-Adjusted Life Years)** | **Kazakhstan** | **Both** | **Age-standardized** | **Gout** | **Kidney dysfunction** | **Rate** | **2021** | **2.438122502** | **3.63390168** | **1.51564791** |
| **YLDs (Years Lived with Disability)** | **Kazakhstan** | **Both** | **Age-standardized** | **Gout** | **Kidney dysfunction** | **Rate** | **2021** | **2.438122502** | **3.63390168** | **1.51564791** |
| **DALYs (Disability-Adjusted Life Years)** | **Philippines** | **Both** | **Age-standardized** | **Gout** | **Kidney dysfunction** | **Rate** | **2021** | **2.414471764** | **3.59624025** | **1.51546136** |
| **YLDs (Years Lived with Disability)** | **Philippines** | **Both** | **Age-standardized** | **Gout** | **Kidney dysfunction** | **Rate** | **2021** | **2.414471764** | **3.59624025** | **1.51546136** |
| **DALYs (Disability-Adjusted Life Years)** | **Lao People's Democratic Republic** | **Both** | **Age-standardized** | **Gout** | **Kidney dysfunction** | **Rate** | **2021** | **2.402216125** | **3.62553622** | **1.52094619** |
| **YLDs (Years Lived with Disability)** | **Lao People's Democratic Republic** | **Both** | **Age-standardized** | **Gout** | **Kidney dysfunction** | **Rate** | **2021** | **2.402216125** | **3.62553622** | **1.52094619** |
| **DALYs (Disability-Adjusted Life Years)** | **Namibia** | **Both** | **Age-standardized** | **Gout** | **Kidney dysfunction** | **Rate** | **2021** | **2.398511939** | **3.52011356** | **1.49295189** |
| **YLDs (Years Lived with Disability)** | **Namibia** | **Both** | **Age-standardized** | **Gout** | **Kidney dysfunction** | **Rate** | **2021** | **2.398511939** | **3.52011356** | **1.49295189** |
| **DALYs (Disability-Adjusted Life Years)** | **Mongolia** | **Both** | **Age-standardized** | **Gout** | **Kidney dysfunction** | **Rate** | **2021** | **2.379830087** | **3.49468579** | **1.48599794** |
| **YLDs (Years Lived with Disability)** | **Mongolia** | **Both** | **Age-standardized** | **Gout** | **Kidney dysfunction** | **Rate** | **2021** | **2.379830087** | **3.49468579** | **1.48599794** |
| **DALYs (Disability-Adjusted Life Years)** | **Argentina** | **Both** | **Age-standardized** | **Gout** | **Kidney dysfunction** | **Rate** | **2021** | **2.377501553** | **3.61028587** | **1.4812028** |
| **YLDs (Years Lived with Disability)** | **Argentina** | **Both** | **Age-standardized** | **Gout** | **Kidney dysfunction** | **Rate** | **2021** | **2.377501553** | **3.61028587** | **1.4812028** |
| **DALYs (Disability-Adjusted Life Years)** | **Lesotho** | **Both** | **Age-standardized** | **Gout** | **Kidney dysfunction** | **Rate** | **2021** | **2.37131819** | **3.53704122** | **1.48296133** |
| **YLDs (Years Lived with Disability)** | **Lesotho** | **Both** | **Age-standardized** | **Gout** | **Kidney dysfunction** | **Rate** | **2021** | **2.37131819** | **3.53704122** | **1.48296133** |
| **DALYs (Disability-Adjusted Life Years)** | **Zimbabwe** | **Both** | **Age-standardized** | **Gout** | **Kidney dysfunction** | **Rate** | **2021** | **2.364761738** | **3.55751404** | **1.44361667** |
| **YLDs (Years Lived with Disability)** | **Zimbabwe** | **Both** | **Age-standardized** | **Gout** | **Kidney dysfunction** | **Rate** | **2021** | **2.364761738** | **3.55751404** | **1.44361667** |
| **DALYs (Disability-Adjusted Life Years)** | **Andorra** | **Both** | **Age-standardized** | **Gout** | **Kidney dysfunction** | **Rate** | **2021** | **2.364525273** | **3.69655147** | **1.46880973** |
| **YLDs (Years Lived with Disability)** | **Andorra** | **Both** | **Age-standardized** | **Gout** | **Kidney dysfunction** | **Rate** | **2021** | **2.364525273** | **3.69655147** | **1.46880973** |
| **DALYs (Disability-Adjusted Life Years)** | **Georgia** | **Both** | **Age-standardized** | **Gout** | **Kidney dysfunction** | **Rate** | **2021** | **2.34650834** | **3.52488824** | **1.48447946** |
| **YLDs (Years Lived with Disability)** | **Georgia** | **Both** | **Age-standardized** | **Gout** | **Kidney dysfunction** | **Rate** | **2021** | **2.34650834** | **3.52488824** | **1.48447946** |
| **DALYs (Disability-Adjusted Life Years)** | **Angola** | **Both** | **Age-standardized** | **Gout** | **Kidney dysfunction** | **Rate** | **2021** | **2.345580269** | **3.50145643** | **1.46941092** |
| **YLDs (Years Lived with Disability)** | **Angola** | **Both** | **Age-standardized** | **Gout** | **Kidney dysfunction** | **Rate** | **2021** | **2.345580269** | **3.50145643** | **1.46941092** |
| **DALYs (Disability-Adjusted Life Years)** | **Cyprus** | **Both** | **Age-standardized** | **Gout** | **Kidney dysfunction** | **Rate** | **2021** | **2.345579788** | **3.61330879** | **1.49769493** |
| **YLDs (Years Lived with Disability)** | **Cyprus** | **Both** | **Age-standardized** | **Gout** | **Kidney dysfunction** | **Rate** | **2021** | **2.345579788** | **3.61330879** | **1.49769493** |
| **DALYs (Disability-Adjusted Life Years)** | **Qatar** | **Both** | **Age-standardized** | **Gout** | **Kidney dysfunction** | **Rate** | **2021** | **2.339259377** | **3.47184844** | **1.43469402** |
| **YLDs (Years Lived with Disability)** | **Qatar** | **Both** | **Age-standardized** | **Gout** | **Kidney dysfunction** | **Rate** | **2021** | **2.339259377** | **3.47184844** | **1.43469402** |
| **DALYs (Disability-Adjusted Life Years)** | **Monaco** | **Both** | **Age-standardized** | **Gout** | **Kidney dysfunction** | **Rate** | **2021** | **2.337912394** | **3.60074112** | **1.43674003** |
| **YLDs (Years Lived with Disability)** | **Monaco** | **Both** | **Age-standardized** | **Gout** | **Kidney dysfunction** | **Rate** | **2021** | **2.337912394** | **3.60074112** | **1.43674003** |
| **DALYs (Disability-Adjusted Life Years)** | **Indonesia** | **Both** | **Age-standardized** | **Gout** | **Kidney dysfunction** | **Rate** | **2021** | **2.326474987** | **3.47077431** | **1.44570062** |
| **YLDs (Years Lived with Disability)** | **Indonesia** | **Both** | **Age-standardized** | **Gout** | **Kidney dysfunction** | **Rate** | **2021** | **2.326474987** | **3.47077431** | **1.44570062** |
| **DALYs (Disability-Adjusted Life Years)** | **Nigeria** | **Both** | **Age-standardized** | **Gout** | **Kidney dysfunction** | **Rate** | **2021** | **2.32212736** | **3.46254046** | **1.47258401** |
| **YLDs (Years Lived with Disability)** | **Nigeria** | **Both** | **Age-standardized** | **Gout** | **Kidney dysfunction** | **Rate** | **2021** | **2.32212736** | **3.46254046** | **1.47258401** |
| **DALYs (Disability-Adjusted Life Years)** | **Democratic People's Republic of Korea** | **Both** | **Age-standardized** | **Gout** | **Kidney dysfunction** | **Rate** | **2021** | **2.319789941** | **3.48949315** | **1.42093514** |
| **YLDs (Years Lived with Disability)** | **Democratic People's Republic of Korea** | **Both** | **Age-standardized** | **Gout** | **Kidney dysfunction** | **Rate** | **2021** | **2.319789941** | **3.48949315** | **1.42093514** |
| **DALYs (Disability-Adjusted Life Years)** | **Malta** | **Both** | **Age-standardized** | **Gout** | **Kidney dysfunction** | **Rate** | **2021** | **2.318486835** | **3.50446563** | **1.42785812** |
| **YLDs (Years Lived with Disability)** | **Malta** | **Both** | **Age-standardized** | **Gout** | **Kidney dysfunction** | **Rate** | **2021** | **2.318486835** | **3.50446563** | **1.42785812** |
| **DALYs (Disability-Adjusted Life Years)** | **Iran (Islamic Republic of)** | **Both** | **Age-standardized** | **Gout** | **Kidney dysfunction** | **Rate** | **2021** | **2.303386689** | **3.47691198** | **1.43752151** |
| **YLDs (Years Lived with Disability)** | **Iran (Islamic Republic of)** | **Both** | **Age-standardized** | **Gout** | **Kidney dysfunction** | **Rate** | **2021** | **2.303386689** | **3.47691198** | **1.43752151** |
| **DALYs (Disability-Adjusted Life Years)** | **Kyrgyzstan** | **Both** | **Age-standardized** | **Gout** | **Kidney dysfunction** | **Rate** | **2021** | **2.302622535** | **3.44616856** | **1.44778031** |
| **YLDs (Years Lived with Disability)** | **Kyrgyzstan** | **Both** | **Age-standardized** | **Gout** | **Kidney dysfunction** | **Rate** | **2021** | **2.302622535** | **3.44616856** | **1.44778031** |
| **DALYs (Disability-Adjusted Life Years)** | **Netherlands** | **Both** | **Age-standardized** | **Gout** | **Kidney dysfunction** | **Rate** | **2021** | **2.28376991** | **3.49621651** | **1.43063179** |
| **YLDs (Years Lived with Disability)** | **Netherlands** | **Both** | **Age-standardized** | **Gout** | **Kidney dysfunction** | **Rate** | **2021** | **2.28376991** | **3.49621651** | **1.43063179** |
| **DALYs (Disability-Adjusted Life Years)** | **Austria** | **Both** | **Age-standardized** | **Gout** | **Kidney dysfunction** | **Rate** | **2021** | **2.271587084** | **3.43408018** | **1.39522313** |
| **YLDs (Years Lived with Disability)** | **Austria** | **Both** | **Age-standardized** | **Gout** | **Kidney dysfunction** | **Rate** | **2021** | **2.271587084** | **3.43408018** | **1.39522313** |
| **DALYs (Disability-Adjusted Life Years)** | **Denmark** | **Both** | **Age-standardized** | **Gout** | **Kidney dysfunction** | **Rate** | **2021** | **2.270236639** | **3.54173637** | **1.39393178** |
| **YLDs (Years Lived with Disability)** | **Denmark** | **Both** | **Age-standardized** | **Gout** | **Kidney dysfunction** | **Rate** | **2021** | **2.270236639** | **3.54173637** | **1.39393178** |
| **DALYs (Disability-Adjusted Life Years)** | **Switzerland** | **Both** | **Age-standardized** | **Gout** | **Kidney dysfunction** | **Rate** | **2021** | **2.257869722** | **3.40425446** | **1.42651562** |
| **YLDs (Years Lived with Disability)** | **Switzerland** | **Both** | **Age-standardized** | **Gout** | **Kidney dysfunction** | **Rate** | **2021** | **2.257869722** | **3.40425446** | **1.42651562** |
| **DALYs (Disability-Adjusted Life Years)** | **San Marino** | **Both** | **Age-standardized** | **Gout** | **Kidney dysfunction** | **Rate** | **2021** | **2.226031509** | **3.40941459** | **1.38750218** |
| **YLDs (Years Lived with Disability)** | **San Marino** | **Both** | **Age-standardized** | **Gout** | **Kidney dysfunction** | **Rate** | **2021** | **2.226031509** | **3.40941459** | **1.38750218** |
| **DALYs (Disability-Adjusted Life Years)** | **Central African Republic** | **Both** | **Age-standardized** | **Gout** | **Kidney dysfunction** | **Rate** | **2021** | **2.219781322** | **3.33171441** | **1.37832113** |
| **YLDs (Years Lived with Disability)** | **Central African Republic** | **Both** | **Age-standardized** | **Gout** | **Kidney dysfunction** | **Rate** | **2021** | **2.219781322** | **3.33171441** | **1.37832113** |
| **DALYs (Disability-Adjusted Life Years)** | **Belgium** | **Both** | **Age-standardized** | **Gout** | **Kidney dysfunction** | **Rate** | **2021** | **2.21778697** | **3.33064629** | **1.37843624** |
| **YLDs (Years Lived with Disability)** | **Belgium** | **Both** | **Age-standardized** | **Gout** | **Kidney dysfunction** | **Rate** | **2021** | **2.21778697** | **3.33064629** | **1.37843624** |
| **DALYs (Disability-Adjusted Life Years)** | **Papua New Guinea** | **Both** | **Age-standardized** | **Gout** | **Kidney dysfunction** | **Rate** | **2021** | **2.216709708** | **3.32303523** | **1.35148053** |
| **YLDs (Years Lived with Disability)** | **Papua New Guinea** | **Both** | **Age-standardized** | **Gout** | **Kidney dysfunction** | **Rate** | **2021** | **2.216709708** | **3.32303523** | **1.35148053** |
| **DALYs (Disability-Adjusted Life Years)** | **Egypt** | **Both** | **Age-standardized** | **Gout** | **Kidney dysfunction** | **Rate** | **2021** | **2.207639981** | **3.36384412** | **1.35260835** |
| **YLDs (Years Lived with Disability)** | **Egypt** | **Both** | **Age-standardized** | **Gout** | **Kidney dysfunction** | **Rate** | **2021** | **2.207639981** | **3.36384412** | **1.35260835** |
| **DALYs (Disability-Adjusted Life Years)** | **Sri Lanka** | **Both** | **Age-standardized** | **Gout** | **Kidney dysfunction** | **Rate** | **2021** | **2.198296821** | **3.32264176** | **1.37240793** |
| **YLDs (Years Lived with Disability)** | **Sri Lanka** | **Both** | **Age-standardized** | **Gout** | **Kidney dysfunction** | **Rate** | **2021** | **2.198296821** | **3.32264176** | **1.37240793** |
| **DALYs (Disability-Adjusted Life Years)** | **Luxembourg** | **Both** | **Age-standardized** | **Gout** | **Kidney dysfunction** | **Rate** | **2021** | **2.172212798** | **3.42687298** | **1.31948525** |
| **YLDs (Years Lived with Disability)** | **Luxembourg** | **Both** | **Age-standardized** | **Gout** | **Kidney dysfunction** | **Rate** | **2021** | **2.172212798** | **3.42687298** | **1.31948525** |
| **DALYs (Disability-Adjusted Life Years)** | **Spain** | **Both** | **Age-standardized** | **Gout** | **Kidney dysfunction** | **Rate** | **2021** | **2.16206428** | **3.35135003** | **1.36122016** |
| **YLDs (Years Lived with Disability)** | **Spain** | **Both** | **Age-standardized** | **Gout** | **Kidney dysfunction** | **Rate** | **2021** | **2.16206428** | **3.35135003** | **1.36122016** |
| **DALYs (Disability-Adjusted Life Years)** | **Timor-Leste** | **Both** | **Age-standardized** | **Gout** | **Kidney dysfunction** | **Rate** | **2021** | **2.143737754** | **3.23102569** | **1.35666773** |
| **YLDs (Years Lived with Disability)** | **Timor-Leste** | **Both** | **Age-standardized** | **Gout** | **Kidney dysfunction** | **Rate** | **2021** | **2.143737754** | **3.23102569** | **1.35666773** |
| **DALYs (Disability-Adjusted Life Years)** | **Republic of Moldova** | **Both** | **Age-standardized** | **Gout** | **Kidney dysfunction** | **Rate** | **2021** | **2.1247512** | **3.1975358** | **1.33582446** |
| **YLDs (Years Lived with Disability)** | **Republic of Moldova** | **Both** | **Age-standardized** | **Gout** | **Kidney dysfunction** | **Rate** | **2021** | **2.1247512** | **3.1975358** | **1.33582446** |
| **DALYs (Disability-Adjusted Life Years)** | **Tajikistan** | **Both** | **Age-standardized** | **Gout** | **Kidney dysfunction** | **Rate** | **2021** | **2.118846668** | **3.21626905** | **1.33930859** |
| **YLDs (Years Lived with Disability)** | **Tajikistan** | **Both** | **Age-standardized** | **Gout** | **Kidney dysfunction** | **Rate** | **2021** | **2.118846668** | **3.21626905** | **1.33930859** |
| **DALYs (Disability-Adjusted Life Years)** | **Jordan** | **Both** | **Age-standardized** | **Gout** | **Kidney dysfunction** | **Rate** | **2021** | **2.11445432** | **3.17353652** | **1.30082015** |
| **YLDs (Years Lived with Disability)** | **Jordan** | **Both** | **Age-standardized** | **Gout** | **Kidney dysfunction** | **Rate** | **2021** | **2.11445432** | **3.17353652** | **1.30082015** |
| **DALYs (Disability-Adjusted Life Years)** | **Sao Tome and Principe** | **Both** | **Age-standardized** | **Gout** | **Kidney dysfunction** | **Rate** | **2021** | **2.068522623** | **3.09574917** | **1.32254399** |
| **YLDs (Years Lived with Disability)** | **Sao Tome and Principe** | **Both** | **Age-standardized** | **Gout** | **Kidney dysfunction** | **Rate** | **2021** | **2.068522623** | **3.09574917** | **1.32254399** |
| **DALYs (Disability-Adjusted Life Years)** | **Myanmar** | **Both** | **Age-standardized** | **Gout** | **Kidney dysfunction** | **Rate** | **2021** | **2.059576974** | **3.06977207** | **1.28630985** |
| **YLDs (Years Lived with Disability)** | **Myanmar** | **Both** | **Age-standardized** | **Gout** | **Kidney dysfunction** | **Rate** | **2021** | **2.059576974** | **3.06977207** | **1.28630985** |
| **DALYs (Disability-Adjusted Life Years)** | **Mauritania** | **Both** | **Age-standardized** | **Gout** | **Kidney dysfunction** | **Rate** | **2021** | **2.059502754** | **3.17557234** | **1.30681534** |
| **YLDs (Years Lived with Disability)** | **Mauritania** | **Both** | **Age-standardized** | **Gout** | **Kidney dysfunction** | **Rate** | **2021** | **2.059502754** | **3.17557234** | **1.30681534** |
| **DALYs (Disability-Adjusted Life Years)** | **Kuwait** | **Both** | **Age-standardized** | **Gout** | **Kidney dysfunction** | **Rate** | **2021** | **2.036120915** | **3.05206414** | **1.23210127** |
| **YLDs (Years Lived with Disability)** | **Kuwait** | **Both** | **Age-standardized** | **Gout** | **Kidney dysfunction** | **Rate** | **2021** | **2.036120915** | **3.05206414** | **1.23210127** |
| **DALYs (Disability-Adjusted Life Years)** | **France** | **Both** | **Age-standardized** | **Gout** | **Kidney dysfunction** | **Rate** | **2021** | **2.009962355** | **3.08172144** | **1.25071935** |
| **YLDs (Years Lived with Disability)** | **France** | **Both** | **Age-standardized** | **Gout** | **Kidney dysfunction** | **Rate** | **2021** | **2.009962355** | **3.08172144** | **1.25071935** |
| **DALYs (Disability-Adjusted Life Years)** | **Bahrain** | **Both** | **Age-standardized** | **Gout** | **Kidney dysfunction** | **Rate** | **2021** | **2.004690144** | **3.02264775** | **1.23958854** |
| **YLDs (Years Lived with Disability)** | **Bahrain** | **Both** | **Age-standardized** | **Gout** | **Kidney dysfunction** | **Rate** | **2021** | **2.004690144** | **3.02264775** | **1.23958854** |
| **DALYs (Disability-Adjusted Life Years)** | **Bhutan** | **Both** | **Age-standardized** | **Gout** | **Kidney dysfunction** | **Rate** | **2021** | **1.982734797** | **2.91569979** | **1.21176077** |
| **YLDs (Years Lived with Disability)** | **Bhutan** | **Both** | **Age-standardized** | **Gout** | **Kidney dysfunction** | **Rate** | **2021** | **1.982734797** | **2.91569979** | **1.21176077** |
| **DALYs (Disability-Adjusted Life Years)** | **Liberia** | **Both** | **Age-standardized** | **Gout** | **Kidney dysfunction** | **Rate** | **2021** | **1.965349082** | **2.95247685** | **1.2234882** |
| **YLDs (Years Lived with Disability)** | **Liberia** | **Both** | **Age-standardized** | **Gout** | **Kidney dysfunction** | **Rate** | **2021** | **1.965349082** | **2.95247685** | **1.2234882** |
| **DALYs (Disability-Adjusted Life Years)** | **China** | **Both** | **Age-standardized** | **Gout** | **Kidney dysfunction** | **Rate** | **2021** | **1.948640224** | **2.92404093** | **1.19688015** |
| **YLDs (Years Lived with Disability)** | **China** | **Both** | **Age-standardized** | **Gout** | **Kidney dysfunction** | **Rate** | **2021** | **1.948640224** | **2.92404093** | **1.19688015** |
| **DALYs (Disability-Adjusted Life Years)** | **Ivory Coast** | **Both** | **Age-standardized** | **Gout** | **Kidney dysfunction** | **Rate** | **2021** | **1.936804012** | **2.90751182** | **1.21231392** |
| **YLDs (Years Lived with Disability)** | **Ivory Coast** | **Both** | **Age-standardized** | **Gout** | **Kidney dysfunction** | **Rate** | **2021** | **1.936804012** | **2.90751182** | **1.21231392** |
| **DALYs (Disability-Adjusted Life Years)** | **Oman** | **Both** | **Age-standardized** | **Gout** | **Kidney dysfunction** | **Rate** | **2021** | **1.929584643** | **2.91201913** | **1.15413516** |
| **YLDs (Years Lived with Disability)** | **Oman** | **Both** | **Age-standardized** | **Gout** | **Kidney dysfunction** | **Rate** | **2021** | **1.929584643** | **2.91201913** | **1.15413516** |
| **DALYs (Disability-Adjusted Life Years)** | **Iceland** | **Both** | **Age-standardized** | **Gout** | **Kidney dysfunction** | **Rate** | **2021** | **1.908502581** | **2.87433109** | **1.18788977** |
| **YLDs (Years Lived with Disability)** | **Iceland** | **Both** | **Age-standardized** | **Gout** | **Kidney dysfunction** | **Rate** | **2021** | **1.908502581** | **2.87433109** | **1.18788977** |
| **DALYs (Disability-Adjusted Life Years)** | **Iraq** | **Both** | **Age-standardized** | **Gout** | **Kidney dysfunction** | **Rate** | **2021** | **1.893232498** | **2.81905325** | **1.17806154** |
| **YLDs (Years Lived with Disability)** | **Iraq** | **Both** | **Age-standardized** | **Gout** | **Kidney dysfunction** | **Rate** | **2021** | **1.893232498** | **2.81905325** | **1.17806154** |
| **DALYs (Disability-Adjusted Life Years)** | **Estonia** | **Both** | **Age-standardized** | **Gout** | **Kidney dysfunction** | **Rate** | **2021** | **1.888757367** | **2.89991836** | **1.14386216** |
| **YLDs (Years Lived with Disability)** | **Estonia** | **Both** | **Age-standardized** | **Gout** | **Kidney dysfunction** | **Rate** | **2021** | **1.888757367** | **2.89991836** | **1.14386216** |
| **DALYs (Disability-Adjusted Life Years)** | **Libya** | **Both** | **Age-standardized** | **Gout** | **Kidney dysfunction** | **Rate** | **2021** | **1.884564717** | **2.83142638** | **1.16791987** |
| **YLDs (Years Lived with Disability)** | **Libya** | **Both** | **Age-standardized** | **Gout** | **Kidney dysfunction** | **Rate** | **2021** | **1.884564717** | **2.83142638** | **1.16791987** |
| **DALYs (Disability-Adjusted Life Years)** | **Finland** | **Both** | **Age-standardized** | **Gout** | **Kidney dysfunction** | **Rate** | **2021** | **1.883664698** | **2.88633503** | **1.16971508** |
| **YLDs (Years Lived with Disability)** | **Finland** | **Both** | **Age-standardized** | **Gout** | **Kidney dysfunction** | **Rate** | **2021** | **1.883664698** | **2.88633503** | **1.16971508** |
| **DALYs (Disability-Adjusted Life Years)** | **Cambodia** | **Both** | **Age-standardized** | **Gout** | **Kidney dysfunction** | **Rate** | **2021** | **1.876391686** | **2.79235189** | **1.15293009** |
| **YLDs (Years Lived with Disability)** | **Cambodia** | **Both** | **Age-standardized** | **Gout** | **Kidney dysfunction** | **Rate** | **2021** | **1.876391686** | **2.79235189** | **1.15293009** |
| **DALYs (Disability-Adjusted Life Years)** | **Sweden** | **Both** | **Age-standardized** | **Gout** | **Kidney dysfunction** | **Rate** | **2021** | **1.87231691** | **2.84877822** | **1.14393118** |
| **YLDs (Years Lived with Disability)** | **Sweden** | **Both** | **Age-standardized** | **Gout** | **Kidney dysfunction** | **Rate** | **2021** | **1.87231691** | **2.84877822** | **1.14393118** |
| **DALYs (Disability-Adjusted Life Years)** | **Benin** | **Both** | **Age-standardized** | **Gout** | **Kidney dysfunction** | **Rate** | **2021** | **1.860655029** | **2.79603941** | **1.15591333** |
| **YLDs (Years Lived with Disability)** | **Benin** | **Both** | **Age-standardized** | **Gout** | **Kidney dysfunction** | **Rate** | **2021** | **1.860655029** | **2.79603941** | **1.15591333** |
| **DALYs (Disability-Adjusted Life Years)** | **Algeria** | **Both** | **Age-standardized** | **Gout** | **Kidney dysfunction** | **Rate** | **2021** | **1.842054995** | **2.73939447** | **1.13546352** |
| **YLDs (Years Lived with Disability)** | **Algeria** | **Both** | **Age-standardized** | **Gout** | **Kidney dysfunction** | **Rate** | **2021** | **1.842054995** | **2.73939447** | **1.13546352** |
| **DALYs (Disability-Adjusted Life Years)** | **Mali** | **Both** | **Age-standardized** | **Gout** | **Kidney dysfunction** | **Rate** | **2021** | **1.83604701** | **2.71132616** | **1.15330955** |
| **YLDs (Years Lived with Disability)** | **Mali** | **Both** | **Age-standardized** | **Gout** | **Kidney dysfunction** | **Rate** | **2021** | **1.83604701** | **2.71132616** | **1.15330955** |
| **DALYs (Disability-Adjusted Life Years)** | **Guinea** | **Both** | **Age-standardized** | **Gout** | **Kidney dysfunction** | **Rate** | **2021** | **1.83133355** | **2.76000708** | **1.12959295** |
| **YLDs (Years Lived with Disability)** | **Guinea** | **Both** | **Age-standardized** | **Gout** | **Kidney dysfunction** | **Rate** | **2021** | **1.83133355** | **2.76000708** | **1.12959295** |
| **DALYs (Disability-Adjusted Life Years)** | **Syrian Arab Republic** | **Both** | **Age-standardized** | **Gout** | **Kidney dysfunction** | **Rate** | **2021** | **1.830877719** | **2.72629491** | **1.14300021** |
| **YLDs (Years Lived with Disability)** | **Syrian Arab Republic** | **Both** | **Age-standardized** | **Gout** | **Kidney dysfunction** | **Rate** | **2021** | **1.830877719** | **2.72629491** | **1.14300021** |
| **DALYs (Disability-Adjusted Life Years)** | **Pakistan** | **Both** | **Age-standardized** | **Gout** | **Kidney dysfunction** | **Rate** | **2021** | **1.826975738** | **2.72153718** | **1.13491607** |
| **YLDs (Years Lived with Disability)** | **Pakistan** | **Both** | **Age-standardized** | **Gout** | **Kidney dysfunction** | **Rate** | **2021** | **1.826975738** | **2.72153718** | **1.13491607** |
| **DALYs (Disability-Adjusted Life Years)** | **Gambia** | **Both** | **Age-standardized** | **Gout** | **Kidney dysfunction** | **Rate** | **2021** | **1.823343618** | **2.70588767** | **1.14785871** |
| **YLDs (Years Lived with Disability)** | **Gambia** | **Both** | **Age-standardized** | **Gout** | **Kidney dysfunction** | **Rate** | **2021** | **1.823343618** | **2.70588767** | **1.14785871** |
| **DALYs (Disability-Adjusted Life Years)** | **Palestine** | **Both** | **Age-standardized** | **Gout** | **Kidney dysfunction** | **Rate** | **2021** | **1.786203278** | **2.66917014** | **1.10335012** |
| **YLDs (Years Lived with Disability)** | **Palestine** | **Both** | **Age-standardized** | **Gout** | **Kidney dysfunction** | **Rate** | **2021** | **1.786203278** | **2.66917014** | **1.10335012** |
| **DALYs (Disability-Adjusted Life Years)** | **Chad** | **Both** | **Age-standardized** | **Gout** | **Kidney dysfunction** | **Rate** | **2021** | **1.779714363** | **2.67192581** | **1.09839088** |
| **YLDs (Years Lived with Disability)** | **Chad** | **Both** | **Age-standardized** | **Gout** | **Kidney dysfunction** | **Rate** | **2021** | **1.779714363** | **2.67192581** | **1.09839088** |
| **DALYs (Disability-Adjusted Life Years)** | **Sudan** | **Both** | **Age-standardized** | **Gout** | **Kidney dysfunction** | **Rate** | **2021** | **1.774455224** | **2.73267005** | **1.08339571** |
| **YLDs (Years Lived with Disability)** | **Sudan** | **Both** | **Age-standardized** | **Gout** | **Kidney dysfunction** | **Rate** | **2021** | **1.774455224** | **2.73267005** | **1.08339571** |
| **DALYs (Disability-Adjusted Life Years)** | **Latvia** | **Both** | **Age-standardized** | **Gout** | **Kidney dysfunction** | **Rate** | **2021** | **1.763700502** | **2.6028435** | **1.10054967** |
| **YLDs (Years Lived with Disability)** | **Latvia** | **Both** | **Age-standardized** | **Gout** | **Kidney dysfunction** | **Rate** | **2021** | **1.763700502** | **2.6028435** | **1.10054967** |
| **DALYs (Disability-Adjusted Life Years)** | **Turkey** | **Both** | **Age-standardized** | **Gout** | **Kidney dysfunction** | **Rate** | **2021** | **1.738612436** | **2.61828316** | **1.09270402** |
| **YLDs (Years Lived with Disability)** | **Turkey** | **Both** | **Age-standardized** | **Gout** | **Kidney dysfunction** | **Rate** | **2021** | **1.738612436** | **2.61828316** | **1.09270402** |
| **DALYs (Disability-Adjusted Life Years)** | **Sierra Leone** | **Both** | **Age-standardized** | **Gout** | **Kidney dysfunction** | **Rate** | **2021** | **1.730727177** | **2.64320603** | **1.0771202** |
| **YLDs (Years Lived with Disability)** | **Sierra Leone** | **Both** | **Age-standardized** | **Gout** | **Kidney dysfunction** | **Rate** | **2021** | **1.730727177** | **2.64320603** | **1.0771202** |
| **DALYs (Disability-Adjusted Life Years)** | **Ukraine** | **Both** | **Age-standardized** | **Gout** | **Kidney dysfunction** | **Rate** | **2021** | **1.723530894** | **2.60593588** | **1.0885061** |
| **YLDs (Years Lived with Disability)** | **Ukraine** | **Both** | **Age-standardized** | **Gout** | **Kidney dysfunction** | **Rate** | **2021** | **1.723530894** | **2.60593588** | **1.0885061** |
| **DALYs (Disability-Adjusted Life Years)** | **Lebanon** | **Both** | **Age-standardized** | **Gout** | **Kidney dysfunction** | **Rate** | **2021** | **1.720272302** | **2.56290025** | **1.0588813** |
| **YLDs (Years Lived with Disability)** | **Lebanon** | **Both** | **Age-standardized** | **Gout** | **Kidney dysfunction** | **Rate** | **2021** | **1.720272302** | **2.56290025** | **1.0588813** |
| **DALYs (Disability-Adjusted Life Years)** | **Guinea-Bissau** | **Both** | **Age-standardized** | **Gout** | **Kidney dysfunction** | **Rate** | **2021** | **1.71955871** | **2.60386625** | **1.08185228** |
| **YLDs (Years Lived with Disability)** | **Guinea-Bissau** | **Both** | **Age-standardized** | **Gout** | **Kidney dysfunction** | **Rate** | **2021** | **1.71955871** | **2.60386625** | **1.08185228** |
| **DALYs (Disability-Adjusted Life Years)** | **Burkina Faso** | **Both** | **Age-standardized** | **Gout** | **Kidney dysfunction** | **Rate** | **2021** | **1.717279934** | **2.58008289** | **1.06985477** |
| **YLDs (Years Lived with Disability)** | **Burkina Faso** | **Both** | **Age-standardized** | **Gout** | **Kidney dysfunction** | **Rate** | **2021** | **1.717279934** | **2.58008289** | **1.06985477** |
| **DALYs (Disability-Adjusted Life Years)** | **Cabo Verde** | **Both** | **Age-standardized** | **Gout** | **Kidney dysfunction** | **Rate** | **2021** | **1.716534416** | **2.57096832** | **1.06437378** |
| **YLDs (Years Lived with Disability)** | **Cabo Verde** | **Both** | **Age-standardized** | **Gout** | **Kidney dysfunction** | **Rate** | **2021** | **1.716534416** | **2.57096832** | **1.06437378** |
| **DALYs (Disability-Adjusted Life Years)** | **Norway** | **Both** | **Age-standardized** | **Gout** | **Kidney dysfunction** | **Rate** | **2021** | **1.713341312** | **2.56103477** | **1.05532885** |
| **YLDs (Years Lived with Disability)** | **Norway** | **Both** | **Age-standardized** | **Gout** | **Kidney dysfunction** | **Rate** | **2021** | **1.713341312** | **2.56103477** | **1.05532885** |
| **DALYs (Disability-Adjusted Life Years)** | **Lithuania** | **Both** | **Age-standardized** | **Gout** | **Kidney dysfunction** | **Rate** | **2021** | **1.701573442** | **2.56543232** | **1.07910536** |
| **YLDs (Years Lived with Disability)** | **Lithuania** | **Both** | **Age-standardized** | **Gout** | **Kidney dysfunction** | **Rate** | **2021** | **1.701573442** | **2.56543232** | **1.07910536** |
| **DALYs (Disability-Adjusted Life Years)** | **Tunisia** | **Both** | **Age-standardized** | **Gout** | **Kidney dysfunction** | **Rate** | **2021** | **1.694681946** | **2.52346455** | **1.04145333** |
| **YLDs (Years Lived with Disability)** | **Tunisia** | **Both** | **Age-standardized** | **Gout** | **Kidney dysfunction** | **Rate** | **2021** | **1.694681946** | **2.52346455** | **1.04145333** |
| **DALYs (Disability-Adjusted Life Years)** | **Niger** | **Both** | **Age-standardized** | **Gout** | **Kidney dysfunction** | **Rate** | **2021** | **1.678541525** | **2.49816307** | **1.04745745** |
| **YLDs (Years Lived with Disability)** | **Niger** | **Both** | **Age-standardized** | **Gout** | **Kidney dysfunction** | **Rate** | **2021** | **1.678541525** | **2.49816307** | **1.04745745** |
| **DALYs (Disability-Adjusted Life Years)** | **Italy** | **Both** | **Age-standardized** | **Gout** | **Kidney dysfunction** | **Rate** | **2021** | **1.669158291** | **2.51620606** | **1.02378272** |
| **YLDs (Years Lived with Disability)** | **Italy** | **Both** | **Age-standardized** | **Gout** | **Kidney dysfunction** | **Rate** | **2021** | **1.669158291** | **2.51620606** | **1.02378272** |
| **DALYs (Disability-Adjusted Life Years)** | **Portugal** | **Both** | **Age-standardized** | **Gout** | **Kidney dysfunction** | **Rate** | **2021** | **1.657228979** | **2.5252048** | **1.02878338** |
| **YLDs (Years Lived with Disability)** | **Portugal** | **Both** | **Age-standardized** | **Gout** | **Kidney dysfunction** | **Rate** | **2021** | **1.657228979** | **2.5252048** | **1.02878338** |
| **DALYs (Disability-Adjusted Life Years)** | **Togo** | **Both** | **Age-standardized** | **Gout** | **Kidney dysfunction** | **Rate** | **2021** | **1.644494932** | **2.47332999** | **1.03175947** |
| **YLDs (Years Lived with Disability)** | **Togo** | **Both** | **Age-standardized** | **Gout** | **Kidney dysfunction** | **Rate** | **2021** | **1.644494932** | **2.47332999** | **1.03175947** |
| **DALYs (Disability-Adjusted Life Years)** | **North Macedonia** | **Both** | **Age-standardized** | **Gout** | **Kidney dysfunction** | **Rate** | **2021** | **1.639238992** | **2.48224214** | **0.97466073** |
| **YLDs (Years Lived with Disability)** | **North Macedonia** | **Both** | **Age-standardized** | **Gout** | **Kidney dysfunction** | **Rate** | **2021** | **1.639238992** | **2.48224214** | **0.97466073** |
| **DALYs (Disability-Adjusted Life Years)** | **Belarus** | **Both** | **Age-standardized** | **Gout** | **Kidney dysfunction** | **Rate** | **2021** | **1.618117467** | **2.3920655** | **1.01046793** |
| **YLDs (Years Lived with Disability)** | **Belarus** | **Both** | **Age-standardized** | **Gout** | **Kidney dysfunction** | **Rate** | **2021** | **1.618117467** | **2.3920655** | **1.01046793** |
| **DALYs (Disability-Adjusted Life Years)** | **Afghanistan** | **Both** | **Age-standardized** | **Gout** | **Kidney dysfunction** | **Rate** | **2021** | **1.595081522** | **2.40437634** | **1.00405872** |
| **YLDs (Years Lived with Disability)** | **Afghanistan** | **Both** | **Age-standardized** | **Gout** | **Kidney dysfunction** | **Rate** | **2021** | **1.595081522** | **2.40437634** | **1.00405872** |
| **DALYs (Disability-Adjusted Life Years)** | **Romania** | **Both** | **Age-standardized** | **Gout** | **Kidney dysfunction** | **Rate** | **2021** | **1.536694857** | **2.26320239** | **0.97323829** |
| **YLDs (Years Lived with Disability)** | **Romania** | **Both** | **Age-standardized** | **Gout** | **Kidney dysfunction** | **Rate** | **2021** | **1.536694857** | **2.26320239** | **0.97323829** |
| **DALYs (Disability-Adjusted Life Years)** | **Bangladesh** | **Both** | **Age-standardized** | **Gout** | **Kidney dysfunction** | **Rate** | **2021** | **1.528358724** | **2.29029397** | **0.9540448** |
| **YLDs (Years Lived with Disability)** | **Bangladesh** | **Both** | **Age-standardized** | **Gout** | **Kidney dysfunction** | **Rate** | **2021** | **1.528358724** | **2.29029397** | **0.9540448** |
| **DALYs (Disability-Adjusted Life Years)** | **India** | **Both** | **Age-standardized** | **Gout** | **Kidney dysfunction** | **Rate** | **2021** | **1.513279329** | **2.25367727** | **0.95715096** |
| **YLDs (Years Lived with Disability)** | **India** | **Both** | **Age-standardized** | **Gout** | **Kidney dysfunction** | **Rate** | **2021** | **1.513279329** | **2.25367727** | **0.95715096** |
| **DALYs (Disability-Adjusted Life Years)** | **Russian Federation** | **Both** | **Age-standardized** | **Gout** | **Kidney dysfunction** | **Rate** | **2021** | **1.507647948** | **2.25321237** | **0.94789733** |
| **YLDs (Years Lived with Disability)** | **Russian Federation** | **Both** | **Age-standardized** | **Gout** | **Kidney dysfunction** | **Rate** | **2021** | **1.507647948** | **2.25321237** | **0.94789733** |
| **DALYs (Disability-Adjusted Life Years)** | **Yemen** | **Both** | **Age-standardized** | **Gout** | **Kidney dysfunction** | **Rate** | **2021** | **1.4571985** | **2.1787478** | **0.89302557** |
| **YLDs (Years Lived with Disability)** | **Yemen** | **Both** | **Age-standardized** | **Gout** | **Kidney dysfunction** | **Rate** | **2021** | **1.4571985** | **2.1787478** | **0.89302557** |
| **DALYs (Disability-Adjusted Life Years)** | **Montenegro** | **Both** | **Age-standardized** | **Gout** | **Kidney dysfunction** | **Rate** | **2021** | **1.409669541** | **2.11782846** | **0.88727274** |
| **YLDs (Years Lived with Disability)** | **Montenegro** | **Both** | **Age-standardized** | **Gout** | **Kidney dysfunction** | **Rate** | **2021** | **1.409669541** | **2.11782846** | **0.88727274** |
| **DALYs (Disability-Adjusted Life Years)** | **Senegal** | **Both** | **Age-standardized** | **Gout** | **Kidney dysfunction** | **Rate** | **2021** | **1.374446722** | **2.10020439** | **0.85110364** |
| **YLDs (Years Lived with Disability)** | **Senegal** | **Both** | **Age-standardized** | **Gout** | **Kidney dysfunction** | **Rate** | **2021** | **1.374446722** | **2.10020439** | **0.85110364** |
| **DALYs (Disability-Adjusted Life Years)** | **Morocco** | **Both** | **Age-standardized** | **Gout** | **Kidney dysfunction** | **Rate** | **2021** | **1.366814037** | **2.01704958** | **0.85904208** |
| **YLDs (Years Lived with Disability)** | **Morocco** | **Both** | **Age-standardized** | **Gout** | **Kidney dysfunction** | **Rate** | **2021** | **1.366814037** | **2.01704958** | **0.85904208** |
| **DALYs (Disability-Adjusted Life Years)** | **Bulgaria** | **Both** | **Age-standardized** | **Gout** | **Kidney dysfunction** | **Rate** | **2021** | **1.334933044** | **2.03899269** | **0.83144124** |
| **YLDs (Years Lived with Disability)** | **Bulgaria** | **Both** | **Age-standardized** | **Gout** | **Kidney dysfunction** | **Rate** | **2021** | **1.334933044** | **2.03899269** | **0.83144124** |
| **DALYs (Disability-Adjusted Life Years)** | **Poland** | **Both** | **Age-standardized** | **Gout** | **Kidney dysfunction** | **Rate** | **2021** | **1.305768007** | **1.95776779** | **0.81290711** |
| **YLDs (Years Lived with Disability)** | **Poland** | **Both** | **Age-standardized** | **Gout** | **Kidney dysfunction** | **Rate** | **2021** | **1.305768007** | **1.95776779** | **0.81290711** |
| **DALYs (Disability-Adjusted Life Years)** | **Albania** | **Both** | **Age-standardized** | **Gout** | **Kidney dysfunction** | **Rate** | **2021** | **1.266335189** | **1.90758499** | **0.79479594** |
| **YLDs (Years Lived with Disability)** | **Albania** | **Both** | **Age-standardized** | **Gout** | **Kidney dysfunction** | **Rate** | **2021** | **1.266335189** | **1.90758499** | **0.79479594** |
| **DALYs (Disability-Adjusted Life Years)** | **Croatia** | **Both** | **Age-standardized** | **Gout** | **Kidney dysfunction** | **Rate** | **2021** | **1.26439718** | **1.92684992** | **0.77732205** |
| **YLDs (Years Lived with Disability)** | **Croatia** | **Both** | **Age-standardized** | **Gout** | **Kidney dysfunction** | **Rate** | **2021** | **1.26439718** | **1.92684992** | **0.77732205** |
| **DALYs (Disability-Adjusted Life Years)** | **Viet Nam** | **Both** | **Age-standardized** | **Gout** | **Kidney dysfunction** | **Rate** | **2021** | **1.257182761** | **1.86494384** | **0.77442145** |
| **YLDs (Years Lived with Disability)** | **Viet Nam** | **Both** | **Age-standardized** | **Gout** | **Kidney dysfunction** | **Rate** | **2021** | **1.257182761** | **1.86494384** | **0.77442145** |
| **DALYs (Disability-Adjusted Life Years)** | **Bosnia and Herzegovina** | **Both** | **Age-standardized** | **Gout** | **Kidney dysfunction** | **Rate** | **2021** | **1.257175415** | **1.88848918** | **0.78908324** |
| **YLDs (Years Lived with Disability)** | **Bosnia and Herzegovina** | **Both** | **Age-standardized** | **Gout** | **Kidney dysfunction** | **Rate** | **2021** | **1.257175415** | **1.88848918** | **0.78908324** |
| **DALYs (Disability-Adjusted Life Years)** | **Hungary** | **Both** | **Age-standardized** | **Gout** | **Kidney dysfunction** | **Rate** | **2021** | **1.252145039** | **1.87290129** | **0.77081106** |
| **YLDs (Years Lived with Disability)** | **Hungary** | **Both** | **Age-standardized** | **Gout** | **Kidney dysfunction** | **Rate** | **2021** | **1.252145039** | **1.87290129** | **0.77081106** |
| **DALYs (Disability-Adjusted Life Years)** | **Slovakia** | **Both** | **Age-standardized** | **Gout** | **Kidney dysfunction** | **Rate** | **2021** | **1.238111504** | **1.86266163** | **0.7850946** |
| **YLDs (Years Lived with Disability)** | **Slovakia** | **Both** | **Age-standardized** | **Gout** | **Kidney dysfunction** | **Rate** | **2021** | **1.238111504** | **1.86266163** | **0.7850946** |
| **DALYs (Disability-Adjusted Life Years)** | **Czechia** | **Both** | **Age-standardized** | **Gout** | **Kidney dysfunction** | **Rate** | **2021** | **1.225414195** | **1.83992818** | **0.74382521** |
| **YLDs (Years Lived with Disability)** | **Czechia** | **Both** | **Age-standardized** | **Gout** | **Kidney dysfunction** | **Rate** | **2021** | **1.225414195** | **1.83992818** | **0.74382521** |
| **DALYs (Disability-Adjusted Life Years)** | **Slovenia** | **Both** | **Age-standardized** | **Gout** | **Kidney dysfunction** | **Rate** | **2021** | **1.202626362** | **1.82154337** | **0.73779996** |
| **YLDs (Years Lived with Disability)** | **Slovenia** | **Both** | **Age-standardized** | **Gout** | **Kidney dysfunction** | **Rate** | **2021** | **1.202626362** | **1.82154337** | **0.73779996** |
| **DALYs (Disability-Adjusted Life Years)** | **Ghana** | **Both** | **Age-standardized** | **Gout** | **Kidney dysfunction** | **Rate** | **2021** | **1.118268336** | **1.67430933** | **0.69560161** |
| **YLDs (Years Lived with Disability)** | **Ghana** | **Both** | **Age-standardized** | **Gout** | **Kidney dysfunction** | **Rate** | **2021** | **1.118268336** | **1.67430933** | **0.69560161** |
| **DALYs (Disability-Adjusted Life Years)** | **Brazil** | **Both** | **Age-standardized** | **Gout** | **Kidney dysfunction** | **Rate** | **2021** | **1.084712163** | **1.629427** | **0.69150301** |
| **YLDs (Years Lived with Disability)** | **Brazil** | **Both** | **Age-standardized** | **Gout** | **Kidney dysfunction** | **Rate** | **2021** | **1.084712163** | **1.629427** | **0.69150301** |
| **DALYs (Disability-Adjusted Life Years)** | **Serbia** | **Both** | **Age-standardized** | **Gout** | **Kidney dysfunction** | **Rate** | **2021** | **1.06252048** | **1.60514632** | **0.63473604** |
| **YLDs (Years Lived with Disability)** | **Serbia** | **Both** | **Age-standardized** | **Gout** | **Kidney dysfunction** | **Rate** | **2021** | **1.06252048** | **1.60514632** | **0.63473604** |
| **DALYs (Disability-Adjusted Life Years)** | **Paraguay** | **Both** | **Age-standardized** | **Gout** | **Kidney dysfunction** | **Rate** | **2021** | **1.041450151** | **1.55840183** | **0.66481572** |
| **YLDs (Years Lived with Disability)** | **Paraguay** | **Both** | **Age-standardized** | **Gout** | **Kidney dysfunction** | **Rate** | **2021** | **1.041450151** | **1.55840183** | **0.66481572** |
| **DALYs (Disability-Adjusted Life Years)** | **Nicaragua** | **Both** | **Age-standardized** | **Gout** | **Kidney dysfunction** | **Rate** | **2021** | **1.016499525** | **1.47652458** | **0.64293336** |
| **YLDs (Years Lived with Disability)** | **Nicaragua** | **Both** | **Age-standardized** | **Gout** | **Kidney dysfunction** | **Rate** | **2021** | **1.016499525** | **1.47652458** | **0.64293336** |
| **DALYs (Disability-Adjusted Life Years)** | **South Sudan** | **Both** | **Age-standardized** | **Gout** | **Kidney dysfunction** | **Rate** | **2021** | **1.014596208** | **1.52515765** | **0.62014136** |
| **YLDs (Years Lived with Disability)** | **South Sudan** | **Both** | **Age-standardized** | **Gout** | **Kidney dysfunction** | **Rate** | **2021** | **1.014596208** | **1.52515765** | **0.62014136** |
| **DALYs (Disability-Adjusted Life Years)** | **Djibouti** | **Both** | **Age-standardized** | **Gout** | **Kidney dysfunction** | **Rate** | **2021** | **1.004495698** | **1.50912713** | **0.60366067** |
| **YLDs (Years Lived with Disability)** | **Djibouti** | **Both** | **Age-standardized** | **Gout** | **Kidney dysfunction** | **Rate** | **2021** | **1.004495698** | **1.50912713** | **0.60366067** |
| **DALYs (Disability-Adjusted Life Years)** | **Zambia** | **Both** | **Age-standardized** | **Gout** | **Kidney dysfunction** | **Rate** | **2021** | **0.997421113** | **1.50330172** | **0.62689419** |
| **YLDs (Years Lived with Disability)** | **Zambia** | **Both** | **Age-standardized** | **Gout** | **Kidney dysfunction** | **Rate** | **2021** | **0.997421113** | **1.50330172** | **0.62689419** |
| **DALYs (Disability-Adjusted Life Years)** | **Kenya** | **Both** | **Age-standardized** | **Gout** | **Kidney dysfunction** | **Rate** | **2021** | **0.992245209** | **1.48335908** | **0.61298001** |
| **YLDs (Years Lived with Disability)** | **Kenya** | **Both** | **Age-standardized** | **Gout** | **Kidney dysfunction** | **Rate** | **2021** | **0.992245209** | **1.48335908** | **0.61298001** |
| **DALYs (Disability-Adjusted Life Years)** | **Ethiopia** | **Both** | **Age-standardized** | **Gout** | **Kidney dysfunction** | **Rate** | **2021** | **0.975405207** | **1.47703967** | **0.60403953** |
| **YLDs (Years Lived with Disability)** | **Ethiopia** | **Both** | **Age-standardized** | **Gout** | **Kidney dysfunction** | **Rate** | **2021** | **0.975405207** | **1.47703967** | **0.60403953** |
| **DALYs (Disability-Adjusted Life Years)** | **Uganda** | **Both** | **Age-standardized** | **Gout** | **Kidney dysfunction** | **Rate** | **2021** | **0.969984673** | **1.43992747** | **0.59574555** |
| **YLDs (Years Lived with Disability)** | **Uganda** | **Both** | **Age-standardized** | **Gout** | **Kidney dysfunction** | **Rate** | **2021** | **0.969984673** | **1.43992747** | **0.59574555** |
| **DALYs (Disability-Adjusted Life Years)** | **Comoros** | **Both** | **Age-standardized** | **Gout** | **Kidney dysfunction** | **Rate** | **2021** | **0.943396646** | **1.38554547** | **0.57438243** |
| **YLDs (Years Lived with Disability)** | **Comoros** | **Both** | **Age-standardized** | **Gout** | **Kidney dysfunction** | **Rate** | **2021** | **0.943396646** | **1.38554547** | **0.57438243** |
| **DALYs (Disability-Adjusted Life Years)** | **Burundi** | **Both** | **Age-standardized** | **Gout** | **Kidney dysfunction** | **Rate** | **2021** | **0.932422535** | **1.41311546** | **0.57336144** |
| **YLDs (Years Lived with Disability)** | **Burundi** | **Both** | **Age-standardized** | **Gout** | **Kidney dysfunction** | **Rate** | **2021** | **0.932422535** | **1.41311546** | **0.57336144** |
| **DALYs (Disability-Adjusted Life Years)** | **Belize** | **Both** | **Age-standardized** | **Gout** | **Kidney dysfunction** | **Rate** | **2021** | **0.920565418** | **1.38186613** | **0.57161912** |
| **YLDs (Years Lived with Disability)** | **Belize** | **Both** | **Age-standardized** | **Gout** | **Kidney dysfunction** | **Rate** | **2021** | **0.920565418** | **1.38186613** | **0.57161912** |
| **DALYs (Disability-Adjusted Life Years)** | **United Republic of Tanzania** | **Both** | **Age-standardized** | **Gout** | **Kidney dysfunction** | **Rate** | **2021** | **0.918681099** | **1.3812017** | **0.57045694** |
| **YLDs (Years Lived with Disability)** | **United Republic of Tanzania** | **Both** | **Age-standardized** | **Gout** | **Kidney dysfunction** | **Rate** | **2021** | **0.918681099** | **1.3812017** | **0.57045694** |
| **DALYs (Disability-Adjusted Life Years)** | **Malawi** | **Both** | **Age-standardized** | **Gout** | **Kidney dysfunction** | **Rate** | **2021** | **0.918451896** | **1.38142617** | **0.57667063** |
| **YLDs (Years Lived with Disability)** | **Malawi** | **Both** | **Age-standardized** | **Gout** | **Kidney dysfunction** | **Rate** | **2021** | **0.918451896** | **1.38142617** | **0.57667063** |
| **DALYs (Disability-Adjusted Life Years)** | **Madagascar** | **Both** | **Age-standardized** | **Gout** | **Kidney dysfunction** | **Rate** | **2021** | **0.904905806** | **1.34191409** | **0.55817598** |
| **YLDs (Years Lived with Disability)** | **Madagascar** | **Both** | **Age-standardized** | **Gout** | **Kidney dysfunction** | **Rate** | **2021** | **0.904905806** | **1.34191409** | **0.55817598** |
| **DALYs (Disability-Adjusted Life Years)** | **Costa Rica** | **Both** | **Age-standardized** | **Gout** | **Kidney dysfunction** | **Rate** | **2021** | **0.894712157** | **1.34160279** | **0.5524963** |
| **YLDs (Years Lived with Disability)** | **Costa Rica** | **Both** | **Age-standardized** | **Gout** | **Kidney dysfunction** | **Rate** | **2021** | **0.894712157** | **1.34160279** | **0.5524963** |
| **DALYs (Disability-Adjusted Life Years)** | **Ecuador** | **Both** | **Age-standardized** | **Gout** | **Kidney dysfunction** | **Rate** | **2021** | **0.886149068** | **1.34843573** | **0.54878495** |
| **YLDs (Years Lived with Disability)** | **Ecuador** | **Both** | **Age-standardized** | **Gout** | **Kidney dysfunction** | **Rate** | **2021** | **0.886149068** | **1.34843573** | **0.54878495** |
| **DALYs (Disability-Adjusted Life Years)** | **Dominica** | **Both** | **Age-standardized** | **Gout** | **Kidney dysfunction** | **Rate** | **2021** | **0.876641709** | **1.36600085** | **0.53349334** |
| **YLDs (Years Lived with Disability)** | **Dominica** | **Both** | **Age-standardized** | **Gout** | **Kidney dysfunction** | **Rate** | **2021** | **0.876641709** | **1.36600085** | **0.53349334** |
| **DALYs (Disability-Adjusted Life Years)** | **Mozambique** | **Both** | **Age-standardized** | **Gout** | **Kidney dysfunction** | **Rate** | **2021** | **0.873353078** | **1.31129445** | **0.53386157** |
| **YLDs (Years Lived with Disability)** | **Mozambique** | **Both** | **Age-standardized** | **Gout** | **Kidney dysfunction** | **Rate** | **2021** | **0.873353078** | **1.31129445** | **0.53386157** |
| **DALYs (Disability-Adjusted Life Years)** | **Rwanda** | **Both** | **Age-standardized** | **Gout** | **Kidney dysfunction** | **Rate** | **2021** | **0.870017953** | **1.2966164** | **0.5304738** |
| **YLDs (Years Lived with Disability)** | **Rwanda** | **Both** | **Age-standardized** | **Gout** | **Kidney dysfunction** | **Rate** | **2021** | **0.870017953** | **1.2966164** | **0.5304738** |
| **DALYs (Disability-Adjusted Life Years)** | **United States Virgin Islands** | **Both** | **Age-standardized** | **Gout** | **Kidney dysfunction** | **Rate** | **2021** | **0.862785041** | **1.31604842** | **0.53069741** |
| **YLDs (Years Lived with Disability)** | **United States Virgin Islands** | **Both** | **Age-standardized** | **Gout** | **Kidney dysfunction** | **Rate** | **2021** | **0.862785041** | **1.31604842** | **0.53069741** |
| **DALYs (Disability-Adjusted Life Years)** | **Puerto Rico** | **Both** | **Age-standardized** | **Gout** | **Kidney dysfunction** | **Rate** | **2021** | **0.854149874** | **1.30318153** | **0.52543341** |
| **YLDs (Years Lived with Disability)** | **Puerto Rico** | **Both** | **Age-standardized** | **Gout** | **Kidney dysfunction** | **Rate** | **2021** | **0.854149874** | **1.30318153** | **0.52543341** |
| **DALYs (Disability-Adjusted Life Years)** | **Trinidad and Tobago** | **Both** | **Age-standardized** | **Gout** | **Kidney dysfunction** | **Rate** | **2021** | **0.846505172** | **1.27547044** | **0.52350412** |
| **YLDs (Years Lived with Disability)** | **Trinidad and Tobago** | **Both** | **Age-standardized** | **Gout** | **Kidney dysfunction** | **Rate** | **2021** | **0.846505172** | **1.27547044** | **0.52350412** |
| **DALYs (Disability-Adjusted Life Years)** | **Eritrea** | **Both** | **Age-standardized** | **Gout** | **Kidney dysfunction** | **Rate** | **2021** | **0.844664564** | **1.24228766** | **0.51713226** |
| **YLDs (Years Lived with Disability)** | **Eritrea** | **Both** | **Age-standardized** | **Gout** | **Kidney dysfunction** | **Rate** | **2021** | **0.844664564** | **1.24228766** | **0.51713226** |
| **DALYs (Disability-Adjusted Life Years)** | **Bolivia (Plurinational State of)** | **Both** | **Age-standardized** | **Gout** | **Kidney dysfunction** | **Rate** | **2021** | **0.84025972** | **1.26311575** | **0.51707746** |
| **YLDs (Years Lived with Disability)** | **Bolivia (Plurinational State of)** | **Both** | **Age-standardized** | **Gout** | **Kidney dysfunction** | **Rate** | **2021** | **0.84025972** | **1.26311575** | **0.51707746** |
| **DALYs (Disability-Adjusted Life Years)** | **Jamaica** | **Both** | **Age-standardized** | **Gout** | **Kidney dysfunction** | **Rate** | **2021** | **0.83137312** | **1.26586177** | **0.51763055** |
| **YLDs (Years Lived with Disability)** | **Jamaica** | **Both** | **Age-standardized** | **Gout** | **Kidney dysfunction** | **Rate** | **2021** | **0.83137312** | **1.26586177** | **0.51763055** |
| **DALYs (Disability-Adjusted Life Years)** | **Somalia** | **Both** | **Age-standardized** | **Gout** | **Kidney dysfunction** | **Rate** | **2021** | **0.826729335** | **1.26480689** | **0.5050345** |
| **YLDs (Years Lived with Disability)** | **Somalia** | **Both** | **Age-standardized** | **Gout** | **Kidney dysfunction** | **Rate** | **2021** | **0.826729335** | **1.26480689** | **0.5050345** |
| **DALYs (Disability-Adjusted Life Years)** | **Suriname** | **Both** | **Age-standardized** | **Gout** | **Kidney dysfunction** | **Rate** | **2021** | **0.823898341** | **1.24502201** | **0.51476443** |
| **YLDs (Years Lived with Disability)** | **Suriname** | **Both** | **Age-standardized** | **Gout** | **Kidney dysfunction** | **Rate** | **2021** | **0.823898341** | **1.24502201** | **0.51476443** |
| **DALYs (Disability-Adjusted Life Years)** | **Saint Vincent and the Grenadines** | **Both** | **Age-standardized** | **Gout** | **Kidney dysfunction** | **Rate** | **2021** | **0.819388425** | **1.23575924** | **0.50500567** |
| **YLDs (Years Lived with Disability)** | **Saint Vincent and the Grenadines** | **Both** | **Age-standardized** | **Gout** | **Kidney dysfunction** | **Rate** | **2021** | **0.819388425** | **1.23575924** | **0.50500567** |
| **DALYs (Disability-Adjusted Life Years)** | **Guyana** | **Both** | **Age-standardized** | **Gout** | **Kidney dysfunction** | **Rate** | **2021** | **0.811497303** | **1.20535685** | **0.52174878** |
| **YLDs (Years Lived with Disability)** | **Guyana** | **Both** | **Age-standardized** | **Gout** | **Kidney dysfunction** | **Rate** | **2021** | **0.811497303** | **1.20535685** | **0.52174878** |
| **DALYs (Disability-Adjusted Life Years)** | **Saint Lucia** | **Both** | **Age-standardized** | **Gout** | **Kidney dysfunction** | **Rate** | **2021** | **0.808535031** | **1.21589067** | **0.50674737** |
| **YLDs (Years Lived with Disability)** | **Saint Lucia** | **Both** | **Age-standardized** | **Gout** | **Kidney dysfunction** | **Rate** | **2021** | **0.808535031** | **1.21589067** | **0.50674737** |
| **DALYs (Disability-Adjusted Life Years)** | **Saint Kitts and Nevis** | **Both** | **Age-standardized** | **Gout** | **Kidney dysfunction** | **Rate** | **2021** | **0.807775512** | **1.23448015** | **0.5148175** |
| **YLDs (Years Lived with Disability)** | **Saint Kitts and Nevis** | **Both** | **Age-standardized** | **Gout** | **Kidney dysfunction** | **Rate** | **2021** | **0.807775512** | **1.23448015** | **0.5148175** |
| **DALYs (Disability-Adjusted Life Years)** | **Antigua and Barbuda** | **Both** | **Age-standardized** | **Gout** | **Kidney dysfunction** | **Rate** | **2021** | **0.805079183** | **1.18842916** | **0.51205819** |
| **YLDs (Years Lived with Disability)** | **Antigua and Barbuda** | **Both** | **Age-standardized** | **Gout** | **Kidney dysfunction** | **Rate** | **2021** | **0.805079183** | **1.18842916** | **0.51205819** |
| **DALYs (Disability-Adjusted Life Years)** | **Grenada** | **Both** | **Age-standardized** | **Gout** | **Kidney dysfunction** | **Rate** | **2021** | **0.801590019** | **1.20633769** | **0.49378835** |
| **YLDs (Years Lived with Disability)** | **Grenada** | **Both** | **Age-standardized** | **Gout** | **Kidney dysfunction** | **Rate** | **2021** | **0.801590019** | **1.20633769** | **0.49378835** |
| **DALYs (Disability-Adjusted Life Years)** | **Mexico** | **Both** | **Age-standardized** | **Gout** | **Kidney dysfunction** | **Rate** | **2021** | **0.800893478** | **1.21187918** | **0.50410636** |
| **YLDs (Years Lived with Disability)** | **Mexico** | **Both** | **Age-standardized** | **Gout** | **Kidney dysfunction** | **Rate** | **2021** | **0.800893478** | **1.21187918** | **0.50410636** |
| **DALYs (Disability-Adjusted Life Years)** | **Bahamas** | **Both** | **Age-standardized** | **Gout** | **Kidney dysfunction** | **Rate** | **2021** | **0.795224573** | **1.19933991** | **0.48660986** |
| **YLDs (Years Lived with Disability)** | **Bahamas** | **Both** | **Age-standardized** | **Gout** | **Kidney dysfunction** | **Rate** | **2021** | **0.795224573** | **1.19933991** | **0.48660986** |
| **DALYs (Disability-Adjusted Life Years)** | **Barbados** | **Both** | **Age-standardized** | **Gout** | **Kidney dysfunction** | **Rate** | **2021** | **0.781472051** | **1.15797198** | **0.48117168** |
| **YLDs (Years Lived with Disability)** | **Barbados** | **Both** | **Age-standardized** | **Gout** | **Kidney dysfunction** | **Rate** | **2021** | **0.781472051** | **1.15797198** | **0.48117168** |
| **DALYs (Disability-Adjusted Life Years)** | **Dominican Republic** | **Both** | **Age-standardized** | **Gout** | **Kidney dysfunction** | **Rate** | **2021** | **0.758936216** | **1.14439439** | **0.47260214** |
| **YLDs (Years Lived with Disability)** | **Dominican Republic** | **Both** | **Age-standardized** | **Gout** | **Kidney dysfunction** | **Rate** | **2021** | **0.758936216** | **1.14439439** | **0.47260214** |
| **DALYs (Disability-Adjusted Life Years)** | **Bermuda** | **Both** | **Age-standardized** | **Gout** | **Kidney dysfunction** | **Rate** | **2021** | **0.743669866** | **1.09513427** | **0.46163322** |
| **YLDs (Years Lived with Disability)** | **Bermuda** | **Both** | **Age-standardized** | **Gout** | **Kidney dysfunction** | **Rate** | **2021** | **0.743669866** | **1.09513427** | **0.46163322** |
| **DALYs (Disability-Adjusted Life Years)** | **Honduras** | **Both** | **Age-standardized** | **Gout** | **Kidney dysfunction** | **Rate** | **2021** | **0.718605422** | **1.1330893** | **0.43988084** |
| **YLDs (Years Lived with Disability)** | **Honduras** | **Both** | **Age-standardized** | **Gout** | **Kidney dysfunction** | **Rate** | **2021** | **0.718605422** | **1.1330893** | **0.43988084** |
| **DALYs (Disability-Adjusted Life Years)** | **Cuba** | **Both** | **Age-standardized** | **Gout** | **Kidney dysfunction** | **Rate** | **2021** | **0.716003475** | **1.08808836** | **0.45144807** |
| **YLDs (Years Lived with Disability)** | **Cuba** | **Both** | **Age-standardized** | **Gout** | **Kidney dysfunction** | **Rate** | **2021** | **0.716003475** | **1.08808836** | **0.45144807** |
| **DALYs (Disability-Adjusted Life Years)** | **Guatemala** | **Both** | **Age-standardized** | **Gout** | **Kidney dysfunction** | **Rate** | **2021** | **0.7137156** | **1.08079785** | **0.44990704** |
| **YLDs (Years Lived with Disability)** | **Guatemala** | **Both** | **Age-standardized** | **Gout** | **Kidney dysfunction** | **Rate** | **2021** | **0.7137156** | **1.08079785** | **0.44990704** |
| **DALYs (Disability-Adjusted Life Years)** | **Panama** | **Both** | **Age-standardized** | **Gout** | **Kidney dysfunction** | **Rate** | **2021** | **0.703920223** | **1.04512716** | **0.44471434** |
| **YLDs (Years Lived with Disability)** | **Panama** | **Both** | **Age-standardized** | **Gout** | **Kidney dysfunction** | **Rate** | **2021** | **0.703920223** | **1.04512716** | **0.44471434** |
| **DALYs (Disability-Adjusted Life Years)** | **Venezuela (Bolivarian Republic of)** | **Both** | **Age-standardized** | **Gout** | **Kidney dysfunction** | **Rate** | **2021** | **0.701519101** | **1.07548919** | **0.4418807** |
| **YLDs (Years Lived with Disability)** | **Venezuela (Bolivarian Republic of)** | **Both** | **Age-standardized** | **Gout** | **Kidney dysfunction** | **Rate** | **2021** | **0.701519101** | **1.07548919** | **0.4418807** |
| **DALYs (Disability-Adjusted Life Years)** | **Haiti** | **Both** | **Age-standardized** | **Gout** | **Kidney dysfunction** | **Rate** | **2021** | **0.700381696** | **1.04488429** | **0.43462307** |
| **YLDs (Years Lived with Disability)** | **Haiti** | **Both** | **Age-standardized** | **Gout** | **Kidney dysfunction** | **Rate** | **2021** | **0.700381696** | **1.04488429** | **0.43462307** |
| **DALYs (Disability-Adjusted Life Years)** | **El Salvador** | **Both** | **Age-standardized** | **Gout** | **Kidney dysfunction** | **Rate** | **2021** | **0.652200316** | **0.97336082** | **0.40255166** |
| **YLDs (Years Lived with Disability)** | **El Salvador** | **Both** | **Age-standardized** | **Gout** | **Kidney dysfunction** | **Rate** | **2021** | **0.652200316** | **0.97336082** | **0.40255166** |
| **DALYs (Disability-Adjusted Life Years)** | **Peru** | **Both** | **Age-standardized** | **Gout** | **Kidney dysfunction** | **Rate** | **2021** | **0.647158585** | **0.96562442** | **0.39232564** |
| **YLDs (Years Lived with Disability)** | **Peru** | **Both** | **Age-standardized** | **Gout** | **Kidney dysfunction** | **Rate** | **2021** | **0.647158585** | **0.96562442** | **0.39232564** |
| **DALYs (Disability-Adjusted Life Years)** | **Colombia** | **Both** | **Age-standardized** | **Gout** | **Kidney dysfunction** | **Rate** | **2021** | **0.629758677** | **0.96728247** | **0.38942919** |
| **YLDs (Years Lived with Disability)** | **Colombia** | **Both** | **Age-standardized** | **Gout** | **Kidney dysfunction** | **Rate** | **2021** | **0.629758677** | **0.96728247** | **0.38942919** |

**Appendix 7：The estimated annual percentage change (EAPC) in age-standardized DALY rates for gout due to renal dysfunction across 204 countries, 1990–2021**

| **location** | **Num_1990** | **ASR_1990** | **Num_2021** | **ASR_2021** | **EAPC_CI** |
| --- | --- | --- | --- | --- | --- |
| **Afghanistan** | **98.5 (59.9-149.7)** | **1.6 (1-2.3)** | **130.9 (81.9-195.3)** | **1.6 (1-2.4)** | **0.15 (0.06-0.23)** |
| **Albania** | **22.2 (13.8-33.9)** | **1.2 (0.7-1.8)** | **56 (34.7-84.3)** | **1.3 (0.8-1.9)** | **0.31 (0.27-0.34)** |
| **Algeria** | **179.7 (109.6-275.5)** | **1.6 (1-2.5)** | **601.6(369.4-905.4)** | **1.8 (1.1-2.7)** | **0.47 (0.43-0.51)** |
| **American Samoa** | **0.6 (0.4-0.9)** | **3.1 (1.9-4.7)** | **1.6 (1-2.3)** | **3.5 (2.2-5.2)** | **0.42 (0.4-0.43)** |
| **Andorra** | **1.3 (0.8-1.9)** | **2.3 (1.4-3.5)** | **3.7 (2.3-5.8)** | **2.4 (1.5-3.7)** | **0.05 (0.02-0.07)** |
| **Angola** | **77.9 (48.5-117.3)** | **2.4 (1.5-3.5)** | **236.6 (149.6-354.2)** | **2.3 (1.5-3.5)** | **0.02 (-0.01-0.05)** |
| **Antigua and Barbuda** | **0.3 (0.2-0.5)** | **0.6 (0.4-0.9)** | **0.8 (0.5-1.2)** | **0.8 (0.5-1.2)** | **0.89 (0.85-0.93)** |
| **Argentina** | **642.5 (390-985.2)** | **2 (1.2-3.1)** | **1377.4 (855-2092.8)** | **2.4 (1.5-3.6)** | **0.49 (0.46-0.52)** |
| **Armenia** | **50.5 (32.1-76.4)** | **2 (1.3-3.1)** | **107 (68.1-160.5)** | **2.5 (1.6-3.7)** | **0.74 (0.7-0.78)** |
| **Australia** | **794.6 (497.7-1193.7)** | **3.9 (2.5-5.9)** | **2563.7 (1557.7-3942.9)** | **5.2 (3.2-8)** | **1.05 (0.96-1.15)** |
| **Austria** | **242.1 (148-365.1)** | **1.9 (1.2-2.9)** | **450.4 (278-675.5)** | **2.3 (1.4-3.4)** | **0.61 (0.59-0.63)** |
| **Azerbaijan** | **98.8 (61.4-145.9)** | **2.1 (1.3-3.1)** | **241.7 (150.1-363.5)** | **2.6 (1.6-3.9)** | **0.76 (0.71-0.81)** |
| **Bahamas** | **1 (0.6-1.5)** | **0.7 (0.4-1)** | **3 (1.9-4.7)** | **0.8 (0.5-1.2)** | **0.66 (0.63-0.68)** |
| **Bahrain** | **2.8 (1.7-4.2)** | **1.8 (1.1-2.7)** | **16.6 (10.2-26)** | **2 (1.2-3)** | **0.39 (0.36-0.43)** |
| **Bangladesh** | **634.6 (389.3-952.8)** | **1.5 (0.9-2.3)** | **1956 (1220.5-2928.9)** | **1.5 (1-2.3)** | **-0.05 (-0.07--0.02)** |
| **Barbados** | **1.9 (1.2-2.9)** | **0.6 (0.4-1)** | **4 (2.5-6)** | **0.8 (0.5-1.2)** | **0.72 (0.7-0.74)** |
| **Belarus** | **188.7 (118-281.2)** | **1.5 (0.9-2.2)** | **264.1 (164.3-391.2)** | **1.6 (1-2.4)** | **0.34 (0.31-0.37)** |
| **Belgium** | **338.1 (213.7-510.2)** | **2.1 (1.3-3.2)** | **564.7 (345.7-845.2)** | **2.2 (1.4-3.3)** | **0.2 (0.19-0.22)** |
| **Belize** | **0.6 (0.4-0.9)** | **0.7 (0.4-1)** | **2.6 (1.6-3.9)** | **0.9 (0.6-1.4)** | **1.06 (0.98-1.14)** |
| **Benin** | **32.5 (20.4-48.2)** | **1.8 (1.1-2.6)** | **86.9 (54.6-132)** | **1.9 (1.2-2.8)** | **0.2 (0.19-0.21)** |
| **Bermuda** | **0.4 (0.2-0.6)** | **0.6 (0.4-1)** | **1.1 (0.7-1.6)** | **0.7 (0.5-1.1)** | **0.55 (0.52-0.58)** |
| **Bhutan** | **3.6 (2.2-5.4)** | **1.7 (1.1-2.6)** | **11.4 (7-16.8)** | **2 (1.2-2.9)** | **0.46 (0.4-0.51)** |
| **Bolivia (Plurinational State of)** | **18.8 (11.8-28.5)** | **0.7 (0.4-1)** | **71.7 (43.9-106.4)** | **0.8 (0.5-1.3)** | **0.86 (0.83-0.89)** |
| **Bosnia and Herzegovina** | **40.7 (25.1-60.6)** | **1.1 (0.7-1.7)** | **80 (49.3-121.3)** | **1.3 (0.8-1.9)** | **0.37 (0.36-0.39)** |
| **Botswana** | **11.2 (6.8-16.8)** | **2.3 (1.4-3.4)** | **35.1 (22.1-53.6)** | **2.6 (1.7-3.9)** | **0.5 (0.42-0.59)** |
| **Brazil** | **717.8 (450.1-1083.7)** | **0.9 (0.6-1.3)** | **2688.4 (1718.6-4033.4)** | **1.1 (0.7-1.6)** | **0.65 (0.61-0.7)** |
| **Brunei Darussalam** | **2.9 (1.8-4.3)** | **3.2 (2-4.8)** | **10.8 (6.7-16.6)** | **3.5 (2.2-5.4)** | **0.36 (0.3-0.42)** |
| **Bulgaria** | **150.7 (90.5-224.5)** | **1.3 (0.8-1.9)** | **198.7 (121.1-308.1)** | **1.3 (0.8-2)** | **0.12 (0.08-0.16)** |
| **Burkina Faso** | **61.9 (38.4-91.8)** | **1.6 (1-2.4)** | **142.6 (88.8-217.2)** | **1.7 (1.1-2.6)** | **0.25 (0.24-0.27)** |
| **Burundi** | **19 (11.8-29)** | **0.9 (0.6-1.4)** | **37.6 (23.2-57.7)** | **0.9 (0.6-1.4)** | **0.08 (0.03-0.12)** |
| **Cabo Verde** | **3.7 (2.3-5.5)** | **1.6 (1-2.3)** | **7.3 (4.6-11)** | **1.7 (1.1-2.6)** | **0.37 (0.32-0.41)** |
| **Cambodia** | **68.1 (43.1-103.4)** | **1.8 (1.1-2.7)** | **206.7 (127.7-313.5)** | **1.9 (1.2-2.8)** | **0.24 (0.21-0.26)** |
| **Cameroon** | **100.8 (63-151)** | **2.5 (1.6-3.8)** | **347.7 (216.5-534)** | **3 (1.9-4.6)** | **0.62 (0.51-0.72)** |
| **Canada** | **1320 (806.5-2016.6)** | **4 (2.4-6.1)** | **3402.6 (2127.3-5069.8)** | **4.4 (2.7-6.6)** | **0.61 (0.53-0.69)** |
| **Central African Republic** | **22.1 (13.8-33.4)** | **2.3 (1.4-3.4)** | **41.9 (25.6-66.4)** | **2.2 (1.4-3.3)** | **-0.05 (-0.07--0.03)** |
| **Chad** | **42.7 (26.1-64.5)** | **1.6 (1-2.4)** | **89.8 (55.5-136.6)** | **1.8 (1.1-2.7)** | **0.34 (0.31-0.36)** |
| **Chile** | **226.4 (138.2-343.1)** | **2.4 (1.5-3.6)** | **714.7 (442.7-1093.8)** | **2.7 (1.7-4.2)** | **0.41 (0.38-0.44)** |
| **China** | **16358.6 (10117-24685.9)** | **2.2 (1.4-3.3)** | **40089.4 (24449.1-60711.2)** | **1.9 (1.2-2.9)** | **0.16 (-0.11-0.42)** |
| **Colombia** | **86.9 (53.9-130.6)** | **0.5 (0.3-0.8)** | **352.4 (218.8-537.8)** | **0.6 (0.4-1)** | **0.52 (0.49-0.55)** |
| **Comoros** | **1.6 (0.9-2.6)** | **1 (0.6-1.5)** | **4.1 (2.5-6)** | **0.9 (0.6-1.4)** | **-0.06 (-0.1--0.02)** |
| **Congo** | **22.9 (14.1-34.7)** | **2.5 (1.5-3.6)** | **64.7 (40.4-99.8)** | **2.8 (1.7-4.2)** | **0.44 (0.41-0.47)** |
| **Cook Islands** | **0.3 (0.2-0.5)** | **2.7 (1.7-3.9)** | **0.8 (0.5-1.2)** | **2.9 (1.8-4.5)** | **0.3 (0.27-0.33)** |
| **Costa Rica** | **12.8 (8-19)** | **0.8 (0.5-1.1)** | **49.5 (30.7-75.2)** | **0.9 (0.6-1.3)** | **0.67 (0.63-0.71)** |
| **Ivory Coast** | **64.1 (40-99.9)** | **1.9 (1.2-2.9)** | **190.2 (120.3-284.2)** | **1.9 (1.2-2.9)** | **0.1 (0.07-0.14)** |
| **Croatia** | **64.3 (40.2-97.5)** | **1.1 (0.7-1.7)** | **119.9 (73.7-180.8)** | **1.3 (0.8-1.9)** | **0.42 (0.4-0.44)** |
| **Cuba** | **60 (35.4-90)** | **0.6 (0.4-0.9)** | **144 (90.1-220.9)** | **0.7 (0.5-1.1)** | **0.62 (0.56-0.67)** |
| **Cyprus** | **18 (10.9-27.7)** | **2.2 (1.4-3.4)** | **51.1 (32.4-78.8)** | **2.3 (1.5-3.6)** | **0.1 (0.07-0.12)** |
| **Czechia** | **155.7 (94.4-233.9)** | **1.1 (0.7-1.7)** | **278.3 (169.1-416.1)** | **1.2 (0.7-1.8)** | **0.4 (0.17-0.63)** |
| **Democratic People's Republic of Korea** | **310.1 (192.9-466.1)** | **2.2 (1.4-3.3)** | **732.7 (448-1105.4)** | **2.3 (1.4-3.5)** | **0.12 (0.06-0.18)** |
| **Democratic Republic of the Congo** | **325 (203.6-477.9)** | **2.5 (1.6-3.6)** | **789.9 (501.9-1177.1)** | **2.4 (1.5-3.6)** | **-0.21 (-0.29--0.13)** |
| **Denmark** | **173.4 (105.9-266.1)** | **2 (1.2-3)** | **295.7 (180.8-459.7)** | **2.3 (1.4-3.5)** | **0.5 (0.47-0.53)** |
| **Djibouti** | **1 (0.6-1.5)** | **0.9 (0.6-1.4)** | **5.2 (3.2-8)** | **1 (0.6-1.5)** | **0.4 (0.36-0.44)** |
| **Dominica** | **0.4 (0.2-0.6)** | **0.7 (0.4-1)** | **0.7 (0.4-1.1)** | **0.9 (0.5-1.4)** | **0.93 (0.9-0.95)** |
| **Dominican Republic** | **19.9 (11.8-30.9)** | **0.6 (0.4-0.9)** | **74.5 (46.4-112.4)** | **0.8 (0.5-1.1)** | **0.88 (0.82-0.93)** |
| **Ecuador** | **33.1 (20.4-50)** | **0.7 (0.4-1)** | **142.6 (88.3-216.4)** | **0.9 (0.5-1.3)** | **1.25 (1.14-1.36)** |
| **Egypt** | **409.7 (258.7-609.5)** | **1.8 (1.1-2.6)** | **1190 (740.7-1856.3)** | **2.2 (1.4-3.4)** | **0.67 (0.66-0.69)** |
| **El Salvador** | **13.9 (8.5-21)** | **0.5 (0.3-0.7)** | **41.2 (25.7-61.9)** | **0.7 (0.4-1)** | **1.15 (1.11-1.2)** |
| **Equatorial Guinea** | **3.7 (2.4-5.6)** | **2.2 (1.4-3.3)** | **12.2 (7.7-18.6)** | **2.7 (1.7-4.2)** | **0.93 (0.84-1.02)** |
| **Eritrea** | **7.2 (4.4-11.2)** | **0.8 (0.5-1.3)** | **18.9 (11.9-29)** | **0.8 (0.5-1.2)** | **0.04 (0-0.08)** |
| **Estonia** | **31.8 (19.1-47.4)** | **1.6 (0.9-2.3)** | **54.5 (33.1-83.7)** | **1.9 (1.1-2.9)** | **0.71 (0.68-0.73)** |
| **Eswatini** | **6.8 (4.2-10.4)** | **2.6 (1.6-4)** | **13.9 (8.9-21.1)** | **2.8 (1.7-4.1)** | **0.1 (0.07-0.14)** |
| **Ethiopia** | **167.1 (104.4-256.7)** | **1 (0.6-1.5)** | **366 (226.9-564.1)** | **1 (0.6-1.5)** | **-0.12 (-0.17--0.08)** |
| **Fiji** | **8.1 (5.1-12.2)** | **2.6 (1.6-3.9)** | **20.1 (12.5-30.1)** | **2.9 (1.8-4.3)** | **0.31 (0.29-0.32)** |
| **Finland** | **120.5 (73.3-186.4)** | **1.6 (1-2.5)** | **270 (167.6-413.3)** | **1.9 (1.2-2.9)** | **0.46 (0.44-0.49)** |
| **France** | **1426.5 (873.3-2127.2)** | **1.6 (1-2.4)** | **3143.1 (1962.6-4866.7)** | **2 (1.3-3.1)** | **0.52 (0.43-0.6)** |
| **Gabon** | **13.5 (8.5-20.7)** | **2.5 (1.6-3.8)** | **27.9 (17.8-42.8)** | **2.9 (1.9-4.4)** | **0.52 (0.5-0.54)** |
| **Gambia** | **5.5 (3.4-8.4)** | **1.8 (1.1-2.6)** | **16.2 (10.2-24.3)** | **1.8 (1.1-2.7)** | **0.16 (0.14-0.17)** |
| **Georgia** | **129.7 (83.6-194.6)** | **2.1 (1.4-3.2)** | **144.1 (90.9-216.7)** | **2.3 (1.5-3.5)** | **0.34 (0.32-0.35)** |
| **Germany** | **2732.1 (1652-4191.4)** | **2 (1.2-3.1)** | **5362.1 (3217.2-8083)** | **2.5 (1.5-3.8)** | **0.72 (0.66-0.78)** |
| **Ghana** | **54.8 (33.2-83.2)** | **1 (0.6-1.6)** | **162.9 (101.4-247)** | **1.1 (0.7-1.7)** | **0.28 (0.25-0.31)** |
| **Greece** | **378.3 (238.4-587.4)** | **2.4 (1.5-3.7)** | **682.2 (423.8-1033.3)** | **2.5 (1.6-3.8)** | **0.48 (0.04-0.92)** |
| **Greenland** | **1.1 (0.7-1.6)** | **4 (2.5-5.9)** | **3.2 (2-4.8)** | **5.3 (3.3-7.9)** | **1 (0.95-1.04)** |
| **Grenada** | **0.5 (0.3-0.7)** | **0.6 (0.4-1)** | **0.9 (0.5-1.4)** | **0.8 (0.5-1.2)** | **0.7 (0.67-0.74)** |
| **Guam** | **1.6 (1-2.4)** | **2.5 (1.6-3.8)** | **6.1 (3.8-9.1)** | **2.8 (1.8-4.3)** | **0.43 (0.42-0.45)** |
| **Guatemala** | **16.9 (10.5-26.5)** | **0.5 (0.3-0.8)** | **76.7 (48.5-115.9)** | **0.7 (0.4-1.1)** | **0.93 (0.91-0.95)** |
| **Guinea** | **53.8 (34.1-79.6)** | **1.8 (1.1-2.6)** | **93.3 (57.4-140.5)** | **1.8 (1.1-2.8)** | **0.13 (0.09-0.17)** |
| **Guinea-Bissau** | **6.1 (3.8-9.4)** | **1.8 (1.1-2.7)** | **10.7 (6.8-16.1)** | **1.7 (1.1-2.6)** | **-0.11 (-0.14--0.09)** |
| **Guyana** | **2.2 (1.4-3.3)** | **0.6 (0.4-1)** | **4.8 (3-7.2)** | **0.8 (0.5-1.2)** | **0.77 (0.75-0.79)** |
| **Haiti** | **16.6 (10.2-24.9)** | **0.6 (0.4-0.9)** | **43.3 (26.4-64.8)** | **0.7 (0.4-1)** | **0.54 (0.51-0.57)** |
| **Honduras** | **10.5 (6.5-16)** | **0.5 (0.3-0.8)** | **43.4 (26.5-68.2)** | **0.7 (0.4-1.1)** | **0.9 (0.85-0.95)** |
| **Hungary** | **169.3 (102.5-252)** | **1.2 (0.7-1.7)** | **259 (158.5-386)** | **1.3 (0.8-1.9)** | **0.22 (0.2-0.24)** |
| **Iceland** | **5.7 (3.5-8.6)** | **1.9 (1.2-2.9)** | **11.8 (7.3-17.8)** | **1.9 (1.2-2.9)** | **0.16 (0.09-0.23)** |
| **India** | **6535.1 (4144.8-9835.4)** | **1.7 (1.1-2.5)** | **16364.1 (10265.9-24635.3)** | **1.5 (1-2.3)** | **-0.36 (-0.43--0.29)** |
| **Indonesia** | **1660.4 (1059.3-2490)** | **2 (1.2-2.9)** | **4906.9 (3083.4-7434.1)** | **2.3 (1.4-3.5)** | **0.54 (0.51-0.57)** |
| **Iran (Islamic Republic of)** | **495.4 (307.7-754.9)** | **2.1 (1.3-3.2)** | **1684.5 (1051-2535.3)** | **2.3 (1.4-3.5)** | **0.27 (0.21-0.33)** |
| **Iraq** | **137.9 (84-208.8)** | **1.8 (1.1-2.8)** | **407.7 (258.3-616.5)** | **1.9 (1.2-2.8)** | **0.14 (0.1-0.18)** |
| **Ireland** | **105 (65.5-162.9)** | **2.5 (1.5-3.8)** | **232.3 (148.9-353.4)** | **2.8 (1.8-4.3)** | **0.39 (0.37-0.41)** |
| **Israel** | **120.8 (72.4-184.9)** | **2.4 (1.5-3.7)** | **335.9 (210.7-515)** | **2.6 (1.6-3.9)** | **0.19 (0.18-0.2)** |
| **Italy** | **1655.3 (1011.2-2483.1)** | **1.8 (1.1-2.7)** | **2743.7 (1702.6-4127.1)** | **1.7 (1-2.5)** | **0.04 (-0.16-0.24)** |
| **Jamaica** | **12 (7.2-17.8)** | **0.7 (0.4-1)** | **26.7 (16.6-40.3)** | **0.8 (0.5-1.3)** | **0.78 (0.71-0.85)** |
| **Japan** | **4826.6 (2978.5-7240)** | **2.9 (1.8-4.3)** | **12615.8 (7772.9-18799.3)** | **3.1 (1.9-4.7)** | **0.36 (0.33-0.4)** |
| **Jordan** | **21.2 (13.4-32.4)** | **1.8 (1.1-2.7)** | **142.1 (87.7-213.2)** | **2.1 (1.3-3.2)** | **0.55 (0.51-0.58)** |
| **Kazakhstan** | **250 (157.5-378.4)** | **2.1 (1.3-3.2)** | **408.7 (254.2-607.4)** | **2.4 (1.5-3.6)** | **0.5 (0.49-0.52)** |
| **Kenya** | **70 (43.2-106.1)** | **1 (0.6-1.5)** | **194.4 (121.7-296.7)** | **1 (0.6-1.5)** | **0.06 (-0.04-0.15)** |
| **Kiribati** | **0.8 (0.5-1.2)** | **2.4 (1.5-3.6)** | **1.6 (1-2.4)** | **2.5 (1.6-3.8)** | **0.17 (0.15-0.19)** |
| **Kuwait** | **10.4 (6.6-15.5)** | **1.9 (1.2-2.9)** | **52.5 (32.6-78.5)** | **2 (1.2-3.1)** | **0.35 (0.28-0.43)** |
| **Kyrgyzstan** | **60.4 (37.8-89)** | **2.1 (1.3-3.1)** | **101.6 (63.6-153.9)** | **2.3 (1.4-3.4)** | **0.29 (0.27-0.3)** |
| **Lao People's Democratic Republic** | **38.6 (23.6-59)** | **2.1 (1.3-3.1)** | **98.7 (62.2-147.5)** | **2.4 (1.5-3.6)** | **0.49 (0.47-0.52)** |
| **Latvia** | **54.8 (33.6-83.2)** | **1.5 (1-2.3)** | **75.2 (46.1-109.6)** | **1.8 (1.1-2.6)** | **0.52 (0.49-0.54)** |
| **Lebanon** | **31.2 (19.8-47.5)** | **1.6 (1-2.3)** | **106.2 (65.1-158.6)** | **1.7 (1.1-2.6)** | **0.35 (0.3-0.4)** |
| **Lesotho** | **16.4 (10.4-24.9)** | **2.1 (1.3-3.1)** | **23.5 (14.6-35.2)** | **2.4 (1.5-3.5)** | **0.49 (0.44-0.54)** |
| **Liberia** | **20.3 (12.8-30.8)** | **1.9 (1.2-2.9)** | **36.2 (23.1-54.4)** | **2 (1.2-3)** | **0.21 (0.14-0.29)** |
| **Libya** | **30.1 (18.8-45.6)** | **1.7 (1.1-2.6)** | **88.9 (55.4-132.2)** | **1.9 (1.2-2.8)** | **0.34 (0.29-0.38)** |
| **Lithuania** | **71.7 (44.3-107.6)** | **1.6 (1-2.4)** | **105.2 (66.7-160.4)** | **1.7 (1.1-2.6)** | **0.24 (0.22-0.27)** |
| **Luxembourg** | **12 (7.3-18.2)** | **2.1 (1.3-3.2)** | **24 (14.7-37.8)** | **2.2 (1.3-3.4)** | **0.12 (0.08-0.15)** |
| **Madagascar** | **40.3 (24.8-61.3)** | **0.9 (0.6-1.4)** | **79.3 (48.9-119)** | **0.9 (0.6-1.3)** | **-0.04 (-0.11-0.03)** |
| **Malawi** | **30 (18.5-45.5)** | **0.9 (0.6-1.4)** | **58.2 (36.5-88)** | **0.9 (0.6-1.4)** | **0.01 (-0.04-0.06)** |
| **Malaysia** | **213.4 (134.4-321.1)** | **2.5 (1.6-3.8)** | **772.4(476.3-1163.8)** | **2.9 (1.8-4.4)** | **0.57 (0.55-0.6)** |
| **Maldives** | **1.8 (1.1-2.8)** | **2.5 (1.5-3.8)** | **7.7 (4.8-11.6)** | **2.5 (1.6-3.8)** | **0.07 (0.04-0.11)** |
| **Mali** | **59.1 (37.2-88.5)** | **1.7 (1.1-2.6)** | **141 (88-207)** | **1.8 (1.2-2.7)** | **0.27 (0.24-0.29)** |
| **Malta** | **8.9 (5.5-13.5)** | **2.1 (1.3-3.1)** | **25 (15.4-37.7)** | **2.3 (1.4-3.5)** | **0.24 (0.22-0.26)** |
| **Marshall Islands** | **0.3 (0.2-0.5)** | **2.3 (1.4-3.5)** | **0.8 (0.5-1.2)** | **2.8 (1.7-4.1)** | **0.61 (0.6-0.63)** |
| **Mauritania** | **17.1 (10.9-26.2)** | **1.9 (1.2-2.8)** | **40.5 (26-62.8)** | **2.1 (1.3-3.2)** | **0.35 (0.31-0.38)** |
| **Mauritius** | **15.7 (9.9-23.7)** | **2.3 (1.4-3.5)** | **54.7 (34.9-82.9)** | **3 (1.9-4.5)** | **0.9 (0.88-0.93)** |
| **Mexico** | **261.7 (164.9-393.8)** | **0.7 (0.4-1)** | **996.1(627.4-1502.6)** | **0.8 (0.5-1.2)** | **1.02 (0.85-1.2)** |
| **Micronesia (Federated States of)** | **1.2 (0.7-1.8)** | **2.7 (1.7-4)** | **2 (1.2-3)** | **3 (1.9-4.5)** | **0.38 (0.35-0.4)** |
| **Monaco** | **1.7 (1-2.5)** | **2.1 (1.3-3.2)** | **2.6 (1.6-3.9)** | **2.3 (1.4-3.6)** | **0.31 (0.3-0.32)** |
| **Mongolia** | **23.2 (14.8-33.8)** | **2.4 (1.5-3.4)** | **49 (30.6-74.9)** | **2.4 (1.5-3.5)** | **0.07 (0.05-0.08)** |
| **Montenegro** | **8.1 (5-12.2)** | **1.4 (0.8-2)** | **13.6 (8.4-20.2)** | **1.4 (0.9-2.1)** | **0.22 (0.17-0.27)** |
| **Morocco** | **162.1 (98.7-241.7)** | **1.2 (0.7-1.8)** | **440.3 (278.5-646.3)** | **1.4 (0.9-2)** | **0.41 (0.35-0.46)** |
| **Mozambique** | **44.2 (27-67)** | **0.9 (0.5-1.3)** | **81.5 (50.7-121.6)** | **0.9 (0.5-1.3)** | **0.04 (-0.01-0.09)** |
| **Myanmar** | **388.4 (241.2-587.8)** | **1.9 (1.2-2.9)** | **918.2 (573.5-1369)** | **2.1 (1.3-3.1)** | **0.29 (0.26-0.32)** |
| **Namibia** | **13.3 (8.2-20)** | **2.3 (1.4-3.4)** | **29.7 (18.3-44.4)** | **2.4 (1.5-3.5)** | **0.13 (0.09-0.16)** |
| **Nauru** | **0.1 (0.1-0.2)** | **3.1 (1.9-4.7)** | **0.1 (0.1-0.2)** | **2.9 (1.8-4.3)** | **-0.17 (-0.28--0.07)** |
| **Nepal** | **195.6 (124.2-295.2)** | **2.4 (1.5-3.6)** | **589.8 (376.9-881.6)** | **2.7 (1.7-4)** | **0.38 (0.34-0.41)** |
| **Netherlands** | **424.3 (257.4-630)** | **2 (1.2-3)** | **869.4(542.9-1335.9)** | **2.3 (1.4-3.5)** | **0.17 (0.08-0.26)** |
| **New Zealand** | **207.9 (128-310.2)** | **5.1 (3.2-7.6)** | **486.8 (301.7-732.1)** | **5.4 (3.4-8.2)** | **0.24 (0.12-0.36)** |
| **Nicaragua** | **10.6 (6.5-16.5)** | **0.7 (0.4-1.1)** | **49.7 (31.1-72.1)** | **1 (0.6-1.5)** | **1.11 (1.05-1.16)** |
| **Niger** | **40.8 (25.1-61.4)** | **1.7 (1.1-2.6)** | **118.7 (73.8-181.9)** | **1.7 (1-2.5)** | **-0.06 (-0.09--0.03)** |
| **Nigeria** | **1043.1 (667.5-1576.8)** | **2.5 (1.6-3.7)** | **1913.8(1220.7-2898.1)** | **2.3 (1.5-3.5)** | **-0.25 (-0.37--0.13)** |
| **Niue** | **0.1 (0-0.1)** | **2.5 (1.5-3.8)** | **0.1 (0-0.1)** | **3 (1.9-4.6)** | **0.55 (0.51-0.58)** |
| **North Macedonia** | **26.3 (16.4-39.7)** | **1.5 (0.9-2.3)** | **52.4 (31.2-80.6)** | **1.6 (1-2.5)** | **0.21 (0.19-0.22)** |
| **Northern Mariana Islands** | **0.5 (0.3-0.8)** | **3.5 (2.1-5.2)** | **1.7 (1.1-2.6)** | **3.6 (2.3-5.4)** | **-0.07 (-0.1--0.03)** |
| **Norway** | **108.5 (66.8-163.2)** | **1.4 (0.9-2.2)** | **185.7 (115.7-276.7)** | **1.7 (1.1-2.6)** | **0.37 (0.07-0.67)** |
| **Oman** | **8.7 (5.3-13)** | **1.4 (0.9-2.2)** | **34.9 (21.6-52.3)** | **1.9 (1.2-2.9)** | **1.04 (0.97-1.11)** |
| **Pakistan** | **762.2 (477.2-1134.5)** | **1.5 (0.9-2.2)** | **1933.5 (1207.7-2930.6)** | **1.8 (1.1-2.7)** | **0.76 (0.65-0.86)** |
| **Palau** | **0.3 (0.2-0.4)** | **2.8 (1.8-4.3)** | **0.7 (0.4-1)** | **3.4 (2.1-5)** | **0.47 (0.43-0.52)** |
| **Palestine** | **13.3 (8.4-20.3)** | **1.6 (1-2.5)** | **41.4 (25.7-63.1)** | **1.8 (1.1-2.7)** | **0.19 (0.14-0.25)** |
| **Panama** | **7.5 (4.6-11.4)** | **0.5 (0.3-0.8)** | **31.4 (20-46.7)** | **0.7 (0.4-1)** | **0.96 (0.94-0.97)** |
| **Papua New Guinea** | **29.2 (18-44.2)** | **2.1 (1.3-3.1)** | **90.6 (54.5-137)** | **2.2 (1.4-3.3)** | **0.27 (0.25-0.29)** |
| **Paraguay** | **17.7 (11.1-25.9)** | **0.8 (0.5-1.2)** | **58.9 (37.2-87.8)** | **1 (0.7-1.6)** | **0.72 (0.69-0.75)** |
| **Peru** | **62.7 (39.9-93.7)** | **0.6 (0.3-0.8)** | **216 (131.7-322.6)** | **0.6 (0.4-1)** | **0.57 (0.55-0.59)** |
| **Philippines** | **511.9 (319.2-771.2)** | **2 (1.2-2.9)** | **1820.1 (1146.7-2732.3)** | **2.4 (1.5-3.6)** | **0.67 (0.59-0.75)** |
| **Poland** | **540.4 (337.4-818.5)** | **1.3 (0.8-1.9)** | **975.7 (610.9-1465.4)** | **1.3 (0.8-2)** | **-0.06 (-0.1--0.01)** |
| **Portugal** | **232.3 (140.1-363)** | **1.6 (1-2.5)** | **461 (282.3-703.2)** | **1.7 (1-2.5)** | **0.06 (-0.01-0.12)** |
| **Puerto Rico** | **25.4 (15.6-38.1)** | **0.7 (0.4-1.1)** | **66.2 (40.9-101.9)** | **0.9 (0.5-1.3)** | **0.58 (0.56-0.6)** |
| **Qatar** | **2.2 (1.3-3.2)** | **2.1 (1.3-3.2)** | **20.9 (12.8-30.9)** | **2.3 (1.4-3.5)** | **0.42 (0.37-0.47)** |
| **Republic of Korea** | **608.9 (374.3-923.8)** | **2.4 (1.5-3.6)** | **2389.5 (1482.7-3561.8)** | **2.5 (1.6-3.7)** | **0.1 (0.07-0.13)** |
| **Republic of Moldova** | **74.6 (47.5-111.8)** | **1.8 (1.2-2.7)** | **128.2 (80.8-193)** | **2.1 (1.3-3.2)** | **0.53 (0.5-0.55)** |
| **Romania** | **345.8 (210.7-520.2)** | **1.3 (0.8-2)** | **597.8 (377.1-879.4)** | **1.5 (1-2.3)** | **0.32 (0.24-0.39)** |
| **Russian Federation** | **2264.7 (1409-3409.9)** | **1.3 (0.8-2)** | **3656.7 (2295.7-5444.6)** | **1.5 (0.9-2.3)** | **0.49 (0.47-0.52)** |
| **Rwanda** | **22 (13.6-34.1)** | **0.9 (0.6-1.4)** | **46.4 (28.1-69.2)** | **0.9 (0.5-1.3)** | **-0.21 (-0.27--0.15)** |
| **Saint Kitts and Nevis** | **0.3 (0.2-0.4)** | **0.7 (0.4-1)** | **0.5 (0.3-0.8)** | **0.8 (0.5-1.2)** | **0.54 (0.51-0.56)** |
| **Saint Lucia** | **0.5 (0.3-0.8)** | **0.6 (0.4-1)** | **1.9 (1.2-2.9)** | **0.8 (0.5-1.2)** | **0.83 (0.8-0.87)** |
| **Saint Vincent and the Grenadines** | **0.4 (0.3-0.6)** | **0.6 (0.4-0.9)** | **1.1 (0.7-1.7)** | **0.8 (0.5-1.2)** | **1.08 (1.04-1.13)** |
| **Samoa** | **2.1 (1.4-3.2)** | **2.8 (1.7-4.1)** | **4 (2.5-6)** | **3 (1.9-4.5)** | **0.16 (0.13-0.2)** |
| **San Marino** | **0.8 (0.5-1.2)** | **2.1 (1.3-3.1)** | **1.9 (1.2-2.9)** | **2.2 (1.4-3.4)** | **0.26 (0.24-0.27)** |
| **Sao Tome and Principe** | **1.1 (0.7-1.7)** | **1.8 (1.1-2.7)** | **2.1 (1.4-3.2)** | **2.1 (1.3-3.1)** | **0.51 (0.47-0.54)** |
| **Saudi Arabia** | **100.1 (62.8-149.8)** | **1.9 (1.2-2.8)** | **422.8 (255.5-641.6)** | **2.5 (1.6-3.6)** | **0.93 (0.9-0.96)** |
| **Senegal** | **43.2 (26.8-65.8)** | **1.5 (0.9-2.2)** | **95.9 (58.8-148.7)** | **1.4 (0.9-2.1)** | **-0.28 (-0.35--0.21)** |
| **Serbia** | **101.4 (61-153.7)** | **1 (0.6-1.5)** | **182.8 (109.5-274.3)** | **1.1 (0.6-1.6)** | **0.27 (0.24-0.29)** |
| **Seychelles** | **1.3 (0.8-1.9)** | **2.2 (1.4-3.4)** | **3 (1.9-4.5)** | **2.7 (1.7-4)** | **0.64 (0.61-0.67)** |
| **Sierra Leone** | **32.8 (20.1-49.6)** | **1.7 (1.1-2.6)** | **58.5 (36.1-89.4)** | **1.7 (1.1-2.6)** | **0.06 (0.01-0.1)** |
| **Singapore** | **55.8 (34.4-84.1)** | **2.8 (1.7-4.3)** | **266.8 (168.9-407.8)** | **3.1 (2-4.8)** | **0.29 (0.26-0.32)** |
| **Slovakia** | **70.8 (44.1-107.2)** | **1.2 (0.8-1.8)** | **120 (75.7-181.9)** | **1.2 (0.8-1.9)** | **0.02 (0-0.05)** |
| **Slovenia** | **26.7 (16.1-40.7)** | **1.1 (0.7-1.7)** | **56.9 (34.8-85.9)** | **1.2 (0.7-1.8)** | **0.27 (0.24-0.3)** |
| **Solomon Islands** | **2.8 (1.8-4.1)** | **2.5 (1.6-3.7)** | **7.5 (4.7-11.1)** | **2.6 (1.6-3.8)** | **0.03 (0-0.05)** |
| **Somalia** | **17.4 (10.7-26.3)** | **0.9 (0.6-1.4)** | **40.2 (24.4-62.1)** | **0.8 (0.5-1.3)** | **-0.35 (-0.39--0.31)** |
| **South Africa** | **508.6 (324.6-763.3)** | **2.7 (1.7-4)** | **1256.3 (794.4-1888.2)** | **2.9 (1.8-4.3)** | **0.32 (0.28-0.36)** |
| **South Sudan** | **23.5 (14.2-35.4)** | **1 (0.6-1.5)** | **31.5 (19.1-47.4)** | **1 (0.6-1.5)** | **0.03 (-0.02-0.08)** |
| **Spain** | **1263.4 (778.2-1910.8)** | **2.2 (1.4-3.4)** | **2344 (1470.3-3576.9)** | **2.2 (1.4-3.4)** | **-0.13 (-0.16--0.1)** |
| **Sri Lanka** | **206.2 (129.6-312.9)** | **2.2 (1.3-3.2)** | **583.7 (362-882.7)** | **2.2 (1.4-3.3)** | **0.1 (0.06-0.14)** |
| **Sudan** | **124.4 (76.1-190.3)** | **1.5 (0.9-2.2)** | **307.4 (189.1-470.2)** | **1.8 (1.1-2.7)** | **0.69 (0.65-0.73)** |
| **Suriname** | **1.6 (1-2.5)** | **0.7 (0.4-1)** | **5.1 (3.2-7.6)** | **0.8 (0.5-1.2)** | **0.7 (0.67-0.73)** |
| **Sweden** | **297.6 (179.4-455.8)** | **1.8 (1.1-2.7)** | **468 (286.3-710.8)** | **1.9 (1.1-2.8)** | **0.51 (0.4-0.62)** |
| **Switzerland** | **227 (140.5-347.8)** | **2 (1.3-3.1)** | **448.5 (283.3-673.2)** | **2.3 (1.4-3.4)** | **0.32 (0.28-0.36)** |
| **Syrian Arab Republic** | **82.7 (51.9-126.6)** | **1.7 (1.1-2.6)** | **217.6 (134.9-325.4)** | **1.8 (1.1-2.7)** | **0.19 (0.13-0.24)** |
| **Taiwan (Province of China)** | **484.9 (311.4-712)** | **3.4 (2.2-4.9)** | **1529.8 (983.8-2248.8)** | **3.5 (2.3-5.2)** | **-0.27 (-1.05-0.51)** |
| **Tajikistan** | **47.7 (29.8-71.6)** | **1.9 (1.2-2.8)** | **105.3 (64.9-162.9)** | **2.1 (1.3-3.2)** | **0.48 (0.44-0.52)** |
| **Thailand** | **777 (499.4-1157.4)** | **2.4 (1.6-3.5)** | **3370.9 (2114.8-5058.8)** | **3.1 (1.9-4.6)** | **1.1 (0.97-1.22)** |
| **Timor-Leste** | **4.5 (2.9-6.8)** | **2 (1.2-3)** | **17.1 (10.6-25.9)** | **2.1 (1.4-3.2)** | **0.24 (0.22-0.27)** |
| **Togo** | **18 (11.1-27.1)** | **1.7 (1-2.6)** | **55.2 (34.9-82.2)** | **1.6 (1-2.5)** | **-0.12 (-0.15--0.09)** |
| **Tokelau** | **0 (0-0)** | **2.3 (1.5-3.5)** | **0 (0-0.1)** | **2.7 (1.7-4.1)** | **0.47 (0.46-0.49)** |
| **Tonga** | **1.3 (0.8-2)** | **2.6 (1.6-3.9)** | **2.3 (1.4-3.5)** | **3 (1.9-4.5)** | **0.3 (0.27-0.34)** |
| **Trinidad and Tobago** | **5.4 (3.3-8)** | **0.7 (0.4-1)** | **16.2 (9.8-24.5)** | **0.8 (0.5-1.3)** | **0.81 (0.78-0.83)** |
| **Tunisia** | **72.8 (43.9-109.4)** | **1.6 (1-2.3)** | **217.9 (133.5-328.3)** | **1.7 (1-2.5)** | **0.28 (0.26-0.31)** |
| **Turkey** | **535.8 (326-808.9)** | **1.7 (1-2.5)** | **1589.7 (1001.2-2420.1)** | **1.7 (1.1-2.6)** | **0.18 (0.12-0.24)** |
| **Turkmenistan** | **39.2 (24.7-59.1)** | **2.2 (1.4-3.4)** | **98.8 (60.6-148.4)** | **2.7 (1.7-4)** | **0.64 (0.6-0.68)** |
| **Tuvalu** | **0.1 (0.1-0.2)** | **2.2 (1.4-3.3)** | **0.3 (0.2-0.4)** | **2.7 (1.7-4)** | **0.58 (0.55-0.61)** |
| **Uganda** | **56.3 (34.6-88.9)** | **1 (0.6-1.6)** | **122.9 (75.8-186.5)** | **1 (0.6-1.4)** | **-0.01 (-0.09-0.08)** |
| **Ukraine** | **1092.9 (679.1-1627.1)** | **1.6 (1-2.3)** | **1376.8 (855-2080.1)** | **1.7 (1.1-2.6)** | **0.39 (0.35-0.42)** |
| **United Arab Emirates** | **9.6 (5.8-14.3)** | **2.2 (1.4-3.4)** | **128.3 (77-200)** | **3 (1.8-4.6)** | **1.05 (0.99-1.1)** |
| **United Kingdom** | **2402.4 (1473.7-3605.7)** | **2.5 (1.5-3.7)** | **3720.7 (2293.4-5560.5)** | **2.6 (1.6-4)** | **0.67 (0.41-0.93)** |
| **United Republic of Tanzania** | **82.3 (50.2-126.9)** | **0.9 (0.5-1.4)** | **204.9 (128.4-302.7)** | **0.9 (0.6-1.4)** | **0.19 (0.13-0.25)** |
| **United States of America** | **13807.6 (8538.6-20519)** | **4.1 (2.6-6.1)** | **44353.6 (28907.7-64911.9)** | **7.3 (4.8-10.6)** | **3.15 (2.7-3.61)** |
| **United States Virgin Islands** | **0.5 (0.3-0.8)** | **0.7 (0.4-1)** | **1.6 (1-2.5)** | **0.9 (0.5-1.3)** | **0.84 (0.78-0.89)** |
| **Uruguay** | **99.6 (61.9-156.9)** | **2.5 (1.6-3.9)** | **164.8 (101.3-247.5)** | **2.8 (1.7-4.2)** | **0.32 (0.3-0.34)** |
| **Uzbekistan** | **253.4 (157.1-372.1)** | **2.3 (1.4-3.4)** | **631.1 (396.5-961.2)** | **2.6 (1.7-4)** | **0.47 (0.45-0.49)** |
| **Vanuatu** | **1.3 (0.8-2)** | **2.4 (1.5-3.7)** | **4 (2.4-6)** | **2.7 (1.6-3.9)** | **0.31 (0.29-0.33)** |
| **Venezuela (Bolivarian Republic of)** | **52.2 (32.6-77.4)** | **0.6 (0.4-0.9)** | **208.3 (132.4-317.8)** | **0.7 (0.4-1.1)** | **0.73 (0.7-0.76)** |
| **Viet Nam** | **467.7 (283.9-687.7)** | **1.2 (0.8-1.8)** | **1144.7 (712.7-1707.3)** | **1.3 (0.8-1.9)** | **0.34 (0.2-0.47)** |
| **Yemen** | **55.8 (34.9-84.9)** | **1.3 (0.8-2)** | **179.7 (110.4-270)** | **1.5 (0.9-2.2)** | **0.48 (0.44-0.52)** |
| **Zambia** | **25 (15.4-38.5)** | **1.1 (0.6-1.6)** | **58.1 (36.2-89.2)** | **1 (0.6-1.5)** | **-0.13 (-0.19--0.07)** |
| **Zimbabwe** | **88 (54.3-133.8)** | **2.4 (1.5-3.6)** | **140.4 (85.2-217.4)** | **2.4 (1.4-3.6)** | **-0.05 (-0.08--0.02)** |

**Appendix 8：The estimated annual percentage change (EAPC) in age-standardized YLD rates for gout due to renal dysfunction across 204 countries, 1990–2021**

| **location** | **Num_1990** | **ASR_1990** | **Num_2021** | **ASR_2021** | **EAPC_CI** |
| --- | --- | --- | --- | --- | --- |
| **Afghanistan** | **98.5 (59.9-149.7)** | **1.6 (1-2.3)** | **130.9 (81.9-195.3)** | **1.6 (1-2.4)** | **0.15 (0.06-0.23)** |
| **Albania** | **22.2 (13.8-33.9)** | **1.2 (0.7-1.8)** | **56 (34.7-84.3)** | **1.3 (0.8-1.9)** | **0.31 (0.27-0.34)** |
| **Algeria** | **179.7 (109.6-275.5)** | **1.6 (1-2.5)** | **601.6 (369.4-905.4)** | **1.8 (1.1-2.7)** | **0.47 (0.43-0.51)** |
| **American Samoa** | **0.6 (0.4-0.9)** | **3.1 (1.9-4.7)** | **1.6 (1-2.3)** | **3.5 (2.2-5.2)** | **0.42 (0.4-0.43)** |
| **Andorra** | **1.3 (0.8-1.9)** | **2.3 (1.4-3.5)** | **3.7 (2.3-5.8)** | **2.4 (1.5-3.7)** | **0.05 (0.02-0.07)** |
| **Angola** | **77.9 (48.5-117.3)** | **2.4 (1.5-3.5)** | **236.6 (149.6-354.2)** | **2.3 (1.5-3.5)** | **0.02 (-0.01-0.05)** |
| **Antigua and Barbuda** | **0.3 (0.2-0.5)** | **0.6 (0.4-0.9)** | **0.8 (0.5-1.2)** | **0.8 (0.5-1.2)** | **0.89 (0.85-0.93)** |
| **Argentina** | **642.5 (390-985.2)** | **2 (1.2-3.1)** | **1377.4 (855-2092.8)** | **2.4 (1.5-3.6)** | **0.49 (0.46-0.52)** |
| **Armenia** | **50.5 (32.1-76.4)** | **2 (1.3-3.1)** | **107 (68.1-160.5)** | **2.5 (1.6-3.7)** | **0.74 (0.7-0.78)** |
| **Australia** | **794.6 (497.7-1193.7)** | **3.9 (2.5-5.9)** | **2563.7 (1557.7-3942.9)** | **5.2 (3.2-8)** | **1.05 (0.96-1.15)** |
| **Austria** | **242.1 (148-365.1)** | **1.9 (1.2-2.9)** | **450.4 (278-675.5)** | **2.3 (1.4-3.4)** | **0.61 (0.59-0.63)** |
| **Azerbaijan** | **98.8 (61.4-145.9)** | **2.1 (1.3-3.1)** | **241.7 (150.1-363.5)** | **2.6 (1.6-3.9)** | **0.76 (0.71-0.81)** |
| **Bahamas** | **1 (0.6-1.5)** | **0.7 (0.4-1)** | **3 (1.9-4.7)** | **0.8 (0.5-1.2)** | **0.66 (0.63-0.68)** |
| **Bahrain** | **2.8 (1.7-4.2)** | **1.8 (1.1-2.7)** | **16.6 (10.2-26)** | **2 (1.2-3)** | **0.39 (0.36-0.43)** |
| **Bangladesh** | **634.6 (389.3-952.8)** | **1.5 (0.9-2.3)** | **1956 (1220.5-2928.9)** | **1.5 (1-2.3)** | **-0.05 (-0.07--0.02)** |
| **Barbados** | **1.9 (1.2-2.9)** | **0.6 (0.4-1)** | **4 (2.5-6)** | **0.8 (0.5-1.2)** | **0.72 (0.7-0.74)** |
| **Belarus** | **188.7 (118-281.2)** | **1.5 (0.9-2.2)** | **264.1 (164.3-391.2)** | **1.6 (1-2.4)** | **0.34 (0.31-0.37)** |
| **Belgium** | **338.1 (213.7-510.2)** | **2.1 (1.3-3.2)** | **564.7 (345.7-845.2)** | **2.2 (1.4-3.3)** | **0.2 (0.19-0.22)** |
| **Belize** | **0.6 (0.4-0.9)** | **0.7 (0.4-1)** | **2.6 (1.6-3.9)** | **0.9 (0.6-1.4)** | **1.06 (0.98-1.14)** |
| **Benin** | **32.5 (20.4-48.2)** | **1.8 (1.1-2.6)** | **86.9 (54.6-132)** | **1.9 (1.2-2.8)** | **0.2 (0.19-0.21)** |
| **Bermuda** | **0.4 (0.2-0.6)** | **0.6 (0.4-1)** | **1.1 (0.7-1.6)** | **0.7 (0.5-1.1)** | **0.55 (0.52-0.58)** |
| **Bhutan** | **3.6 (2.2-5.4)** | **1.7 (1.1-2.6)** | **11.4 (7-16.8)** | **2 (1.2-2.9)** | **0.46 (0.4-0.51)** |
| **Bolivia (Plurinational State of)** | **18.8 (11.8-28.5)** | **0.7 (0.4-1)** | **71.7 (43.9-106.4)** | **0.8 (0.5-1.3)** | **0.86 (0.83-0.89)** |
| **Bosnia and Herzegovina** | **40.7 (25.1-60.6)** | **1.1 (0.7-1.7)** | **80 (49.3-121.3)** | **1.3 (0.8-1.9)** | **0.37 (0.36-0.39)** |
| **Botswana** | **11.2 (6.8-16.8)** | **2.3 (1.4-3.4)** | **35.1 (22.1-53.6)** | **2.6 (1.7-3.9)** | **0.5 (0.42-0.59)** |
| **Brazil** | **717.8 (450.1-1083.7)** | **0.9 (0.6-1.3)** | **2688.4 (1718.6-4033.4)** | **1.1 (0.7-1.6)** | **0.65 (0.61-0.7)** |
| **Brunei Darussalam** | **2.9 (1.8-4.3)** | **3.2 (2-4.8)** | **10.8 (6.7-16.6)** | **3.5 (2.2-5.4)** | **0.36 (0.3-0.42)** |
| **Bulgaria** | **150.7 (90.5-224.5)** | **1.3 (0.8-1.9)** | **198.7 (121.1-308.1)** | **1.3 (0.8-2)** | **0.12 (0.08-0.16)** |
| **Burkina Faso** | **61.9 (38.4-91.8)** | **1.6 (1-2.4)** | **142.6 (88.8-217.2)** | **1.7 (1.1-2.6)** | **0.25 (0.24-0.27)** |
| **Burundi** | **19 (11.8-29)** | **0.9 (0.6-1.4)** | **37.6 (23.2-57.7)** | **0.9 (0.6-1.4)** | **0.08 (0.03-0.12)** |
| **Cabo Verde** | **3.7 (2.3-5.5)** | **1.6 (1-2.3)** | **7.3 (4.6-11)** | **1.7 (1.1-2.6)** | **0.37 (0.32-0.41)** |
| **Cambodia** | **68.1 (43.1-103.4)** | **1.8 (1.1-2.7)** | **206.7 (127.7-313.5)** | **1.9 (1.2-2.8)** | **0.24 (0.21-0.26)** |
| **Cameroon** | **100.8 (63-151)** | **2.5 (1.6-3.8)** | **347.7 (216.5-534)** | **3 (1.9-4.6)** | **0.62 (0.51-0.72)** |
| **Canada** | **1320 (806.5-2016.6)** | **4 (2.4-6.1)** | **3402.6 (2127.3-5069.8)** | **4.4 (2.7-6.6)** | **0.61 (0.53-0.69)** |
| **Central African Republic** | **22.1 (13.8-33.4)** | **2.3 (1.4-3.4)** | **41.9 (25.6-66.4)** | **2.2 (1.4-3.3)** | **-0.05 (-0.07--0.03)** |
| **Chad** | **42.7 (26.1-64.5)** | **1.6 (1-2.4)** | **89.8 (55.5-136.6)** | **1.8 (1.1-2.7)** | **0.34 (0.31-0.36)** |
| **Chile** | **226.4 (138.2-343.1)** | **2.4 (1.5-3.6)** | **714.7 (442.7-1093.8)** | **2.7 (1.7-4.2)** | **0.41 (0.38-0.44)** |
| **China** | **16358.6 (10117-24685.9)** | **2.2 (1.4-3.3)** | **40089.4 (24449.1-60711.2)** | **1.9 (1.2-2.9)** | **0.16 (-0.11-0.42)** |
| **Colombia** | **86.9 (53.9-130.6)** | **0.5 (0.3-0.8)** | **352.4 (218.8-537.8)** | **0.6 (0.4-1)** | **0.52 (0.49-0.55)** |
| **Comoros** | **1.6 (0.9-2.6)** | **1 (0.6-1.5)** | **4.1 (2.5-6)** | **0.9 (0.6-1.4)** | **-0.06 (-0.1--0.02)** |
| **Congo** | **22.9 (14.1-34.7)** | **2.5 (1.5-3.6)** | **64.7 (40.4-99.8)** | **2.8 (1.7-4.2)** | **0.44 (0.41-0.47)** |
| **Cook Islands** | **0.3 (0.2-0.5)** | **2.7 (1.7-3.9)** | **0.8 (0.5-1.2)** | **2.9 (1.8-4.5)** | **0.3 (0.27-0.33)** |
| **Costa Rica** | **12.8 (8-19)** | **0.8 (0.5-1.1)** | **49.5 (30.7-75.2)** | **0.9 (0.6-1.3)** | **0.67 (0.63-0.71)** |
| **Ivory Coast** | **64.1 (40-99.9)** | **1.9 (1.2-2.9)** | **190.2 (120.3-284.2)** | **1.9 (1.2-2.9)** | **0.1 (0.07-0.14)** |
| **Croatia** | **64.3 (40.2-97.5)** | **1.1 (0.7-1.7)** | **119.9 (73.7-180.8)** | **1.3 (0.8-1.9)** | **0.42 (0.4-0.44)** |
| **Cuba** | **60 (35.4-90)** | **0.6 (0.4-0.9)** | **144 (90.1-220.9)** | **0.7 (0.5-1.1)** | **0.62 (0.56-0.67)** |
| **Cyprus** | **18 (10.9-27.7)** | **2.2 (1.4-3.4)** | **51.1 (32.4-78.8)** | **2.3 (1.5-3.6)** | **0.1 (0.07-0.12)** |
| **Czechia** | **155.7 (94.4-233.9)** | **1.1 (0.7-1.7)** | **278.3 (169.1-416.1)** | **1.2 (0.7-1.8)** | **0.4 (0.17-0.63)** |
| **Democratic People's Republic of Korea** | **310.1 (192.9-466.1)** | **2.2 (1.4-3.3)** | **732.7 (448-1105.4)** | **2.3 (1.4-3.5)** | **0.12 (0.06-0.18)** |
| **Democratic Republic of the Congo** | **325 (203.6-477.9)** | **2.5 (1.6-3.6)** | **789.9 (501.9-1177.1)** | **2.4 (1.5-3.6)** | **-0.21 (-0.29--0.13)** |
| **Denmark** | **173.4 (105.9-266.1)** | **2 (1.2-3)** | **295.7 (180.8-459.7)** | **2.3 (1.4-3.5)** | **0.5 (0.47-0.53)** |
| **Djibouti** | **1 (0.6-1.5)** | **0.9 (0.6-1.4)** | **5.2 (3.2-8)** | **1 (0.6-1.5)** | **0.4 (0.36-0.44)** |
| **Dominica** | **0.4 (0.2-0.6)** | **0.7 (0.4-1)** | **0.7 (0.4-1.1)** | **0.9 (0.5-1.4)** | **0.93 (0.9-0.95)** |
| **Dominican Republic** | **19.9 (11.8-30.9)** | **0.6 (0.4-0.9)** | **74.5 (46.4-112.4)** | **0.8 (0.5-1.1)** | **0.88 (0.82-0.93)** |
| **Ecuador** | **33.1 (20.4-50)** | **0.7 (0.4-1)** | **142.6 (88.3-216.4)** | **0.9 (0.5-1.3)** | **1.25 (1.14-1.36)** |
| **Egypt** | **409.7 (258.7-609.5)** | **1.8 (1.1-2.6)** | **1190 (740.7-1856.3)** | **2.2 (1.4-3.4)** | **0.67 (0.66-0.69)** |
| **El Salvador** | **13.9 (8.5-21)** | **0.5 (0.3-0.7)** | **41.2 (25.7-61.9)** | **0.7 (0.4-1)** | **1.15 (1.11-1.2)** |
| **Equatorial Guinea** | **3.7 (2.4-5.6)** | **2.2 (1.4-3.3)** | **12.2 (7.7-18.6)** | **2.7 (1.7-4.2)** | **0.93 (0.84-1.02)** |
| **Eritrea** | **7.2 (4.4-11.2)** | **0.8 (0.5-1.3)** | **18.9 (11.9-29)** | **0.8 (0.5-1.2)** | **0.04 (0-0.08)** |
| **Estonia** | **31.8 (19.1-47.4)** | **1.6 (0.9-2.3)** | **54.5 (33.1-83.7)** | **1.9 (1.1-2.9)** | **0.71 (0.68-0.73)** |
| **Eswatini** | **6.8 (4.2-10.4)** | **2.6 (1.6-4)** | **13.9 (8.9-21.1)** | **2.8 (1.7-4.1)** | **0.1 (0.07-0.14)** |
| **Ethiopia** | **167.1 (104.4-256.7)** | **1 (0.6-1.5)** | **366 (226.9-564.1)** | **1 (0.6-1.5)** | **-0.12 (-0.17--0.08)** |
| **Fiji** | **8.1 (5.1-12.2)** | **2.6 (1.6-3.9)** | **20.1 (12.5-30.1)** | **2.9 (1.8-4.3)** | **0.31 (0.29-0.32)** |
| **Finland** | **120.5 (73.3-186.4)** | **1.6 (1-2.5)** | **270 (167.6-413.3)** | **1.9 (1.2-2.9)** | **0.46 (0.44-0.49)** |
| **France** | **1426.5 (873.3-2127.2)** | **1.6 (1-2.4)** | **3143.1 (1962.6-4866.7)** | **2 (1.3-3.1)** | **0.52 (0.43-0.6)** |
| **Gabon** | **13.5 (8.5-20.7)** | **2.5 (1.6-3.8)** | **27.9 (17.8-42.8)** | **2.9 (1.9-4.4)** | **0.52 (0.5-0.54)** |
| **Gambia** | **5.5 (3.4-8.4)** | **1.8 (1.1-2.6)** | **16.2 (10.2-24.3)** | **1.8 (1.1-2.7)** | **0.16 (0.14-0.17)** |
| **Georgia** | **129.7 (83.6-194.6)** | **2.1 (1.4-3.2)** | **144.1 (90.9-216.7)** | **2.3 (1.5-3.5)** | **0.34 (0.32-0.35)** |
| **Germany** | **2732.1 (1652-4191.4)** | **2 (1.2-3.1)** | **5362.1 (3217.2-8083)** | **2.5 (1.5-3.8)** | **0.72 (0.66-0.78)** |
| **Ghana** | **54.8 (33.2-83.2)** | **1 (0.6-1.6)** | **162.9 (101.4-247)** | **1.1 (0.7-1.7)** | **0.28 (0.25-0.31)** |
| **Greece** | **378.3 (238.4-587.4)** | **2.4 (1.5-3.7)** | **682.2 (423.8-1033.3)** | **2.5 (1.6-3.8)** | **0.48 (0.04-0.92)** |
| **Greenland** | **1.1 (0.7-1.6)** | **4 (2.5-5.9)** | **3.2 (2-4.8)** | **5.3 (3.3-7.9)** | **1 (0.95-1.04)** |
| **Grenada** | **0.5 (0.3-0.7)** | **0.6 (0.4-1)** | **0.9 (0.5-1.4)** | **0.8 (0.5-1.2)** | **0.7 (0.67-0.74)** |
| **Guam** | **1.6 (1-2.4)** | **2.5 (1.6-3.8)** | **6.1 (3.8-9.1)** | **2.8 (1.8-4.3)** | **0.43 (0.42-0.45)** |
| **Guatemala** | **16.9 (10.5-26.5)** | **0.5 (0.3-0.8)** | **76.7 (48.5-115.9)** | **0.7 (0.4-1.1)** | **0.93 (0.91-0.95)** |
| **Guinea** | **53.8 (34.1-79.6)** | **1.8 (1.1-2.6)** | **93.3 (57.4-140.5)** | **1.8 (1.1-2.8)** | **0.13 (0.09-0.17)** |
| **Guinea-Bissau** | **6.1 (3.8-9.4)** | **1.8 (1.1-2.7)** | **10.7 (6.8-16.1)** | **1.7 (1.1-2.6)** | **-0.11 (-0.14--0.09)** |
| **Guyana** | **2.2 (1.4-3.3)** | **0.6 (0.4-1)** | **4.8 (3-7.2)** | **0.8 (0.5-1.2)** | **0.77 (0.75-0.79)** |
| **Haiti** | **16.6 (10.2-24.9)** | **0.6 (0.4-0.9)** | **43.3 (26.4-64.8)** | **0.7 (0.4-1)** | **0.54 (0.51-0.57)** |
| **Honduras** | **10.5 (6.5-16)** | **0.5 (0.3-0.8)** | **43.4 (26.5-68.2)** | **0.7 (0.4-1.1)** | **0.9 (0.85-0.95)** |
| **Hungary** | **169.3 (102.5-252)** | **1.2 (0.7-1.7)** | **259 (158.5-386)** | **1.3 (0.8-1.9)** | **0.22 (0.2-0.24)** |
| **Iceland** | **5.7 (3.5-8.6)** | **1.9 (1.2-2.9)** | **11.8 (7.3-17.8)** | **1.9 (1.2-2.9)** | **0.16 (0.09-0.23)** |
| **India** | **6535.1 (4144.8-9835.4)** | **1.7 (1.1-2.5)** | **16364.1 (10265.9-24635.3)** | **1.5 (1-2.3)** | **-0.36 (-0.43--0.29)** |
| **Indonesia** | **1660.4 (1059.3-2490)** | **2 (1.2-2.9)** | **4906.9 (3083.4-7434.1)** | **2.3 (1.4-3.5)** | **0.54 (0.51-0.57)** |
| **Iran (Islamic Republic of)** | **495.4 (307.7-754.9)** | **2.1 (1.3-3.2)** | **1684.5 (1051-2535.3)** | **2.3 (1.4-3.5)** | **0.27 (0.21-0.33)** |
| **Iraq** | **137.9 (84-208.8)** | **1.8 (1.1-2.8)** | **407.7 (258.3-616.5)** | **1.9 (1.2-2.8)** | **0.14 (0.1-0.18)** |
| **Ireland** | **105 (65.5-162.9)** | **2.5 (1.5-3.8)** | **232.3 (148.9-353.4)** | **2.8 (1.8-4.3)** | **0.39 (0.37-0.41)** |
| **Israel** | **120.8 (72.4-184.9)** | **2.4 (1.5-3.7)** | **335.9 (210.7-515)** | **2.6 (1.6-3.9)** | **0.19 (0.18-0.2)** |
| **Italy** | **1655.3 (1011.2-2483.1)** | **1.8 (1.1-2.7)** | **2743.7 (1702.6-4127.1)** | **1.7 (1-2.5)** | **0.04 (-0.16-0.24)** |
| **Jamaica** | **12 (7.2-17.8)** | **0.7 (0.4-1)** | **26.7 (16.6-40.3)** | **0.8 (0.5-1.3)** | **0.78 (0.71-0.85)** |
| **Japan** | **4826.6 (2978.5-7240)** | **2.9 (1.8-4.3)** | **12615.8 (7772.9-18799.3)** | **3.1 (1.9-4.7)** | **0.36 (0.33-0.4)** |
| **Jordan** | **21.2 (13.4-32.4)** | **1.8 (1.1-2.7)** | **142.1 (87.7-213.2)** | **2.1 (1.3-3.2)** | **0.55 (0.51-0.58)** |
| **Kazakhstan** | **250 (157.5-378.4)** | **2.1 (1.3-3.2)** | **408.7 (254.2-607.4)** | **2.4 (1.5-3.6)** | **0.5 (0.49-0.52)** |
| **Kenya** | **70 (43.2-106.1)** | **1 (0.6-1.5)** | **194.4 (121.7-296.7)** | **1 (0.6-1.5)** | **0.06 (-0.04-0.15)** |
| **Kiribati** | **0.8 (0.5-1.2)** | **2.4 (1.5-3.6)** | **1.6 (1-2.4)** | **2.5 (1.6-3.8)** | **0.17 (0.15-0.19)** |
| **Kuwait** | **10.4 (6.6-15.5)** | **1.9 (1.2-2.9)** | **52.5 (32.6-78.5)** | **2 (1.2-3.1)** | **0.35 (0.28-0.43)** |
| **Kyrgyzstan** | **60.4 (37.8-89)** | **2.1 (1.3-3.1)** | **101.6 (63.6-153.9)** | **2.3 (1.4-3.4)** | **0.29 (0.27-0.3)** |
| **Lao People's Democratic Republic** | **38.6 (23.6-59)** | **2.1 (1.3-3.1)** | **98.7 (62.2-147.5)** | **2.4 (1.5-3.6)** | **0.49 (0.47-0.52)** |
| **Latvia** | **54.8 (33.6-83.2)** | **1.5 (1-2.3)** | **75.2 (46.1-109.6)** | **1.8 (1.1-2.6)** | **0.52 (0.49-0.54)** |
| **Lebanon** | **31.2 (19.8-47.5)** | **1.6 (1-2.3)** | **106.2 (65.1-158.6)** | **1.7 (1.1-2.6)** | **0.35 (0.3-0.4)** |
| **Lesotho** | **16.4 (10.4-24.9)** | **2.1 (1.3-3.1)** | **23.5 (14.6-35.2)** | **2.4 (1.5-3.5)** | **0.49 (0.44-0.54)** |
| **Liberia** | **20.3 (12.8-30.8)** | **1.9 (1.2-2.9)** | **36.2 (23.1-54.4)** | **2 (1.2-3)** | **0.21 (0.14-0.29)** |
| **Libya** | **30.1 (18.8-45.6)** | **1.7 (1.1-2.6)** | **88.9 (55.4-132.2)** | **1.9 (1.2-2.8)** | **0.34 (0.29-0.38)** |
| **Lithuania** | **71.7 (44.3-107.6)** | **1.6 (1-2.4)** | **105.2 (66.7-160.4)** | **1.7 (1.1-2.6)** | **0.24 (0.22-0.27)** |
| **Luxembourg** | **12 (7.3-18.2)** | **2.1 (1.3-3.2)** | **24 (14.7-37.8)** | **2.2 (1.3-3.4)** | **0.12 (0.08-0.15)** |
| **Madagascar** | **40.3 (24.8-61.3)** | **0.9 (0.6-1.4)** | **79.3 (48.9-119)** | **0.9 (0.6-1.3)** | **-0.04 (-0.11-0.03)** |
| **Malawi** | **30 (18.5-45.5)** | **0.9 (0.6-1.4)** | **58.2 (36.5-88)** | **0.9 (0.6-1.4)** | **0.01 (-0.04-0.06)** |
| **Malaysia** | **213.4 (134.4-321.1)** | **2.5 (1.6-3.8)** | **772.4 (476.3-1163.8)** | **2.9 (1.8-4.4)** | **0.57 (0.55-0.6)** |
| **Maldives** | **1.8 (1.1-2.8)** | **2.5 (1.5-3.8)** | **7.7 (4.8-11.6)** | **2.5 (1.6-3.8)** | **0.07 (0.04-0.11)** |
| **Mali** | **59.1 (37.2-88.5)** | **1.7 (1.1-2.6)** | **141 (88-207)** | **1.8 (1.2-2.7)** | **0.27 (0.24-0.29)** |
| **Malta** | **8.9 (5.5-13.5)** | **2.1 (1.3-3.1)** | **25 (15.4-37.7)** | **2.3 (1.4-3.5)** | **0.24 (0.22-0.26)** |
| **Marshall Islands** | **0.3 (0.2-0.5)** | **2.3 (1.4-3.5)** | **0.8 (0.5-1.2)** | **2.8 (1.7-4.1)** | **0.61 (0.6-0.63)** |
| **Mauritania** | **17.1 (10.9-26.2)** | **1.9 (1.2-2.8)** | **40.5 (26-62.8)** | **2.1 (1.3-3.2)** | **0.35 (0.31-0.38)** |
| **Mauritius** | **15.7 (9.9-23.7)** | **2.3 (1.4-3.5)** | **54.7 (34.9-82.9)** | **3 (1.9-4.5)** | **0.9 (0.88-0.93)** |
| **Mexico** | **261.7 (164.9-393.8)** | **0.7 (0.4-1)** | **996.1 (627.4-1502.6)** | **0.8 (0.5-1.2)** | **1.02 (0.85-1.2)** |
| **Micronesia (Federated States of)** | **1.2 (0.7-1.8)** | **2.7 (1.7-4)** | **2 (1.2-3)** | **3 (1.9-4.5)** | **0.38 (0.35-0.4)** |
| **Monaco** | **1.7 (1-2.5)** | **2.1 (1.3-3.2)** | **2.6 (1.6-3.9)** | **2.3 (1.4-3.6)** | **0.31 (0.3-0.32)** |
| **Mongolia** | **23.2 (14.8-33.8)** | **2.4 (1.5-3.4)** | **49 (30.6-74.9)** | **2.4 (1.5-3.5)** | **0.07 (0.05-0.08)** |
| **Montenegro** | **8.1 (5-12.2)** | **1.4 (0.8-2)** | **13.6 (8.4-20.2)** | **1.4 (0.9-2.1)** | **0.22 (0.17-0.27)** |
| **Morocco** | **162.1 (98.7-241.7)** | **1.2 (0.7-1.8)** | **440.3 (278.5-646.3)** | **1.4 (0.9-2)** | **0.41 (0.35-0.46)** |
| **Mozambique** | **44.2 (27-67)** | **0.9 (0.5-1.3)** | **81.5 (50.7-121.6)** | **0.9 (0.5-1.3)** | **0.04 (-0.01-0.09)** |
| **Myanmar** | **388.4 (241.2-587.8)** | **1.9 (1.2-2.9)** | **918.2 (573.5-1369)** | **2.1 (1.3-3.1)** | **0.29 (0.26-0.32)** |
| **Namibia** | **13.3 (8.2-20)** | **2.3 (1.4-3.4)** | **29.7 (18.3-44.4)** | **2.4 (1.5-3.5)** | **0.13 (0.09-0.16)** |
| **Nauru** | **0.1 (0.1-0.2)** | **3.1 (1.9-4.7)** | **0.1 (0.1-0.2)** | **2.9 (1.8-4.3)** | **-0.17 (-0.28--0.07)** |
| **Nepal** | **195.6 (124.2-295.2)** | **2.4 (1.5-3.6)** | **589.8 (376.9-881.6)** | **2.7 (1.7-4)** | **0.38 (0.34-0.41)** |
| **Netherlands** | **424.3 (257.4-630)** | **2 (1.2-3)** | **869.4 (542.9-1335.9)** | **2.3 (1.4-3.5)** | **0.17 (0.08-0.26)** |
| **New Zealand** | **207.9 (128-310.2)** | **5.1 (3.2-7.6)** | **486.8 (301.7-732.1)** | **5.4 (3.4-8.2)** | **0.24 (0.12-0.36)** |
| **Nicaragua** | **10.6 (6.5-16.5)** | **0.7 (0.4-1.1)** | **49.7 (31.1-72.1)** | **1 (0.6-1.5)** | **1.11 (1.05-1.16)** |
| **Niger** | **40.8 (25.1-61.4)** | **1.7 (1.1-2.6)** | **118.7 (73.8-181.9)** | **1.7 (1-2.5)** | **-0.06 (-0.09--0.03)** |
| **Nigeria** | **1043.1 (667.5-1576.8)** | **2.5 (1.6-3.7)** | **1913.8 (1220.7-2898.1)** | **2.3 (1.5-3.5)** | **-0.25 (-0.37--0.13)** |
| **Niue** | **0.1 (0-0.1)** | **2.5 (1.5-3.8)** | **0.1 (0-0.1)** | **3 (1.9-4.6)** | **0.55 (0.51-0.58)** |
| **North Macedonia** | **26.3 (16.4-39.7)** | **1.5 (0.9-2.3)** | **52.4 (31.2-80.6)** | **1.6 (1-2.5)** | **0.21 (0.19-0.22)** |
| **Northern Mariana Islands** | **0.5 (0.3-0.8)** | **3.5 (2.1-5.2)** | **1.7 (1.1-2.6)** | **3.6 (2.3-5.4)** | **-0.07 (-0.1--0.03)** |
| **Norway** | **108.5 (66.8-163.2)** | **1.4 (0.9-2.2)** | **185.7 (115.7-276.7)** | **1.7 (1.1-2.6)** | **0.37 (0.07-0.67)** |
| **Oman** | **8.7 (5.3-13)** | **1.4 (0.9-2.2)** | **34.9 (21.6-52.3)** | **1.9 (1.2-2.9)** | **1.04 (0.97-1.11)** |
| **Pakistan** | **762.2 (477.2-1134.5)** | **1.5 (0.9-2.2)** | **1933.5 (1207.7-2930.6)** | **1.8 (1.1-2.7)** | **0.76 (0.65-0.86)** |
| **Palau** | **0.3 (0.2-0.4)** | **2.8 (1.8-4.3)** | **0.7 (0.4-1)** | **3.4 (2.1-5)** | **0.47 (0.43-0.52)** |
| **Palestine** | **13.3 (8.4-20.3)** | **1.6 (1-2.5)** | **41.4 (25.7-63.1)** | **1.8 (1.1-2.7)** | **0.19 (0.14-0.25)** |
| **Panama** | **7.5 (4.6-11.4)** | **0.5 (0.3-0.8)** | **31.4 (20-46.7)** | **0.7 (0.4-1)** | **0.96 (0.94-0.97)** |
| **Papua New Guinea** | **29.2 (18-44.2)** | **2.1 (1.3-3.1)** | **90.6 (54.5-137)** | **2.2 (1.4-3.3)** | **0.27 (0.25-0.29)** |
| **Paraguay** | **17.7 (11.1-25.9)** | **0.8 (0.5-1.2)** | **58.9 (37.2-87.8)** | **1 (0.7-1.6)** | **0.72 (0.69-0.75)** |
| **Peru** | **62.7 (39.9-93.7)** | **0.6 (0.3-0.8)** | **216 (131.7-322.6)** | **0.6 (0.4-1)** | **0.57 (0.55-0.59)** |
| **Philippines** | **511.9 (319.2-771.2)** | **2 (1.2-2.9)** | **1820.1 (1146.7-2732.3)** | **2.4 (1.5-3.6)** | **0.67 (0.59-0.75)** |
| **Poland** | **540.4 (337.4-818.5)** | **1.3 (0.8-1.9)** | **975.7 (610.9-1465.4)** | **1.3 (0.8-2)** | **-0.06 (-0.1--0.01)** |
| **Portugal** | **232.3 (140.1-363)** | **1.6 (1-2.5)** | **461 (282.3-703.2)** | **1.7 (1-2.5)** | **0.06 (-0.01-0.12)** |
| **Puerto Rico** | **25.4 (15.6-38.1)** | **0.7 (0.4-1.1)** | **66.2 (40.9-101.9)** | **0.9 (0.5-1.3)** | **0.58 (0.56-0.6)** |
| **Qatar** | **2.2 (1.3-3.2)** | **2.1 (1.3-3.2)** | **20.9 (12.8-30.9)** | **2.3 (1.4-3.5)** | **0.42 (0.37-0.47)** |
| **Republic of Korea** | **608.9 (374.3-923.8)** | **2.4 (1.5-3.6)** | **2389.5 (1482.7-3561.8)** | **2.5 (1.6-3.7)** | **0.1 (0.07-0.13)** |
| **Republic of Moldova** | **74.6 (47.5-111.8)** | **1.8 (1.2-2.7)** | **128.2 (80.8-193)** | **2.1 (1.3-3.2)** | **0.53 (0.5-0.55)** |
| **Romania** | **345.8 (210.7-520.2)** | **1.3 (0.8-2)** | **597.8 (377.1-879.4)** | **1.5 (1-2.3)** | **0.32 (0.24-0.39)** |
| **Russian Federation** | **2264.7 (1409-3409.9)** | **1.3 (0.8-2)** | **3656.7 (2295.7-5444.6)** | **1.5 (0.9-2.3)** | **0.49 (0.47-0.52)** |
| **Rwanda** | **22 (13.6-34.1)** | **0.9 (0.6-1.4)** | **46.4 (28.1-69.2)** | **0.9 (0.5-1.3)** | **-0.21 (-0.27--0.15)** |
| **Saint Kitts and Nevis** | **0.3 (0.2-0.4)** | **0.7 (0.4-1)** | **0.5 (0.3-0.8)** | **0.8 (0.5-1.2)** | **0.54 (0.51-0.56)** |
| **Saint Lucia** | **0.5 (0.3-0.8)** | **0.6 (0.4-1)** | **1.9 (1.2-2.9)** | **0.8 (0.5-1.2)** | **0.83 (0.8-0.87)** |
| **Saint Vincent and the Grenadines** | **0.4 (0.3-0.6)** | **0.6 (0.4-0.9)** | **1.1 (0.7-1.7)** | **0.8 (0.5-1.2)** | **1.08 (1.04-1.13)** |
| **Samoa** | **2.1 (1.4-3.2)** | **2.8 (1.7-4.1)** | **4 (2.5-6)** | **3 (1.9-4.5)** | **0.16 (0.13-0.2)** |
| **San Marino** | **0.8 (0.5-1.2)** | **2.1 (1.3-3.1)** | **1.9 (1.2-2.9)** | **2.2 (1.4-3.4)** | **0.26 (0.24-0.27)** |
| **Sao Tome and Principe** | **1.1 (0.7-1.7)** | **1.8 (1.1-2.7)** | **2.1 (1.4-3.2)** | **2.1 (1.3-3.1)** | **0.51 (0.47-0.54)** |
| **Saudi Arabia** | **100.1 (62.8-149.8)** | **1.9 (1.2-2.8)** | **422.8 (255.5-641.6)** | **2.5 (1.6-3.6)** | **0.93 (0.9-0.96)** |
| **Senegal** | **43.2 (26.8-65.8)** | **1.5 (0.9-2.2)** | **95.9 (58.8-148.7)** | **1.4 (0.9-2.1)** | **-0.28 (-0.35--0.21)** |
| **Serbia** | **101.4 (61-153.7)** | **1 (0.6-1.5)** | **182.8 (109.5-274.3)** | **1.1 (0.6-1.6)** | **0.27 (0.24-0.29)** |
| **Seychelles** | **1.3 (0.8-1.9)** | **2.2 (1.4-3.4)** | **3 (1.9-4.5)** | **2.7 (1.7-4)** | **0.64 (0.61-0.67)** |
| **Sierra Leone** | **32.8 (20.1-49.6)** | **1.7 (1.1-2.6)** | **58.5 (36.1-89.4)** | **1.7 (1.1-2.6)** | **0.06 (0.01-0.1)** |
| **Singapore** | **55.8 (34.4-84.1)** | **2.8 (1.7-4.3)** | **266.8 (168.9-407.8)** | **3.1 (2-4.8)** | **0.29 (0.26-0.32)** |
| **Slovakia** | **70.8 (44.1-107.2)** | **1.2 (0.8-1.8)** | **120 (75.7-181.9)** | **1.2 (0.8-1.9)** | **0.02 (0-0.05)** |
| **Slovenia** | **26.7 (16.1-40.7)** | **1.1 (0.7-1.7)** | **56.9 (34.8-85.9)** | **1.2 (0.7-1.8)** | **0.27 (0.24-0.3)** |
| **Solomon Islands** | **2.8 (1.8-4.1)** | **2.5 (1.6-3.7)** | **7.5 (4.7-11.1)** | **2.6 (1.6-3.8)** | **0.03 (0-0.05)** |
| **Somalia** | **17.4 (10.7-26.3)** | **0.9 (0.6-1.4)** | **40.2 (24.4-62.1)** | **0.8 (0.5-1.3)** | **-0.35 (-0.39--0.31)** |
| **South Africa** | **508.6 (324.6-763.3)** | **2.7 (1.7-4)** | **1256.3 (794.4-1888.2)** | **2.9 (1.8-4.3)** | **0.32 (0.28-0.36)** |
| **South Sudan** | **23.5 (14.2-35.4)** | **1 (0.6-1.5)** | **31.5 (19.1-47.4)** | **1 (0.6-1.5)** | **0.03 (-0.02-0.08)** |
| **Spain** | **1263.4 (778.2-1910.8)** | **2.2 (1.4-3.4)** | **2344 (1470.3-3576.9)** | **2.2 (1.4-3.4)** | **-0.13 (-0.16--0.1)** |
| **Sri Lanka** | **206.2 (129.6-312.9)** | **2.2 (1.3-3.2)** | **583.7 (362-882.7)** | **2.2 (1.4-3.3)** | **0.1 (0.06-0.14)** |
| **Sudan** | **124.4 (76.1-190.3)** | **1.5 (0.9-2.2)** | **307.4 (189.1-470.2)** | **1.8 (1.1-2.7)** | **0.69 (0.65-0.73)** |
| **Suriname** | **1.6 (1-2.5)** | **0.7 (0.4-1)** | **5.1 (3.2-7.6)** | **0.8 (0.5-1.2)** | **0.7 (0.67-0.73)** |
| **Sweden** | **297.6 (179.4-455.8)** | **1.8 (1.1-2.7)** | **468 (286.3-710.8)** | **1.9 (1.1-2.8)** | **0.51 (0.4-0.62)** |
| **Switzerland** | **227 (140.5-347.8)** | **2 (1.3-3.1)** | **448.5 (283.3-673.2)** | **2.3 (1.4-3.4)** | **0.32 (0.28-0.36)** |
| **Syrian Arab Republic** | **82.7 (51.9-126.6)** | **1.7 (1.1-2.6)** | **217.6 (134.9-325.4)** | **1.8 (1.1-2.7)** | **0.19 (0.13-0.24)** |
| **Taiwan (Province of China)** | **484.9 (311.4-712)** | **3.4 (2.2-4.9)** | **1529.8 (983.8-2248.8)** | **3.5 (2.3-5.2)** | **-0.27 (-1.05-0.51)** |
| **Tajikistan** | **47.7 (29.8-71.6)** | **1.9 (1.2-2.8)** | **105.3 (64.9-162.9)** | **2.1 (1.3-3.2)** | **0.48 (0.44-0.52)** |
| **Thailand** | **777 (499.4-1157.4)** | **2.4 (1.6-3.5)** | **3370.9 (2114.8-5058.8)** | **3.1 (1.9-4.6)** | **1.1 (0.97-1.22)** |
| **Timor-Leste** | **4.5 (2.9-6.8)** | **2 (1.2-3)** | **17.1 (10.6-25.9)** | **2.1 (1.4-3.2)** | **0.24 (0.22-0.27)** |
| **Togo** | **18 (11.1-27.1)** | **1.7 (1-2.6)** | **55.2 (34.9-82.2)** | **1.6 (1-2.5)** | **-0.12 (-0.15--0.09)** |
| **Tokelau** | **0 (0-0)** | **2.3 (1.5-3.5)** | **0 (0-0.1)** | **2.7 (1.7-4.1)** | **0.47 (0.46-0.49)** |
| **Tonga** | **1.3 (0.8-2)** | **2.6 (1.6-3.9)** | **2.3 (1.4-3.5)** | **3 (1.9-4.5)** | **0.3 (0.27-0.34)** |
| **Trinidad and Tobago** | **5.4 (3.3-8)** | **0.7 (0.4-1)** | **16.2 (9.8-24.5)** | **0.8 (0.5-1.3)** | **0.81 (0.78-0.83)** |
| **Tunisia** | **72.8 (43.9-109.4)** | **1.6 (1-2.3)** | **217.9 (133.5-328.3)** | **1.7 (1-2.5)** | **0.28 (0.26-0.31)** |
| **Turkey** | **535.8 (326-808.9)** | **1.7 (1-2.5)** | **1589.7 (1001.2-2420.1)** | **1.7 (1.1-2.6)** | **0.18 (0.12-0.24)** |
| **Turkmenistan** | **39.2 (24.7-59.1)** | **2.2 (1.4-3.4)** | **98.8 (60.6-148.4)** | **2.7 (1.7-4)** | **0.64 (0.6-0.68)** |
| **Tuvalu** | **0.1 (0.1-0.2)** | **2.2 (1.4-3.3)** | **0.3 (0.2-0.4)** | **2.7 (1.7-4)** | **0.58 (0.55-0.61)** |
| **Uganda** | **56.3 (34.6-88.9)** | **1 (0.6-1.6)** | **122.9 (75.8-186.5)** | **1 (0.6-1.4)** | **-0.01 (-0.09-0.08)** |
| **Ukraine** | **1092.9 (679.1-1627.1)** | **1.6 (1-2.3)** | **1376.8 (855-2080.1)** | **1.7 (1.1-2.6)** | **0.39 (0.35-0.42)** |
| **United Arab Emirates** | **9.6 (5.8-14.3)** | **2.2 (1.4-3.4)** | **128.3 (77-200)** | **3 (1.8-4.6)** | **1.05 (0.99-1.1)** |
| **United Kingdom** | **2402.4 (1473.7-3605.7)** | **2.5 (1.5-3.7)** | **3720.7 (2293.4-5560.5)** | **2.6 (1.6-4)** | **0.67 (0.41-0.93)** |
| **United Republic of Tanzania** | **82.3 (50.2-126.9)** | **0.9 (0.5-1.4)** | **204.9 (128.4-302.7)** | **0.9 (0.6-1.4)** | **0.19 (0.13-0.25)** |
| **United States of America** | **13807.6 (8538.6-20519)** | **4.1 (2.6-6.1)** | **44353.6 (28907.7-64911.9)** | **7.3 (4.8-10.6)** | **3.15 (2.7-3.61)** |
| **United States Virgin Islands** | **0.5 (0.3-0.8)** | **0.7 (0.4-1)** | **1.6 (1-2.5)** | **0.9 (0.5-1.3)** | **0.84 (0.78-0.89)** |
| **Uruguay** | **99.6 (61.9-156.9)** | **2.5 (1.6-3.9)** | **164.8 (101.3-247.5)** | **2.8 (1.7-4.2)** | **0.32 (0.3-0.34)** |
| **Uzbekistan** | **253.4 (157.1-372.1)** | **2.3 (1.4-3.4)** | **631.1 (396.5-961.2)** | **2.6 (1.7-4)** | **0.47 (0.45-0.49)** |
| **Vanuatu** | **1.3 (0.8-2)** | **2.4 (1.5-3.7)** | **4 (2.4-6)** | **2.7 (1.6-3.9)** | **0.31 (0.29-0.33)** |
| **Venezuela (Bolivarian Republic of)** | **52.2 (32.6-77.4)** | **0.6 (0.4-0.9)** | **208.3 (132.4-317.8)** | **0.7 (0.4-1.1)** | **0.73 (0.7-0.76)** |
| **Viet Nam** | **467.7 (283.9-687.7)** | **1.2 (0.8-1.8)** | **1144.7 (712.7-1707.3)** | **1.3 (0.8-1.9)** | **0.34 (0.2-0.47)** |
| **Yemen** | **55.8 (34.9-84.9)** | **1.3 (0.8-2)** | **179.7 (110.4-270)** | **1.5 (0.9-2.2)** | **0.48 (0.44-0.52)** |
| **Zambia** | **25 (15.4-38.5)** | **1.1 (0.6-1.6)** | **58.1 (36.2-89.2)** | **1 (0.6-1.5)** | **-0.13 (-0.19--0.07)** |
| **Zimbabwe** | **88 (54.3-133.8)** | **2.4 (1.5-3.6)** | **140.4 (85.2-217.4)** | **2.4 (1.4-3.6)** | **-0.05 (-0.08--0.02)** |

**Appendix 9：The DALYs rate and DALYs cases for gout due to renal dysfunction across different age groups globally in 1990**

| **measure** | **location** | **sex** | **age** | **cause** | **rei** | **metric** | **year** | **val** | **upper** | **lower** |
| --- | --- | --- | --- | --- | --- | --- | --- | --- | --- | --- |
| **DALYs (Disability-Adjusted Life Years)** | **Global** | **Male** | **25-29 years** | **Gout** | **Kidney dysfunction** | **Number** | **1990** | **135.8505492** | **228.4917847** | **62.55216834** |
| **DALYs (Disability-Adjusted Life Years)** | **Global** | **Female** | **25-29 years** | **Gout** | **Kidney dysfunction** | **Number** | **1990** | **43.60641522** | **75.161777** | **18.27481163** |
| **DALYs (Disability-Adjusted Life Years)** | **Global** | **Both** | **25-29 years** | **Gout** | **Kidney dysfunction** | **Number** | **1990** | **179.4569644** | **302.301204** | **80.56467917** |
| **DALYs (Disability-Adjusted Life Years)** | **Global** | **Male** | **25-29 years** | **Gout** | **Kidney dysfunction** | **Rate** | **1990** | **0.061050918** | **0.102683671** | **0.028110797** |
| **DALYs (Disability-Adjusted Life Years)** | **Global** | **Female** | **25-29 years** | **Gout** | **Kidney dysfunction** | **Rate** | **1990** | **0.019812131** | **0.034148988** | **0.008302975** |
| **DALYs (Disability-Adjusted Life Years)** | **Global** | **Both** | **25-29 years** | **Gout** | **Kidney dysfunction** | **Rate** | **1990** | **0.040544283** | **0.068298188** | **0.018201785** |
| **YLDs (Years Lived with Disability)** | **Global** | **Male** | **25-29 years** | **Gout** | **Kidney dysfunction** | **Number** | **1990** | **135.8505492** | **228.4917847** | **62.55216834** |
| **YLDs (Years Lived with Disability)** | **Global** | **Female** | **25-29 years** | **Gout** | **Kidney dysfunction** | **Number** | **1990** | **43.60641522** | **75.161777** | **18.27481163** |
| **YLDs (Years Lived with Disability)** | **Global** | **Both** | **25-29 years** | **Gout** | **Kidney dysfunction** | **Number** | **1990** | **179.4569644** | **302.301204** | **80.56467917** |
| **YLDs (Years Lived with Disability)** | **Global** | **Male** | **25-29 years** | **Gout** | **Kidney dysfunction** | **Rate** | **1990** | **0.061050918** | **0.102683671** | **0.028110797** |
| **YLDs (Years Lived with Disability)** | **Global** | **Female** | **25-29 years** | **Gout** | **Kidney dysfunction** | **Rate** | **1990** | **0.019812131** | **0.034148988** | **0.008302975** |
| **YLDs (Years Lived with Disability)** | **Global** | **Both** | **25-29 years** | **Gout** | **Kidney dysfunction** | **Rate** | **1990** | **0.040544283** | **0.068298188** | **0.018201785** |
| **DALYs (Disability-Adjusted Life Years)** | **Global** | **Male** | **30-34 years** | **Gout** | **Kidney dysfunction** | **Number** | **1990** | **316.7155015** | **502.3464278** | **154.3155678** |
| **DALYs (Disability-Adjusted Life Years)** | **Global** | **Female** | **30-34 years** | **Gout** | **Kidney dysfunction** | **Number** | **1990** | **92.17687497** | **153.8900424** | **41.50254349** |
| **DALYs (Disability-Adjusted Life Years)** | **Global** | **Both** | **30-34 years** | **Gout** | **Kidney dysfunction** | **Number** | **1990** | **408.8923765** | **647.5587363** | **193.175255** |
| **DALYs (Disability-Adjusted Life Years)** | **Global** | **Male** | **30-34 years** | **Gout** | **Kidney dysfunction** | **Rate** | **1990** | **0.162157553** | **0.257200127** | **0.079009189** |
| **DALYs (Disability-Adjusted Life Years)** | **Global** | **Female** | **30-34 years** | **Gout** | **Kidney dysfunction** | **Rate** | **1990** | **0.048486318** | **0.080948302** | **0.021830915** |
| **DALYs (Disability-Adjusted Life Years)** | **Global** | **Both** | **30-34 years** | **Gout** | **Kidney dysfunction** | **Rate** | **1990** | **0.106089393** | **0.168012702** | **0.05012039** |
| **YLDs (Years Lived with Disability)** | **Global** | **Male** | **30-34 years** | **Gout** | **Kidney dysfunction** | **Number** | **1990** | **316.7155015** | **502.3464278** | **154.3155678** |
| **YLDs (Years Lived with Disability)** | **Global** | **Female** | **30-34 years** | **Gout** | **Kidney dysfunction** | **Number** | **1990** | **92.17687497** | **153.8900424** | **41.50254349** |
| **YLDs (Years Lived with Disability)** | **Global** | **Both** | **30-34 years** | **Gout** | **Kidney dysfunction** | **Number** | **1990** | **408.8923765** | **647.5587363** | **193.175255** |
| **YLDs (Years Lived with Disability)** | **Global** | **Male** | **30-34 years** | **Gout** | **Kidney dysfunction** | **Rate** | **1990** | **0.162157553** | **0.257200127** | **0.079009189** |
| **YLDs (Years Lived with Disability)** | **Global** | **Female** | **30-34 years** | **Gout** | **Kidney dysfunction** | **Rate** | **1990** | **0.048486318** | **0.080948302** | **0.021830915** |
| **YLDs (Years Lived with Disability)** | **Global** | **Both** | **30-34 years** | **Gout** | **Kidney dysfunction** | **Rate** | **1990** | **0.106089393** | **0.168012702** | **0.05012039** |
| **DALYs (Disability-Adjusted Life Years)** | **Global** | **Male** | **35-39 years** | **Gout** | **Kidney dysfunction** | **Number** | **1990** | **640.6857339** | **1037.036995** | **344.6342572** |
| **DALYs (Disability-Adjusted Life Years)** | **Global** | **Female** | **35-39 years** | **Gout** | **Kidney dysfunction** | **Number** | **1990** | **175.5622546** | **294.6634573** | **88.03358891** |
| **DALYs (Disability-Adjusted Life Years)** | **Global** | **Both** | **35-39 years** | **Gout** | **Kidney dysfunction** | **Number** | **1990** | **816.2479885** | **1330.641681** | **428.6008787** |
| **DALYs (Disability-Adjusted Life Years)** | **Global** | **Male** | **35-39 years** | **Gout** | **Kidney dysfunction** | **Rate** | **1990** | **0.358343066** | **0.580026987** | **0.192757993** |
| **DALYs (Disability-Adjusted Life Years)** | **Global** | **Female** | **35-39 years** | **Gout** | **Kidney dysfunction** | **Rate** | **1990** | **0.101215858** | **0.169880563** | **0.050753479** |
| **DALYs (Disability-Adjusted Life Years)** | **Global** | **Both** | **35-39 years** | **Gout** | **Kidney dysfunction** | **Rate** | **1990** | **0.231727693** | **0.377760842** | **0.121677106** |
| **YLDs (Years Lived with Disability)** | **Global** | **Male** | **35-39 years** | **Gout** | **Kidney dysfunction** | **Number** | **1990** | **640.6857339** | **1037.036995** | **344.6342572** |
| **YLDs (Years Lived with Disability)** | **Global** | **Female** | **35-39 years** | **Gout** | **Kidney dysfunction** | **Number** | **1990** | **175.5622546** | **294.6634573** | **88.03358891** |
| **YLDs (Years Lived with Disability)** | **Global** | **Both** | **35-39 years** | **Gout** | **Kidney dysfunction** | **Number** | **1990** | **816.2479885** | **1330.641681** | **428.6008787** |
| **YLDs (Years Lived with Disability)** | **Global** | **Male** | **35-39 years** | **Gout** | **Kidney dysfunction** | **Rate** | **1990** | **0.358343066** | **0.580026987** | **0.192757993** |
| **YLDs (Years Lived with Disability)** | **Global** | **Female** | **35-39 years** | **Gout** | **Kidney dysfunction** | **Rate** | **1990** | **0.101215858** | **0.169880563** | **0.050753479** |
| **YLDs (Years Lived with Disability)** | **Global** | **Both** | **35-39 years** | **Gout** | **Kidney dysfunction** | **Rate** | **1990** | **0.231727693** | **0.377760842** | **0.121677106** |
| **DALYs (Disability-Adjusted Life Years)** | **Global** | **Male** | **40-44 years** | **Gout** | **Kidney dysfunction** | **Number** | **1990** | **1056.740834** | **1839.213857** | **531.7900012** |
| **DALYs (Disability-Adjusted Life Years)** | **Global** | **Female** | **40-44 years** | **Gout** | **Kidney dysfunction** | **Number** | **1990** | **284.4922345** | **499.2444578** | **141.5532509** |
| **DALYs (Disability-Adjusted Life Years)** | **Global** | **Both** | **40-44 years** | **Gout** | **Kidney dysfunction** | **Number** | **1990** | **1341.233068** | **2345.895338** | **667.7904082** |
| **DALYs (Disability-Adjusted Life Years)** | **Global** | **Male** | **40-44 years** | **Gout** | **Kidney dysfunction** | **Rate** | **1990** | **0.722527318** | **1.25752901** | **0.363601737** |
| **DALYs (Disability-Adjusted Life Years)** | **Global** | **Female** | **40-44 years** | **Gout** | **Kidney dysfunction** | **Rate** | **1990** | **0.202882015** | **0.356029829** | **0.100946899** |
| **DALYs (Disability-Adjusted Life Years)** | **Global** | **Both** | **40-44 years** | **Gout** | **Kidney dysfunction** | **Rate** | **1990** | **0.468174188** | **0.818864127** | **0.233100599** |
| **YLDs (Years Lived with Disability)** | **Global** | **Male** | **40-44 years** | **Gout** | **Kidney dysfunction** | **Number** | **1990** | **1056.740834** | **1839.213857** | **531.7900012** |
| **YLDs (Years Lived with Disability)** | **Global** | **Female** | **40-44 years** | **Gout** | **Kidney dysfunction** | **Number** | **1990** | **284.4922345** | **499.2444578** | **141.5532509** |
| **YLDs (Years Lived with Disability)** | **Global** | **Both** | **40-44 years** | **Gout** | **Kidney dysfunction** | **Number** | **1990** | **1341.233068** | **2345.895338** | **667.7904082** |
| **YLDs (Years Lived with Disability)** | **Global** | **Male** | **40-44 years** | **Gout** | **Kidney dysfunction** | **Rate** | **1990** | **0.722527318** | **1.25752901** | **0.363601737** |
| **YLDs (Years Lived with Disability)** | **Global** | **Female** | **40-44 years** | **Gout** | **Kidney dysfunction** | **Rate** | **1990** | **0.202882015** | **0.356029829** | **0.100946899** |
| **YLDs (Years Lived with Disability)** | **Global** | **Both** | **40-44 years** | **Gout** | **Kidney dysfunction** | **Rate** | **1990** | **0.468174188** | **0.818864127** | **0.233100599** |
| **DALYs (Disability-Adjusted Life Years)** | **Global** | **Male** | **45-49 years** | **Gout** | **Kidney dysfunction** | **Number** | **1990** | **1684.314071** | **2878.695333** | **847.4882284** |
| **DALYs (Disability-Adjusted Life Years)** | **Global** | **Female** | **45-49 years** | **Gout** | **Kidney dysfunction** | **Number** | **1990** | **477.2095732** | **816.4270254** | **230.8736115** |
| **DALYs (Disability-Adjusted Life Years)** | **Global** | **Both** | **45-49 years** | **Gout** | **Kidney dysfunction** | **Number** | **1990** | **2161.523644** | **3686.459694** | **1075.874475** |
| **DALYs (Disability-Adjusted Life Years)** | **Global** | **Male** | **45-49 years** | **Gout** | **Kidney dysfunction** | **Rate** | **1990** | **1.422624786** | **2.431436869** | **0.715815287** |
| **DALYs (Disability-Adjusted Life Years)** | **Global** | **Female** | **45-49 years** | **Gout** | **Kidney dysfunction** | **Rate** | **1990** | **0.4193381** | **0.717418462** | **0.202875439** |
| **DALYs (Disability-Adjusted Life Years)** | **Global** | **Both** | **45-49 years** | **Gout** | **Kidney dysfunction** | **Rate** | **1990** | **0.930906807** | **1.587653427** | **0.463348562** |
| **YLDs (Years Lived with Disability)** | **Global** | **Male** | **45-49 years** | **Gout** | **Kidney dysfunction** | **Number** | **1990** | **1684.314071** | **2878.695333** | **847.4882284** |
| **YLDs (Years Lived with Disability)** | **Global** | **Female** | **45-49 years** | **Gout** | **Kidney dysfunction** | **Number** | **1990** | **477.2095732** | **816.4270254** | **230.8736115** |
| **YLDs (Years Lived with Disability)** | **Global** | **Both** | **45-49 years** | **Gout** | **Kidney dysfunction** | **Number** | **1990** | **2161.523644** | **3686.459694** | **1075.874475** |
| **YLDs (Years Lived with Disability)** | **Global** | **Male** | **45-49 years** | **Gout** | **Kidney dysfunction** | **Rate** | **1990** | **1.422624786** | **2.431436869** | **0.715815287** |
| **YLDs (Years Lived with Disability)** | **Global** | **Female** | **45-49 years** | **Gout** | **Kidney dysfunction** | **Rate** | **1990** | **0.4193381** | **0.717418462** | **0.202875439** |
| **YLDs (Years Lived with Disability)** | **Global** | **Both** | **45-49 years** | **Gout** | **Kidney dysfunction** | **Rate** | **1990** | **0.930906807** | **1.587653427** | **0.463348562** |
| **DALYs (Disability-Adjusted Life Years)** | **Global** | **Male** | **50-54 years** | **Gout** | **Kidney dysfunction** | **Number** | **1990** | **2795.392627** | **4701.833426** | **1527.988605** |
| **DALYs (Disability-Adjusted Life Years)** | **Global** | **Female** | **50-54 years** | **Gout** | **Kidney dysfunction** | **Number** | **1990** | **840.5140794** | **1427.746206** | **441.4369623** |
| **DALYs (Disability-Adjusted Life Years)** | **Global** | **Both** | **50-54 years** | **Gout** | **Kidney dysfunction** | **Number** | **1990** | **3635.906707** | **6140.456723** | **1971.060512** |
| **DALYs (Disability-Adjusted Life Years)** | **Global** | **Male** | **50-54 years** | **Gout** | **Kidney dysfunction** | **Rate** | **1990** | **2.596629483** | **4.367515023** | **1.419342751** |
| **DALYs (Disability-Adjusted Life Years)** | **Global** | **Female** | **50-54 years** | **Gout** | **Kidney dysfunction** | **Rate** | **1990** | **0.801124337** | **1.360836493** | **0.420749518** |
| **DALYs (Disability-Adjusted Life Years)** | **Global** | **Both** | **50-54 years** | **Gout** | **Kidney dysfunction** | **Rate** | **1990** | **1.710439673** | **2.888655193** | **0.92724604** |
| **YLDs (Years Lived with Disability)** | **Global** | **Male** | **50-54 years** | **Gout** | **Kidney dysfunction** | **Number** | **1990** | **2795.392627** | **4701.833426** | **1527.988605** |
| **YLDs (Years Lived with Disability)** | **Global** | **Female** | **50-54 years** | **Gout** | **Kidney dysfunction** | **Number** | **1990** | **840.5140794** | **1427.746206** | **441.4369623** |
| **YLDs (Years Lived with Disability)** | **Global** | **Both** | **50-54 years** | **Gout** | **Kidney dysfunction** | **Number** | **1990** | **3635.906707** | **6140.456723** | **1971.060512** |
| **YLDs (Years Lived with Disability)** | **Global** | **Male** | **50-54 years** | **Gout** | **Kidney dysfunction** | **Rate** | **1990** | **2.596629483** | **4.367515023** | **1.419342751** |
| **YLDs (Years Lived with Disability)** | **Global** | **Female** | **50-54 years** | **Gout** | **Kidney dysfunction** | **Rate** | **1990** | **0.801124337** | **1.360836493** | **0.420749518** |
| **YLDs (Years Lived with Disability)** | **Global** | **Both** | **50-54 years** | **Gout** | **Kidney dysfunction** | **Rate** | **1990** | **1.710439673** | **2.888655193** | **0.92724604** |
| **DALYs (Disability-Adjusted Life Years)** | **Global** | **Male** | **55-59 years** | **Gout** | **Kidney dysfunction** | **Number** | **1990** | **4231.533211** | **6840.09179** | **2402.402455** |
| **DALYs (Disability-Adjusted Life Years)** | **Global** | **Female** | **55-59 years** | **Gout** | **Kidney dysfunction** | **Number** | **1990** | **1374.830241** | **2217.838592** | **770.8503855** |
| **DALYs (Disability-Adjusted Life Years)** | **Global** | **Both** | **55-59 years** | **Gout** | **Kidney dysfunction** | **Number** | **1990** | **5606.363452** | **9058.671495** | **3202.554075** |
| **DALYs (Disability-Adjusted Life Years)** | **Global** | **Male** | **55-59 years** | **Gout** | **Kidney dysfunction** | **Rate** | **1990** | **4.555520226** | **7.363802892** | **2.586342215** |
| **DALYs (Disability-Adjusted Life Years)** | **Global** | **Female** | **55-59 years** | **Gout** | **Kidney dysfunction** | **Rate** | **1990** | **1.489331151** | **2.402548331** | **0.835049635** |
| **DALYs (Disability-Adjusted Life Years)** | **Global** | **Both** | **55-59 years** | **Gout** | **Kidney dysfunction** | **Rate** | **1990** | **3.027194761** | **4.891292391** | **1.729241246** |
| **YLDs (Years Lived with Disability)** | **Global** | **Male** | **55-59 years** | **Gout** | **Kidney dysfunction** | **Number** | **1990** | **4231.533211** | **6840.09179** | **2402.402455** |
| **YLDs (Years Lived with Disability)** | **Global** | **Female** | **55-59 years** | **Gout** | **Kidney dysfunction** | **Number** | **1990** | **1374.830241** | **2217.838592** | **770.8503855** |
| **YLDs (Years Lived with Disability)** | **Global** | **Both** | **55-59 years** | **Gout** | **Kidney dysfunction** | **Number** | **1990** | **5606.363452** | **9058.671495** | **3202.554075** |
| **YLDs (Years Lived with Disability)** | **Global** | **Male** | **55-59 years** | **Gout** | **Kidney dysfunction** | **Rate** | **1990** | **4.555520226** | **7.363802892** | **2.586342215** |
| **YLDs (Years Lived with Disability)** | **Global** | **Female** | **55-59 years** | **Gout** | **Kidney dysfunction** | **Rate** | **1990** | **1.489331151** | **2.402548331** | **0.835049635** |
| **YLDs (Years Lived with Disability)** | **Global** | **Both** | **55-59 years** | **Gout** | **Kidney dysfunction** | **Rate** | **1990** | **3.027194761** | **4.891292391** | **1.729241246** |
| **DALYs (Disability-Adjusted Life Years)** | **Global** | **Male** | **60-64 years** | **Gout** | **Kidney dysfunction** | **Number** | **1990** | **6388.386359** | **10453.58335** | **3587.679468** |
| **DALYs (Disability-Adjusted Life Years)** | **Global** | **Female** | **60-64 years** | **Gout** | **Kidney dysfunction** | **Number** | **1990** | **2324.248965** | **3816.265408** | **1284.26642** |
| **DALYs (Disability-Adjusted Life Years)** | **Global** | **Both** | **60-64 years** | **Gout** | **Kidney dysfunction** | **Number** | **1990** | **8712.635325** | **14216.98975** | **4861.577124** |
| **DALYs (Disability-Adjusted Life Years)** | **Global** | **Male** | **60-64 years** | **Gout** | **Kidney dysfunction** | **Rate** | **1990** | **8.133294515** | **13.30884943** | **4.567609425** |
| **DALYs (Disability-Adjusted Life Years)** | **Global** | **Female** | **60-64 years** | **Gout** | **Kidney dysfunction** | **Rate** | **1990** | **2.832269525** | **4.650402076** | **1.56497376** |
| **DALYs (Disability-Adjusted Life Years)** | **Global** | **Both** | **60-64 years** | **Gout** | **Kidney dysfunction** | **Rate** | **1990** | **5.424741128** | **8.851912901** | **3.026959856** |
| **YLDs (Years Lived with Disability)** | **Global** | **Male** | **60-64 years** | **Gout** | **Kidney dysfunction** | **Number** | **1990** | **6388.386359** | **10453.58335** | **3587.679468** |
| **YLDs (Years Lived with Disability)** | **Global** | **Female** | **60-64 years** | **Gout** | **Kidney dysfunction** | **Number** | **1990** | **2324.248965** | **3816.265408** | **1284.26642** |
| **YLDs (Years Lived with Disability)** | **Global** | **Both** | **60-64 years** | **Gout** | **Kidney dysfunction** | **Number** | **1990** | **8712.635325** | **14216.98975** | **4861.577124** |
| **YLDs (Years Lived with Disability)** | **Global** | **Male** | **60-64 years** | **Gout** | **Kidney dysfunction** | **Rate** | **1990** | **8.133294515** | **13.30884943** | **4.567609425** |
| **YLDs (Years Lived with Disability)** | **Global** | **Female** | **60-64 years** | **Gout** | **Kidney dysfunction** | **Rate** | **1990** | **2.832269525** | **4.650402076** | **1.56497376** |
| **YLDs (Years Lived with Disability)** | **Global** | **Both** | **60-64 years** | **Gout** | **Kidney dysfunction** | **Rate** | **1990** | **5.424741128** | **8.851912901** | **3.026959856** |
| **DALYs (Disability-Adjusted Life Years)** | **Global** | **Male** | **65-69 years** | **Gout** | **Kidney dysfunction** | **Number** | **1990** | **8381.052212** | **13669.27481** | **4913.906674** |
| **DALYs (Disability-Adjusted Life Years)** | **Global** | **Female** | **65-69 years** | **Gout** | **Kidney dysfunction** | **Number** | **1990** | **3604.465409** | **5994.031503** | **2058.773335** |
| **DALYs (Disability-Adjusted Life Years)** | **Global** | **Both** | **65-69 years** | **Gout** | **Kidney dysfunction** | **Number** | **1990** | **11985.51762** | **19849.96142** | **7084.277157** |
| **DALYs (Disability-Adjusted Life Years)** | **Global** | **Male** | **65-69 years** | **Gout** | **Kidney dysfunction** | **Rate** | **1990** | **14.6186541** | **23.84263875** | **8.57108393** |
| **DALYs (Disability-Adjusted Life Years)** | **Global** | **Female** | **65-69 years** | **Gout** | **Kidney dysfunction** | **Rate** | **1990** | **5.438384239** | **9.04373957** | **3.106258261** |
| **DALYs (Disability-Adjusted Life Years)** | **Global** | **Both** | **65-69 years** | **Gout** | **Kidney dysfunction** | **Rate** | **1990** | **9.696278813** | **16.05861061** | **5.731177299** |
| **YLDs (Years Lived with Disability)** | **Global** | **Male** | **65-69 years** | **Gout** | **Kidney dysfunction** | **Number** | **1990** | **8381.052212** | **13669.27481** | **4913.906674** |
| **YLDs (Years Lived with Disability)** | **Global** | **Female** | **65-69 years** | **Gout** | **Kidney dysfunction** | **Number** | **1990** | **3604.465409** | **5994.031503** | **2058.773335** |
| **YLDs (Years Lived with Disability)** | **Global** | **Both** | **65-69 years** | **Gout** | **Kidney dysfunction** | **Number** | **1990** | **11985.51762** | **19849.96142** | **7084.277157** |
| **YLDs (Years Lived with Disability)** | **Global** | **Male** | **65-69 years** | **Gout** | **Kidney dysfunction** | **Rate** | **1990** | **14.6186541** | **23.84263875** | **8.57108393** |
| **YLDs (Years Lived with Disability)** | **Global** | **Female** | **65-69 years** | **Gout** | **Kidney dysfunction** | **Rate** | **1990** | **5.438384239** | **9.04373957** | **3.106258261** |
| **YLDs (Years Lived with Disability)** | **Global** | **Both** | **65-69 years** | **Gout** | **Kidney dysfunction** | **Rate** | **1990** | **9.696278813** | **16.05861061** | **5.731177299** |
| **DALYs (Disability-Adjusted Life Years)** | **Global** | **Male** | **70-74 years** | **Gout** | **Kidney dysfunction** | **Number** | **1990** | **8900.94084** | **14343.6898** | **5056.638203** |
| **DALYs (Disability-Adjusted Life Years)** | **Global** | **Female** | **70-74 years** | **Gout** | **Kidney dysfunction** | **Number** | **1990** | **4477.971288** | **7083.875074** | **2501.081849** |
| **DALYs (Disability-Adjusted Life Years)** | **Global** | **Both** | **70-74 years** | **Gout** | **Kidney dysfunction** | **Number** | **1990** | **13378.91213** | **21169.56484** | **7593.21672** |
| **DALYs (Disability-Adjusted Life Years)** | **Global** | **Male** | **70-74 years** | **Gout** | **Kidney dysfunction** | **Rate** | **1990** | **23.66106534** | **38.12933796** | **13.44188767** |
| **DALYs (Disability-Adjusted Life Years)** | **Global** | **Female** | **70-74 years** | **Gout** | **Kidney dysfunction** | **Rate** | **1990** | **9.518962164** | **15.0584125** | **5.316627097** |
| **DALYs (Disability-Adjusted Life Years)** | **Global** | **Both** | **70-74 years** | **Gout** | **Kidney dysfunction** | **Rate** | **1990** | **15.80289355** | **25.00505097** | **8.968950118** |
| **YLDs (Years Lived with Disability)** | **Global** | **Male** | **70-74 years** | **Gout** | **Kidney dysfunction** | **Number** | **1990** | **8900.94084** | **14343.6898** | **5056.638203** |
| **YLDs (Years Lived with Disability)** | **Global** | **Female** | **70-74 years** | **Gout** | **Kidney dysfunction** | **Number** | **1990** | **4477.971288** | **7083.875074** | **2501.081849** |
| **YLDs (Years Lived with Disability)** | **Global** | **Both** | **70-74 years** | **Gout** | **Kidney dysfunction** | **Number** | **1990** | **13378.91213** | **21169.56484** | **7593.21672** |
| **YLDs (Years Lived with Disability)** | **Global** | **Male** | **70-74 years** | **Gout** | **Kidney dysfunction** | **Rate** | **1990** | **23.66106534** | **38.12933796** | **13.44188767** |
| **YLDs (Years Lived with Disability)** | **Global** | **Female** | **70-74 years** | **Gout** | **Kidney dysfunction** | **Rate** | **1990** | **9.518962164** | **15.0584125** | **5.316627097** |
| **YLDs (Years Lived with Disability)** | **Global** | **Both** | **70-74 years** | **Gout** | **Kidney dysfunction** | **Rate** | **1990** | **15.80289355** | **25.00505097** | **8.968950118** |
| **DALYs (Disability-Adjusted Life Years)** | **Global** | **Male** | **75-79 years** | **Gout** | **Kidney dysfunction** | **Number** | **1990** | **8414.609168** | **12945.10854** | **4804.156291** |
| **DALYs (Disability-Adjusted Life Years)** | **Global** | **Female** | **75-79 years** | **Gout** | **Kidney dysfunction** | **Number** | **1990** | **5152.212764** | **7921.688652** | **2887.934663** |
| **DALYs (Disability-Adjusted Life Years)** | **Global** | **Both** | **75-79 years** | **Gout** | **Kidney dysfunction** | **Number** | **1990** | **13566.82193** | **20887.70051** | **7691.877497** |
| **DALYs (Disability-Adjusted Life Years)** | **Global** | **Male** | **75-79 years** | **Gout** | **Kidney dysfunction** | **Rate** | **1990** | **33.3478599** | **51.30264013** | **19.03930744** |
| **DALYs (Disability-Adjusted Life Years)** | **Global** | **Female** | **75-79 years** | **Gout** | **Kidney dysfunction** | **Rate** | **1990** | **14.1845599** | **21.80920556** | **7.950774572** |
| **DALYs (Disability-Adjusted Life Years)** | **Global** | **Both** | **75-79 years** | **Gout** | **Kidney dysfunction** | **Rate** | **1990** | **22.03997818** | **33.93311019** | **12.49583823** |
| **YLDs (Years Lived with Disability)** | **Global** | **Male** | **75-79 years** | **Gout** | **Kidney dysfunction** | **Number** | **1990** | **8414.609168** | **12945.10854** | **4804.156291** |
| **YLDs (Years Lived with Disability)** | **Global** | **Female** | **75-79 years** | **Gout** | **Kidney dysfunction** | **Number** | **1990** | **5152.212764** | **7921.688652** | **2887.934663** |
| **YLDs (Years Lived with Disability)** | **Global** | **Both** | **75-79 years** | **Gout** | **Kidney dysfunction** | **Number** | **1990** | **13566.82193** | **20887.70051** | **7691.877497** |
| **YLDs (Years Lived with Disability)** | **Global** | **Male** | **75-79 years** | **Gout** | **Kidney dysfunction** | **Rate** | **1990** | **33.3478599** | **51.30264013** | **19.03930744** |
| **YLDs (Years Lived with Disability)** | **Global** | **Female** | **75-79 years** | **Gout** | **Kidney dysfunction** | **Rate** | **1990** | **14.1845599** | **21.80920556** | **7.950774572** |
| **YLDs (Years Lived with Disability)** | **Global** | **Both** | **75-79 years** | **Gout** | **Kidney dysfunction** | **Rate** | **1990** | **22.03997818** | **33.93311019** | **12.49583823** |
| **DALYs (Disability-Adjusted Life Years)** | **Global** | **Male** | **80-84 years** | **Gout** | **Kidney dysfunction** | **Number** | **1990** | **5646.838174** | **9022.973154** | **3221.648393** |
| **DALYs (Disability-Adjusted Life Years)** | **Global** | **Female** | **80-84 years** | **Gout** | **Kidney dysfunction** | **Number** | **1990** | **4215.93708** | **6680.348572** | **2440.478745** |
| **DALYs (Disability-Adjusted Life Years)** | **Global** | **Both** | **80-84 years** | **Gout** | **Kidney dysfunction** | **Number** | **1990** | **9862.775255** | **15651.15549** | **5627.261629** |
| **DALYs (Disability-Adjusted Life Years)** | **Global** | **Male** | **80-84 years** | **Gout** | **Kidney dysfunction** | **Rate** | **1990** | **42.51036128** | **67.92648146** | **24.2531188** |
| **DALYs (Disability-Adjusted Life Years)** | **Global** | **Female** | **80-84 years** | **Gout** | **Kidney dysfunction** | **Rate** | **1990** | **19.08312529** | **30.23810041** | **11.04664533** |
| **DALYs (Disability-Adjusted Life Years)** | **Global** | **Both** | **80-84 years** | **Gout** | **Kidney dysfunction** | **Rate** | **1990** | **27.87990603** | **44.24238949** | **15.90703644** |

**Appendix 10：****The DALYs rate and DALYs cases for gout due to renal dysfunction across different age groups globally in 2021.**

| **measure** | **location** | **sex** | **age** | **cause** | **rei** | **metric** | **year** | **val** | **upper** | **lower** |
| --- | --- | --- | --- | --- | --- | --- | --- | --- | --- | --- |
| **DALYs (Disability-Adjusted Life Years)** | **Global** | **Male** | **75-79 years** | **Gout** | **Kidney dysfunction** | **Number** | **2021** | **22143.89921** | **33795.28086** | **12689.17368** |
| **DALYs (Disability-Adjusted Life Years)** | **Global** | **Female** | **75-79 years** | **Gout** | **Kidney dysfunction** | **Number** | **2021** | **11294.23964** | **17079.50191** | **6484.238692** |
| **DALYs (Disability-Adjusted Life Years)** | **Global** | **Both** | **75-79 years** | **Gout** | **Kidney dysfunction** | **Number** | **2021** | **33438.13884** | **50889.21864** | **19304.74746** |
| **DALYs (Disability-Adjusted Life Years)** | **Global** | **Male** | **75-79 years** | **Gout** | **Kidney dysfunction** | **Rate** | **2021** | **37.03815501** | **56.52639761** | **21.22406615** |
| **DALYs (Disability-Adjusted Life Years)** | **Global** | **Female** | **75-79 years** | **Gout** | **Kidney dysfunction** | **Rate** | **2021** | **15.66519119** | **23.68939136** | **8.993685464** |
| **DALYs (Disability-Adjusted Life Years)** | **Global** | **Both** | **75-79 years** | **Gout** | **Kidney dysfunction** | **Rate** | **2021** | **25.35412656** | **38.58622921** | **14.63762719** |
| **DALYs (Disability-Adjusted Life Years)** | **Global** | **Male** | **80-84 years** | **Gout** | **Kidney dysfunction** | **Number** | **2021** | **17406.85186** | **27424.36202** | **9921.099759** |
| **DALYs (Disability-Adjusted Life Years)** | **Global** | **Female** | **80-84 years** | **Gout** | **Kidney dysfunction** | **Number** | **2021** | **10598.01856** | **16820.38978** | **6157.841236** |
| **DALYs (Disability-Adjusted Life Years)** | **Global** | **Both** | **80-84 years** | **Gout** | **Kidney dysfunction** | **Number** | **2021** | **28004.87042** | **44037.12734** | **16106.35228** |
| **DALYs (Disability-Adjusted Life Years)** | **Global** | **Male** | **80-84 years** | **Gout** | **Kidney dysfunction** | **Rate** | **2021** | **47.49240777** | **74.82392537** | **27.06847391** |
| **DALYs (Disability-Adjusted Life Years)** | **Global** | **Female** | **80-84 years** | **Gout** | **Kidney dysfunction** | **Rate** | **2021** | **20.80850179** | **33.02571218** | **12.09051009** |
| **DALYs (Disability-Adjusted Life Years)** | **Global** | **Both** | **80-84 years** | **Gout** | **Kidney dysfunction** | **Rate** | **2021** | **31.97521462** | **50.28041826** | **18.38980374** |
| **DALYs (Disability-Adjusted Life Years)** | **Global** | **Male** | **85-89 years** | **Gout** | **Kidney dysfunction** | **Number** | **2021** | **9833.01435** | **15353.82639** | **5946.114045** |
| **DALYs (Disability-Adjusted Life Years)** | **Global** | **Female** | **85-89 years** | **Gout** | **Kidney dysfunction** | **Number** | **2021** | **7334.088046** | **11240.68262** | **4520.158211** |
| **DALYs (Disability-Adjusted Life Years)** | **Global** | **Both** | **85-89 years** | **Gout** | **Kidney dysfunction** | **Number** | **2021** | **17167.1024** | **26523.41873** | **10540.65443** |
| **DALYs (Disability-Adjusted Life Years)** | **Global** | **Male** | **85-89 years** | **Gout** | **Kidney dysfunction** | **Rate** | **2021** | **56.99419472** | **88.99396867** | **34.46491276** |
| **DALYs (Disability-Adjusted Life Years)** | **Global** | **Female** | **85-89 years** | **Gout** | **Kidney dysfunction** | **Rate** | **2021** | **25.76154468** | **39.48375665** | **15.87740111** |
| **DALYs (Disability-Adjusted Life Years)** | **Global** | **Both** | **85-89 years** | **Gout** | **Kidney dysfunction** | **Rate** | **2021** | **37.54687207** | **58.01045435** | **23.0538966** |
| **DALYs (Disability-Adjusted Life Years)** | **Global** | **Male** | **90-94 years** | **Gout** | **Kidney dysfunction** | **Number** | **2021** | **3831.115892** | **6152.129379** | **2313.12965** |
| **DALYs (Disability-Adjusted Life Years)** | **Global** | **Female** | **90-94 years** | **Gout** | **Kidney dysfunction** | **Number** | **2021** | **3695.739828** | **6032.707318** | **2233.694841** |
| **DALYs (Disability-Adjusted Life Years)** | **Global** | **Both** | **90-94 years** | **Gout** | **Kidney dysfunction** | **Number** | **2021** | **7526.85572** | **12198.49838** | **4559.783435** |
| **DALYs (Disability-Adjusted Life Years)** | **Global** | **Male** | **90-94 years** | **Gout** | **Kidney dysfunction** | **Rate** | **2021** | **65.73074611** | **105.5525506** | **39.68654095** |
| **DALYs (Disability-Adjusted Life Years)** | **Global** | **Female** | **90-94 years** | **Gout** | **Kidney dysfunction** | **Rate** | **2021** | **30.64238672** | **50.01882145** | **18.52017304** |
| **DALYs (Disability-Adjusted Life Years)** | **Global** | **Both** | **90-94 years** | **Gout** | **Kidney dysfunction** | **Rate** | **2021** | **42.07445097** | **68.18851605** | **25.488782** |
| **DALYs (Disability-Adjusted Life Years)** | **Global** | **Male** | **25-29 years** | **Gout** | **Kidney dysfunction** | **Number** | **2021** | **160.8374092** | **271.1857439** | **77.53992599** |
| **DALYs (Disability-Adjusted Life Years)** | **Global** | **Female** | **25-29 years** | **Gout** | **Kidney dysfunction** | **Number** | **2021** | **48.54017649** | **83.75650914** | **20.25123647** |
| **DALYs (Disability-Adjusted Life Years)** | **Global** | **Both** | **25-29 years** | **Gout** | **Kidney dysfunction** | **Number** | **2021** | **209.3775857** | **354.4073909** | **100.4087** |
| **DALYs (Disability-Adjusted Life Years)** | **Global** | **Male** | **25-29 years** | **Gout** | **Kidney dysfunction** | **Rate** | **2021** | **0.054089185** | **0.091199031** | **0.026076467** |
| **DALYs (Disability-Adjusted Life Years)** | **Global** | **Female** | **25-29 years** | **Gout** | **Kidney dysfunction** | **Rate** | **2021** | **0.016681203** | **0.028783565** | **0.006959492** |
| **DALYs (Disability-Adjusted Life Years)** | **Global** | **Both** | **25-29 years** | **Gout** | **Kidney dysfunction** | **Rate** | **2021** | **0.03558766** | **0.060238204** | **0.017066348** |
| **DALYs (Disability-Adjusted Life Years)** | **Global** | **Male** | **30-34 years** | **Gout** | **Kidney dysfunction** | **Number** | **2021** | **461.8000612** | **732.1873937** | **222.1867852** |
| **DALYs (Disability-Adjusted Life Years)** | **Global** | **Female** | **30-34 years** | **Gout** | **Kidney dysfunction** | **Number** | **2021** | **125.3633242** | **209.8190163** | **54.73397163** |
| **DALYs (Disability-Adjusted Life Years)** | **Global** | **Both** | **30-34 years** | **Gout** | **Kidney dysfunction** | **Number** | **2021** | **587.1633854** | **928.7040959** | **275.2722223** |
| **DALYs (Disability-Adjusted Life Years)** | **Global** | **Male** | **30-34 years** | **Gout** | **Kidney dysfunction** | **Rate** | **2021** | **0.151137659** | **0.239629869** | **0.072717163** |
| **DALYs (Disability-Adjusted Life Years)** | **Global** | **Female** | **30-34 years** | **Gout** | **Kidney dysfunction** | **Rate** | **2021** | **0.041937229** | **0.070189812** | **0.018309909** |
| **DALYs (Disability-Adjusted Life Years)** | **Global** | **Both** | **30-34 years** | **Gout** | **Kidney dysfunction** | **Rate** | **2021** | **0.097135259** | **0.153636816** | **0.045538668** |
| **DALYs (Disability-Adjusted Life Years)** | **Global** | **Male** | **35-39 years** | **Gout** | **Kidney dysfunction** | **Number** | **2021** | **970.1532288** | **1578.410131** | **527.1068833** |
| **DALYs (Disability-Adjusted Life Years)** | **Global** | **Female** | **35-39 years** | **Gout** | **Kidney dysfunction** | **Number** | **2021** | **246.177615** | **409.0747754** | **126.2446068** |
| **DALYs (Disability-Adjusted Life Years)** | **Global** | **Both** | **35-39 years** | **Gout** | **Kidney dysfunction** | **Number** | **2021** | **1216.330844** | **2001.95451** | **650.5913533** |
| **DALYs (Disability-Adjusted Life Years)** | **Global** | **Male** | **35-39 years** | **Gout** | **Kidney dysfunction** | **Rate** | **2021** | **0.34273358** | **0.557617229** | **0.18621515** |
| **DALYs (Disability-Adjusted Life Years)** | **Global** | **Female** | **35-39 years** | **Gout** | **Kidney dysfunction** | **Rate** | **2021** | **0.08861598** | **0.14725369** | **0.045443976** |
| **DALYs (Disability-Adjusted Life Years)** | **Global** | **Both** | **35-39 years** | **Gout** | **Kidney dysfunction** | **Rate** | **2021** | **0.216866527** | **0.356939827** | **0.115997623** |
| **DALYs (Disability-Adjusted Life Years)** | **Global** | **Male** | **40-44 years** | **Gout** | **Kidney dysfunction** | **Number** | **2021** | **1824.487873** | **3086.999429** | **944.6212591** |
| **DALYs (Disability-Adjusted Life Years)** | **Global** | **Female** | **40-44 years** | **Gout** | **Kidney dysfunction** | **Number** | **2021** | **461.6276543** | **827.5537382** | **231.6489186** |
| **DALYs (Disability-Adjusted Life Years)** | **Global** | **Both** | **40-44 years** | **Gout** | **Kidney dysfunction** | **Number** | **2021** | **2286.115528** | **3933.733208** | **1183.690183** |
| **DALYs (Disability-Adjusted Life Years)** | **Global** | **Male** | **40-44 years** | **Gout** | **Kidney dysfunction** | **Rate** | **2021** | **0.723541047** | **1.224217947** | **0.374610467** |
| **DALYs (Disability-Adjusted Life Years)** | **Global** | **Female** | **40-44 years** | **Gout** | **Kidney dysfunction** | **Rate** | **2021** | **0.186072762** | **0.333570158** | **0.093372989** |
| **DALYs (Disability-Adjusted Life Years)** | **Global** | **Both** | **40-44 years** | **Gout** | **Kidney dysfunction** | **Rate** | **2021** | **0.456993881** | **0.786352214** | **0.23661935** |
| **DALYs (Disability-Adjusted Life Years)** | **Global** | **Male** | **45-49 years** | **Gout** | **Kidney dysfunction** | **Number** | **2021** | **3438.885777** | **5807.046821** | **1783.640319** |
| **DALYs (Disability-Adjusted Life Years)** | **Global** | **Female** | **45-49 years** | **Gout** | **Kidney dysfunction** | **Number** | **2021** | **912.9538437** | **1581.792716** | **450.5289383** |
| **DALYs (Disability-Adjusted Life Years)** | **Global** | **Both** | **45-49 years** | **Gout** | **Kidney dysfunction** | **Number** | **2021** | **4351.839621** | **7360.543939** | **2232.230431** |
| **DALYs (Disability-Adjusted Life Years)** | **Global** | **Male** | **45-49 years** | **Gout** | **Kidney dysfunction** | **Rate** | **2021** | **1.445747981** | **2.441350705** | **0.749863345** |
| **DALYs (Disability-Adjusted Life Years)** | **Global** | **Female** | **45-49 years** | **Gout** | **Kidney dysfunction** | **Rate** | **2021** | **0.387431617** | **0.67126779** | **0.191191653** |
| **DALYs (Disability-Adjusted Life Years)** | **Global** | **Both** | **45-49 years** | **Gout** | **Kidney dysfunction** | **Rate** | **2021** | **0.919070138** | **1.554481949** | **0.471427376** |
| **DALYs (Disability-Adjusted Life Years)** | **Global** | **Male** | **50-54 years** | **Gout** | **Kidney dysfunction** | **Number** | **2021** | **5945.706626** | **10044.59388** | **3268.743743** |
| **DALYs (Disability-Adjusted Life Years)** | **Global** | **Female** | **50-54 years** | **Gout** | **Kidney dysfunction** | **Number** | **2021** | **1659.967581** | **2835.288848** | **870.5483456** |
| **DALYs (Disability-Adjusted Life Years)** | **Global** | **Both** | **50-54 years** | **Gout** | **Kidney dysfunction** | **Number** | **2021** | **7605.674208** | **12913.02846** | **4143.048663** |
| **DALYs (Disability-Adjusted Life Years)** | **Global** | **Male** | **50-54 years** | **Gout** | **Kidney dysfunction** | **Rate** | **2021** | **2.678482514** | **4.524991016** | **1.472536993** |
| **DALYs (Disability-Adjusted Life Years)** | **Global** | **Female** | **50-54 years** | **Gout** | **Kidney dysfunction** | **Rate** | **2021** | **0.744571844** | **1.271757515** | **0.390480992** |
| **DALYs (Disability-Adjusted Life Years)** | **Global** | **Both** | **50-54 years** | **Gout** | **Kidney dysfunction** | **Rate** | **2021** | **1.709436127** | **2.902306458** | **0.931183333** |
| **DALYs (Disability-Adjusted Life Years)** | **Global** | **Male** | **55-59 years** | **Gout** | **Kidney dysfunction** | **Number** | **2021** | **9233.172551** | **14891.28616** | **5352.216044** |
| **DALYs (Disability-Adjusted Life Years)** | **Global** | **Female** | **55-59 years** | **Gout** | **Kidney dysfunction** | **Number** | **2021** | **2846.106558** | **4630.796748** | **1617.836644** |
| **DALYs (Disability-Adjusted Life Years)** | **Global** | **Both** | **55-59 years** | **Gout** | **Kidney dysfunction** | **Number** | **2021** | **12079.27911** | **19528.69152** | **7074.644786** |
| **DALYs (Disability-Adjusted Life Years)** | **Global** | **Male** | **55-59 years** | **Gout** | **Kidney dysfunction** | **Rate** | **2021** | **4.74165988** | **7.647362132** | **2.748609749** |
| **DALYs (Disability-Adjusted Life Years)** | **Global** | **Female** | **55-59 years** | **Gout** | **Kidney dysfunction** | **Rate** | **2021** | **1.415948588** | **2.303838589** | **0.804879742** |
| **DALYs (Disability-Adjusted Life Years)** | **Global** | **Both** | **55-59 years** | **Gout** | **Kidney dysfunction** | **Rate** | **2021** | **3.052419588** | **4.93487732** | **1.787754396** |
| **DALYs (Disability-Adjusted Life Years)** | **Global** | **Male** | **60-64 years** | **Gout** | **Kidney dysfunction** | **Number** | **2021** | **13654.51788** | **22115.23948** | **7810.079113** |
| **DALYs (Disability-Adjusted Life Years)** | **Global** | **Female** | **60-64 years** | **Gout** | **Kidney dysfunction** | **Number** | **2021** | **4654.01161** | **7597.582335** | **2585.015248** |
| **DALYs (Disability-Adjusted Life Years)** | **Global** | **Both** | **60-64 years** | **Gout** | **Kidney dysfunction** | **Number** | **2021** | **18308.52949** | **29639.05233** | **10381.91606** |
| **DALYs (Disability-Adjusted Life Years)** | **Global** | **Male** | **60-64 years** | **Gout** | **Kidney dysfunction** | **Rate** | **2021** | **8.778956048** | **14.21864302** | **5.021366692** |
| **DALYs (Disability-Adjusted Life Years)** | **Global** | **Female** | **60-64 years** | **Gout** | **Kidney dysfunction** | **Rate** | **2021** | **2.828998369** | **4.618284146** | **1.571333407** |
| **DALYs (Disability-Adjusted Life Years)** | **Global** | **Both** | **60-64 years** | **Gout** | **Kidney dysfunction** | **Rate** | **2021** | **5.720560003** | **9.260818977** | **3.243863676** |
| **DALYs (Disability-Adjusted Life Years)** | **Global** | **Male** | **65-69 years** | **Gout** | **Kidney dysfunction** | **Number** | **2021** | **20475.22193** | **33196.78218** | **12175.18616** |
| **DALYs (Disability-Adjusted Life Years)** | **Global** | **Female** | **65-69 years** | **Gout** | **Kidney dysfunction** | **Number** | **2021** | **7998.764376** | **13104.54383** | **4621.286607** |
| **DALYs (Disability-Adjusted Life Years)** | **Global** | **Both** | **65-69 years** | **Gout** | **Kidney dysfunction** | **Number** | **2021** | **28473.98631** | **46160.69359** | **16942.7881** |
| **DALYs (Disability-Adjusted Life Years)** | **Global** | **Male** | **65-69 years** | **Gout** | **Kidney dysfunction** | **Rate** | **2021** | **15.5311532** | **25.18088993** | **9.235293374** |
| **DALYs (Disability-Adjusted Life Years)** | **Global** | **Female** | **65-69 years** | **Gout** | **Kidney dysfunction** | **Rate** | **2021** | **5.554353346** | **9.099813849** | **3.209027985** |
| **DALYs (Disability-Adjusted Life Years)** | **Global** | **Both** | **65-69 years** | **Gout** | **Kidney dysfunction** | **Rate** | **2021** | **10.32256507** | **16.73445924** | **6.142204001** |
| **DALYs (Disability-Adjusted Life Years)** | **Global** | **Male** | **70-74 years** | **Gout** | **Kidney dysfunction** | **Number** | **2021** | **25120.97588** | **39780.6681** | **14509.61231** |
| **DALYs (Disability-Adjusted Life Years)** | **Global** | **Female** | **70-74 years** | **Gout** | **Kidney dysfunction** | **Number** | **2021** | **11162.39191** | **17833.9525** | **6356.785053** |
| **DALYs (Disability-Adjusted Life Years)** | **Global** | **Both** | **70-74 years** | **Gout** | **Kidney dysfunction** | **Number** | **2021** | **36283.36779** | **57020.28245** | **20805.79741** |
| **DALYs (Disability-Adjusted Life Years)** | **Global** | **Male** | **70-74 years** | **Gout** | **Kidney dysfunction** | **Rate** | **2021** | **26.06142526** | **41.26992971** | **15.05280602** |
| **DALYs (Disability-Adjusted Life Years)** | **Global** | **Female** | **70-74 years** | **Gout** | **Kidney dysfunction** | **Rate** | **2021** | **10.19882637** | **16.29448119** | **5.808051494** |
| **DALYs (Disability-Adjusted Life Years)** | **Global** | **Both** | **70-74 years** | **Gout** | **Kidney dysfunction** | **Rate** | **2021** | **17.62704295** | **27.70136923** | **10.10779062** |
| **DALYs (Disability-Adjusted Life Years)** | **Global** | **Male** | **95+ years** | **Gout** | **Kidney dysfunction** | **Number** | **2021** | **1107.423021** | **1803.458986** | **652.7953634** |
| **DALYs (Disability-Adjusted Life Years)** | **Global** | **Female** | **95+ years** | **Gout** | **Kidney dysfunction** | **Number** | **2021** | **1387.283565** | **2263.367279** | **812.9281765** |
| **DALYs (Disability-Adjusted Life Years)** | **Global** | **Both** | **95+ years** | **Gout** | **Kidney dysfunction** | **Number** | **2021** | **2494.706587** | **4084.63494** | **1466.546046** |
| **DALYs (Disability-Adjusted Life Years)** | **Global** | **Male** | **95+ years** | **Gout** | **Kidney dysfunction** | **Rate** | **2021** | **73.24023458** | **119.2730842** | **43.17310065** |
| **DALYs (Disability-Adjusted Life Years)** | **Global** | **Female** | **95+ years** | **Gout** | **Kidney dysfunction** | **Rate** | **2021** | **35.22573859** | **57.4711516** | **20.64177516** |
| **DALYs (Disability-Adjusted Life Years)** | **Global** | **Both** | **95+ years** | **Gout** | **Kidney dysfunction** | **Rate** | **2021** | **45.77184038** | **74.9431855** | **26.90757777** |

**Appendix 11: The global DALY cases of gout attributable to kidney dysfunction across different age groups from 1990 to 2021.**

| **measure** | **location** | **sex** | **age** | **cause** | **rei** | **metric** | **year** | **val** | **upper** | **lower** |
| --- | --- | --- | --- | --- | --- | --- | --- | --- | --- | --- |
| **DALYs (Disability-Adjusted Life Years)** | **Global** | **Both** | **25-29 years** | **Gout** | **Kidney dysfunction** | **Number** | **1990** | **179.4569644** | **302.301204** | **80.56467917** |
| **DALYs (Disability-Adjusted Life Years)** | **Global** | **Both** | **25-29 years** | **Gout** | **Kidney dysfunction** | **Number** | **1991** | **183.9612227** | **304.9747868** | **84.25106353** |
[truncated: 684,073 more chars]
